# *Cotesia vestalis* teratocytes express a diversity of genes and exhibit novel immune functions in parasitism

**DOI:** 10.1038/srep26967

**Published:** 2016-06-02

**Authors:** Fei Gao, Qi-juan Gu, Jing Pan, Ze-hua Wang, Chuan-lin Yin, Fei Li, Qi-sheng Song, Michael R. Strand, Xue-xin Chen, Min Shi

**Affiliations:** 1 State Key Laboratory of Rice Biology and Ministry of Agriculture Key Laboratory of Agricultural Entomology, Institute of Insect Sciences, Zhejiang University, 866 Yuhangtang Road, Hangzhou 310058, China; 2 Department of Entomology, College of Plant Protection, Nanjing Agricultural University and Key Lab of Monitoring and Management of Plant Diseases and Insects, Ministry of Agriculture, 1 Weigang Road, Nanjing, Jiangsu 210095, China; 3 Molecular Insect Physiology, Division of Plant Sciences, University of Missouri, Columbia, Missouri 65211, USA; 4 Department of Entomology, University of Georgia, Athens, Georgia 30602, USA

## Abstract

Some endoparasitoid wasps lay eggs that produce cells called teratocytes. In this study, we sequenced and analyzed the transcriptome of teratocytes from the solitary endoparasitoid *Cotesia vestalis* (Braconidae), which parasitizes larval stage *Plutella xylostella* (Plutellidae). Results identified many teratocyte transcripts with potential functions in affecting host immune defenses, growth or metabolism. Characterization of teratocyte-secreted venom-like protein 8 (TSVP-8) indicated it inhibits melanization of host hemolymph *in vitro*, while two predicted anti-microbial peptides (CvT-def 1 and 3) inhibited the growth of bacteria. Results also showed the parasitized hosts lacking teratocytes experienced higher mortality after immune challenge by pathogens than hosts with teratocytes. Taken together, these findings indicate that *C. vestalis* teratocytes secrete products that alter host immune functions while also producing anti-microbial peptides with functions that help protect the host from infection by other organisms.

Some species of endoparasitic wasps lay eggs that produce cells called teratoctyes^1^. Teratocytes originate from cellular membranes that envelope the wasp embryo, and are released into the host when the egg hatches^2^,^3^,^4^. Teratocytes increase greatly in size and ploidy over the period required for wasp larvae to develop in hosts but rarely divide^5^,^6^,^7^,^8^. Teratocytes also commonly have numerous microvilli on their surface, which are thought to play roles in uptake or secretion of molecules^7^,^8^,^9^,^10^,^11^,^12^,^13^,^14^,^15^.

Teratocytes from some wasp species produce factors that promote the growth of parasitoid larvae^3^ or serve as a source of nutrients that parasitoid larvae consume^16^. Teratocytes have also been reported to attach to the surface of host tissues, like the fat body, which results in extraoral digestion and release of nutrients that wasp larvae consume^11^,^17^. Teratocytes produced by *Aphidius ervi* secrete a fatty acid binding protein (FABP) and an enolase that are hypothesized to promote growth of parasitoid larvae^18^,^19^,^20^,^21^ while teratocytes from *Dinocampus coccinellae* synthesize a storage-like protein that may have nutritional functions^16^.

Other studies report that teratocytes secrete factors, which alter the physiology of hosts for the benefit of wasp larvae. For example, injection of *Microplitis croceipes* teratocytes into *Heliothis virescens* larvae reduces juvenile hormone esterase (JHE) and arylphorin levels, which is associated with delays in pupation^22^,^23^,^24^,^25^,^26^. Injection of teratocytes from *Toxoneuron nigriceps* and *Microplitis demolitor* also disrupt pupation^5^,^27^, while experiments with teratocytes from *Cotesia congregata* elevate host hemolymph JH titers^6^. Delayed pupation caused by *M. croceipes* teratocytes is attributed at least in part to secretion of a 14 kDa teratocyte-secreted protein (TSP-14) that inhibits translation of some host proteins^28^. Injection of secretory products from *Cotesia plutellae* teratocytes likewise reduces the expression of the ecdysone receptor in final instar *Plutella xylostella*^29^.

Teratocytes have also been reported to alter host immune responses^1^,^30^. *Cotesia kariyai* teratocytes alter hemocyte activity and reduce encapsulation of wasp larvae during the early stages of parasitism^12^. Later in parasitism, teratocytes from this species reduce hemolymph phenoloxidase (PO) activity by producing a putative PO inhibitor^12^. In contrast, young teratocytes from *Cotesia glomerata* inhibit hemolymph PO activity in *Pieris rapae crucivora*^31^. Lastly, several lines of evidence suggest teratocytes interact with other factors wasps introduce into hosts, such as polydnaviruses (PDVs) and venom, because only when teratocytes, PDVs and venom are injected together do hosts exhibit immune alterations that are similar to naturally parasitized hosts^1^,^5^,^32^.

Two recent studies report transcriptome data for teratocytes from *Microplitis demolitor* and *Cotesia plutellae* that were produced in primary culture^33^,^34^. However, it is also possible that the host environment affects teratocyte gene expression. We therefore conducted a transcriptome analysis of teratocytes from *Cotesia vestalis* collected directly from parasitized *Plutella xylostella* larvae. Our *de novo* assembly identified more than 11,000 transcripts expressed in *C. vestalis* teratocytes. Functional experiments further support roles for specific teratocyte products in parasitism.

## Results

### Transcriptome sequencing and annotation

*C. vestalis* teratocytes were collected from parasitized *P. xylostella* larvae at two day intervals (1, 3, and 5 days post-parasitism) to produce a pooled cDNA library that was sequenced using the Illumina platform. This yielded a total of 6,967,361 reads that after quality filtering and *de novo* assembly identified 11,924 teratocyte loci (unigenes) with an average length of 491 bp (see Supplementary Table S1, Fig. S1A). Searches against the NCBI nucleotide non-redundant (Nr) database identified 8,072 of these unigenes as homologs of known genes while 3,852 were novel. The top match for 16.06% of the unigenes (first hit) was sequences from *Drosophila melanogaster*, followed by *Megachile rotundata* (14.95%), *Nasonia vitripennis* (13.20%), *Harpegnathos saltator* (8.27%), *Camponotus floridanus* (7.35%) and *Acromyrmex echinatior* (6.80%) (see Supplementary Fig. S1B). Unigenes for the *C. vestalis* teratocyte transcriptome that were homologs of known genes were further annotated using the gene ontology (GO) and clusters of orthologous groups (COG) databases (see Supplementary Fig. S1).

**Figure 1:**
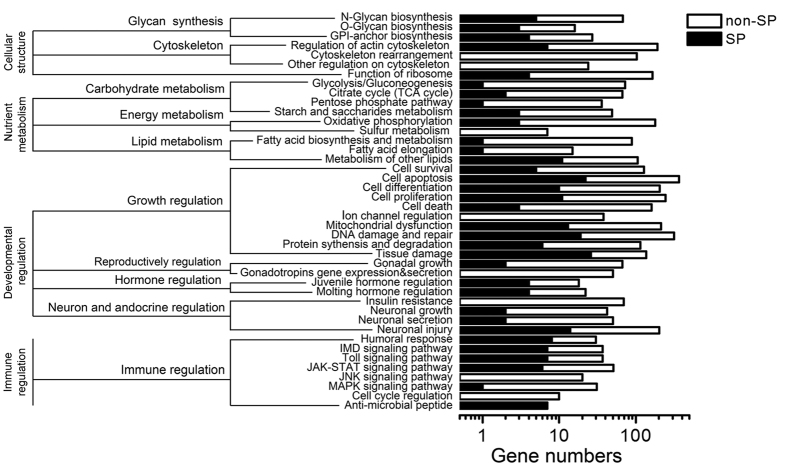
KEGG classification of annotated teratocyte genes. SP: unigenes with signal peptides; Non-SP: unigenes without signal peptides.

Searches against the Kyoto Encyclopedia of Genes and Genomes (KEGG) and Insect Innate Immunity Databases (IIID) mapped 6,134 unigenes to 244 pathways. These included 645 genes in pathways associated with glycan biosynthesis and the cytoskeleton, and 1,502 genes that mapped to pathways associated with nutrient metabolism (carbohydrate, energy and lipid metabolism), regulation of development (growth, reproductive system, hormone, and neuron and endocrine regulation), and immunity (Fig. 1). Several unigenes with potential functions in protein biosynthesis and degradation, regulation of juvenile hormone and ecdysteroid titers, apoptosis, mitochondrial dysfunction, and neuronal growth were also identified. We analyzed each unigene for the presence of a signal peptide by examining corresponding open reading frames (ORFs) from genomic data we recently developed for *C. vestalis* (Genbank Accession number LQNH00000000). This analysis identified 461 unigenes with signal peptides that were also homologs of known genes (see Supplementary Table S2) of which 308 could be assigned to KEGG functional groups (Fig. 1). Overall, a majority of *C. vestalis* teratocyte unigenes that were homologs of known genes belonged to four functional groups: regulation of host development, regulation of host immunity, nutrient metabolism, and cellular structure (Fig. 1, see Supplementary Table S3).

### Unigenes with potential roles in known teratocyte functions

As previously noted, several studies in the literature identify teratocytes as having roles in altering development and immunity of parasitized hosts. Results of this study identified 12 unigenes expressed in *C. vestalis* teratocytes for predicted neurohormones, proteins with functions in biosynthesis of ecdysone, metabolism of juvenile hormone, or chitin metabolism (Fig. 1). Several immune gene homologs were also identified. These included transcripts that were similar to proteins that have been detected in hymenopteran venoms (venom protein Vn4.6, venom protein Vn50, venom protein-like PO inhibitors) and transcripts for certain anti-microbial peptides (AMPs) such as defensins, crustin and hymenoptaecin. Several transcripts also corresponded to immune recognition proteins (peptidoglycan-recognition protein-LF (PGRP-LF), a C-type lectin (CTL), adhesion molecules (integrins), and components of the Relish, Toll and mitogen-activated protein kinase 1 (MAPK-1) pathways that are known regulators of the insect immune system (Fig. 1).

Other teratocyte transcripts of interest were genes with predicted functions in formation of microvilli or metabolism (see Supplementary Table S3). These included supervillin, Moesin/ezrin/radixin homolog 1 and plastin-3 genes, which are all known components of microvilli, and FABP, matrix metalloproteinase 14 (MMP-14), maltase, trehalase, vitellogenin and a hexamerin-like proteins that have likely metabolic, enzymatic, and nutritional functions.

**Figure 2:**
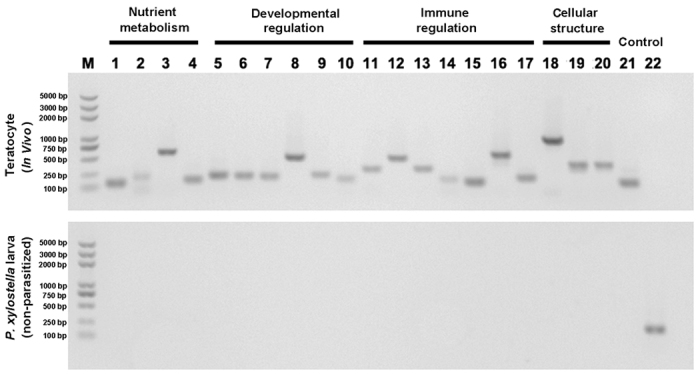
RT-PCR analysis of select teratocyte transcripts. M: marker. Lanes 1–4: *Tre*, *FABP*, *MMP-14*, *HexL*. Lanes 5–10: *JHE*, *TSP-13*, *TSVP-GGCT*, *VEP-2*, *VEP-8*, *Chi-3*. Lanes11–17: *TSVP-8*, *TSVP-42*, *CTL*, *CvT-def 1*, *CvT-def 2*, *TSVP-allergen*, *TSVP-SEP*. Lanes 18–20: *Sup*, *MERH-1*, *Pla-3*. Lane 21: negative control (*β-tubulin* of *P. xylostella*). Lane 22: positive control (*18 S rRNA* of *C. vestalis*).

We selected a subset of the above transcripts and used RT-PCR to confirm each was a teratocyte product and not a contaminant from *P. xylostella* RNA. The 20 transcripts we analyzed also had predicted functions related to nutrition metabolism, regulation of host development, regulation of immunity, and cellular structure (see Supplementary Table S4). Results showed that each was amplified using specific primers and teratocyte cDNA as template, whereas none were amplified using cDNA from non-parasitized *P. xylostella* larvae (Fig. 2). Sequencing of these PCR products further showed that each matched the corresponding expressed sequence tag (EST) in our transcriptome data set, which strongly supported that these transcripts derived from wasp and not host genes. Lastly, we cloned full-length cDNAs for select teratocyte transcripts in preparation for functional experiments presented below (see Supplementary Fig. S2).

### Gene expression in *C. vestalis* teratocytes varies temporally

**Figure 3:**
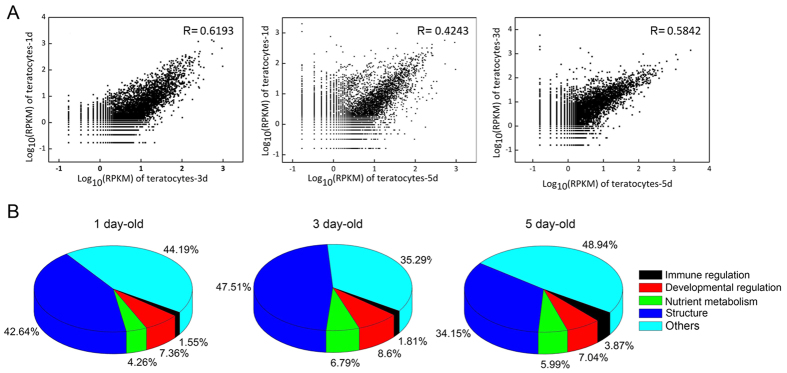
Digital gene expression data. **(A)** Pearson correlation coefficients for 1-day and 3-day old teratocytes, 1-day and 5-day old teratocytes, and 3-day and 5-day old teratocytes. **(B)** Percentage of teratocyte unigenes with RPKM values ≥ 1000 that were assigned to the categories of “structure”, “nutrient metabolism”, “developmental regulation” and “immune regulation” at 1, 3 and 5 days post-parasitism.

Digital gene expression (DGE) was used to profile expression patterns in teratocytes at different times post-parasitism. We sequenced a total of six DGE libraries generated from teratocytes that were collected from host larvae at 1, 3 and 5 days post-parasitism (two libraries per sample time) (see Supplementary Table S5). Saturation analysis showed that the percentage of identified transcripts plateaued when the number of sequenced tags was ≥4 million (see Supplementary Fig. S3). Reads Per Kb per Million reads (RPKM) was then used to fit Pearson correlation coefficients (R^2^) for each pair-wise comparison of DGE samples (Fig. 3A). The lowest correlation coefficient was between 1-day and 5-day old teratocytes, which suggested that gene expression in *C. vestalis* teratocytes changes in *P. xylostella* larvae during the course of parasitism.

Cross-referencing to the functional assignments made using the KEGG and IIID databases indicated that most teratocyte transcripts with RPKM values >1000 included factors assigned to the categories of “cellular structure”, “nutrient metabolism”, “developmental regulation” and “immune regulation” (Table 1). They also included several unknowns (Table 1). Transcripts in the category of “immune regulation” that had RPKM values ≥1000 and signal peptides were more abundant in 5 day old teratocytes than 1 day old teratocytes (Fig. 3B). In contrast, genes assigned to the categories of “nutrient metabolism”, “development regulation” and “cellular structure” exhibited variable abundance patterns between teratocytes collected at 1, 3 and 5 days post-parasitism (Fig. 3B).

**Figure 4:**
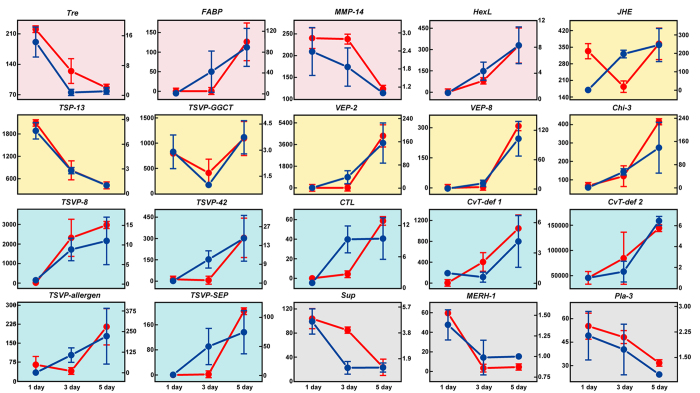
RPKM and qPCR abundance estimates of teratocyte transcripts. The same genes examined in Fig. 2 were analyzed at 1, 3 and 5 days post-parasitism. The left vertical axis shows RPKM values (Red) while the right vertical axis shows relative expression levels (Dark Blue) as measured by qPCR. The different colored backgrounds for each graph indicate the functional category a given gene was assigned to by KEGG or IIID analysis. Pink = nutrient metabolism; yellow = developmental regulation; blue = immune regulation; and grey = cellular structure. Error values for RPKM and qPCR data at each sample time indicate standard errors (SE).

For the 20 genes we analyzed by RT-PCR (see Fig. 2), we also compared their RPKM values to qPCR data generated from teratocytes collected at 1, 3 and 5 days post-parasitism. This showed that RPKM and qPCR data produced similar transcript abundance patterns (Fig. 4). As noted above, transcript abundance was higher in 5-day old teratocytes for genes in the functional category of immune regulation (Fig. 4). Genes assigned to cellular structure were mainly expressed in 1-day old teratocytes while genes in the categories of nutrient metabolism and developmental regulation showed variable expression patterns (Fig. 4).

### Two teratocyte proteins share similarities with venom components

**Figure 5:**
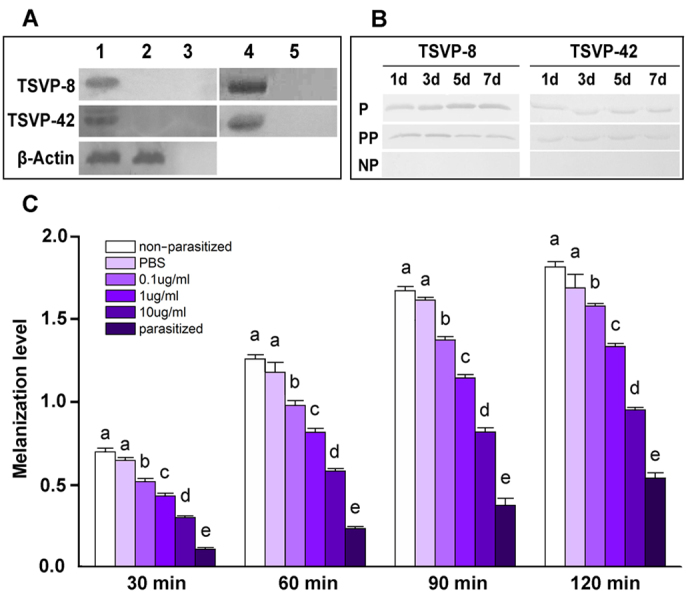
Detection and functional analysis of TSVP-8 and TSVP-42. **(A)** Immunoblot analysis of *C. vestalis* venom (lane 1), *C. vestalis* whole body without a venom apparatus (lane 2), PBS (lane 3), conditioned medium from a culture containing teratocytes (lane 4), and culture medium without teratocytes (lane 5). The upper blot was probed with a rTSVP-8 antibody, the middle blot was probed with a rTSVP-42 antibody, and the lower blot was probed with a ß-actin antibody. **(B)** Immunoblot analysis of cell-free hemolymph from parasitized *P. xylostella* (P), pseudo-parasitized *P. xylostella* (PP), and non-parasitized *P. xylostella* (NP). Samples for each treatment were collected at 1, 3, 5 and 7 days and probed with either the TSVP-8 (left blots) or TSVP-42 antibodies (right blots). **(C)** Melanization inhibition assay. Cell-free hemolymph was collected from non-parasitized *P. xylostella* larvae, parasitized *P. xylostella* larvae, larvae injected with PBS only, or larvae injected 3× with 0.1–10 μg/ml of rTSVP-8 protein in PBS. Each bar in the graph indicates melanization level ± SE from three independent experiments. Different letters above a given bar indicates significantly different means (p < 0.05).

We named two transcripts in our data set as teratocyte-secreted venom protein 8 (TSVP-8) and TSVP-42. We produced recombinant proteins for each in *Escherichia coli* and then used these products as antigens to generate polyclonal antibodies. Immunoblotting showed that these antibodies detected proteins corresponding to the predicted molecular mass of TSVP-8 and TSVP-42 in medium that we cultured *C. vestalis* teratocytes in for 24 h (Fig. 5A). They also detected proteins of similar molecular mass in venom collected from *C. vestalis* venom glands and reservoirs, but not in whole body extracts of *C. vestalis* without a venom apparatus (Fig. 5B). Together, these data indicated that teratocytes secrete TSVP-8 and TSVP-42, while also suggesting that antigenically similar or identical proteins are present in venom, which underlies our naming of these factors.

To assess the potential roles of TSVP-8 and TSVP-42 in parasitism, we gamma-irradiated *C. vestalis* females at a dose that does not affect female oviposition but renders all eggs inviable such that no parasitoid larva or teratocytes are produced (see Methods). We then compared the presence of TSVP-8 and TSVP-42 in the hemolymph of hosts stung by irradiated females (pseudo-parasitized) to hosts stung by normal females (parasitized). TSVP-8 and TSVP-42 were detected in both parasitized and pseudo-parasitized hosts, but abundance was qualitatively greater in the former (Fig. 5B). This suggested the presence of teratocytes increases the abundance of these proteins in parasitized hosts relative to pseudo-parasitized hosts, which contained venom but no teratocytes. BLAST analysis indicated that *C. vestalis* TSVP-8 was a homolog of a venom protein from *Cotesia rubecula* named Vn4.6 (see Supplementary Fig. S4). Vn4.6 was also previously reported to inhibit melanization of host hemolymph by interacting with the enzyme phenoloxidase^35^. To assess whether TSVP-8 reduced melanization, we added different amounts of recombinant TSVP-8 (rTSVP-8) to *P. xylostella* hemolymph and compared outcomes to hemolymph from non-parasitized and parasitized larvae. Results showed that rTSVP-8 dose-dependently reduced hemolymph melanization (Fig. 5C).

### Teratocyte defensins have anti-microbial activity and provide immune protection

**Figure 6:**
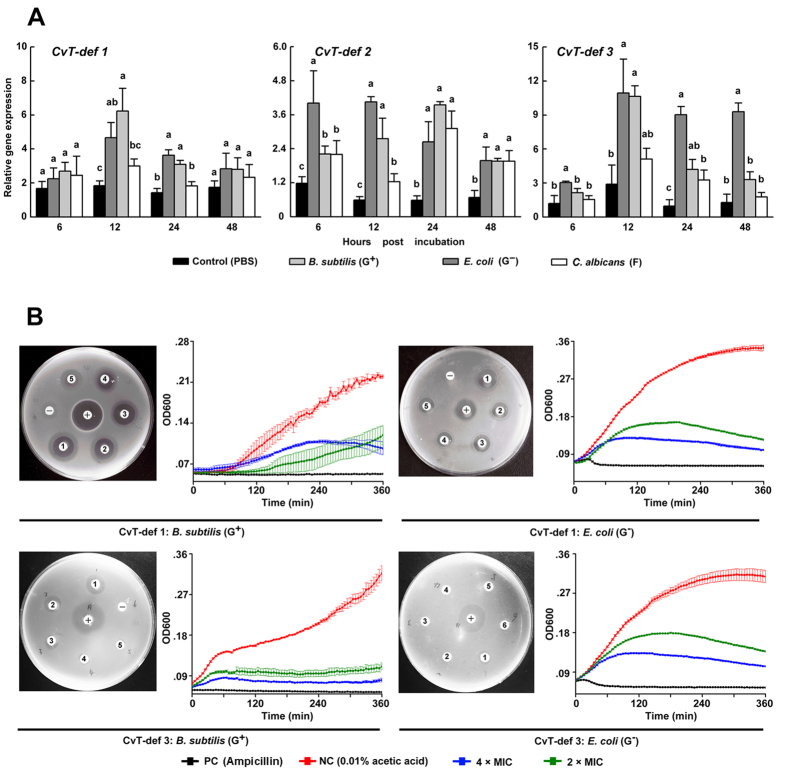
Expression and anti-microbial activity of CvT-def 1 and 3. (**A**) Expression levels of *CvT-def*s from 6–48 h post-challenge with *B. subtilis, E. coli, C. albicans,* or PBS (control) as measured by qPCR. Each time point and treatment was replicated 3 times with error bars indicating SE. For each time point, different letters indicate significantly different means (p < 0.05). (**B**) Outcomes of zone inhibition and kinetic growth assays using CvT-def 1 and 3 against *B. subtilis* and *E. coli*. For each treatment the left image shows a culture plate and inhibition zones which are identified 1–5, +, and −. Concentrations for CvT-def 1 are 4.55 μM (1), 2.28 μM (2) 1.14 μM (3), 0.57 μM (4), and 0.24 μM (5). Concentrations for CvT-def 3 are 8.77 μM (1), 4.39 μM (2), 2.19 μM (3), 1.1 μM (4), 0.55 μM (5). Ampicillin at 50 μg/ml is the positive control (+) while 0.01% acetic acid is the negative control (−). The right image shows graphs for the growth kinetic experiments using 2× and 4× concentrations of the MICs determined for CvT-def 1 and 3. Ampicillin at 50 μg/ml or 0.01% acetic acid served as controls. Growth kinetics were monitored at OD600 over 360 min with each treatment replicated 3 times. Error bars for each treatment and time point indicate SE.

We functionally examined three defensin genes, *CvT-def 1-3*, expressed in *C. vestalis* teratocytes that produce the predicted AMPs CvT-def 1-3 [GenBank KC306694, KC306695 and JX282323] (see Supplementary Fig. S2). As a first step, we asked whether expression of these genes was up-regulated if teratocytes in primary culture were exposed to three potential pathogens: the gram-positive bacterium *Bacillus subtiles,* the gram-negative bacterium *E. coli,* and spores of the fungus *Candida albicans.* qPCR assays detected increases in transcript abundance for all three genes between 6 and 48 h after exposure to *B. subtiles* and *E. coli* (Fig. 6A). In contrast, only *CvT-def 2* exhibited an increase in transcript abundance at 24 and 48 h after exposure to *C. albicans* (Fig. 6A).

We then synthesized CvT-def 1 and CvT-def 3 for use in functional assays. “Inhibition zone assays” showed that CvT-def 1 exhibited antimicrobial activity against *B. subtilis* and *E. coli*, while CvT-def 3 showed activity against only *B. subtilis* (Fig. 6B). We next determined that the Minimum Inhibitory Concentration (MIC) for CvT-def 1 was 0.03 μM for *B. subtilis* and 1.14 μM for *E. coli,* while the MIC for CvT-def 3 was 0.27 μM for *B. subtilis* and 35.1 μM for *E. coli*. When CvT-def 1 was present at 2× or 4× higher concentrations than the MIC, we observed a more rapid inhibition of *B. subtilis* than *E. coli* growth in kinetic assays (Fig. 6B). In contrast, the same assays using CvT-def 3 showed a similar rate of growth inhibition by *B. subtilis* and *E. coli* (Fig. 6B).

**Figure 7:**
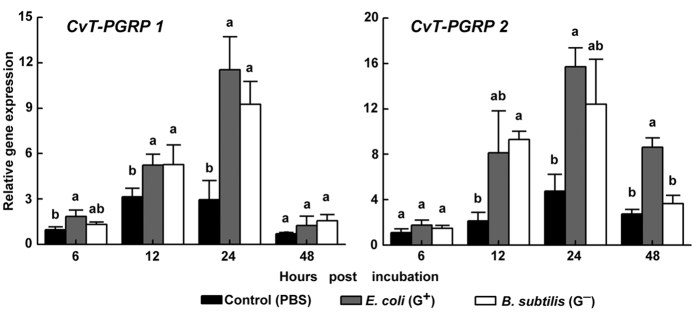
Expression of *CvT-PGRP1* and *CvT-PGRP2* in response to bacterial challenge. Expression levels from 6–48 h post-challenge with *B. subtilis, E. coli,* or PBS (control) as measured by qPCR. Each time point and treatment was replicated 3 times with error bars indicating SE. For each time point, different letters indicate significantly different means (p < 0.05).

Peptidoglycan recognition proteins (PGRPs) are known to play important roles in recognition of bacteria and activation of the pathways that regulate the expression of several AMPs including defensins. We assessed whether the addition of heat-killed *E. coli* or *B. subtilis* to primary teratocyte cultures increased expression of two PGRP genes, *CvT-PGRP 1* and *2* [GenBank KT322130 and KT309131] identified in our teratocyte transcriptome data set (see Supplementary Fig. S2). qPCR assays indicated that exposure of teratocytes to heat-killed *B. subtilis* or *E. coli* significantly elevated transcript abundance of *CvT-PGRP 1* and *2* relative to control teratocytes exposed to only phosphate buffered saline (PBS) (Fig. 7).

**Figure 8:**
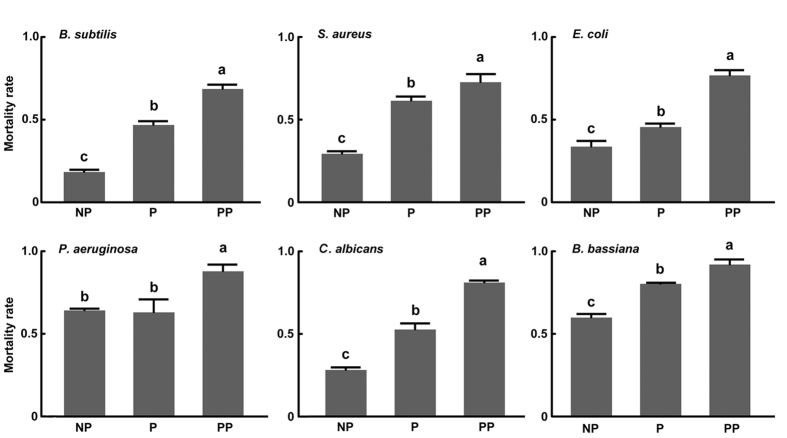
Mortality of non-parasitized (NP), parasitized (P) and pseudo-parasitized *P. xylostella* larvae after systemic infection with *E. coli*, *B. subtilis*, *S. aureus*, *P. aeruginosa*, *C. albicans* or *B. bassiana*. Each treatment and pathogen challenge was replicated 5 times with error bars indicating SE. For each pathogen, different letters above a given bar indicates significantly different means (p < 0.05).

Lastly, we compared the susceptibility of pseudo-parasitized hosts and parasitized hosts to systemic infection by *B. subtilis*, *E. coli*, and *C. albicans.* We also bioassayed two other bacteria, *Pseudomonas aeruginosa* and *Staphylococcus aureus,* and the fungus *Beauveria bassiana*. Results showed that mortality rates to each of the above organisms were higher in pseudo-parasitized hosts than parasitized and non-parasitized hosts (Fig. 8).

## Discussion

Endoparasitic braconids like *C. vestalis* parasitize hosts by injecting eggs, PDVs and venom, which is followed by the release of teratocytes when wasp eggs hatch^1^. Previous studies establish that PDVs and venoms have important functions in parasitism but the roles of teratocytes are less understood with only two transcriptome data sets reported and only a small number of studies that have investigated the function of specific teratocyte genes^16^,^18^,^28^,^33^,^34^. Here we provide a new transcriptome data set for *C. vestalis* teratocytes that were collected directly from hosts at different times post-parasitism. Our results also provide new information about teratocyte function by showing that TSVP-8 reduces the melanization of host hemolymph and two defensins exhibit anti-microbial activity.

Our identification of genes with predicted functions in cellular structure including the formation of microvilli was not surprising in light of studies documenting that teratocytes rapidly produce microvilli after release from eggs^1^,^36^. Our own observations also indicate that *C. vestalis* teratocytes have microvilli on their surface within 2 h of egg hatching and microvilli thereafter persist (see Supplementary Fig. S5). Consistent with a potential role in nutrition or altering host growth^18^,^20^,^21^,^22^,^24^, our results also identify numerous genes with predicted functions in metabolism of carbohydrates and lipids, and amino acid transport. Identification of transcripts for matrix metalloproteinases could be involved in digestion of fat body cells as reported in other studies^17^,^22^. Our detection of genes with functions in hormone production also supports earlier studies that implicate teratocytes in altering host growth, molting and metamorphosis^22^,^23^,^26^,^27^,^29^.

Our most intriguing results show that *C. vestalis* teratocytes express several immunity-related genes including pattern-recognition proteins, signal transduction factors, and effector molecules. DGE and qPCR data further indicate these transcripts are more abundant in the later stages of parasitism. This is potentially important for two reasons. First, the literature indicates that PDVs and/or venom disable several components of the host immune system including the pathways that regulate the production of AMPs^35^,^37^,^38^,^39^,^40^,^41^,^42^,^43^,^44^,^45^. This could increase the risk of infection of parasitized hosts by opportunistic pathogens. Thus, the production of AMPs by teratocytes could represent a compensatory adaptation by wasps that augments or replaces the production of anti-microbial defense molecules by hosts. This suggestion is supported by our finding that CvT-def 1 and 3 both exhibit anti-microbial activity as well as our data showing that parasitized hosts containing teratocytes exhibit lower mortality upon pathogen challenge than pseudo-parasitized hosts. Upregulated expression of *CvT-PGRPs* and the *CvT-defs* in response to bacterial or fungal challenge further suggests *C. vestalis* teratocytes have the ability to recognize pathogens, which results in upregulated expression of AMP genes.

Comparisons to the transcriptome data for teratocytes from *M. demolitor* and *C. plutellae* indicates that more than 50% of identified genes are homologs of genes that are also expressed in *C. vestalis* teratocytes (see Supplementary Fig. S6). However, profiles are more similar between the *M. demolitor* and *C. plutellae* data sets and the transcriptome data from 1-day old *C. vestalis* teratocytes than from 3-day and 5-day old *C. vestalis* teratocytes. Homologs for structural genes with potential functions in microvilli production were similarly detected in *M. demolitor, C. plutellae,* and day 1 *C. vestalis* teratocytes as were genes like *TSP-13*, *trehalase*, *maltase* and *hymenoptaecin*. In contrast, *TSVP-8* and *CvT-def 1* which are most abundant in day 5 teratocytes from *C. vestalis* were not identified in the *M. demolitor* and *C. plutellae* data sets. These differences may reflect that the data for *M. demolitor* and *C. plutellae* derive from teratocytes, which were relatively young and cultured *in vitro*. It could also reflect species differences in teratocyte function^46^.

## Methods

### Cultures and teratocyte collection

*P. xylostella* and *C. vestalis* cultures were established from pupae and parasitized host larvae that were collected from cabbage (*Brassica* spp.) fields in the suburbs of Hangzhou, Zhejiang Province, China. *P. xylostella* and *C. vestalis* colonies were thereafter reared on cabbage grown at 25 °C, 60–65% RH, and a photoperiod of 14 h Light: 10 h Dark. Adult wasps were fed a 20% honey-water solution. For experiments, third instar *P. xylostella* were parasitized by exposing larvae individually to a single mated female wasp until oviposition was observed.

Teratocytes were collected from hosts at 1, 3, and 5 days post-parasitism. Parasitized larvae were surface sterilized using 70% ethanol and then dissected in SFX-Insect cell culture medium (HyClone, USA) containing 20 μM of phenylthiourea. The dissected host was gently agitated in cell culture medium to release teratocytes and hemocytes from hemocoel. After 30 min, host hemocytes had attached to the bottom of the culture well while teratocytes remained non-adhesive. In this manner, teratocytes were separated from host hemocytes, and could be collected using a pipette and transferred to a micro-centrifuge tube with virtually no hemocyte contamination. Teratocytes were washed five times with SFX medium by gently centrifuging the cells at 500 × *g* for 5 min and decanting the supernatant. Teratocytes were then resuspended in fresh medium or PBS (pH = 7.0).

### Transcriptome and DGE sequencing

Total RNA was isolated from samples of teratocytes collected at 1, 3 and 5 days post-parasitism using the High Pure RNA Isolation Kit (Roche Diagnostics, Germany) followed by a second DNAase treatment (Qiagen, Germany). Each teratocyte sample was produced by dissecting 2000 parasitized host larvae, which were then pooled. *C. vestalis* usually lays one egg per host with each egg producing ~800 teratocytes. Thus, 2000 host larvae per sample time yields an estimated 1.6 × 10^6^ teratocytes for a total of 4.8 × 10^6^ teratocytes after pooling. RNA quality and concentration were assessed using a NanoDrop 2000 spectrometer (ThermoFisher Scientific, Waltham, MA), and by visualizing aliquots of samples after agarose electrophoresis and staining with ethidium bromide. A teratocyte cDNA library was then prepared by the Beijing Genome Institute (BGI, China) using a SMARTer^TM^ PCR cDNA Synthesis Kit (Clontech Laboratories, Mountain View, CA) and an Advantage 2 PCR Kit (Clontech Laboratories). For first-strand cDNA synthesis, a 3.5 μl aliquot of total RNA (about 80 ng) was mixed with 1 μl of 12 μM 3′ SMART CDs Primer II A. The mixture was incubated at 72 °C for 3 min and then 42 °C for 2 min in a hot-lid thermal cycler (Eppendorf, Germany). After adding 5.5 μl Master Mix (2 μl First-Strand Buffer, 0.25 μl 100 mM DTT, 1 μl 10 mM dNTP, 1 μl 12 mM SMARTer II A Oligonucleotide, 0.25 μl RNase inhibitor, and 1 μl SMARTScribe^TM^ reverse transcriptase), the reaction was incubated at 42 °C for 90 min and then terminated by heating at 70 °C for 10 min. First-strand cDNA was then used in PCR by combining a 2 μl aliquot with reaction reagents (10 μl 10×Advantage 2 PCR Buffer, 2 ml 10 μM 50×dNTP, 4 μl 12 μM 5′ PCR Primer II A, 2 μl 50×Advantage 2 Polymerase and 80 μl deionized water) followed by the following reaction conditions: 95 °C for 1 min and variable number of cycles of 95 °C for 15 s, 65 °C for 30 s and 68 °C for 6 min. After purification of amplified cDNAs using a QIAquick PCR Purification Kit (Qiagen), a paired-end sequencing library was produced using the Illumina RNA-seq library preparation kit. The library was then sequenced using the HiSeq 2000 platform (Illumina, San Diego, CA). Raw reads were quality filtered by SolexaQA^47^ with default parameters, and *de novo* assemblies were performed using Trinity (version 1.1.1)^48^ with default parameters. Assembled sequences were searched against the *C. vestalis* genome to remove contaminated sequences. Sequences were functionally annotated using BLAST against the NCBI Nr, GO, COG and KEGG databases.

Subsequently, teratocyte samples collected from hosts at 1, 3 and 5 days post-parasitism were used to produce 2 sequencing libraries per time point (6 libraries total) as described above. DGE was then used to determine the number of raw read fragments that mapped to genes in the teratocyte transcription dataset using Bowtie2 (Version 2.2.5)^49^ with default parameters. We used an FDR < 0.001 as the threshold to judge the significance of gene expression differences.

### RT-PCR, qPCR, and cDNA cloning

Teratocytes were collected from parasitized *P. xylostella* at 5 days post-parasitism and total RNA was extracted using the SV Total RNA Isolation System (Promega, USA). First-strand cDNA synthesis was performed using the ReverTra Ace qPCR RT Kit (TOYOBO, Japan). EST specific primers for 20 genes of interest were designed (see supplementary Table S6). The resulting DNA fragments were cloned into pGEM-T and bi-directionally Sanger sequenced to confirm their identity. *18S rRNA* of *C. vestalis* and *β-tubulin* of *P. xylostella* [GenBank No. EU127912] were used as positive and negative controls respectively in RT-PCR assays.

Total RNA extracted as above was used for cDNA cDNA library construction using the SMARTer^TM^ cDNA Library Construction Kit (Clontech, USA). Primers were then designed for use in 3′- and 5′-rapid amplification of cDNA ends (RACE) reactions (see supplementary Table S7). Amplified RACE products were purified using the AxyPrep DNA Gel Extraction Kit (Axygen, China), cloned into pGEM-T vector (Promega) and Sanger sequenced. Putatively complete open reading frames (ORFs) were then PCR amplified from the above teratocyte cDNA library using gene specific primers (see supplementary Table S8) followed by cloning into pGEM-T vector and sequencing.

qPCR reactions were performed using first strand cDNA templates produced as above from 1, 3 and 5 day old teratocytes and gene specific primers (supplementary Table S9). To normalize differences in total RNA among samples, *18S RNA* from *C. vestalis* was used as the endogenous control. All qPCR assays were performed using the ABI Prism 7500 fast real-time PCR system (Applied Biosystems, USA) in 20-μl reactions. Each 20-μl reaction contained 2-μl of template cDNA, 10-μl of Thunderbird Sybr qPCR Mix (TOYOBO, Japan) and 0.5 μM each of the corresponding forward and reverse primers. Three biological replicates were conducted, and data were analyzed using the 2^−ΔΔCT^ method^50^.

### TSVP antibodies and immunoblotting

The ORFs for TSVP-8 and TSVP-42 without their predicted signal peptides were amplified using specific primers containing restriction sites (see Supplementary Table S10) for directional cloning into pET-28a (His-tagged prokaryotic expression vector) (Novagen, USA). The resulting constructs were used to transform *E. coli* TG1 cells (TransGen Biotech, China). After sequencing of positive clones to ensure in-frame insertion, plasmids were used to transform *E. coli* BL21 (DE3) cells (TransGen Biotech, China). Transformed bacteria were then grown in Luria-Bertani (LB) medium containing kanamycin (50 ng/ml) (Sigma, USA) at 37 °C to an optical density of 0.5 at 600 nm followed by induction using isopropyl-b-D-thiogalactoside (Merck, Germany) at a final concentration of 1 mM. After 5 h, bacterial cells were harvested by centrifugation. Recombinant proteins were purified using the HisTALON™ Purification Kit (Clontech, USA) according to manufacturer’s instructions. Purified proteins were separated on 12% Sodium Dodecyl Sulfate Polyacrylamide Gel electrophoresis (SDS-PAGE) and visualized by immunoblotting using a mouse monoclonal anti-His antibody (Sigma, USA) and methods described below. rTSVP-8 and rTSVP-42 were then used as antigens to produce polyclonal antibodies in New Zealand white rabbits at the Beijing Proteomics Institute (BPI).

*C. vestalis* adult females were frozen at −20 °C for 10 minutes, and dissected under a stereo microscope (Leica MZ125, Germany) to collect venom gland reservoirs. Fifty pairs of venom reservoirs were opened using forceps in PBS (pH = 7.0) and centrifuged at 8,000 × *g* for 5 minutes. The supernatant was then collected and run on 15% SDS-PAGE gels. The remaining *C. vestalis* bodies without venom glands were extracted in SDS-PAGE buffer, centrifuged, and supernatants run on the above gel as a negative control. Teratocytes were also collected from 120 parasitized *P. xylostella* larvae at 6 days post-parasitism and incubated in 1 ml of SFX medium in a 24-well plate for 24 h. The resulting teratocyte conditioned medium was collected and concentrated to 50 μl using an alpha 1-2LD plus freeze drier (Christ, Germany). The proteins in the medium were then separated on 15% SDS-PAGE gels.

For pseudo-parasitization, *C. vestalis* adult females were exposed to a Cesium^137^ source at a dose of 120 Gy for 1 h^51^. These gamma-irradiated females were then used to pseudo-parasitize hosts while other hosts were parasitized by normal (non-irradiated) females. Hemolymph was collected from hosts at 1, 3, 5 and 7 days post-parasitism by piercing the cuticle of individual larvae using a sterile needle. Hemolymph collected from non-parasitized larvae serving as a control. After centrifugation at 12,000 × *g* for 5 min supernatant referred to as cell free hemolymph was isolated from each treatment and run on 15% SDS-PAGE gels.

All gels were transferred to polyvinylidene fluoride (Bio-Rad) membranes, and probed with the rTSVP-8 antibody (1:1000), the rTSVP-42 antibody (1:1000) or a commercial antibody to β-actin (1:2000) (HangZhou HuaAn Biotechnology Co., Ltd, China). Binding was visualized using a horseradish peroxididase (HRP)-conjugated goat anti-rabbit polyclonal secondary antibody (HangZhou HuaAn Biotechnology Co., Ltd, China) (1:2000) followed by use of the Pro-light HRP Chemiluminesent Detection Reagent (TianGen, China).

### Melanization assay

A 2 μl volume of rTSVP-8 at a concentration of 0.1, 1 or 10 μg/ml in PBS was injected 3 × into third instar *P. xylostella*, using a glass needle (World Precision Instruments Inc., USA) mounted onto a NN-153 type micromanipulator (Narishige, Japan). Larvae injected with PBS only or parasitized by *C. vestalis* served as a control. Hemolymph was then collected from larvae 12 h later as described above. Ten microliters of cell-free hemolymph were then added to 300 μl of PBS containing 20 mM DL-3, 4-dihydroxyphenylalanine in 96 well plate (Corning, USA) and incubated at room temperature in the dark followed by measurement of absorbance at 490 nm at 30, 60, 90 and 120 min using a Synergy plate reader (BioTek, USA). Melanization was then expressed as absorption units as is convention in the literature^35^.

### AMP and immune assays

The bacteria *B. subtilis* (ATCC6633), *E. coli* DH5α (ATCC69925), *S. aureus* (ATCC2592), and *P. aeruginosa* (ATCC9027), and fungi *C. albicans* (ATCC10231), and *B. bassiana* (ATCC7159) were obtained from The American Type Culture Collection (ATCC). *B. subtilis*, *S. aureus, P. aeruginosa* and *C. albicans* were cultured in Mueller-Hinton (MH) broth, DH5α *E. coli* were cultured in LB medium. *C. albicans* was cultured in Yeast Mold (YM) broth while *B. bassiana* was cultured in Martin Modified medium.

CvT-def 1 and CvT-def 3 peptides were synthesized by Sangon Biotech (Shanghai) Co., Ltd based on the predicted defensin domain (see Supplementary Table S11). The anti-microbial activity of these peptides was first measured *in vitro* using a zone inhibition assay. Briefly, each peptide was dissolved in 0.01% acetic acid. Overnight cultures of *B. subtilis, E. coli* and *C. albicans* were inoculated into fresh MH broth or LB medium and grown to stationary phase at 37 °C. Twenty μl of each culture was then diluted in 10 ml of pre-heated MH broth/LB medium containing 0.1% agar. The mixture was spread on a 9-cm Petri dish and then dried at room temperature. After settling, 6-mm filter paper disks were placed on the surface of the plate and then 2 μl of synthetic peptide at five different concentrations was added to each disk. The same volume of 0.01% acetic acid served as a negative control while 2 μl of ampicillin (Sigma, USA) (50 ng/ml) served as a positive control. Plates were incubated at 37 °C for 16 h and then qualitatively assessed for growth inhibition. Each treatment was replicated three times.

We then determined the MICs for CvT-def 1 and 3 against *B. subtilis* and *E. coli* by incubating fresh cultures with different concentrations of each peptide at for 24 h followed by measurement of growth at an optical density of 600 nm using a plate reader. Bacteria cultured with 50 ng/ml of ampicillin or 0.01% acetic acid served as controls. The MIC was expressed as an interval (a-b), where a was the highest peptide concentration tested that bacteria still grew and b was the lowest concentration that caused 100% growth inhibition^52^. Growth kinetics of *B. subtilis* and *E. coli* were also by serially diluting fresh cultures of each bacterium to an OD600 of 0.06 in 96 well culture plates followed by incubation in medium containing 2× or 4× the MIC for CvT-def 1 and 3 at 37 °C. Cultures containing ampicillin (50 ng/ml) or 0.01% acetic acid served as controls. The OD600 was then measured on a plate reader every 10 min with each treatment replicated three times.

*In vivo* anti-microbial assays consisted of inoculating pseudo-parasitized, parasitized or non-parasitized *P. xylostella* larvae with *E. coli*, *P. aeruginosa*, *B. subtilis, S. aureus,* or *C. albicans.* Larvae were inoculated 6 h after pseudo-parasitism or parasitism by pricking with a needle dipped in cultures of each grown to an OD600 of 0.8. Larvae were inoculated with inoculation with *B. bassiana* by placing them in a petri dish containing a sporulating *B. bassiana* culture and shaking for 30 sec. Larvae were then reared at 24 ^°^C (*E. coli*, *P. aeruginosa*, *B. subtilis*, *S. aureus* and *C. albicans*) or 29 ^°^C (*B. bassiana*). Mortality for each treatment was monitored until larvae died or pupated. Five biological replicates were conducted for each treatment with each replicate consisting of 50 larvae.

### Statistical analysis

Data generated for transcript abundances, anti-melanization activity of TSVPs, and *in vivo* mortality after inoculation with bacteria or fungi were analyzed by one-way analysis of variance (ANOVA) and post hoc Least Significant Difference (LSD) tests using SPSS software (SPSS 17.0, SPSS Inc., Chicago, IL).

## Additional Information

**Accession codes:** The RNA sequencing reads have been deposited in the short read archive under the accession SRR531389, and the individual data of digital gene expression also have been deposited in the short read archive under the accession SRR1915896, SRR1915897, SRR1915898, SRR1867933, SRR1867932 and SRR1867793.

**How to cite this article**: Gao, F. *et al.* *Cotesia vestalis* teratocytes express a diversity of genes and exhibit novel immune functions in parasitism. *Sci. Rep.* **6**, 26967; doi: 10.1038/srep26967 (2016).

## Supplementary Material

Supplementary Information

## Figures and Tables

**Table 1:** Expression patterns of genes with predicted signal peptides in 1, 3 and 5 day old teratocytes with RPKM values ≥1000.

Unigene ID	RPKM			Predicted function	Homolog
	1 day-old	3 day-old	5 day-old		
Unigene80	45668.64 ± 12507.66	84511.40 ± 51495.81	144127.01 ± 6763.96	IR	Defensin precursor
Unigene148	889.53 ± 590.43	5005.05 ± 376.59	11207.30 ± 170.27	IR	Hymenoptaecin
Unigene149	4839.70 ± 1592.85	8770.34 ± 1028.46	58408.59 ± 7778.68	None	Hypothetical protein
Unigene150	24532.78 ± 5104.14	6007.84 ± 86.87	20694.67 ± 1533.79	None	Hypothetical protein
Unigene151	5376.59 ± 833.58	14061.08 ± 5122.25	40104.39 ± 3965.24	None	Epididymal secretory protein E1
Unigene152	785.28 ± 140.29	1131.28 ± 169.12	2425.81 ± 35.36	DR	Serine protease inhibitor
Unigene165	8.54 ± 4.01	1.30 ± 0.60	1395.51 ± 303.98	IR	Hymenoptaecin
Unigene172	11339.35 ± 1237.77	15558.26 ± 5243.00	11350.60 ± 578.22	None	Epididymal secretory protein E1
Unigene174	7550.55 ± 129.48	5335.31 ± 193.60	4831.73 ± 38.11	None	Serine proteinase stubble-like
Unigene176	6679.48 ± 2307.97	1354.01 ± 150.61	3481.11 ± 80.67	None	Hypothetical protein
Unigene178	13207.59 ± 6523.08	7677.09 ± 1011.63	489.23 ± 126.87	None	Hypothetical protein
Unigene216	955.09 ± 25.87	163.03 ± 1.24	540.82 ± 27.21	None	Vascular endothelial growth factor receptor 1
Unigene262	669.39 ± 223.33	666.63 ± 90.96	1224.79 ± 153.56	DR	Teratocyte secreted protein
Unigene265	28.52 ± 21.99	101.18 ± 30.29	2912.45 ± 135.30	None	Hypothetical protein
Unigene278	4957.09 ± 148.96	807.93 ± 3.75	75.16 ± 20.80	None	Ribonuclease T2 domain-containing protein
Unigene284	4.15 ± 4.31	13.33 ± 9.58	3718.02 ± 72.85	IR	Defensin precursor
Unigene302	3007.96 ± 27.28	887.21 ± 235.46	335.43 ± 121.96	None	Hypothetical protein
Unigene342	807.71 ± 108.56	339.26 ± 125.01	2236.23 ± 303.47	None	Peptidyl-prolylcis-trans isomerase 5
Unigene350	745.96 ± 148.69	3014.69 ± 195.29	2977.59 ± 249.20	None	Paramyosin
Unigene502	0.00 ± 0.00	0.00 ± 0.00	1940.93 ± 467.36	None	Apolipoprotein
Unigene588	0.00 ± 0.00	0.00 ± 0.00	9789.24 ± 618.90	None	Chymotrypsin-1
Unigene421	0.48 ± 0.53	0.55 ± 0.20	6830.75 ± 356.12	NM	Maltase
Unigene971	30.47 ± 21.65	65.13 ± 16.48	1316.85 ± 415.54	IR	Defensin

IR: immune regulation; DR: developmental regulation; NM: nutrient metabolism.
