# Supplementary material for: Cotesia vestalis teratocytes express a diversity of genes and exhibit novel immune functions in parasitism
Source: Sci Rep. 2016 Jun 2;6:26967. doi: 10.1038/srep26967 (PMC4890588; doi:10.1038/srep26967)
Supplement: Supplementary Information [file srep26967-s1.pdf]

Supplementary information

Cotesia vestalis teratocytes express a diversity of genes and exhibit novel immune functions in parasitism

Fei Gao[1], Qi-juan Gu[1], Jing Pan[1], Ze-hua Wang[1], Chuan-lin Yin[2], Fei Li[1], Qi-sheng Song[3], Michael R. Strand[4], Xue-xin Chen[1], Min Shi[1,*]

[1] Ministry of Agriculture Key Lab of Agricultural Entomology and Institute of Insect Sciences, Zhejiang University, 866 Yuhangtang Road, Hangzhou 310058, China

[2] Department of Entomology, College of Plant Protection, Nanjing Agricultural University and Key Lab of Monitoring and Management of Plant Diseases and Insects, Ministry of Agriculture, 1 Weigang Road, Nanjing, Jiangsu 210095, China

[3] Molecular Insect Physiology, Division of Plant Sciences, University of Missouri, Columbia, Missouri 65211, USA.

[4] Department of Entomology, University of Georgia, Athens, Georgia 30602, USA

***Corresponding author**: Min Shi, State Key Laboratory of Rice Biology and Ministry of Agriculture Key Laboratory of Agricultural Entomology, Institute of Insect Sciences, Zhejiang University, 866 Yuhangtang Road, Hangzhou 310058, China, email: shimin0623@zju.edu.cn

Funding: this work was jointly by the 973 Program (2013CB127600), the National Science Fund for Innovative Research Groups (31321063)

Table S1 Summary of the transcriptome of *Cotesia vestalis* teratocyte

Nucleotides	1,254,124,980
Reads	6,967,361
Even length of reads	180bp
Contigs	233,765
Even length of contigs	124bp
Unigenes	11,924
Even length of Unigenes	491bp
Gap content	0.00%
GC content	40.81%

Table S2 Unigenes with identified signal peptides that are also homologs of known genes

Unigene ID	Homolog	Species of homolog
CL67.Contig1	Vacuolar proton pump subunit H	*Camponotus floridanus*
CL67.Contig2	V-type proton ATPase subunit H	*Acromyrmex echinatior*
CL77.Contig1	40S ribosomal protein S16	*Harpegnathos saltator*
CL77.Contig2	Ribosomal protein S16	*Lysiphlebus testaceipes*
CL100.Contig2	Hypothetical protein EAG_15273	*Camponotus floridanus*
CL154.Contig1	Venom protein Vn4.6	*Cotesia rubecula*
CL154.Contig2	Venom protein Vn4.6	*Cotesia rubecula*
CL156.Contig1	Conserved hypothetical protein	*Glyptapanteles flavicoxis*
CL162.Contig1	Aspartylglucosaminidase precursor	*Nasonia vitripennis*
CL179.Contig1	Hypothetical protein SINV_00409	*Solenopsis invicta*
CL179.Contig2	T-cell immunomodulatory protein precursor	*Apis mellifera*
CL194.Contig1	Hypothetical protein SINV_04265	*Solenopsis invicta*
CL202.Contig1	Vascular endothelial growth factor receptor 1	*Acromyrmex echinatior*
CL241.Contig1	Vascular endothelial growth factor receptor 1	*Acromyrmex echinatior*
CL241.Contig2	Hypothetical protein KGM_12338	*Danaus plexippus*
CL317.Contig1	Hypothetical protein LOC100118695	*Nasonia vitripennis*
CL317.Contig2	Hypothetical protein LOC100118695	*Nasonia vitripennis*
CL323.Contig1	Protein ADRM1	*Camponotus floridanus*
CL323.Contig2	Proteasomal ubiquitin receptor ADRM1-like protein	*Acromyrmex echinatior*
CL323.Contig3	Protein ADRM1-like protein	*Harpegnathos saltator*
CL328.Contig1	Hypothetical protein LOC100678084	*Nasonia vitripennis*
CL328.Contig2	Hypothetical protein LOC100678084	*Nasonia vitripennis*
CL334.Contig1	Calcium-transporting ATPase sarcoplasmic/endoplasmic reticulum type	*Acromyrmex echinatior*
CL335.Contig1	Conserved hypothetical protein	*Culex quinquefasciatus*
CL356.Contig1	CG2206-PB-like protein	*Daphnia pulex*
CL356.Contig2	Venom protein 2	*Microctonus hyperodae*
CL382.Contig1	Tolkin	*Apis mellifera*
CL396.Contig1	Hypothetical protein EAI_14262	*Harpegnathos saltator*
CL430.Contig2	Tenascin-X-like	*Nasonia vitripennis*
CL475.Contig1	Importin subunit beta-1-like isoform 2	*Nasonia vitripennis*
CL488.Contig1	Phenoloxidase inhibitor-like protein	*Simulium nigrimanum*
CL492.Contig1	Hypothetical protein SINV_01823	*Solenopsis invicta*
CL494.Contig1	Slit homolog 1 protein-like	*Bombus terrestris*
CL495.Contig1	Hypothetical protein LOC100162921	*Acyrthosiphon pisum*
CL495.Contig3	Venom protein 2	*Microctonus hyperodae*
CL531.Contig1	Ribonuclease Oy-like	*Nasonia vitripennis*
CL568.Contig1	--	
CL570.Contig2	Hypothetical protein SINV_04265	*Solenopsis invicta*
CL578.Contig1	Hypothetical protein EAI_17152	*Harpegnathos saltator*
CL590.Contig1	Serine/threonine-protein kinase/endoribonuclease IRE1-like	*Nasonia vitripennis*
CL611.Contig2	Venom protein Vem17	*Chelonus inanitus*
CL616.Contig1	GA21974	*Drosophila pseudoobscura pseudoobscura*
CL649.Contig1	Hypothetical protein SINV_04265	*Solenopsis invicta*
CL668.Contig1	Endoplasmin-like	*Megachile rotundata*
CL668.Contig2	Endoplasmin	*Camponotus floridanus*
CL674.Contig1	Ribonuclease T2 domain	*Glyptapanteles indiensis*
CL692.Contig1	Aminopeptidase N	*Camponotus floridanus*
Unigene2	GF25073	*Drosophila ananassae*
Unigene43	Peptidyl-prolyl cis-trans isomerase FKBP14-like	*Bombus impatiens*
Unigene80	Defensin 2 precursor	*Cotesia vestalis*
Unigene95	Vascular endothelial growth factor receptor 2	*Harpegnathos saltator*
Unigene96	Vascular endothelial growth factor receptor 2	*Harpegnathos saltator*

Unigene124	Sparc	*Danaus plexippus*
Unigene148	Hymenoptaecin	*Nasonia vitripennis*
Unigene149	--	
Unigene150	--	
Unigene151	Epididymal secretory protein E1	*Camponotus floridanus*
Unigene152	Serine protease inhibitor 3/4	*Nasonia vitripennis*
Unigene159	Ribosomal protein S26	*Rhodnius prolixus*
Unigene164	Hymenoptaecin	*Bombus perezi*
Unigene165	Hymenoptaecin	*Bombus perezi*
Unigene172	Protein NPC2 homolog	*Nasonia vitripennis*
Unigene174	Serine proteinase stubble-like	*Apis florea*
Unigene176	Hypothetical protein DAPPUDRAFT_311900	*Daphnia pulex*
Unigene178	GA27145	*Drosophila pseudoobscura pseudoobscura*
Unigene216	Vascular endothelial growth factor receptor 1	*Camponotus floridanus*
Unigene239	Hexamerin-like	*Megachile rotundata*
Unigene244	Calreticulin	*Cotesia rubecula*
Unigene262	Teratocyte secreted protein	*Microplitis croceipes*
Unigene278	Ribonuclease T2 domain-containing protein	*Glyptapanteles flavicoxis*
Unigene280	Heat shock 70 kDa protein cognate 3-like	*Nasonia vitripennis*
Unigene284	Defensin 2 precursor	*Cotesia vestalis*
Unigene288	Ribonuclease T2 domain-containing protein	*Glyptapanteles flavicoxis*
Unigene311	Hypothetical protein AND_07138	*Anopheles darlingi*
Unigene312	Conserved hypothetical protein	*Culex quinquefasciatus*
Unigene321	Protein disulfide-isomerase A6-like	*Bombus terrestris*
Unigene326	Cathepsin L-like	*Apis florea*
Unigene333	Neuroserpin-like	*Megachile rotundata*
Unigene336	Venom protein 2	*Microctonus hyperodae*
Unigene338	Venom protein 2	*Microctonus hyperodae*
Unigene340	Vitellogenin	*Harpegnathos saltator*
Unigene342	peptidylprolyl isomerase B precursor	*Bombyx mori*
Unigene348	Venom protein 2	*Microctonus hyperodae*
Unigene349	Vascular endothelial growth factor receptor 2	*Harpegnathos saltator*
Unigene350	Paramyosin, long form-like	*Nasonia vitripennis*
Unigene351	Venom protein 2	*Microctonus hyperodae*
Unigene357	hypothetical protein EAI_01992	*Harpegnathos saltator*
Unigene359	Protein disulfide-isomerase A3-like	*Megachile rotundata*
Unigene371	Venom protein 2	*Microctonus hyperodae*
Unigene378	Vitellogenin	*Harpegnathos saltator*
Unigene384	Ecdysteroid regulated 16 kDa	*Tribolium castaneum*
Unigene389	Dolichyl-diphosphooligosaccharide--protein glycosyltransferase subunit 2-like	*Nasonia vitripennis*
Unigene396	hypothetical protein G5I_04912	*Acromyrmex echinatior*
Unigene397	Signal sequence receptor, alpha precursor	*Nasonia vitripennis*
Unigene415	Dolichyl-diphosphooligosaccharide--protein glycosyltransferase 48 kDa subunit-like	*Megachile rotundata*
Unigene421	Maltase	*Cerapachys biroi*
Unigene422	Cystatin	*Glyptapanteles flavicoxis*
Unigene424	Serine protease inhibitor 3/4	*Nasonia vitripennis*
Unigene432	Protein disulfide-isomerase	*Acromyrmex echinatior*
Unigene434	Mesencephalic astrocyte-derived neurotrophic factor homolog	*Nasonia vitripennis*
Unigene473	Transmembrane emp24 domain-containing protein	*Acromyrmex echinatior*
Unigene489	Metalloproteinase inhibitor 3	*Harpegnathos saltator*
Unigene491	Translocon-associated protein subunit beta	*Acromyrmex echinatior*
Unigene494	Chitinase 3	*Drosophila melanogaster*
Unigene495	Chymotrypsin-1	*Nasonia vitripennis*
Unigene498	Hypothetical protein SINV_10017	*Solenopsis invicta*

Unigene502	Apolipoprotein D-like	*Megachile rotundata*
Unigene510	Chitin synthase-like protein	*Glyptapanteles indiensis*
Unigene511	Niemann-Pick C1 protein	*Acromyrmex echinatior*
Unigene516	Neuroserpin-like	*Megachile rotundata*
Unigene522	AGAP004674-PA	*Anopheles gambiae str.*
Unigene535	Translocon-associated protein subunit delta-like	*Nasonia vitripennis*
Unigene569	Neuroserpin-like	*Megachile rotundata*
Unigene588	Chymotrypsin-1	*Nasonia vitripennis*
Unigene602	Chymotrypsin-1	*Nasonia vitripennis*
Unigene629	Hypothetical protein SINV_05458	*Solenopsis invicta*
Unigene636	Astakine-like	*Nasonia vitripennis*
Unigene646	Protein ERGIC-53-like	*Megachile rotundata*
Unigene653	G-protein coupled receptor Mth2	*Acromyrmex echinatior*
Unigene662	Hypothetical protein LOC725426 isoform 2	*Apis mellifera*
Unigene665	Esterase FE4	*Nasonia vitripennis*
Unigene671	Dolichyl-diphosphooligosaccharide--protein glycosyltransferase subunit 1-like	*Bombus impatiens*
Unigene678	Counting factor associated protein D-like	*Bombus terrestris*
Unigene688	Hypothetical protein LOC100119712	*Nasonia vitripennis*
Unigene697	Protein kish-A-like	*Bombus impatiens*
Unigene699	Ribonuclease Oy-like	*Megachile rotundata*
Unigene721	Endoplasmic reticulum resident protein 44	*Apis mellifera*
Unigene723	Hypothetical protein KGM_18288	*Danaus plexippus*
Unigene739	Venom protein Vn50	*Cotesia rubecula*
Unigene753	Tolloid-like protein 2-like	*Nasonia vitripennis*
Unigene758	Protein toll	*Harpegnathos saltator*
Unigene771	GJ24134	*Drosophila virilis*
Unigene772	Protein canopy-1-like	*Bombus terrestris*
Unigene785	Protein yellow	*Camponotus floridanus*
Unigene797	Hypothetical protein SINV_15287	*Solenopsis invicta*
Unigene833	Dolichol-phosphate mannosyltransferase subunit 3-like	*Megachile rotundata*
Unigene842	Peroxiredoxin-4-like	*Nasonia vitripennis*
Unigene847	A disintegrin and metalloproteinase with thrombospondin motifs 1	*Acromyrmex echinatior*
Unigene849	Lysosomal alpha-mannosidase-like isoform 1	*Nasonia vitripennis*
Unigene859	Hypothetical protein LOC100123845	*Nasonia vitripennis*
Unigene886	Adenosine deaminase CECR1-like	*Nasonia vitripennis*
Unigene899	Protein toll	*Acromyrmex echinatior*
Unigene913	Hypothetical protein TcasGA2_TC010724	*Tribolium castaneum*
Unigene929	Chymotrypsin-1	*Nasonia vitripennis*
Unigene954	Neutral alpha-glucosidase AB-like	*Megachile rotundata*
Unigene955	Hypothetical protein LOC100121961	*Nasonia vitripennis*
Unigene971	Defensinis	*Apis cerana japonica*
Unigene979	15 kDa selenoprotein-like	*Nasonia vitripennis*
Unigene1003	Vesicle-trafficking protein SEC22b-B-like	*Megachile rotundata*
Unigene1011	Membrane metallo-endopeptidase-like 1-like	*Bombus impatiens*
Unigene1035	CD63 antigen isoform 1	*Apis mellifera*
Unigene1046	AGAP004674-PA	*Anopheles gambiae str.*
Unigene1055	C-type lectin	*Acyrthosiphon pisum*
Unigene1080	Hypoxia up-regulated protein 1-like isoform 2	*Nasonia vitripennis*
Unigene1085	Hypothetical protein	*Cotesia congregata*
Unigene1094	Venom acid phosphatase Acph-1-like	*Apis mellifera*
Unigene1118	Thioredoxin-related transmembrane protein 1-like	*Nasonia vitripennis*
Unigene1127	Nucleobindin-2	*Nasonia vitripennis*
Unigene1138	Venom protein Ci-48a	*Chelonus inanitus*
Unigene1148	UDP-glucose:glycoprotein glucosyltransferase-like	*Nasonia vitripennis*

Unigene1223	Immediate early response 3-interacting protein 1-like	*Bombus impatiens*
Unigene1242	Retinoid-inducible serine carboxypeptidase-like	*Apis florea*
Unigene1249	Vesicular integral-membrane protein VIP36-like	*Nasonia vitripennis*
Unigene1281	SelT-like protein-like	*Nasonia vitripennis*
Unigene1289	cytochrome oxidase subunit I	*Cotesia chilonis*
Unigene1314	--	
Unigene1321	Glutaminyl-peptide cyclotransferase-like	*Bombus impatiens*
Unigene1401	C-type lectin	*Acyrthosiphon pisum*
Unigene1407	Small glutamine-rich tetratricopeptide repeat-containing protein alpha	*Apis mellifera*
Unigene1409	Protein disulfide-isomerase-like	*Nasonia vitripennis*
Unigene1422	Uncharacterized protein LOC100882893	*Megachile rotundata*
Unigene1525	AGAP006426-PA	*Anopheles gambiae str.*
Unigene1537	Aminopeptidase N	*Camponotus floridanus*
Unigene1550	Uncharacterized protein LOC100867171	*Apis florea*
Unigene1556	Bone morphogenetic protein	*Aedes aegypti*
Unigene1590	DnaJ homolog subfamily C member 3-like	*Megachile rotundata*
Unigene1628	Basigin-like	*Bombus terrestris*
Unigene1649	Tenascin-X-like	*Nasonia vitripennis*
Unigene1674	Hypothetical protein SINV_04265	*Solenopsis invicta*
Unigene1679	UPF0139 membrane protein pMsmaA27-like	*Nasonia vitripennis*
Unigene1694	Ero1-like protein-like	*Megachile rotundata*
Unigene1695	A disintegrin and metalloproteinase with thrombospondin motifs 1	*Acromyrmex echinatior*
Unigene1728	Glucosidase 2 subunit beta	*Camponotus floridanus*
Unigene1730	Peptidylprolyl isomerase B precursor	*Bombyx mori*
Unigene1748	Lysosomal aspartic protease-like	*Nasonia vitripennis*
Unigene1775	Conserved hypothetical protein	*Glyptapanteles indiensis*
Unigene1814	Protein GPR107-like	*Megachile rotundata*
Unigene1820	DnaJ homolog subfamily B member 11-like	*Apis mellifera*
Unigene1843	Tolloid-like protein 2-like	*Acyrthosiphon pisum*
Unigene1846	Transmembrane 9 superfamily member 2-like	*Nasonia vitripennis*
Unigene1849	Asparagine-rich cocoon silk protein	*Cotesia glomerata*
Unigene1883	Hypothetical protein AaeL_AAEL012205	*Aedes aegypti*
Unigene1899	Hypothetical protein GIP_L1_00310	*Glyptapanteles indiensis*
Unigene1926	Hypoxia up-regulated protein 1-like	*Bombus terrestris*
Unigene1981	Apolipoprotein D-like	*Megachile rotundata*
Unigene1991	Protein disulfide-isomerase	*Harpegnathos saltator*
Unigene1994	Hypothetical protein	*Locusta migratoria*
Unigene2015	Venom serine protease	*Bombus ignitus*
Unigene2082	A disintegrin and metalloproteinase with thrombospondin motifs 1	*Acromyrmex echinatior*
Unigene2090	Uncharacterized protein LOC100883027	*Megachile rotundata*
Unigene2113	Protein CREG1-like	*Nasonia vitripennis*
Unigene2141	Regucalcin-like	*Apis mellifera*
Unigene2169	Sodium/hydrogen exchanger 7	*Apis mellifera*
Unigene2202	Uncharacterized protein LOC100865627	*Apis florea*
Unigene2219	Endoplasmic reticulum resident protein 29-like	*Apis florea*
Unigene2227	Protein canopy homolog 3-like	*Megachile rotundata*
Unigene2236	Esterase FE4	*Nasonia vitripennis*
Unigene2241	Hypothetical protein SINV_07362	*Solenopsis invicta*
Unigene2243	UDP-glucose:glycoprotein glucosyltransferase-like	*Megachile rotundata*
Unigene2277	Transmembrane 9 superfamily member 4-like	*Bombus impatiens*
Unigene2294	Hypothetical protein SINV_08281	*Solenopsis invicta*
Unigene2307	Transmembrane emp24 domain-containing protein 5	*Acromyrmex echinatior*
Unigene2309	Extracellular sulfatase SULF-1 homolog	*Megachile rotundata*
Unigene2328	Extracellular sulfatase SULF-1 homolog	*Bombus impatiens*

Unigene2350	Transmembrane 9 superfamily member 2-like isoform 1	*Apis mellifera*
Unigene2406	Hypothetical protein SINV_04535	*Solenopsis invicta*
Unigene2426	Transmembrane protein 129-like	*Nasonia vitripennis*
Unigene2450	Transmembrane 9 superfamily member 4	*Acromyrmex echinatior*
Unigene2473	Tolloid like	*Achaearanea tepidariorum*
Unigene2516	Putative phospholipase B-like lamina ancestor-like isoform 1	*Nasonia vitripennis*
Unigene2547	General vesicular transport factor p115	*Acromyrmex echinatior*
Unigene2582	Uncharacterized protein LOC100880824	*Megachile rotundata*
Unigene2637	Hypothetical protein SINV_06137	*Solenopsis invicta*
Unigene2641	Renin receptor-like isoform 2	*Nasonia vitripennis*
Unigene2700	Hypothetical protein GIP_L1_00660	*Glyptapanteles indiensis*
Unigene2704	Tolloid-like protein 2	*Acromyrmex echinatior*
Unigene2713	Uncharacterized protein LOC100880959	*Megachile rotundata*
Unigene2729	Angiotensin-converting enzyme	*Camponotus floridanus*
Unigene2802	Serine/threonine-protein kinase/endoribonuclease IRE1-like	*Megachile rotundata*
Unigene2804	Retinoid-inducible serine carboxypeptidase-like	*Apis florea*
Unigene2843	Dolichyl pyrophosphate Man9GlcNAc2 alpha-1,3-glucosyltransferase-like	*Nasonia vitripennis*
Unigene2864	Transmembrane 9 superfamily member 4-like isoform 1	*Apis mellifera*
Unigene2868	Hypothetical protein LOC100744939 isoform 2	*Bombus impatiens*
Unigene2902	Hypothetical protein SINV_02630	*Solenopsis invicta*
Unigene2920	Hypothetical protein EAG_11674	*Camponotus floridanus*
Unigene2921	Mitochondrial 2-oxoglutarate/malate carrier protein-like	*Nasonia vitripennis*
Unigene2967	Ubiquitin-conjugating enzyme E2 R2	*Harpegnathos saltator*
Unigene2994	Probable pyruvate dehydrogenase E1 component subunit alpha, mitochondrial	*Camponotus floridanus*
Unigene3032	Putative serine protease K12H4.7-like	*Apis florea*
Unigene3045	Putative esterase CG3488	*Camponotus floridanus*
Unigene3068	Hypothetical protein SINV_01752	*Solenopsis invicta*
Unigene3172	Calumenin-like	*Nasonia vitripennis*
Unigene3194	Large proline-rich protein BAG6-like	*Megachile rotundata*
Unigene3200	Sodium/hydrogen exchanger 7	*Acromyrmex echinatior*
Unigene3237	Juvenile hormone esterase	*Camponotus floridanus*
Unigene3239	Zinc transporter foi-like	*Bombus impatiens*
Unigene3277	Selenoprotein M-like	*Nasonia vitripennis*
Unigene3305	Hypothetical protein TcasGA2_TC002380	*Tribolium castaneum*
Unigene3342	Hypothetical protein LOC100744939 isoform 2	*Bombus impatiens*
Unigene3386	Calnexin	*Camponotus floridanus*
Unigene3470	hypothetical protein	*Cotesia congregata*
Unigene3500	Serine/threonine-protein kinase/endoribonuclease IRE1-like	*Nasonia vitripennis*
Unigene3582	Torsin-like protein	*Camponotus floridanus*
Unigene3601	Ecdysone-inducible gene E1	*Drosophila melanogaster*
Unigene3624	Zinc transporter ZIP9-like	*Bombus impatiens*
Unigene3662	UDP-glucose:glycoprotein glucosyltransferase-like	*Nasonia vitripennis*
Unigene3791	UPF0480 protein C15orf24 homolog	*Apis mellifera*
Unigene3804	hypothetical protein GIP_L1_00320	*Glyptapanteles indiensis*
Unigene3857	Hypothetical protein SINV_04265	*Solenopsis invicta*
Unigene3933	Venom protein 2	*Microctonus hyperodae*
Unigene3954	LDLR chaperone boca-like	*Nasonia vitripennis*
Unigene3956	DnaJ homolog subfamily C member 1-like	*Nasonia vitripennis*
Unigene3985	hypothetical protein GIP_L2_0100	*Glyptapanteles indiensis*
Unigene4089	Protein YIPF1	*Camponotus floridanus*
Unigene4238	Hypothetical protein SINV_02630	*Solenopsis invicta*
Unigene4274	Chitinase domain-containing protein 1-like	*Bombus terrestris*
Unigene4295	Neuferricin-like	*Megachile rotundata*
Unigene4301	Hypothetical protein LOC100746784	*Bombus impatiens*

Unigene4326	Exportin-7	*Acromyrmex echinatior*
Unigene4375	GD20531	*Drosophila simulans*
Unigene4426	GJ10807	*Drosophila virilis*
Unigene4537	Hypoxia up-regulated protein 1	*Acromyrmex echinatior*
Unigene4608	Serine/threonine-protein kinase/endoribonuclease ire-1	*Camponotus floridanus*
Unigene4657	Adenosine deaminase CECR1-like	*Nasonia vitripennis*
Unigene4681	Vascular endothelial growth factor receptor 1	*Acromyrmex echinatior*
Unigene4714	Sodium/hydrogen exchanger 8	*Acromyrmex echinatior*
Unigene4735	Hypothetical protein EAG_14954	*Camponotus floridanus*
Unigene4790	Sialin-like	*Bombus impatiens*
Unigene4869	UDP-glucuronosyltransferase 2C1	*Acromyrmex echinatior*
Unigene4972	Nuclear pore complex protein Nup93-like	*Nasonia vitripennis*
Unigene4983	Uncharacterized protein LOC100879467	*Megachile rotundata*
Unigene4998	Putative alpha-1,2-glucosyltransferase ALG10-B	*Harpegnathos saltator*
Unigene5028	Hypothetical protein SINV_11023	*Solenopsis invicta*
Unigene5038	Iron/zinc purple acid phosphatase-like protein	*Acromyrmex echinatior*
Unigene5094	Transport and Golgi organization protein 1	*Acromyrmex echinatior*
Unigene5153	Cytosolic endo-beta-N-acetylglucosaminidase-like	*Megachile rotundata*
Unigene5159	Vacuolar fusion protein MON1 homolog A-like	*Bombus terrestris*
Unigene5184	Ileal sodium/bile acid cotransporter-like isoform 1	*Nasonia vitripennis*
Unigene5189	Chitooligosaccharidolytic beta-N-acetylglucosaminidase-like	*Nasonia vitripennis*
Unigene5238	Hypothetical protein AaeL_AAEL014309	*Aedes aegypti*
Unigene5322	Retinol dehydrogenase 11-like	*Nasonia vitripennis*
Unigene5326	Esterase FE4-like	*Apis mellifera*
Unigene5395	Constitutive coactivator of PPAR-gamma-like protein 1 homolog	*Megachile rotundata*
Unigene5463	Chitooligosaccharidolytic beta-N-acetylglucosaminidase-like	*Nasonia vitripennis*
Unigene5464	Protein sel-1 homolog 1-like	*Bombus impatiens*
Unigene5497	Venom protein 2	*Microctonus hyperodae*
Unigene5502	CTL-like protein 1-like	*Megachile rotundata*
Unigene5562	Hypothetical protein SINV_07042	*Solenopsis invicta*
Unigene5589	Extracellular Cu/Zn superoxide dismutase	*Lasius niger*
Unigene5597	Large proline-rich protein BAG6-like	*Bombus impatiens*
Unigene5615	Hypothetical protein SINV_04568	*Solenopsis invicta*
Unigene5678	Protein sel-1 homolog 1-like	*Bombus impatiens*
Unigene5757	Uncharacterized protein KIAA0195-like	*Megachile rotundata*
Unigene5772	Conserved hypothetical protein	*Glyptapanteles flavicoxis*
Unigene5786	Hypothetical protein DAPPUDRAFT_229736	*Daphnia pulex*
Unigene5795	Protein spinster-like	*Megachile rotundata*
Unigene5807	Mediator of RNA polymerase II transcription subunit 16-like	*Apis florea*
Unigene5809	Type-1 angiotensin II receptor-associated protein-like	*Apis mellifera*
Unigene5811	Hypothetical protein LOC100647501	*Bombus terrestris*
Unigene5849	Protein disulfide-isomerase like protein ERp57	*Bombyx mori*
Unigene5898	Alpha-2-macroglobulin receptor-associated protein-like	*Apis mellifera*
Unigene5926	Cysteine-rich with EGF-like domain protein 2	*Acromyrmex echinatior*
Unigene6014	Hypothetical protein DAPPUDRAFT_231233	*Daphnia pulex*
Unigene6037	Serine/threonine-protein kinase/endoribonuclease IRE1-like	*Nasonia vitripennis*
Unigene6049	Hypothetical protein SINV_07475	*Solenopsis invicta*
Unigene6106	Ribonuclease Oy	
Unigene6145	Hypothetical protein AaeL_AAEL002560	*Aedes aegypti*
Unigene6149	Abhydrolase domain-containing protein 13-like	*Apis florea*
Unigene6178	Venom protein Vem17	*Chelonus inanitus*
Unigene6244	Uncharacterized protein LOC100881454	*Megachile rotundata*
Unigene6298	Hypothetical protein LOC412103	*Apis mellifera*
Unigene6299	Chitooligosaccharidolytic beta-N-acetylglucosaminidase	*Harpegnathos saltator*
Unigene6355	Sodium/hydrogen exchanger 8	*Camponotus floridanus*

Unigene6444	Conserved hypothetical protein	*Pediculus humanus corporis*
Unigene6457	Cuticular protein RR-2 family member 59 precursor	*Nasonia vitripennis*
Unigene6534	Sodium-dependent phosphate transport protein 1, chloroplastic-like	*Megachile rotundata*
Unigene6542	Hypothetical protein SINV_00047	*Solenopsis invicta*
Unigene6545	E3 ubiquitin-protein ligase UBR5	*Harpegnathos saltator*
Unigene6617	Zinc finger protein 84	*Acromyrmex echinatior*
Unigene6626	Protein FAM114A2-like	*Bombus terrestris*
Unigene6707	Protein sel-1 homolog 1-like	*Apis florea*
Unigene6715	Hypothetical protein LOC100651997	*Bombus terrestris*
Unigene6740	Plasma membrane calcium-transporting ATPase 3-like	*Nasonia vitripennis*
Unigene6743	Hypothetical protein SINV_02878	*Solenopsis invicta*
Unigene6782	Calnexin-like	*Megachile rotundata*
Unigene6793	Cysteine-rich with EGF-like domain protein 1-like	*Apis florea*
Unigene6806	Peroxisomal membrane protein PEX13-like	*Nasonia vitripennis*
Unigene6809	SID1 transmembrane family member 2-like	*Nasonia vitripennis*
Unigene6905	Hypothetical protein SINV_07362	*Solenopsis invicta*
Unigene6917	Lactosylceramide 4-alpha-galactosyltransferase	*Harpegnathos saltator*
Unigene6935	Zinc transporter foi-like	*Bombus impatiens*
Unigene7025	Protein sel-1 homolog 1-like	*Nasonia vitripennis*
Unigene7116	Large proline-rich protein BAG6-like	*Megachile rotundata*
Unigene7127	Metal transporter CNNM2-like	*Nasonia vitripennis*
Unigene7133	Interferon-related developmental regulator 1	*Camponotus floridanus*
Unigene7151	Carboxypeptidase D	*Acromyrmex echinatior*
Unigene7291	Transmembrane protein 59-like	*Apis florea*
Unigene7313	Alpha-2-macroglobulin receptor-associated protein-like	*Nasonia vitripennis*
Unigene7325	E3 ubiquitin-protein ligase UBR2-like	*Megachile rotundata*
Unigene7327	Peptidyl-alpha-hydroxyglycine alpha-amidating lyase 1-like	*Megachile rotundata*
Unigene7378	Hypothetical protein GIP_L1_00280	*Glyptapanteles indiensis*
Unigene7422	Sterol regulatory element-binding protein 1-like	*Nasonia vitripennis*
Unigene7534	Protein spitz-like	*Megachile rotundata*
Unigene7569	Hypothetical protein SINV_04474	*Solenopsis invicta*
Unigene7689	Chitooligosaccharidolytic beta-N-acetylglucosaminidase-like	*Megachile rotundata*
Unigene7699	Torsin-like protein-like	*Megachile rotundata*
Unigene7763	Juvenile hormone esterase binding protein	*Nasonia vitripennis*
Unigene7812	Transmembrane 9 superfamily member 3	*Megachile rotundata*
Unigene7824	Heat shock protein 70-3	*Bombyx mori*
Unigene7877	Leucine-rich repeat and immunoglobulin-like domain-containing nogo receptor-interacting protein 2	*Camponotus floridanus*
Unigene7888	Metal transporter CNNM2-like	*Apis florea*
Unigene7912	Peptidyl-alpha-hydroxyglycine alpha-amidating lyase 1-like	*Nasonia vitripennis*
Unigene7950	Transmembrane protein 62-like	*Bombus terrestris*
Unigene8015	Activin receptor type-1	*Harpegnathos saltator*
Unigene8071	Zinc finger protein 256-like	*Megachile rotundata*
Unigene8074	Ileal sodium/bile acid cotransporter-like isoform 1	*Nasonia vitripennis*
Unigene8084	Hypothetical protein SINV_06668	*Solenopsis invicta*
Unigene8208	Retinoid-inducible serine carboxypeptidase-like	*Megachile rotundata*
Unigene8231	Hypothetical protein GIP_L2_0100	*Glyptapanteles indiensis*
Unigene8233	ER degradation-enhancing alpha-mannosidase-like 3-like	*Apis florea*
Unigene8275	Hypothetical protein LOC100118474	*Nasonia vitripennis*
Unigene8439	Metaxin-2	*Camponotus floridanus*
Unigene8632	Anoctamin-5-like	*Nasonia vitripennis*
Unigene8643	Uncharacterized protein LOC100866151	*Apis florea*
Unigene8695	Hypothetical protein SINV_10590	*Solenopsis invicta*
Unigene8719	Hypothetical protein LOC100118313	*Nasonia vitripennis*
Unigene8734	Metal transporter CNNM2-like	*Apis florea*

Unigene8747	Transmembrane protein 62-like	*Megachile rotundata*
Unigene8792	Protein Malvolio-like isoform 2	*Nasonia vitripennis*
Unigene8816	Hypothetical protein LOC100746955	*Bombus impatiens*
Unigene8912	Membrane-bound transcription factor site-1 protease	*Apis florea*
Unigene9146	Constitutive coactivator of PPAR-gamma-like protein 1 homolog isoform 1	*Apis mellifera*
Unigene9209	Lectin C-type domain	*Glyptapanteles indiensis*
Unigene9326	Lipoyl synthase, mitochondrial-like	*Megachile rotundata*
Unigene9333	Serine/threonine-protein phosphatase 1 regulatory subunit 10	*Harpegnathos saltator*
Unigene9340	Venom carboxylesterase-6-like	*Apis florea*
Unigene9397	Apolipoprotein D	*Harpegnathos saltator*
Unigene9408	Hypothetical protein EAG_10732	*Camponotus floridanus*
Unigene9477	Alkaline phosphatase, tissue-nonspecific isozyme-like	*Megachile rotundata*
Unigene9512	Phospholipid hydroperoxide glutathione peroxidase, mitochondrial-like	*Nasonia vitripennis*
Unigene9581	Hypothetical protein GIP_L2_0090	*Glyptapanteles indiensis*
Unigene9781	Histidine protein methyltransferase 1 homolog	*Megachile rotundata*
Unigene9786	GPI transamidase component PIG-S-like	*Bombus terrestris*
Unigene9788	Hypothetical protein	*Cotesia congregata*
Unigene9814	P325-like protein	*Glyptapanteles flavicoxis*
Unigene9830	Venom acid phosphatase Acph-1-like	*Megachile rotundata*
Unigene9869	SID1 transmembrane family member 1-like	*Bombus impatiens*
Unigene9954	RING finger protein 13	*Acromyrmex echinatior*
Unigene9955	Peptidyl-alpha-hydroxyglycine alpha-amidating lyase 1-like isoform 1	*Apis mellifera*
Unigene9990	Nodal modulator 2-like isoform 2	*Apis mellifera*
Unigene10000	ER degradation-enhancing alpha-mannosidase-like 2	*Harpegnathos saltator*
Unigene10005	CD109 antigen-like	*Megachile rotundata*
Unigene10011	hypothetical protein EAI_01992	*Harpegnathos saltator*
Unigene10013	Transmembrane protein 62-like	*Apis florea*
Unigene10040	Beta-galactosidase-like	*Apis florea*
Unigene10071	Nodal modulator 1-like	*Nasonia vitripennis*
Unigene10113	Hypothetical protein G5I_04001	*Acromyrmex echinatior*
Unigene10264	Coiled-coil domain-containing protein 47-like	*Bombus impatiens*
Unigene10413	Membrane-bound transcription factor site-1 protease	*Apis florea*
Unigene10442	Leucine-rich repeat and immunoglobulin-like domain-containing nogo receptor-interacting protein 2-like	*Nasonia vitripennis*
Unigene10591	Hypothetical protein GIP_L1_00060	*Glyptapanteles indiensis*
Unigene10595	GI23303	*Drosophila mojavensis*
Unigene10722	Protein VAC14-like protein	*Camponotus floridanus*
Unigene10754	SID1 transmembrane family member 1	*Harpegnathos saltator*
Unigene11013	Sodium-dependent phosphate transport protein 1, chloroplastic-like	*Megachile rotundata*
Unigene11025	Hypothetical protein SINV_15078	*Solenopsis invicta*
Unigene11078	Hypothetical protein SINV_00384	*Solenopsis invicta*
Unigene11137	Sphingomyelin phosphodiesterase	*Acromyrmex echinatior*
Unigene11261	Hypothetical protein SINV_03032	*Solenopsis invicta*
Unigene11276	Odorant-binding protein 5	*Microplitis mediator*
Unigene11418	E3 ubiquitin-protein ligase HUWE1	*Acromyrmex echinatior*
Unigene11423	Laminin subunit gamma-1-like	*Megachile rotundata*
Unigene11439	PAB-dependent poly(A)-specific ribonuclease subunit 3-like	*Megachile rotundata*
Unigene11448	Sodium-coupled monocarboxylate transporter 2-like isoform 2	*Nasonia vitripennis*
Unigene11754	Zinc finger protein 830-like	*Megachile rotundata*
Unigene11792	Carboxypeptidase D	*Camponotus floridanus*
Unigene11815	Uncharacterized protein LOC100864781	*Apis florea*
Unigene11832	Lachesin	*Camponotus floridanus*
Unigene11844	PCI domain-containing protein 2-like	*Bombus terrestris*

Unigene11897	Hypothetical protein AaeL_AAEL008674	*Aedes aegypti*
Unigene11912	DnaJ-like protein subfamily C member 1	*Acromyrmex echinatior*
Unigene11936	Alkaline phosphatase, tissue-nonspecific isozyme	*Acromyrmex echinatior*
Unigene11974	Crustin-4	*Panulirus japonicus*
Unigene11995	Thioredoxin domain-containing protein 5-like	*Apis florea*
Unigene12021	Uncharacterized protein LOC100864171	*Apis florea*
Unigene12084	Leucine-rich repeats and immunoglobulin-like domains protein 3	*Harpegnathos saltator*
Unigene12314	Hypothetical protein LOC100741699	*Bombus impatiens*
Unigene12357	2-hydroxyacyl-CoA lyase 1	*Harpegnathos saltator*
Unigene12400	Post-GPI attachment to proteins factor 3-like	*Bombus terrestris*
Unigene12487	Solute carrier family 12 member 9	*Camponotus floridanus*
Unigene12540	MOB kinase activator-like 2-like	*Megachile rotundata*
Unigene12598	Sodium/hydrogen exchanger 8	*Harpegnathos saltator*
Unigene12612	Armadillo segment polarity protein	*Harpegnathos saltator*
Unigene12627	Contactin	*Harpegnathos saltator*
Unigene12652	Coiled-coil domain-containing protein 109A	*Camponotus floridanus*
Unigene12746	ER degradation-enhancing alpha-mannosidase-like 3-like	*Apis florea*
Unigene12947	Solute carrier family 12 member 9-like	*Megachile rotundata*
Unigene12984	Allatostatin C preprohormone	*Nasonia vitripennis*
Unigene13151	ER degradation-enhancing alpha-mannosidase-like 3-like	*Bombus terrestris*
Unigene13311	Thioredoxin domain-containing protein 5-like isoform 1	*Apis mellifera*
Unigene13434	E3 ubiquitin-protein ligase RNF123	*Camponotus floridanus*
Unigene13448	hypothetical protein EAG_00552	*Camponotus floridanus*
Unigene13538	Contactin	*Camponotus floridanus*
Unigene13661	Innexin inx7	*Harpegnathos saltator*
Unigene13663	Low affinity cationic amino acid transporter 2-like	*Megachile rotundata*
Unigene13676	hypothetical protein SINV_05419	*Solenopsis invicta*
Unigene13714	hypothetical protein GIP_L1_00280	*Glyptapanteles indiensis*
Unigene13453	Protein toll-like	*Megachile rotundata*
Unigene12543	Venom allergen 3	*Camponotus floridanus*
Unigene19070	Venom phospholipase A2	*Bombus ignitus*
Unigene16846	NPF-like	*Nasonia vitripennis*

Table S3 Functional division of teratocyte unigenes

Cellular structure			
	Unigene ID	ignal peptid	Homologs
N-Glycan biosynthesis	Unigene11817	SP	PREDICTED: dolichyl pyrophosphate Man9GlcNAc2 alpha-1,3-glucosyltransferase-like [Nasonia vitripennis]
	Unigene389	SP	PREDICTED: dolichyl-diphosphooligosaccharide--protein glycosyltransferase subunit 2-like [Nasonia vitripennis]
	Unigene3922	SP	Dolichyl pyrophosphate Man9GlcNAc2 alpha-1,3-glucosyltransferase [Harpegnathos saltator]
	Unigene415	SP	PREDICTED: dolichyl-diphosphooligosaccharide--protein glycosyltransferase 48 kDa subunit-like [Megachile rotundata]
	Unigene671	SP	PREDICTED: dolichyl-diphosphooligosaccharide--protein glycosyltransferase subunit 1-like [Bombus impatiens]
	CL349.Contig2	NO	Alpha-1,3-mannosyl-glycoprotein 4-beta-N-acetylglucosaminyltransferase B [Harpegnathos saltator]
	Unigene10315	NO	PREDICTED: lethal(2)neighbour of Tid protein-like, partial [Nasonia vitripennis]
	Unigene10732	NO	hypothetical protein DAPPUDRAFT_226428 [Daphnia pulex]
	Unigene11070	NO	Alpha-1,2-mannosyltransferase ALG9 [Camponotus floridanus]
	Unigene11435	NO	Endoplasmic reticulum mannosyl-oligosaccharide 1,2-alpha-mannosidase [Harpegnathos saltator]
	Unigene1165	NO	hypothetical protein SINV_01208 [Solenopsis invicta]
	Unigene11958	NO	Endoplasmic reticulum mannosyl-oligosaccharide 1,2-alpha-mannosidase [Harpegnathos saltator]
	Unigene11969	NO	PREDICTED: alpha-mannosidase 2-like [Nasonia vitripennis]
	Unigene12012	NO	PREDICTED: mannosyl-oligosaccharide glucosidase-like [Nasonia vitripennis]
	Unigene12147	NO	PREDICTED: endoplasmic reticulum mannosyl-oligosaccharide 1,2-alpha-mannosidase-like [Nasonia vitripennis]
	Unigene12419	NO	hypothetical protein [Amblyomma maculatum]
	Unigene12667	NO	PREDICTED: alpha-mannosidase 2-like [Nasonia vitripennis]
	Unigene13236	NO	Alpha-mannosidase 2 [Camponotus floridanus]
	Unigene13265	NO	Mannosyl-oligosaccharide glucosidase [Acromyrmex echinatior]
	Unigene13444	NO	PREDICTED: alpha-mannosidase 2-like [Nasonia vitripennis]
	Unigene13666	NO	hypothetical protein SINV_13340 [Solenopsis invicta]
	Unigene13809	NO	PREDICTED: alpha-1,6-mannosyl-glycoprotein 2-beta-N-acetylglucosaminyltransferase-like [Nasonia vitripennis]
	Unigene1429	NO	PREDICTED: dolichol-phosphate mannosyltransferase-like [Megachile rotundata]
	Unigene1568	NO	PREDICTED: alpha-1,3/1,6-mannosyltransferase ALG2-like [Megachile rotundata]
	Unigene2055	NO	PREDICTED: UDP-N-acetylglucosamine--dolichyl-phosphate N-acetylglucosaminephosphotransferase-like [Bombus terrestris]
	Unigene2476	NO	PREDICTED: dolichol-phosphate mannosyltransferase-like [Bombus impatiens]
	Unigene2486	NO	PREDICTED: dolichyldiphosphatase 1-like [Megachile rotundata]
	Unigene2843	NO	PREDICTED: dolichyl pyrophosphate Man9GlcNAc2 alpha-1,3-glucosyltransferase-like [Nasonia vitripennis]
	Unigene2881	NO	PREDICTED: GDP-Man:Man(3)GlcNAc(2)-PP-Dol alpha-1,2-mannosyltransferase-like [Bombus impatiens]
	Unigene3107	NO	PREDICTED: dolichol kinase-like [Megachile rotundata]
	Unigene3159	NO	PREDICTED: dolichyldiphosphatase 1-like [Megachile rotundata]
	Unigene3211	NO	PREDICTED: dolichol kinase-like [Megachile rotundata]
	Unigene3264	NO	PREDICTED: protein RFT1 homolog [Apis florea]
	Unigene3306	NO	Probable dolichyl pyrophosphate Glc1Man9GlcNAc2 alpha-1,3-glucosyltransferase [Camponotus floridanus]
	Unigene3525	NO	PREDICTED: alpha-1,3/1,6-mannosyltransferase ALG2-like [Apis florea]
	Unigene3650	NO	PREDICTED: UDP-N-acetylglucosamine transferase subunit ALG13 homolog [Nasonia vitripennis]
	Unigene385	NO	PREDICTED: dolichyl-diphosphooligosaccharide--protein glycosyltransferase subunit DAD1-like [Apis mellifera]
	Unigene4014	NO	PREDICTED: probable dolichyl pyrophosphate Glc1Man9GlcNAc2 alpha-1,3-glucosyltransferase-like isoform 2 [Bombus terrestris]
	Unigene4071	NO	Transmembrane protein 15 [Camponotus floridanus]
	Unigene4138	NO	hypothetical protein SINV_09245 [Solenopsis invicta]
	Unigene4621	NO	PREDICTED: endoplasmic reticulum mannosyl-oligosaccharide 1,2-alpha-mannosidase-like [Nasonia vitripennis]
	Unigene4705	NO	Asparagine-linked glycosylation protein 11-like protein [Camponotus floridanus]
	Unigene4998	NO	Putative alpha-1,2-glucosyltransferase ALG10-B [Harpegnathos saltator]
	Unigene501	NO	PREDICTED: magnesium transporter protein 1-like [Nasonia vitripennis]

	Unigene5195	NO	PREDICTED: alpha-1,2-mannosyltransferase ALG9-like [Megachile rotundata]
	Unigene5468	NO	PREDICTED: protein RFT1 homolog [Nasonia vitripennis]
	Unigene5581	NO	Putative alpha-1,2-glucosyltransferase ALG10-B [Camponotus floridanus]
	Unigene5648	NO	Transmembrane protein 15 [Harpegnathos saltator]
	Unigene6058	NO	dolichyl-diphosphooligosaccharide protein glycotransferase [Danaus plexippus]
	Unigene6196	NO	Asparagine-linked glycosylation protein 11-like protein [Harpegnathos saltator]
	Unigene6259	NO	hypothetical protein SINV_03838 [Solenopsis invicta]
	Unigene6624	NO	Alpha-1,2-mannosyltransferase ALG9 [Camponotus floridanus]
	Unigene6887	NO	PREDICTED: golgin subfamily A member 7-like [Nasonia vitripennis]
	Unigene6910	NO	hypothetical protein KGM_02087 [Danaus plexippus]
	Unigene734	NO	Dolichyl-diphosphooligosaccharide--protein glycosyltransferase subunit STT3A [Camponotus floridanus]
	Unigene7565	NO	Endoplasmic reticulum mannosyl-oligosaccharide 1,2-alpha-mannosidase [Camponotus floridanus]
	Unigene7696	NO	Alpha-mannosidase 2 [Camponotus floridanus]
	Unigene7810	NO	Probable dolichyl-P-Man:Man(7)GlcNAc(2)-PP-dolichyl-alpha-1, 6-mannosyltransferase [Harpegnathos saltator]
	Unigene7983	NO	PREDICTED: protein RFT1 homolog [Nasonia vitripennis]
	Unigene8011	NO	PREDICTED: UDP-N-acetylglucosamine transferase subunit ALG14 homolog [Apis mellifera]
	Unigene806	NO	PREDICTED: dolichyl-diphosphooligosaccharide--protein glycosyltransferase subunit STT3B-like [Bombus impatiens]
	Unigene832	NO	hypothetical protein SINV_13340 [Solenopsis invicta]
	Unigene833	NO	PREDICTED: dolichol-phosphate mannosyltransferase subunit 3-like [Megachile rotundata]
	Unigene8764	NO	Alpha-1,3-mannosyl-glycoprotein 4-beta-N-acetylglucosaminyltransferase B [Acromyrmex echinatior]
	Unigene8976	NO	PREDICTED: chitobiosyldiphosphodolichol beta-mannosyltransferase-like [Nasonia vitripennis]
	Unigene9097	NO	hypothetical protein EAG_12245 [Camponotus floridanus]
	Unigene954	NO	PREDICTED: neutral alpha-glucosidase AB-like [Megachile rotundata]
	CL349.Contig1	NO	Alpha-1,3-mannosyl-glycoprotein 4-beta-N-acetylglucosaminyltransferase B [Harpegnathos saltator]
**GPI-anchor biosynthesis**	Unigene11937	SP	PREDICTED: ecdysteroid UDP-glucosyltransferase-like [Nasonia vitripennis]
	Unigene12255	SP	UDP-glucuronosyltransferase 1-9 [Acromyrmex echinatior]
	Unigene4869	SP	UDP-glucuronosyltransferase 2C1 [Acromyrmex echinatior]
	Unigene9684	SP	GPI transamidase component PIG-T [Harpegnathos saltator]
	Unigene11758	NO	PREDICTED: protein O-mannosyl-transferase 2-like [Megachile rotundata]
	Unigene1240	NO	PREDICTED: stromal cell-derived factor 2-like protein 1-like [Nasonia vitripennis]
	Unigene12560	NO	PREDICTED: protein O-mannosyl-transferase 2-like [Megachile rotundata]
	Unigene12618	NO	PREDICTED: hypothetical protein LOC100679084 [Nasonia vitripennis]
	Unigene12639	NO	PREDICTED: protein O-mannosyltransferase 1-like [Nasonia vitripennis]
	Unigene1969	NO	PREDICTED: mitochondrial import receptor subunit TOM70-like [Bombus impatiens]
	Unigene4556	NO	UDP-N-acetylglucosamine--peptide N-acetylglucosaminyltransferase 110 kDa subunit [Acromyrmex echinatior]
	Unigene5032	NO	PREDICTED: UDP-N-acetylglucosamine--peptide N-acetylglucosaminyltransferase 110 kDa subunit isoform 1 [Apis mellifera]
	Unigene5459	NO	PREDICTED: UDP-N-acetylglucosamine--peptide N-acetylglucosaminyltransferase 110 kDa subunit-like isoform 2 [Apis florea]
	Unigene5997	NO	PREDICTED: UDP-N-acetylglucosamine--peptide N-acetylglucosaminyltransferase 110 kDa subunit-like [Bombus impatiens]
	Unigene6259	NO	hypothetical protein SINV_03838 [Solenopsis invicta]
	Unigene7845	NO	PREDICTED: UDP-N-acetylglucosamine--peptide N-acetylglucosaminyltransferase 110 kDa subunit-like [Megachile rotundata]
	Unigene3980	NO	PREDICTED: GPI mannosyltransferase 1-like [Megachile rotundata]
	Unigene4975	NO	N-acetylglucosaminyl-phosphatidylinositol de-N-acetylase [Harpegnathos saltator]
	Unigene5358	NO	PREDICTED: mature T-cell proliferation 1 neighbor protein-like [Apis florea]
	Unigene7338	NO	GPI mannosyltransferase 4 [Acromyrmex echinatior]
	Unigene7940	NO	PREDICTED: L antigen family member 3-like [Apis florea]
	Unigene8620	NO	Glycosylphosphatidylinositol anchor attachment 1 protein [Harpegnathos saltator]
	Unigene9483	NO	GPI transamidase component PIG-S [Acromyrmex echinatior]
	Unigene9548	NO	PREDICTED: GPI ethanolamine phosphate transferase 3-like [Megachile rotundata]
	Unigene9786	NO	PREDICTED: GPI transamidase component PIG-S-like [Bombus terrestris]
	Unigene9866	NO	Phosphatidylinositol glycan anchor biosynthesis class U protein [Camponotus floridanus]
	Unigene10167	NO	AGAP002674-PA [Anopheles gambiae str. PEST]

O-Glycan biosynthesis	Unigene11937	SP	PREDICTED: ecdysteroid UDP-glucosyltransferase-like [Nasonia vitripennis]
	Unigene12255	SP	UDP-glucuronosyltransferase 1-9 [Acromyrmex echinatior]
	Unigene4869	SP	UDP-glucuronosyltransferase 2C1 [Acromyrmex echinatior]
	Unigene11758	NO	PREDICTED: protein O-mannosyl-transferase 2-like [Megachile rotundata]
	Unigene1240	NO	PREDICTED: stromal cell-derived factor 2-like protein 1-like [Nasonia vitripennis]
	Unigene12560	NO	PREDICTED: protein O-mannosyl-transferase 2-like [Megachile rotundata]
	Unigene12618	NO	PREDICTED: hypothetical protein LOC100679084 [Nasonia vitripennis]
	Unigene12639	NO	PREDICTED: protein O-mannosyltransferase 1-like [Nasonia vitripennis]
	Unigene1969	NO	PREDICTED: mitochondrial import receptor subunit TOM70-like [Bombus impatiens]
	Unigene4556	NO	UDP-N-acetylglucosamine--peptide N-acetylglucosaminyltransferase 110 kDa subunit [Acromyrmex echinatior]
	Unigene5032	NO	PREDICTED: UDP-N-acetylglucosamine--peptide N-acetylglucosaminyltransferase 110 kDa subunit isoform 1 [Apis mellifera]
	Unigene5459	NO	PREDICTED: UDP-N-acetylglucosamine--peptide N-acetylglucosaminyltransferase 110 kDa subunit-like isoform 2 [Apis florea]
	Unigene5997	NO	PREDICTED: UDP-N-acetylglucosamine--peptide N-acetylglucosaminyltransferase 110 kDa subunit-like [Bombus impatiens]
	Unigene6259	NO	hypothetical protein SINV_03838 [Solenopsis invicta]
	Unigene7845	NO	PREDICTED: UDP-N-acetylglucosamine--peptide N-acetylglucosaminyltransferase 110 kDa subunit-like [Megachile rotundata]
	Unigene10167	NO	AGAP002674-PA [Anopheles gambiae str. PEST]
	CL22.Contig1	SP	hypothetical protein EAG_13414 [Camponotus floridanus]
	CL61.Contig5	SP	hypothetical protein GIP_L3_0010 [Glyptapanteles indiensis]
	CL61.Contig6	SP	hypothetical protein GIP_L3_0010 [Glyptapanteles indiensis]
	Unigene2547	SP	General vesicular transport factor p115 [Acromyrmex echinatior]
	Unigene350	SP	PREDICTED: paramyosin, long form-like [Nasonia vitripennis]
	Unigene5094	SP	Transport and Golgi organization protein 1 [Acromyrmex echinatior]
	Unigene96	SP	Vascular endothelial growth factor receptor 2 [Harpegnathos saltator]
	Unigene5245	NO	Abl interactor 2 [Camponotus floridanus]
	CL284.Contig3	NO	ACTB, partial [Hipposideros caffer]
	Unigene352	NO	actin [Ciona intestinalis]
	CL284.Contig1	NO	actin 5C, isoform B [Drosophila melanogaster]
	CL284.Contig2	NO	actin 5C, isoform B [Drosophila melanogaster]
	Unigene1795	NO	actin-depolymerizing factor 1 [Danaus plexippus]
	Unigene2068	NO	actin-depolymerizing factor 1 [Danaus plexippus]
	Unigene3680	NO	Actin-like protein 87C [Camponotus floridanus]
	Unigene1575	NO	Actin-related protein 3 [Harpegnathos saltator]
	Unigene6163	NO	Adapter molecule Crk [Camponotus floridanus]
	Unigene7027	NO	AGAP005160-PA [Anopheles gambiae str. PEST]
	CL339.Contig2	NO	AGAP008244-PA [Anopheles gambiae str. PEST]
	Unigene4824	NO	Arp2/3 complex p21 subunit [Spodoptera frugiperda]
	CL549.Contig1	NO	chickadee [Tribolium castaneum]
	CL549.Contig2	NO	chickadee [Tribolium castaneum]
	CL538.Contig2	NO	chloride channel, putative [Toxoplasma gondii GT1]
	Unigene3817	NO	EH domain-binding protein 1 [Camponotus floridanus]
	Unigene8404	NO	FK506-binding protein 4 [Camponotus floridanus]
	Unigene10697	NO	FYVE, RhoGEF and PH domain-containing protein 4 [Acromyrmex echinatior]
	Unigene975	NO	Growth hormone-inducible transmembrane protein [Camponotus floridanus]
	Unigene2152	NO	Guanine nucleotide-binding protein subunit gamma-1 [Camponotus floridanus]
	Unigene7501	NO	hemocyte-specific integrin alpha subunit 1 [Manduca sexta]
	CL270.Contig2	NO	hypothetical protein BRAFLDRAFT_63199 [Branchiostoma floridae]
	Unigene12621	NO	hypothetical protein EAI_15474 [Harpegnathos saltator]
	Unigene9365	NO	hypothetical protein KGM_00084 [Danaus plexippus]
	CL339.Contig1	NO	hypothetical protein KGM_00562 [Danaus plexippus]
	Unigene13811	NO	hypothetical protein KGM_12314 [Danaus plexippus]
	Unigene7629	NO	hypothetical protein PPL_08880 [Polysphondylium pallidum PN500]
	Unigene6546	NO	hypothetical protein SINV_04187 [Solenopsis invicta]
	CL51.Contig2	NO	hypothetical protein SINV_04265 [Solenopsis invicta]
	Unigene3614	NO	hypothetical protein SINV_05158 [Solenopsis invicta]
	Unigene4101	NO	hypothetical protein SINV_05984 [Solenopsis invicta]
	CL515.Contig1	NO	hypothetical protein SINV_06010 [Solenopsis invicta]
	CL515.Contig2	NO	hypothetical protein SINV_06010 [Solenopsis invicta]
	Unigene3166	NO	hypothetical protein SINV_07779 [Solenopsis invicta]
	Unigene4771	NO	hypothetical protein SINV_11664 [Solenopsis invicta]
	Unigene11278	NO	hypothetical protein SINV_12937 [Solenopsis invicta]
	Unigene11447	NO	hypothetical protein SINV_14862 [Solenopsis invicta]

	Unigene9125	NO	hypothetical protein SINV_80297 [Solenopsis invicta]
	Unigene7329	NO	hypothetical protein SINV_80305 [Solenopsis invicta]
	Unigene10125	NO	hypothetical protein SINV_80464 [Solenopsis invicta]
	Unigene11321	NO	hypothetical protein SINV_80464 [Solenopsis invicta]
	Unigene13525	NO	Insulin receptor [Camponotus floridanus]
	Unigene11935	NO	Integrin alpha-PS1 [Harpegnathos saltator]
	Unigene10194	NO	Integrin beta-PS [Acromyrmex echinatior]
	Unigene9242	NO	Integrin beta-PS [Harpegnathos saltator]
	Unigene1089	NO	Interferon regulatory factor 2-binding protein 2-A [Harpegnathos saltator]
	Unigene1539	NO	IQ motif containing GTPase activating protein homologue [Ciona intestinalis]
	Unigene11842	NO	Membrane-associated protein Hem [Harpegnathos saltator]
	Unigene6415	NO	Merlin [Acromyrmex echinatior]
	Unigene6363	NO	Mitogen-activated protein kinase 1 [Harpegnathos saltator]
	Unigene8586	NO	Moesin [Danaus plexippus]
	Unigene12637	NO	moesin/ezrin/radixin [Culex quinquefasciatus]
	Unigene13159	NO	PREDICTED: moesin/ezrin/radixin homolog 1-like [Apis florea]
	Unigene6523	NO	Myosin heavy chain, non-muscle [Acromyrmex echinatior]
	Unigene7183	NO	Myosin heavy chain, non-muscle [Acromyrmex echinatior]
	Unigene7196	NO	Myosin heavy chain, non-muscle [Acromyrmex echinatior]
	CL340.Contig1	NO	myosin regulatory light chain MRLC2 [Xenopus (Silurana) tropicalis]
	Unigene7868	NO	Palmitoyltransferase ZDHHC2 [Camponotus floridanus]
	Unigene6633	NO	Paxillin [Camponotus floridanus]
	Unigene5702	NO	Paxillin [Harpegnathos saltator]
	Unigene11213	NO	Phosphatidylinositol 3-kinase regulatory subunit alpha [Camponotus floridanus]
	Unigene3041	NO	Phosphatidylinositol-5-phosphate 4-kinase type-2 beta [Harpegnathos saltator]
	Unigene6588	NO	plasmatocyte-specific integrin beta 1 [Manduca sexta]
	Unigene593	NO	PREDICTED: actin-5C-like [Megachile rotundata]
	Unigene1966	NO	PREDICTED: actin-related protein 2/3 complex subunit 1A-like [Bombus terrestris]
	Unigene2451	NO	PREDICTED: actin-related protein 2/3 complex subunit 3-like [Megachile rotundata]
	Unigene5971	NO	PREDICTED: actin-related protein 2/3 complex subunit 4-like [Megachile rotundata]
	CL415.Contig2	NO	PREDICTED: actin-related protein 2-like isoform 2 [Nasonia vitripennis]
	Unigene3803	NO	PREDICTED: actin-related protein 2-like isoform 4 [Megachile rotundata]
	Unigene13165	NO	PREDICTED: alpha-actinin, sarcomeric-like [Megachile rotundata]
	Unigene9797	NO	PREDICTED: alpha-actinin, sarcomeric-like [Megachile rotundata]
	Unigene2037	NO	PREDICTED: alpha-actinin-4 [Oreochromis niloticus]
	Unigene3691	NO	PREDICTED: bicaudal D-related protein homolog [Megachile rotundata]
	Unigene1925	NO	PREDICTED: cdc42 homolog isoform 1 [Nasonia vitripennis]
	Unigene672	NO	PREDICTED: cofilin/actin-depolymerizing factor homolog [Megachile rotundata]
	Unigene7980	NO	PREDICTED: dual specificity mitogen-activated protein kinase kinase 2-like [Apis florea]
	Unigene6321	NO	PREDICTED: G kinase-anchoring protein 1-like [Bombus impatiens]
	Unigene11486	NO	PREDICTED: guanine nucleotide-binding protein subunit alpha homolog [Bombus terrestris]
	Unigene12762	NO	PREDICTED: guanine nucleotide-binding protein subunit alpha homolog, partial [Apis mellifera]
	Unigene8493	NO	PREDICTED: hypothetical protein LOC100123892 [Nasonia vitripennis]
	Unigene6929	NO	PREDICTED: hypothetical protein LOC100678771 isoform 1 [Nasonia vitripennis]
	Unigene11872	NO	PREDICTED: hypothetical protein LOC100741633 [Bombus impatiens]
	Unigene9359	NO	PREDICTED: hypothetical protein LOC100743083 [Bombus impatiens]
	Unigene12247	NO	PREDICTED: hypothetical protein LOC100743521 [Bombus impatiens]
	Unigene5791	NO	PREDICTED: hypothetical protein LOC100749917 [Bombus impatiens]
	Unigene7225	NO	PREDICTED: hypothetical protein LOC408577 [Apis mellifera]
	Unigene11834	NO	PREDICTED: insulin-like peptide receptor-like [Bombus terrestris]
	Unigene10003	NO	PREDICTED: integrin beta-PS-like [Bombus impatiens]
	Unigene4836	NO	PREDICTED: leucine-rich repeat flightless-interacting protein 2-like isoform 2 [Nasonia vitripennis]
	Unigene12595	NO	PREDICTED: LIM domain kinase 1-like isoform 3 [Bombus terrestris]
**Regulation of actin cytoskeleton**	Unigene7835	NO	PREDICTED: LOW QUALITY PROTEIN: integrin beta-PS [Apis mellifera]
	Unigene5508	NO	PREDICTED: LOW QUALITY PROTEIN: myosin heavy chain, non-muscle-like [Apis florea]
	Unigene9641	NO	PREDICTED: LOW QUALITY PROTEIN: phosphatidylinositol-4,5-bisphosphate 3-kinase catalytic subunit delta isoform-like [Apis florea]
	Unigene1916	NO	PREDICTED: LOW QUALITY PROTEIN: protein still life, isoform SIF type 1-like [Apis florea]
	Unigene8181	NO	PREDICTED: LOW QUALITY PROTEIN: serine/threonine-protein kinase PAK 1-like [Apis florea]

Unigene2986	NO	PREDICTED: mannosyl-oligosaccharide glucosidase-like [Apis florea]
Unigene12793	NO	PREDICTED: membrane-associated protein Hem-like [Megachile rotundata]
Unigene4920	NO	PREDICTED: mitogen-activated protein kinase 1-like [Megachile rotundata]
Unigene1048	NO	PREDICTED: moesin/ezrin/radixin homolog 1-like isoform 1 [Megachile rotundata]
Unigene11665	NO	PREDICTED: myosin heavy chain, non-muscle-like [Megachile rotundata]
Unigene3912	NO	PREDICTED: myosin heavy chain, non-muscle-like [Megachile rotundata]
Unigene8657	NO	PREDICTED: myosin heavy chain, non-muscle-like [Megachile rotundata]
Unigene8965	NO	PREDICTED: myosin heavy chain, non-muscle-like [Megachile rotundata]
Unigene1900	NO	PREDICTED: myosin heavy chain, striated muscle-like [Amphimedon queenslandica]
Unigene10196	NO	PREDICTED: paxillin-like isoform 1 [Apis florea]
Unigene7401	NO	PREDICTED: peptidyl-prolyl cis-trans isomerase FKBP4-like [Bombus impatiens]
Unigene4015	NO	PREDICTED: peptidyl-prolyl cis-trans isomerase FKBP4-like [Megachile rotundata]
Unigene7891	NO	PREDICTED: peptidyl-prolyl cis-trans isomerase FKBP4-like [Megachile rotundata]
Unigene8344	NO	PREDICTED: phosphatidylinositol 3-kinase regulatory subunit alpha-like, partial [Apis florea]
CL592.Contig1	NO	PREDICTED: phosphatidylinositol-4-phosphate 5-kinase type-1 alpha-like isoform 2 [Bombus terrestris]
Unigene7627	NO	PREDICTED: phosphatidylinositol-4-phosphate 5-kinase type-1 alpha-like isoform 2 [Bombus terrestris]
Unigene1858	NO	PREDICTED: PRKC apoptosis WT1 regulator protein-like isoform 2 [Bombus terrestris]
Unigene7847	NO	PREDICTED: probable protein BRICK1-B-like [Bombus terrestris]
Unigene5979	NO	PREDICTED: protein ECT2-like [Megachile rotundata]
Unigene9820	NO	PREDICTED: protein ECT2-like isoform 3 [Nasonia vitripennis]
Unigene8996	NO	PREDICTED: protein expanded-like [Bombus terrestris]
Unigene5016	NO	PREDICTED: protein flightless-1-like isoform 2 [Megachile rotundata]
Unigene11690	NO	PREDICTED: protein son of sevenless-like [Megachile rotundata]
Unigene3505	NO	PREDICTED: ras GTPase-activating-like protein IQGAP1-like [Oreochromis niloticus]
Unigene722	NO	PREDICTED: ras-like GTP-binding protein Rho1 [Nasonia vitripennis]
Unigene2069	NO	PREDICTED: ras-like protein 1-like [Apis florea]
Unigene25	NO	PREDICTED: ras-like protein 2-like [Nasonia vitripennis]
Unigene7452	NO	PREDICTED: ras-related C3 botulinum toxin substrate 1 [Nasonia vitripennis]
Unigene5595	NO	PREDICTED: ras-related C3 botulinum toxin substrate 1 isoform 1 [Apis mellifera]
Unigene8131	NO	PREDICTED: rho GTPase-activating protein 1-like [Megachile rotundata]
Unigene6006	NO	PREDICTED: rho-associated protein kinase 2 [Apis florea]
Unigene7671	NO	PREDICTED: rho-associated protein kinase 2 [Megachile rotundata]
Unigene8427	NO	PREDICTED: rho-associated protein kinase 2 [Megachile rotundata]
Unigene11200	NO	PREDICTED: rho-associated protein kinase 2-like [Bombus impatiens]
Unigene9295	NO	PREDICTED: rho-associated protein kinase 2-like [Bombus impatiens]
Unigene4156	NO	PREDICTED: rho-associated protein kinase 2-like isoform 2 [Nasonia vitripennis]
Unigene5630	NO	PREDICTED: rho-associated protein kinase 2-like isoform 2 [Nasonia vitripennis]
Unigene2056	NO	PREDICTED: SAP domain-containing ribonucleoprotein-like [Bombus impatiens]
Unigene11455	NO	PREDICTED: serine/threonine/tyrosine-interacting protein-like [Bombus impatiens]
Unigene5102	NO	PREDICTED: serine/threonine-protein kinase PAK 1 isoform 2 [Megachile rotundata]
Unigene5522	NO	PREDICTED: serine/threonine-protein kinase PAK 1-like isoform 2 [Bombus impatiens]
Unigene6177	NO	PREDICTED: similar to putative Rho-associated kinase, partial [Hydra magnipapillata]
Unigene5605	NO	PREDICTED: transcriptional repressor p66-beta-like [Bombus terrestris]
Unigene3230	NO	PREDICTED: tubulin polyglutamylase TTLL5-like [Apis florea]
CL227.Contig1	NO	PREDICTED: uncharacterized protein LOC100865369 isoform 3 [Apis florea]
Unigene3878	NO	PREDICTED: uncharacterized protein LOC100874943 [Megachile rotundata]
Unigene901	NO	PREDICTED: uncharacterized protein LOC100876574 [Megachile rotundata]
Unigene2535	NO	PREDICTED: uncharacterized protein LOC100876689 [Megachile rotundata]
Unigene1552	NO	PREDICTED: uncharacterized protein LOC100879778 [Megachile rotundata]
Unigene8727	NO	PREDICTED: uncharacterized protein LOC100879778 [Megachile rotundata]
CL592.Contig2	NO	PREDICTED: uncharacterized protein LOC100882813 [Megachile rotundata]
Unigene4080	NO	PREDICTED: uncharacterized protein LOC100882813 [Megachile rotundata]
Unigene5822	NO	PREDICTED: uncharacterized protein LOC100882813 [Megachile rotundata]
CL35.Contig1	NO	PREDICTED: uncharacterized protein LOC100883249 [Megachile rotundata]
CL35.Contig2	NO	PREDICTED: uncharacterized protein LOC100883249 [Megachile rotundata]
Unigene9334	NO	PREDICTED: uncharacterized protein LOC100883678 [Megachile rotundata]
Unigene4713	NO	PREDICTED: uncharacterized protein LOC100883899 [Megachile rotundata]
Unigene11795	NO	PREDICTED: vinculin-like [Megachile rotundata]
Unigene5846	NO	PREDICTED: vinculin-like [Megachile rotundata]
Unigene8948	NO	PREDICTED: vinculin-like [Megachile rotundata]
Unigene10329	NO	PREDICTED: vinculin-like isoform 2 [Bombus impatiens]

	Unigene3217	NO	Probable actin-related protein 2/3 complex subunit 2 [Camponotus floridanus]
	Unigene7112	NO	Probable actin-related protein 2/3 complex subunit 2 [Harpegnathos saltator]
	Unigene1064	NO	profilin [Bombyx mori]
	Unigene12471	NO	Protein enabled [Acromyrmex echinatior]
	Unigene12523	NO	protein phosphatase 1 catalytic subunit [Danaus plexippus]
	Unigene9765	NO	Protein phosphatase 1 regulatory subunit 12A [Harpegnathos saltator]
	Unigene5858	NO	Protein still life, isoforms C/SIF type 2 [Acromyrmex echinatior]
	CL321.Contig1	NO	Protein still life, isoforms C/SIF type 2 [Camponotus floridanus]
	CL321.Contig2	NO	Protein still life, isoforms C/SIF type 2 [Camponotus floridanus]
	Unigene8580	NO	putative syntaxin interacting protein 1 [Danaus plexippus]
	Unigene4470	NO	Ras-like GTP-binding protein RHO [Lepeophtheirus salmonis]
	Unigene6671	NO	Ras-like GTP-binding protein RHO [Lepeophtheirus salmonis]
	Unigene6140	NO	Ras-like GTP-binding protein Rho1 [Danaus plexippus]
	Unigene1894	NO	Ras-like GTP-binding protein Rho1 [Salmo salar]
	Unigene1968	NO	Ras-related protein Rac1 [Camponotus floridanus]
	Unigene9307	NO	Rho guanine nucleotide exchange factor 7 [Harpegnathos saltator]
	Unigene8839	NO	Rho-associated protein kinase 2 [Camponotus floridanus]
	Unigene3295	NO	Scaffold attachment factor B2 [Camponotus floridanus]
	Unigene11643	NO	Serine/threonine-protein kinase PAK 1 [Acromyrmex echinatior]
	Unigene6973	NO	Serine/threonine-protein kinase PAK 1 [Harpegnathos saltator]
	Unigene1037	NO	Serine/threonine-protein phosphatase alpha-1 isoform [Camponotus floridanus]
	Unigene5945	NO	serine/threonine-protein phosphatase PP1-beta catalytic subunit [Danio rerio]
	Unigene3506	NO	Serine/threonine-protein phosphatase PP1-beta catalytic subunit [Harpegnathos saltator]
	Unigene862	NO	Thymosin beta-4 [Harpegnathos saltator]
	Unigene6344	NO	Uncharacterized protein [Acromyrmex echinatior]
	Unigene10075	NO	Vinculin [Camponotus floridanus]
	Unigene7544	NO	--
	Unigene2387	NO	Cdc42 [Ascaris suum]
	CL77.Contig1	SP	40S ribosomal protein S16 [Harpegnathos saltator]
	CL77.Contig2	SP	ribosomal protein S16 [Lysiphlebus testaceipes]
	Unigene159	SP	ribosomal protein S26 [Rhodnius prolixus]
	Unigene7116	SP	PREDICTED: large proline-rich protein BAG6-like [Megachile rotundata]
	Unigene2016	NO	39S ribosomal protein L13, mitochondrial [Harpegnathos saltator]
	Unigene1734	NO	39S ribosomal protein L2, mitochondrial [Harpegnathos saltator]
	Unigene219	NO	40S ribosomal protein S10 [Camponotus floridanus]
	Unigene192	NO	40S ribosomal protein S12 [Harpegnathos saltator]
	Unigene1750	NO	40S ribosomal protein S15Aa [Caligus rogercresseyi]
	Unigene238	NO	40S ribosomal protein S23 [Camponotus floridanus]
	Unigene89	NO	40S ribosomal protein S3 [Danaus plexippus]
	Unigene199	NO	40S ribosomal protein S3a [Harpegnathos saltator]
	Unigene2360	NO	40S ribosomal protein S3a, partial [Collodictyon triciliatum]
	Unigene1093	NO	40S ribosomal protein S4 [Bombyx mori]
	Unigene170	NO	40S ribosomal protein S5 isoform 2 [Apis mellifera]
	Unigene556	NO	40S ribosomal protein S8 [Aedes aegypti]
	Unigene783	NO	40S ribosomal protein S9 [Lepeophtheirus salmonis]
	Unigene231	NO	40S ribosomal protein SA [Camponotus floridanus]
	Unigene271	NO	60S acidic ribosomal protein P2 [Camponotus floridanus]
	Unigene261	NO	60S ribosomal protein L12 [Harpegnathos saltator]
	Unigene1212	NO	60S ribosomal protein L13A, putative [Pediculus humanus corporis]
	Unigene255	NO	60S ribosomal protein L14 [Acromyrmex echinatior]
	Unigene712	NO	60S ribosomal protein L15 [Lepeophtheirus salmonis]
	Unigene251	NO	60S ribosomal protein L17 [Camponotus floridanus]
	Unigene260	NO	60S ribosomal protein L18a [Camponotus floridanus]
	Unigene200	NO	60S ribosomal protein L19 [Harpegnathos saltator]
	Unigene7	NO	60S ribosomal protein L21 [Harpegnathos saltator]
	Unigene194	NO	60S ribosomal protein L23 [Camponotus floridanus]
	Unigene189	NO	60S ribosomal protein L24 [Harpegnathos saltator]
	Unigene171	NO	60S ribosomal protein L27 [Harpegnathos saltator]
	Unigene35	NO	60S ribosomal protein L27 [Macaca fascicularis]
	Unigene266	NO	60S ribosomal protein L29 [Acromyrmex echinatior]
	Unigene163	NO	60S ribosomal protein L34 [Acromyrmex echinatior]
	Unigene233	NO	60S ribosomal protein L36 [Camponotus floridanus]
	Unigene218	NO	60S ribosomal protein L37 [Acromyrmex echinatior]
	Unigene141	NO	60S ribosomal protein L37a [Harpegnathos saltator]
	Unigene657	NO	60S ribosomal protein L38 [Danaus plexippus]
	Unigene257	NO	60S ribosomal protein L40A [Latrodectus hesperus]

Function of ribosome	Unigene182	NO	60S ribosomal protein L44 isoform 2 [Apis mellifera]
	Unigene267	NO	60S ribosomal protein L5 [Acromyrmex echinatior]
	Unigene162	NO	60S ribosomal protein L6 [Camponotus floridanus]
	Unigene179	NO	60S ribosomal protein L7 [Solenopsis invicta]
	Unigene634	NO	60S ribosomal protein L7a [Lepeophtheirus salmonis]
	CL310.Contig2	NO	60S ribosomal protein L8 [Harpegnathos saltator]
	Unigene195	NO	GK16381 [Drosophila willistoni]
	Unigene1023	NO	hypothetical protein DAPPUDRAFT_300212 [Daphnia pulex]
	Unigene168	NO	hypothetical protein DAPPUDRAFT_312612 [Daphnia pulex]
	Unigene283	NO	hypothetical protein SINV_03506 [Solenopsis invicta]
	Unigene237	NO	hypothetical protein SINV_04293 [Solenopsis invicta]
	Unigene175	NO	hypothetical protein SINV_04528 [Solenopsis invicta]
	Unigene252	NO	hypothetical protein SINV_09784 [Solenopsis invicta]
	Unigene277	NO	hypothetical protein TcasGA2_TC003842 [Tribolium castaneum]
	Unigene1279	NO	mCG130981 [Mus musculus]
	Unigene201	NO	PREDICTED: 40S ribosomal protein S11-like [Megachile rotundata]
	Unigene2454	NO	PREDICTED: 40S ribosomal protein S12, mitochondrial-like [Apis florea]
	Unigene97	NO	PREDICTED: 40S ribosomal protein S13 [Apis mellifera]
	Unigene235	NO	PREDICTED: 40S ribosomal protein S15 [Nasonia vitripennis]
	Unigene197	NO	PREDICTED: 40S ribosomal protein S15a-like [Nasonia vitripennis]
	Unigene215	NO	PREDICTED: 40S ribosomal protein S18-like [Nasonia vitripennis]
	Unigene264	NO	PREDICTED: 40S ribosomal protein S19a-like [Nasonia vitripennis]
	Unigene274	NO	PREDICTED: 40S ribosomal protein S19a-like [Nasonia vitripennis]
	Unigene228	NO	PREDICTED: 40S ribosomal protein S21-like [Nasonia vitripennis]
	Unigene204	NO	PREDICTED: 40S ribosomal protein S25-like [Bombus terrestris]
	CL292.Contig1	NO	PREDICTED: 40S ribosomal protein S27-like [Nasonia vitripennis]
	Unigene242	NO	PREDICTED: 40S ribosomal protein S28-like [Megachile rotundata]
	Unigene222	NO	PREDICTED: 40S ribosomal protein S3-like [Nasonia vitripennis]
	Unigene245	NO	PREDICTED: 40S ribosomal protein S6-like [Nasonia vitripennis]
	Unigene254	NO	PREDICTED: 40S ribosomal protein S8-like [Megachile rotundata]
	Unigene246	NO	PREDICTED: 40S ribosomal protein S9-like [Nasonia vitripennis]
	Unigene1901	NO	PREDICTED: 60S acidic ribosomal protein P0-like [Amphimedon queenslandica]
	Unigene275	NO	PREDICTED: 60S acidic ribosomal protein P1-like [Nasonia vitripennis]
	CL646.Contig2	NO	PREDICTED: 60S ribosomal protein L10a-like [Bombus impatiens]
	CL679.Contig1	NO	PREDICTED: 60S ribosomal protein L10-like [Bombus impatiens]
	CL92.Contig2	NO	PREDICTED: 60S ribosomal protein L11-like [Megachile rotundata]
	Unigene582	NO	PREDICTED: 60S ribosomal protein L12-like [Bombus terrestris]
	Unigene249	NO	PREDICTED: 60S ribosomal protein L13a-like [Nasonia vitripennis]
	CL487.Contig2	NO	PREDICTED: 60S ribosomal protein L13-like isoform 2 [Apis florea]
	CL487.Contig3	NO	PREDICTED: 60S ribosomal protein L13-like isoform 2 [Apis florea]
	Unigene276	NO	PREDICTED: 60S ribosomal protein L15-like [Megachile rotundata]
	Unigene223	NO	PREDICTED: 60S ribosomal protein L18-like [Megachile rotundata]
	Unigene241	NO	PREDICTED: 60S ribosomal protein L23a-like [Megachile rotundata]
	Unigene2670	NO	PREDICTED: 60S ribosomal protein L23a-like [Megachile rotundata]
	Unigene234	NO	PREDICTED: 60S ribosomal protein L27a-like [Bombus terrestris]
	Unigene253	NO	PREDICTED: 60S ribosomal protein L28-like [Megachile rotundata]
	CL248.Contig1	NO	PREDICTED: 60S ribosomal protein L30-like [Nasonia vitripennis]
	Unigene642	NO	PREDICTED: 60S ribosomal protein L30-like [Nasonia vitripennis]
	Unigene167	NO	PREDICTED: 60S ribosomal protein L31-like [Megachile rotundata]
	Unigene236	NO	PREDICTED: 60S ribosomal protein L35a-like [Nasonia vitripennis]
	Unigene187	NO	PREDICTED: 60S ribosomal protein L35-like [Nasonia vitripennis]
	Unigene258	NO	PREDICTED: 60S ribosomal protein L38-like [Nasonia vitripennis]
	CL9.Contig1	NO	PREDICTED: 60S ribosomal protein L39-like [Bombus impatiens]
	CL9.Contig2	NO	PREDICTED: 60S ribosomal protein L39-like [Bombus impatiens]
	CL619.Contig1	NO	PREDICTED: 60S ribosomal protein L3-like [Bombus terrestris]
	Unigene1515	NO	PREDICTED: 60S ribosomal protein L4-like [Amphimedon queenslandica]
	Unigene205	NO	PREDICTED: 60S ribosomal protein L4-like [Megachile rotundata]
	Unigene220	NO	PREDICTED: 60S ribosomal protein L9-like [Megachile rotundata]
	Unigene952	NO	PREDICTED: acyl carrier protein, mitochondrial-like [Megachile rotundata]
	Unigene1346	NO	PREDICTED: hypothetical protein LOC551870 [Apis mellifera]
	CL636.Contig1	NO	PREDICTED: probable ribosome biogenesis protein RLP24-like [Nasonia vitripennis]
	Unigene1505	NO	PREDICTED: similar to ribosomal protein L6 isoform 1 [Ciona intestinalis]
	Unigene1056	NO	PREDICTED: similar to ribosomal protein S19 isoform 1 [Ciona intestinalis]
	Unigene213	NO	PREDICTED: ubiquitin-40S ribosomal protein S27a-like isoform 1 [Nasonia vitripennis]
	Unigene3373	NO	PREDICTED: ubiquitin-like protein 4A-like isoform 2 [Apis mellifera]

	Unigene1717	NO	PREDICTED: ubiquitin-like protein FUBI-like [Nasonia vitripennis]
	Unigene225	NO	PREDICTED: ubiquitin-like protein FUBI-like [Nasonia vitripennis]
	Unigene908	NO	putative 40S ribosomal protein RPSA [Flustra foliacea]
	Unigene884	NO	putative 60S ribosomal protein RPL21 [Flustra foliacea]
	Unigene749	NO	putative Ribosomal Protein [Angiostrongylus cantonensis]
	Unigene193	NO	putative ribosomal protein L22 [Cotesia congregata]
	Unigene1205	NO	putative ribosomal protein S11 [Maconellicoccus hirsutus]
	CL580.Contig1	NO	putative ribosomal protein S4 [Cotesia congregata]
	CL580.Contig2	NO	putative ribosomal protein S4 [Cotesia congregata]
	Unigene3665	NO	RecName: Full=40S ribosomal protein SA
	Unigene6174	NO	ribosomal biogenesis protein RLP24 [Manduca sexta]
	Unigene190	NO	ribosomal protein 49 [Athalia rosae]
	CL92.Contig1	NO	ribosomal protein L11 [Bombyx mori]
	Unigene439	NO	ribosomal protein L13 [Helicoverpa zea]
	CL487.Contig1	NO	ribosomal protein L13 [Lysiphlebus testaceipes]
	Unigene702	NO	Ribosomal protein L13A [Choristoneura parallela]
	Unigene599	NO	ribosomal protein L14 [Euphydryas aurinia]
	Unigene689	NO	ribosomal protein L19 [Manduca sexta]
	Unigene620	NO	ribosomal protein L22 [Spodoptera frugiperda]
	Unigene625	NO	ribosomal protein L23A [Manduca sexta]
	Unigene214	NO	ribosomal protein L26 [Lysiphlebus testaceipes]
	CL619.Contig2	NO	ribosomal protein L3 [Bombyx mori]
	Unigene1159	NO	ribosomal protein L32 [Gryllus bimaculatus]
	Unigene781	NO	ribosomal protein L34 [Euphydryas aurinia]
	Unigene1006	NO	ribosomal protein L34 [Rhipicephalus sanguineus]
	Unigene798	NO	ribosomal protein L35A [Euphydryas aurinia]
	Unigene113	NO	ribosomal protein L4 [Heliothis virescens]
	Unigene565	NO	ribosomal protein L5 [Bombyx mori]
	Unigene568	NO	ribosomal protein L6 [Euphydryas aurinia]
	Unigene883	NO	ribosomal protein L7 [Spodoptera frugiperda]
	Unigene609	NO	ribosomal protein L7A [Manduca sexta]
	Unigene1408	NO	ribosomal protein L7-like [Ixodes scapularis]
	CL310.Contig1	NO	ribosomal protein L8 [Manduca sexta]
	Unigene938	NO	ribosomal protein L9 [Bombyx mori]
	Unigene1034	NO	ribosomal protein P0 [Manduca sexta]
	Unigene1045	NO	ribosomal protein S10 [Spodoptera frugiperda]
	Unigene650	NO	ribosomal protein S11 [Heliothis virescens]
	Unigene649	NO	ribosomal protein S11 isoform 1 [Bombyx mori]
	Unigene1626	NO	ribosomal protein S12 [Artemia franciscana]
	Unigene879	NO	ribosomal protein S13 [Spodoptera frugiperda]
	Unigene497	NO	ribosomal protein S15 [Heliconius melpomene]
	Unigene198	NO	ribosomal protein S17 [Lysiphlebus testaceipes]
	Unigene549	NO	ribosomal protein S19 [Spodoptera frugiperda]
	Unigene1701	NO	ribosomal protein S2 [Bombyx mori]
	Unigene207	NO	ribosomal protein S2 [Lysiphlebus testaceipes]
	Unigene737	NO	ribosomal protein S21 [Heliconius melpomene cythera]
	Unigene583	NO	Ribosomal protein S23 [Spodoptera frugiperda]
	Unigene902	NO	ribosomal protein S23e [Artemia franciscana]
	Unigene726	NO	ribosomal protein S24 [Heliconius melpomene]
	Unigene325	NO	ribosomal protein S25 [Euphydryas aurinia]
	Unigene1136	NO	ribosomal protein S27 [Bombyx mori]
	Unigene227	NO	ribosomal protein S29e [Sphaerius sp. APV-2005]
	Unigene120	NO	ribosomal protein S3 [Heliconius melpomene cythera]
	Unigene779	NO	ribosomal protein S30 [Manduca sexta]
	Unigene596	NO	ribosomal protein S3A [Manduca sexta]
	Unigene843	NO	ribosomal protein s4a [Ancylostoma caninum]
	Unigene615	NO	ribosomal protein S6 [Heliconius melpomene cythera]
	Unigene531	NO	ribosomal protein S7e [Pteromalus puparum]
	Unigene405	NO	ribosomal protein S8 [Lonomia obliqua]
	Unigene480	NO	ribosomal protein SA [Euphydryas aurinia]
	Unigene760	NO	Ubiquitin-40S ribosomal protein S27a [Danaus plexippus]
	CL223.Contig1	NO	tubulin subunit beta [Schistosoma mansoni]
	CL227.Contig1	NO	PREDICTED: uncharacterized protein LOC100865369 isoform 3 [Apis florea]
	CL27.Contig1	NO	beta-tubulin [Danaus plexippus]
	CL27.Contig2	NO	PREDICTED: tubulin beta chain-like [Oreochromis niloticus]
	CL27.Contig3	NO	tubulin beta-2 chain, putative [Pediculus humanus corporis]

	CL27.Contig4	NO	beta-tubulin [Agrotis ipsilon]
	CL27.Contig5	NO	beta-tubulin [Bombyx mori]
	CL27.Contig6	NO	Tubulin beta-1 chain [Camponotus floridanus]
	CL27.Contig7	NO	beta-tubulin [Danaus plexippus]
	CL284.Contig1	NO	actin 5C, isoform B [Drosophila melanogaster]
	CL284.Contig2	NO	actin 5C, isoform B [Drosophila melanogaster]
	CL284.Contig3	NO	ACTB, partial [Hipposideros caffer]
	CL317.Contig1	NO	PREDICTED: hypothetical protein LOC100118695 [Nasonia vitripennis]
	CL317.Contig2	NO	PREDICTED: hypothetical protein LOC100118695 [Nasonia vitripennis]
	CL320.Contig2	NO	PREDICTED: protein kinase C and casein kinase substrate in neurons protein 2-like isoform 1 [Nasonia vitripennis]
	CL330.Contig1	NO	14-3-3 zeta [Heliothis virescens]
	CL330.Contig2	NO	14-3-3 zeta [Heliothis virescens]
	CL415.Contig2	NO	PREDICTED: actin-related protein 2-like isoform 2 [Nasonia vitripennis]
	CL475.Contig2	NO	14-3-3 zeta [Linepithema humile]
	CL515.Contig1	NO	hypothetical protein SINV_06010 [Solenopsis invicta]
	CL515.Contig2	NO	hypothetical protein SINV_06010 [Solenopsis invicta]
	CL71.Contig2	NO	PREDICTED: tubulin alpha-1 chain-like [Megachile rotundata]
	CL71.Contig3	NO	PREDICTED: tubulin alpha-1 chain-like [Bombus terrestris]
	CL71.Contig4	NO	PREDICTED: tubulin alpha-1 chain-like [Nasonia vitripennis]
	Unigene10003	NO	PREDICTED: integrin beta-PS-like [Bombus impatiens]
	Unigene10083	NO	putative armadillo protein [Danaus plexippus]
	Unigene10194	NO	Integrin beta-PS [Acromyrmex echinatior]
	Unigene10383	NO	PREDICTED: cytoplasmic protein NCK1-like isoform 2 [Megachile rotundata]
	Unigene1089	NO	Interferon regulatory factor 2-binding protein 2-A [Harpegnathos saltator]
	Unigene11200	NO	PREDICTED: rho-associated protein kinase 2-like [Bombus impatiens]
	Unigene11278	NO	hypothetical protein SINV_12937 [Solenopsis invicta]
	Unigene1185	NO	beta-tubulin 1 [Notothenia neglecta]
	Unigene11880	NO	GG10803 [Drosophila erecta]
	Unigene12395	NO	Rho-guanine nucleotide exchange factor [Harpegnathos saltator]
	Unigene12612	NO	Armadillo segment polarity protein [Harpegnathos saltator]
	Unigene12735	NO	PREDICTED: armadillo segment polarity protein isoform 2 [Nasonia vitripennis]
	Unigene1274	NO	PREDICTED: DNA replication licensing factor MCM8 [Apis mellifera]
	Unigene1378	NO	beta-tubulin, partial [Schistocerca gregaria]
	Unigene13811	NO	hypothetical protein KGM_12314 [Danaus plexippus]
	Unigene1499	NO	hypothetical protein EAG_06465 [Camponotus floridanus]
	Unigene1575	NO	Actin-related protein 3 [Harpegnathos saltator]
	Unigene1860	NO	14-3-3 protein zeta chain [cattle, brain, Peptide, 245 aa]
	Unigene1894	NO	Ras-like GTP-binding protein Rho1 [Salmo salar]
	Unigene1925	NO	PREDICTED: cdc42 homolog isoform 1 [Nasonia vitripennis]
	Unigene1966	NO	PREDICTED: actin-related protein 2/3 complex subunit 1A-like [Bombus terrestris]
	Unigene2387	NO	Cdc42 [Ascaris suum]
	Unigene2451	NO	PREDICTED: actin-related protein 2/3 complex subunit 3-like [Megachile rotundata]
	Unigene2504	NO	hypothetical protein TRIADDRAFT_20496 [Trichoplax adhaerens]
	Unigene2594	NO	14-3-3 epsilon [Plutella xylostella]
	Unigene2759	NO	Zinc finger protein 330-like protein [Harpegnathos saltator]
	Unigene2986	NO	PREDICTED: mannosyl-oligosaccharide glucosidase-like [Apis florea]
**Cytoskeleton rearrangment**	Unigene3166	NO	hypothetical protein SINV_07779 [Solenopsis invicta]
	Unigene3217	NO	Probable actin-related protein 2/3 complex subunit 2 [Camponotus floridanus]
	Unigene3230	NO	PREDICTED: tubulin polyglutamylase TTLL5-like [Apis florea]
	Unigene3481	NO	beta-tubulin [Heliothis virescens]
	Unigene352	NO	actin [Ciona intestinalis]
	Unigene3614	NO	hypothetical protein SINV_05158 [Solenopsis invicta]
	Unigene3680	NO	Actin-like protein 87C [Camponotus floridanus]
	Unigene3803	NO	PREDICTED: actin-related protein 2-like isoform 4 [Megachile rotundata]
	Unigene4156	NO	PREDICTED: rho-associated protein kinase 2-like isoform 2 [Nasonia vitripennis]
	Unigene4174	NO	PREDICTED: LIM and SH3 domain protein F42H10.3-like isoform 1 [Bombus impatiens]
	Unigene425	NO	hypothetical protein [Trichomonas vaginalis G3]
	Unigene4470	NO	Ras-like GTP-binding protein RHO [Lepeophtheirus salmonis]
	Unigene4596	NO	PREDICTED: hypothetical protein LOC100117990 isoform 1 [Nasonia vitripennis]
	Unigene4704	NO	beta-tubulin [Bombyx mori]
	Unigene4740	NO	beta-tubulin [Danaus plexippus]
	Unigene4824	NO	Arp2/3 complex p21 subunit [Spodoptera frugiperda]
	Unigene5515	NO	PREDICTED: armadillo segment polarity protein-like [Megachile rotundata]
	Unigene5605	NO	PREDICTED: transcriptional repressor p66-beta-like [Bombus terrestris]

	Unigene5630	NO	PREDICTED: rho-associated protein kinase 2-like isoform 2 [Nasonia vitripennis]
	Unigene593	NO	PREDICTED: actin-5C-like [Megachile rotundata]
	Unigene5971	NO	PREDICTED: actin-related protein 2/3 complex subunit 4-like [Megachile rotundata]
	Unigene6006	NO	PREDICTED: rho-associated protein kinase 2 [Apis florea]
	Unigene610	NO	hypothetical protein DAPPUDRAFT_305638 [Daphnia pulex]
	Unigene6140	NO	Ras-like GTP-binding protein Rho1 [Danaus plexippus]
	Unigene6177	NO	PREDICTED: similar to putative Rho-associated kinase, partial [Hydra magnipapillata]
	Unigene6308	NO	AT-rich interactive domain-containing protein 4B [Harpegnathos saltator]
	Unigene6415	NO	Merlin [Acromyrmex echinatior]
	Unigene6588	NO	plasmatocyte-specific integrin beta 1 [Manduca sexta]
	Unigene6671	NO	Ras-like GTP-binding protein RHO [Lepeophtheirus salmonis]
	Unigene7027	NO	AGAP005160-PA [Anopheles gambiae str. PEST]
	Unigene7102	NO	Multiple C2 and transmembrane domain-containing protein 2 [Acromyrmex echinatior]
	Unigene711	NO	tubulin, alpha 4 like [Danio rerio]
	Unigene7112	NO	Probable actin-related protein 2/3 complex subunit 2 [Harpegnathos saltator]
	Unigene7157	NO	PREDICTED: drebrin-like protein-like [Nasonia vitripennis]
	Unigene722	NO	PREDICTED: ras-like GTP-binding protein Rho1 [Nasonia vitripennis]
	Unigene745	NO	14-3-3 protein epsilon [Harpegnathos saltator]
	Unigene7563	NO	Arsenite-resistance protein 2-like protein [Harpegnathos saltator]
	Unigene758	NO	Protein toll [Harpegnathos saltator]
	Unigene7670	NO	RecName: Full=Putative uncharacterized protein DDB_G0281733
	Unigene7671	NO	PREDICTED: rho-associated protein kinase 2 [Megachile rotundata]
	Unigene7835	NO	PREDICTED: LOW QUALITY PROTEIN: integrin beta-PS [Apis mellifera]
	Unigene8340	NO	Tubulin beta chain [Camponotus floridanus]
	Unigene8361	NO	PREDICTED: tyrosine-protein kinase shark-like [Nasonia vitripennis]
	Unigene8427	NO	PREDICTED: rho-associated protein kinase 2 [Megachile rotundata]
	Unigene8586	NO	Moesin [Danaus plexippus]
	Unigene8823	NO	PREDICTED: cytoplasmic protein NCK1-like isoform 2 [Megachile rotundata]
	Unigene8839	NO	Rho-associated protein kinase 2 [Camponotus floridanus]
	Unigene906	NO	PREDICTED: uncharacterized protein LOC100883453 [Megachile rotundata]
	Unigene9242	NO	Integrin beta-PS [Harpegnathos saltator]
	Unigene9295	NO	PREDICTED: rho-associated protein kinase 2-like [Bombus impatiens]
	Unigene9378	NO	PREDICTED: armadillo segment polarity protein-like [Megachile rotundata]
	Unigene975	NO	Growth hormone-inducible transmembrane protein [Camponotus floridanus]
**Other regulation on cytoskeleton**	Unigene1048	NO	moesin/ezrin/radixin [Culex quinquefasciatus]
	Unigene11254	NO	Supervillin [Camponotus floridanus]
	Unigene12637	NO	PREDICTED: moesin/ezrin/radixin homolog 1-like [Acyrthosiphon pisum]
	Unigene13165	NO	PREDICTED: alpha-actinin, sarcomeric-like [Megachile rotundata]
	Unigene2759	NO	Zinc finger protein 330-like protein [Harpegnathos saltator]
	Unigene2944	NO	Plastin-3 [Harpegnathos saltator]
	Unigene3803	NO	PREDICTED: actin-related protein 2-like isoform 4 [Megachile rotundata]
	Unigene400	NO	ezrin-radixin-moesin-binding phosphoprotein 50 [Culex quinquefasciatus]
	Unigene4156	NO	PREDICTED: rho-associated protein kinase 2-like isoform 2 [Nasonia vitripennis]
	Unigene4630	NO	Plastin-3 [Harpegnathos saltator]
	Unigene6415	NO	Merlin [Acromyrmex echinatior]
	Unigene6965	NO	Plastin-3 [Harpegnathos saltator]
	Unigene758	NO	Protein toll [Harpegnathos saltator]
	Unigene8063	NO	merlin/moesin/ezrin/radixin [Culex quinquefasciatus]
	Unigene8340	NO	Tubulin beta chain [Camponotus floridanus]
	Unigene8586	NO	PREDICTED: moesin/ezrin/radixin homolog 1-like [Nasonia vitripennis]
	Unigene952	NO	PREDICTED: acyl carrier protein, mitochondrial-like [Megachile rotundata]

Nutrient metabolism

	Unigene ID	ignal peptid	Homologs
	Unigene2994	SP	Probable pyruvate dehydrogenase E1 component subunit alpha, mitochondrial [Camponotus floridanus]
	CL421.Contig2	NO	PREDICTED: glyceraldehyde-3-phosphate dehydrogenase 2 [Nasonia vitripennis]
	CL516.Contig1	NO	phosphoglucose isomerase [Polypterus ornatipinnis]
	CL575.Contig1	NO	PREDICTED: pyruvate kinase isoform 2 [Nasonia vitripennis]
	CL575.Contig2	NO	PREDICTED: pyruvate kinase isoform 1 [Nasonia vitripennis]
	CL575.Contig3	NO	PREDICTED: pyruvate kinase isoform 2 [Nasonia vitripennis]
	CL575.Contig4	NO	PREDICTED: pyruvate kinase isoform 1 [Nasonia vitripennis]
	CL733.Contig1	NO	glucose-6-phosphate isomerase [Nasonia vitripennis]
	CL746.Contig1	NO	fructose 1,6-bisphosphate aldolase [Bombyx mori]
	CL746.Contig2	NO	fructose 1,6-bisphosphate aldolase [Bombyx mori]
	CL760.Contig1	NO	PREDICTED: retinal dehydrogenase 1-like [Megachile rotundata]

	CL760.Contig2	NO	aldehyde dehydrogenase isoform A [Lysiphlebus testaceipes]
	Unigene10041	NO	fructose 1,6-bisphosphate aldolase [Bombyx mori]
	Unigene10176	NO	enolase [Gracillariidae gen. 1 sp. ex Schinus terebinthifolius]
	Unigene10747	NO	Phosphoglucomutase [Acromyrmex echinatior]
	Unigene11567	NO	ADP-dependent glucokinase [Camponotus floridanus]
	Unigene11850	NO	Dihydrolipoyllysine-residue acetyltransferase component of pyruvate dehydrogenase complex, mitochondrial [Camponotus floridanus]
	Unigene12036	NO	aldo-keto reductase, partial [Schistocerca gregaria]
	Unigene12317	NO	PREDICTED: similar to triosephosphate isomerase [Tribolium castaneum]
	Unigene12474	NO	PREDICTED: uncharacterized protein LOC100875107 [Megachile rotundata]
	Unigene1264	NO	PREDICTED: retinal dehydrogenase 1-like [Megachile rotundata]
	Unigene12766	NO	hexokinase [Biston betularia]
	Unigene1317	NO	hypothetical protein SINV_02533 [Solenopsis invicta]
	Unigene1413	NO	Phosphoglycerate kinase [Camponotus floridanus]
	Unigene1432	NO	PREDICTED: hexokinase type 2-like isoform 1 [Nasonia vitripennis]
	Unigene1479	NO	hypothetical protein SINV_14712 [Solenopsis invicta]
	Unigene1725	NO	PREDICTED: pyruvate dehydrogenase E1 component subunit beta, mitochondrial-like [Nasonia vitripennis]
	Unigene1726	NO	Hexokinase-2 [Acromyrmex echinatior]
	Unigene1788	NO	Phosphoglycerate mutase 1 [Acromyrmex echinatior]
	Unigene1963	NO	PREDICTED: alcohol dehydrogenase class-3-like [Megachile rotundata]
	Unigene1989	NO	PREDICTED: glucose-6-phosphate 1-epimerase-like [Nasonia vitripennis]
	Unigene2041	NO	glucose-6-phosphate isomerase [Nasonia vitripennis]
	Unigene2238	NO	PREDICTED: glyceraldehyde 3-phosphate dehydrogenase-like [Saccoglossus kowalevskii]
	Unigene2494	NO	PREDICTED: hexokinase type 2-like isoform 2 [Nasonia vitripennis]
	Unigene2549	NO	PREDICTED: aldehyde dehydrogenase, mitochondrial-like [Nasonia vitripennis]
	Unigene2695	NO	aldose reductase, putative [Pediculus humanus corporis]
	Unigene2723	NO	PREDICTED: similar to triosephosphate isomerase 1b [Ciona intestinalis]
**Glycolysis / Gluconeogenesis**	Unigene3468	NO	PREDICTED: aldehyde dehydrogenase, dimeric NADP-preferring-like isoform 2 [Nasonia vitripennis]
	Unigene365	NO	Fructose-bisphosphate aldolase [Harpegnathos saltator]
	Unigene3869	NO	mitochondrial aldehyde dehydrogenase [Danaus plexippus]
	Unigene3943	NO	hypothetical protein DAPPUDRAFT_327265 [Daphnia pulex]
	Unigene418	NO	PREDICTED: coiled-coil-helix-coiled-coil-helix domain-containing protein 2, mitochondrial-like [Nasonia vitripennis]
	Unigene5128	NO	fructose-bisphosphate aldolase B [Lepidosiren paradoxa]
	Unigene5186	NO	PREDICTED: 6-phosphofructokinase, muscle type-like [Oreochromis niloticus]
	Unigene5307	NO	triosephosphate isomerase [Bombyx mori]
	Unigene5530	NO	glycerol 3-phosphate dehydrogenase [Suberites domuncula]
	Unigene5847	NO	PREDICTED: 6-phosphofructokinase, liver type [Oreochromis niloticus]
	Unigene5948	NO	triosephosphate isomerase [Trichinella spiralis]
	Unigene6230	NO	Dihydrolipoyllysine-residue acetyltransferase component of pyruvate dehydrogenase complex, mitochondrial [Harpegnathos saltator]
	Unigene6248	NO	PREDICTED: 6-phosphofructokinase, muscle type-like [Oreochromis niloticus]
	Unigene6342	NO	UPF0010 protein YMR099C [Acromyrmex echinatior]
	Unigene693	NO	PREDICTED: uncharacterized protein LOC100879794 [Megachile rotundata]
	Unigene7019	NO	PREDICTED: similar to pyruvate dehydrogenase (lipoamide) beta, partial [Hydra magnipapillata]
	Unigene7486	NO	Dihydrolipoyllysine-residue acetyltransferase component of pyruvate dehydrogenase complex, mitochondrial [Harpegnathos saltator]
	Unigene784	NO	PREDICTED: triosephosphate isomerase-like [Nasonia vitripennis]
	Unigene8037	NO	PREDICTED: aldehyde dehydrogenase X, mitochondrial-like [Nasonia vitripennis]
	Unigene8127	NO	hypothetical protein EAI_11500 [Harpegnathos saltator]
	Unigene8318	NO	PREDICTED: dihydrolipoyllysine-residue acetyltransferase component of pyruvate dehydrogenase complex, mitochondrial-like [Nasonia vitripennis]
	Unigene8368	NO	aldehyde dehydroxygenase [Heliothis virescens]
	Unigene864	NO	Septin-4 [Harpegnathos saltator]
	Unigene8650	NO	PREDICTED: dihydrolipoyllysine-residue acetyltransferase component of pyruvate dehydrogenase complex, mitochondrial-like [Nasonia vitripennis]
	Unigene881	NO	PREDICTED: enolase-like isoform 2 [Nasonia vitripennis]
	Unigene8822	NO	dihydrolipoamide dehydrogenase [Bombyx mori]
	Unigene8936	NO	PREDICTED: retinal dehydrogenase 1-like isoform 1 [Apis mellifera]
	Unigene9223	NO	PREDICTED: aldehyde dehydrogenase, dimeric NADP-preferring-like isoform 3 [Nasonia vitripennis]

	Unigene9293	NO	PREDICTED: dihydrolipoyllysine-residue acetyltransferase component of pyruvate dehydrogenase complex, mitochondrial-like [Nasonia vitripennis]
	Unigene944	NO	glucose-6-phosphate isomerase [Nasonia vitripennis]
	Unigene9489	NO	6-phosphofructokinase [Harpegnathos saltator]
	Unigene9523	NO	hypothetical protein SINV_80107 [Solenopsis invicta]
	Unigene9527	NO	PREDICTED: phosphoglucomutase-like [Bombus impatiens]
	Unigene9595	NO	hypothetical protein SINV_08321 [Solenopsis invicta]
	Unigene9657	NO	fructose 1,6-bisphosphate aldolase [Antheraea yamamai]
**Citrate cycle (TCA cycle)**	Unigene2994	SP	Probable pyruvate dehydrogenase E1 component subunit alpha, mitochondrial [Camponotus floridanus]
	Unigene772	SP	PREDICTED: protein canopy-1-like [Bombus terrestris]
	CL107.Contig2	NO	2-oxoglutarate dehydrogenase E1 component, mitochondrial [Acromyrmex echinatior]
	CL124.Contig1	NO	succinate dehydrogenase cytochrome b560 subunit, mitochondrial [Nasonia vitripennis]
	CL124.Contig2	NO	PREDICTED: succinate dehydrogenase cytochrome b560 subunit, mitochondrial-like [Apis florea]
	CL429.Contig1	NO	Isocitrate dehydrogenase [NADP] cytoplasmic [Camponotus floridanus]
	CL57.Contig1	NO	Pyruvate carboxylase, mitochondrial [Harpegnathos saltator]
	CL57.Contig2	NO	PREDICTED: pyruvate carboxylase, mitochondrial-like isoform 1 [Bombus terrestris]
	Unigene1007	NO	PREDICTED: malate dehydrogenase, mitochondrial-like [Nasonia vitripennis]
	Unigene10269	NO	putative mitochondrial malate dehydrogenase [Danaus plexippus]
	Unigene10320	NO	putative mitochondrial malate dehydrogenase [Danaus plexippus]
	Unigene11149	NO	PREDICTED: pyruvate carboxylase, mitochondrial [Megachile rotundata]
	Unigene1137	NO	PREDICTED: malate dehydrogenase, cytoplasmic-like [Nasonia vitripennis]
	Unigene11588	NO	hypothetical protein DAPPUDRAFT_46417 [Daphnia pulex]
	Unigene11682	NO	Pyruvate carboxylase, mitochondrial [Camponotus floridanus]
	Unigene11756	NO	putative citrate synthase [Danaus plexippus]
	Unigene11850	NO	Dihydrolipoyllysine-residue acetyltransferase component of pyruvate dehydrogenase complex, mitochondrial [Camponotus floridanus]
	Unigene1228	NO	PREDICTED: succinyl-CoA ligase [ADP-forming] subunit beta, mitochondrial-like [Nasonia vitripennis]
	Unigene12337	NO	isocitrate dehydrogenase [Danaus plexippus]
	Unigene12474	NO	PREDICTED: uncharacterized protein LOC100875107 [Megachile rotundata]
	Unigene1290	NO	PREDICTED: succinate dehydrogenase [ubiquinone] flavoprotein subunit, mitochondrial-like [Megachile rotundata]
	Unigene1317	NO	hypothetical protein SINV_02533 [Solenopsis invicta]
	Unigene1725	NO	PREDICTED: pyruvate dehydrogenase E1 component subunit beta, mitochondrial-like [Nasonia vitripennis]
	Unigene1738	NO	succinate dehydrogenase iron sulfur subunit B [Lysiphlebus testaceipes]
	Unigene1876	NO	Succinyl-CoA ligase [GDP-forming] subunit alpha, mitochondrial [Camponotus floridanus]
	Unigene1928	NO	PREDICTED: cytoplasmic aconitate hydratase [Megachile rotundata]
	Unigene1987	NO	PREDICTED: aconitate hydratase, mitochondrial-like isoform 2 [Nasonia vitripennis]
	Unigene2044	NO	PREDICTED: isocitrate dehydrogenase [NAD] subunit gamma 1, mitochondrial-like [Nasonia vitripennis]
	Unigene2076	NO	--
	Unigene2153	NO	PREDICTED: dihydrolipoyllysine-residue succinyltransferase component of 2-oxoglutarate dehydrogenase complex, mitochondrial-like [Apis florea]
	Unigene2190	NO	PREDICTED: isocitrate dehydrogenase [NAD] subunit gamma 1, mitochondrial-like isoform 1 [Bombus terrestris]
	Unigene2213	NO	hypothetical protein SINV_07766 [Solenopsis invicta]
	Unigene2231	NO	PREDICTED: cytoplasmic aconitate hydratase [Megachile rotundata]
	Unigene2616	NO	PREDICTED: dihydrolipoyllysine-residue succinyltransferase component of 2-oxoglutarate dehydrogenase complex, mitochondrial-like [Nasonia vitripennis]
	Unigene2794	NO	Probable citrate synthase 1, mitochondrial [Harpegnathos saltator]
	Unigene2856	NO	succinate dehydrogenase [ubiquinone] cytochrome b small subunit, mitochondrial [Nasonia vitripennis]
	Unigene3119	NO	probable isocitrate dehydrogenase 3 (NAD+) alpha [Lysiphlebus testaceipes]
	Unigene3126	NO	PREDICTED: aconitate hydratase, mitochondrial-like [Bombus impatiens]
	Unigene3303	NO	PREDICTED: dihydrolipoyllysine-residue succinyltransferase component of 2-oxoglutarate dehydrogenase complex, mitochondrial-like [Megachile rotundata]
	Unigene3445	NO	Probable isocitrate dehydrogenase [NAD] subunit beta, mitochondrial [Harpegnathos saltator]
	Unigene3511	NO	PREDICTED: ATP-citrate synthase-like isoform 1 [Nasonia vitripennis]
	Unigene3581	NO	PREDICTED: ATP-citrate synthase-like [Bombus impatiens]
	Unigene3674	NO	PREDICTED: ATP-citrate synthase-like isoform 1 [Nasonia vitripennis]
	Unigene3714	NO	PREDICTED: ATP-citrate synthase-like [Bombus impatiens]

	Unigene3783	NO	PREDICTED: ATP-citrate synthase-like [Megachile rotundata]
	Unigene3943	NO	hypothetical protein DAPPUDRAFT_327265 [Daphnia pulex]
	Unigene3955	NO	hypothetical protein SINV_06506 [Solenopsis invicta]
	Unigene418	NO	PREDICTED: coiled-coil-helix-coiled-coil-helix domain-containing protein 2, mitochondrial-like [Nasonia vitripennis]
	Unigene4285	NO	PREDICTED: probable citrate synthase 2, mitochondrial-like [Bombus impatiens]
	Unigene4674	NO	Probable fumarate hydratase, mitochondrial [Harpegnathos saltator]
	Unigene4724	NO	PREDICTED: probable citrate synthase 1, mitochondrial-like [Megachile rotundata]
	Unigene4909	NO	ATP-citrate synthase [Camponotus floridanus]
	Unigene6230	NO	Dihydrolipoyllysine-residue acetyltransferase component of pyruvate dehydrogenase complex, mitochondrial [Harpegnathos saltator]
	Unigene6243	NO	PREDICTED: pyruvate carboxylase, mitochondrial isoform 2 [Apis florea]
	Unigene697	NO	PREDICTED: protein kish-A-like [Bombus impatiens]
	Unigene7019	NO	PREDICTED: similar to pyruvate dehydrogenase (lipoamide) beta, partial [Hydra magnipapillata]
	Unigene7486	NO	Dihydrolipoyllysine-residue acetyltransferase component of pyruvate dehydrogenase complex, mitochondrial [Harpegnathos saltator]
	Unigene8127	NO	hypothetical protein EAI_11500 [Harpegnathos saltator]
	Unigene8318	NO	PREDICTED: dihydrolipoyllysine-residue acetyltransferase component of pyruvate dehydrogenase complex, mitochondrial-like [Nasonia vitripennis]
	Unigene8490	NO	PREDICTED: ATP-citrate synthase-like [Megachile rotundata]
	Unigene8650	NO	PREDICTED: dihydrolipoyllysine-residue acetyltransferase component of pyruvate dehydrogenase complex, mitochondrial-like [Nasonia vitripennis]
	Unigene8822	NO	dihydrolipoamide dehydrogenase [Bombyx mori]
	Unigene9224	NO	hypothetical protein KGM_01506 [Danaus plexippus]
	Unigene9293	NO	PREDICTED: dihydrolipoyllysine-residue acetyltransferase component of pyruvate dehydrogenase complex, mitochondrial-like [Nasonia vitripennis]
	Unigene9510	NO	hypothetical protein SINV_00337 [Solenopsis invicta]
	Unigene9665	NO	Probable fumarate hydratase, mitochondrial [Harpegnathos saltator]
	CL107.Contig1	NO	PREDICTED: 2-oxoglutarate dehydrogenase, mitochondrial-like [Megachile rotundata]
**Pentose phosphate pathway**	Unigene2141	SP	PREDICTED: regucalcin-like [Apis mellifera]
	CL733.Contig1	NO	glucose-6-phosphate isomerase [Nasonia vitripennis]
	CL746.Contig1	NO	fructose 1,6-bisphosphate aldolase [Bombyx mori]
	CL746.Contig2	NO	fructose 1,6-bisphosphate aldolase [Bombyx mori]
	Unigene10041	NO	fructose 1,6-bisphosphate aldolase [Bombyx mori]
	Unigene10747	NO	Phosphoglucomutase [Acromyrmex echinatior]
	Unigene1141	NO	PREDICTED: transaldolase-like isoform 1 [Megachile rotundata]
	Unigene11	NO	PREDICTED: ribose-phosphate pyrophosphokinase 1-like isoform 1 [Megachile rotundata]
	Unigene12831	NO	3-hydroxyisobutyrate dehydrogenase [Danaus plexippus]
	Unigene2041	NO	glucose-6-phosphate isomerase [Nasonia vitripennis]
	Unigene2400	NO	PREDICTED: 6-phosphogluconate dehydrogenase, decarboxylating-like [Nasonia vitripennis]
	Unigene3042	NO	6-phosphogluconolactonase [Heterocephalus glaber]
	Unigene3175	NO	PREDICTED: ribulose-phosphate 3-epimerase-like [Megachile rotundata]
	Unigene3341	NO	PREDICTED: putative oxidoreductase GLYR1 homolog [Megachile rotundata]
	Unigene365	NO	Fructose-bisphosphate aldolase [Harpegnathos saltator]
	Unigene4044	NO	PREDICTED: 6-phosphogluconate dehydrogenase, decarboxylating-like [Bombus impatiens]
	Unigene4957	NO	PREDICTED: ribose-5-phosphate isomerase [Nasonia vitripennis]
	Unigene4986	NO	PREDICTED: glucose-6-phosphate 1-dehydrogenase-like [Megachile rotundata]
	Unigene5128	NO	fructose-bisphosphate aldolase B [Lepidosiren paradoxa]
	Unigene5186	NO	PREDICTED: 6-phosphofructokinase, muscle type-like [Oreochromis niloticus]
	Unigene5847	NO	PREDICTED: 6-phosphofructokinase, liver type [Oreochromis niloticus]
	Unigene6248	NO	PREDICTED: 6-phosphofructokinase, muscle type-like [Oreochromis niloticus]
	Unigene6908	NO	Glucose-6-phosphate 1-dehydrogenase [Camponotus floridanus]
	Unigene7905	NO	Phosphoribosyl pyrophosphate synthetase-associated protein 2 [Harpegnathos saltator]
	Unigene854	NO	PREDICTED: 6-phosphogluconolactonase-like [Nasonia vitripennis]
	Unigene864	NO	Septin-4 [Harpegnathos saltator]
	Unigene8851	NO	Glucose-6-phosphate 1-dehydrogenase [Acromyrmex echinatior]
	Unigene8973	NO	PREDICTED: putative deoxyribose-phosphate aldolase-like [Megachile rotundata]
	Unigene9308	NO	hypothetical protein SINV_01997 [Solenopsis invicta]
	Unigene944	NO	glucose-6-phosphate isomerase [Nasonia vitripennis]
	Unigene946	NO	PREDICTED: transketolase-like protein 2-like isoform 2 [Bombus terrestris]
	Unigene9489	NO	6-phosphofructokinase [Harpegnathos saltator]
	Unigene9527	NO	PREDICTED: phosphoglucomutase-like [Bombus impatiens]

	Unigene9595	NO	hypothetical protein SINV_08321 [Solenopsis invicta]
	Unigene9657	NO	fructose 1,6-bisphosphate aldolase [Antheraea yamamai]
	CL516.Contig1	NO	phosphoglucose isomerase [Polypterus ornatipinnis]
**Starch and sucrose metabolism**	Unigene11937	SP	PREDICTED: ecdysteroid UDP-glucosyltransferase-like [Nasonia vitripennis]
	Unigene12255	SP	UDP-glucuronosyltransferase 1-9 [Acromyrmex echinatior]
	Unigene4869	SP	UDP-glucuronosyltransferase 2C1 [Acromyrmex echinatior]
	CL142.Contig2	NO	PREDICTED: LOW QUALITY PROTEIN: glycogen debranching enzyme-like [Apis florea]
	CL3.Contig1	NO	Reticulon-1 [Camponotus floridanus]
	CL3.Contig2	NO	Reticulon-1 [Camponotus floridanus]
	CL392.Contig1	NO	RecName: Full=Alpha,alpha-trehalose-phosphate synthase [UDP-forming] B; AltName: Full=Trehalose-6-phosphate synthase B; AltName: Full=UDP-glucose-glucosephosphate glucosyltransferase B
	CL516.Contig1	NO	phosphoglucose isomerase [Polypterus ornatipinnis]
	CL733.Contig1	NO	glucose-6-phosphate isomerase [Nasonia vitripennis]
	CL97.Contig1	NO	PREDICTED: UTP--glucose-1-phosphate uridylyltransferase isoform 1 [Apis mellifera]
	CL97.Contig2	NO	PREDICTED: UTP--glucose-1-phosphate uridylyltransferase isoform 1 [Apis mellifera]
	Unigene10091	NO	glycogen phosphorylase [Culex quinquefasciatus]
	Unigene10406	NO	PREDICTED: UDP-glucose 6-dehydrogenase-like [Bombus terrestris]
	Unigene10539	NO	PREDICTED: trehalase-like [Bombus impatiens]
	Unigene10747	NO	Phosphoglucomutase [Acromyrmex echinatior]
	Unigene12618	NO	PREDICTED: hypothetical protein LOC100679084 [Nasonia vitripennis]
	Unigene12658	NO	PREDICTED: UDP-glucuronic acid decarboxylase 1-like [Bombus terrestris]
	Unigene12766	NO	hexokinase [Biston betularia]
	Unigene13448	NO	hypothetical protein EAG_00552 [Camponotus floridanus]
	Unigene13527	NO	PREDICTED: LOW QUALITY PROTEIN: alpha,alpha-trehalose-phosphate synthase [UDP-forming] A-like [Apis florea]
	Unigene13558	NO	PREDICTED: trehalase isoform 2 [Nasonia vitripennis]
	Unigene1432	NO	PREDICTED: hexokinase type 2-like isoform 1 [Nasonia vitripennis]
	Unigene1479	NO	hypothetical protein SINV_14712 [Solenopsis invicta]
	Unigene1726	NO	Hexokinase-2 [Acromyrmex echinatior]
	Unigene2041	NO	glucose-6-phosphate isomerase [Nasonia vitripennis]
	Unigene2290	NO	reticulon/nogo [Danaus plexippus]
	Unigene2494	NO	PREDICTED: hexokinase type 2-like isoform 2 [Nasonia vitripennis]
	Unigene3060	NO	PREDICTED: LOW QUALITY PROTEIN: glycogen debranching enzyme-like [Apis florea]
	Unigene3691	NO	PREDICTED: bicaudal D-related protein homolog [Megachile rotundata]
	Unigene421	NO	Maltase 1 [Harpegnathos saltator]
	Unigene4401	NO	PREDICTED: putative glycogen [starch] synthase-like [Apis florea]
	Unigene4481	NO	PREDICTED: glycogen debranching enzyme-like [Bombus impatiens]
	Unigene4835	NO	PREDICTED: LOW QUALITY PROTEIN: glycogen debranching enzyme-like [Apis florea]
	Unigene4940	NO	PREDICTED: 1,4-alpha-glucan-branching enzyme-like [Nasonia vitripennis]
	Unigene5004	NO	PREDICTED: 1,4-alpha-glucan-branching enzyme-like [Megachile rotundata]
	Unigene5062	NO	trehalase precursor [Apis mellifera]
	Unigene5586	NO	UTP--glucose-1-phosphate uridylyltransferase [Lepeophtheirus salmonis]
	Unigene5697	NO	Putative glycogen [starch] synthase [Acromyrmex echinatior]
	Unigene5774	NO	PREDICTED: UDP-glucose 6-dehydrogenase-like [Apis mellifera]
	Unigene5821	NO	1,4-alpha-glucan-branching enzyme [Harpegnathos saltator]
	Unigene5940	NO	RecName: Full=Alpha,alpha-trehalose-phosphate synthase [UDP-forming] A; AltName: Full=Trehalose-6-phosphate synthase A; AltName: Full=UDP-glucose-glucosephosphate glucosyltransferase A
	Unigene5964	NO	PREDICTED: putative glycogen [starch] synthase-like [Megachile rotundata]
	Unigene6443	NO	PREDICTED: alpha,alpha-trehalose-phosphate synthase [UDP-forming] A-like [Nasonia vitripennis]
	Unigene6498	NO	PREDICTED: alpha,alpha-trehalose-phosphate synthase [UDP-forming] A-like [Megachile rotundata]
	Unigene7101	NO	PREDICTED: UDP-glucose 6-dehydrogenase-like [Bombus terrestris]
	Unigene944	NO	glucose-6-phosphate isomerase [Nasonia vitripennis]
	Unigene9527	NO	PREDICTED: phosphoglucomutase-like [Bombus impatiens]
	Unigene9595	NO	hypothetical protein SINV_08321 [Solenopsis invicta]
	CL142.Contig1	NO	PREDICTED: LOW QUALITY PROTEIN: glycogen debranching enzyme-like [Apis florea]
	Unigene2202	SP	PREDICTED: uncharacterized protein LOC100865627 [Apis florea]
	Unigene4911	SP	PREDICTED: V-type proton ATPase 116 kDa subunit a isoform 1-like isoform 2 [Nasonia vitripennis]

Unigene5701	SP	V-type proton ATPase 116 kDa subunit a isoform 1 [Acromyrmex echinatior]
CL124.Contig2	NO	PREDICTED: succinate dehydrogenase cytochrome b560 subunit, mitochondrial-like [Apis florea]
CL205.Contig1	NO	PREDICTED: NADH dehydrogenase [ubiquinone] flavoprotein 1, mitochondrial-like [Apis florea]
CL205.Contig2	NO	PREDICTED: NADH dehydrogenase [ubiquinone] flavoprotein 1, mitochondrial [Apis mellifera]
CL226.Contig1	NO	PREDICTED: V-type proton ATPase subunit d-like [Megachile rotundata]
CL231.Contig1	NO	ATP synthase subunit alpha, mitochondrial [Harpegnathos saltator]
CL352.Contig1	NO	cytochrome c oxidase subunit I [Cotesia vestalis]
CL352.Contig2	NO	cytochrome oxidase subunit I [Bucculatrix artemisiella]
CL352.Contig3	NO	cytochrome c oxidase subunit I [Cotesia vestalis]
CL374.Contig1	NO	ATP synthase subunit beta, mitochondrial [Harpegnathos saltator]
CL374.Contig2	NO	ATP-synthase subunit beta [Schistocerca gregaria]
CL374.Contig3	NO	ATP synthase subunit beta, putative [Pediculus humanus corporis]
CL401.Contig2	NO	PREDICTED: ATP synthase subunit epsilon, mitochondrial-like isoform 1 [Apis mellifera]
CL537.Contig1	NO	Inorganic pyrophosphatase, putative [Pediculus humanus corporis]
CL560.Contig1	NO	cytochrome b [Cotesia vestalis]
CL597.Contig1	NO	cytochrome b, partial (mitochondrion) [Caligo atreus]
CL597.Contig2	NO	cytochrome b [Cotesia vestalis]
CL67.Contig1	NO	Vacuolar proton pump subunit H [Camponotus floridanus]
CL67.Contig2	NO	V-type proton ATPase subunit H [Acromyrmex echinatior]
CL685.Contig1	NO	vacuolar H+-ATPase v1 sector subunit D [Glossina morsitans morsitans]
CL685.Contig2	NO	PREDICTED: V-type proton ATPase subunit D 1-like [Nasonia vitripennis]
CL740.Contig1	NO	PREDICTED: NADH dehydrogenase [ubiquinone] flavoprotein 1, mitochondrial-like [Megachile rotundata]
CL750.Contig1	NO	PREDICTED: ATP synthase lipid-binding protein, mitochondrial-like [Megachile rotundata]
Unigene10094	NO	V-ATPase subunit A [Ostrinia furnacalis]
Unigene1012	NO	Cytochrome b-c1 complex subunit 2, mitochondrial [Camponotus floridanus]
Unigene1014	NO	PREDICTED: NADH dehydrogenase [ubiquinone] iron-sulfur protein 7, mitochondrial-like [Apis florea]
Unigene10226	NO	PREDICTED: TBC1 domain family member 24-like isoform 3 [Megachile rotundata]
Unigene1043	NO	PREDICTED: cytochrome c oxidase subunit 6C-like [Acyrthosiphon pisum]
Unigene1044	NO	GF11341 [Drosophila ananassae]
Unigene10590	NO	hypothetical protein KGM_04009 [Danaus plexippus]
Unigene1074	NO	NADH dehydrogenase [ubiquinone] 1 alpha subcomplex subunit 13 [Camponotus floridanus]
Unigene1090	NO	PREDICTED: similar to NADH:ubiquinone dehydrogenase, putative [Tribolium castaneum]
Unigene10978	NO	hypothetical protein DAPPUDRAFT_308098 [Daphnia pulex]
Unigene11284	NO	TBC1 domain family member 24 [Camponotus floridanus]
Unigene1135	NO	PREDICTED: inorganic pyrophosphatase-like [Megachile rotundata]
Unigene11641	NO	unnamed protein product [Tetraodon nigroviridis]
Unigene11691	NO	vacuolar ATPase subunit C [Bombyx mori]
Unigene11731	NO	GE14316 [Drosophila yakuba]
Unigene11808	NO	cytochrome c oxidase subunit II [Cotesia vestalis]
Unigene1229	NO	PREDICTED: v-type proton ATPase 21 kDa proteolipid subunit-like [Apis mellifera]
Unigene1239	NO	PREDICTED: V-type proton ATPase subunit B-like [Megachile rotundata]
Unigene1243	NO	GI19985 [Drosophila mojavensis]
Unigene1289	NO	cytochrome oxidase subunit I [Cotesia chilonis]
Unigene1290	NO	PREDICTED: succinate dehydrogenase [ubiquinone] flavoprotein subunit, mitochondrial-like [Megachile rotundata]
Unigene1327	NO	hypothetical protein SINV_09347 [Solenopsis invicta]
Unigene1350	NO	putative V-ATPase C-subunit [Lutzomyia longipalpis]
Unigene13656	NO	cytochrome oxidase subunit II [Xyrosaris lichneuta]
Unigene1371	NO	NADH dehydrogenase [ubiquinone] 1 beta subcomplex subunit 8, mitochondrial [Nasonia vitripennis]
Unigene1404	NO	hypothetical protein AND_29000 [Anopheles darlingi]
Unigene1440	NO	NADH dehydrogenase (ubiquinone) 1 beta subcomplex, 7 [Tribolium castaneum]
Unigene1447	NO	NADH dehydrogenase [ubiquinone] iron-sulfur protein 6, mitochondrial [Nasonia vitripennis]
Unigene1546	NO	PREDICTED: hypothetical protein LOC100119331 [Nasonia vitripennis]
Unigene1574	NO	AGAP009602-PA [Anopheles gambiae str. PEST]
Unigene1603	NO	PREDICTED: V-type proton ATPase catalytic subunit A-like [Megachile rotundata]

Oxidative phosphorylation	Unigene1611	NO	PREDICTED: probable NADH dehydrogenase [ubiquinone] 1 alpha subcomplex subunit 12-like [Megachile rotundata]
	Unigene1633	NO	NADH dehydrogenase [ubiquinone] iron-sulfur protein 8, mitochondrial [Nasonia vitripennis]
	Unigene1646	NO	NADH dehydrogenase [Culex quinquefasciatus]
	Unigene1653	NO	AGAP012374-PA [Anopheles gambiae str. PEST]
	Unigene1688	NO	GD20916 [Drosophila simulans]
	Unigene1736	NO	AGAP009824-PA [Anopheles gambiae str. PEST]
	Unigene1737	NO	PREDICTED: V-type proton ATPase subunit E-like isoform 1 [Megachile rotundata]
	Unigene1738	NO	succinate dehydrogenase iron sulfur subunit B [Lysiphlebus testaceipes]
	Unigene1749	NO	NADH dehydrogenase [ubiquinone] 1 beta subcomplex subunit 2, mitochondrial [Camponotus floridanus]
	Unigene1800	NO	NADH dehydrogenase (ubiquinone) 1 alpha subcomplex, 9, 39kDa [Nasonia vitripennis]
	Unigene1833	NO	NADH dehydrogenase [ubiquinone] 1 alpha subcomplex subunit 2 [Camponotus floridanus]
	Unigene1986	NO	V-type proton ATPase subunit C [Acromyrmex echinatior]
	Unigene2076	NO	--
	Unigene2201	NO	PREDICTED: V-type proton ATPase subunit e 2-like [Megachile rotundata]
	Unigene2224	NO	NADH dehydrogenase [Lysiphlebus testaceipes]
	Unigene2244	NO	AGAP006918-PA [Anopheles gambiae str. PEST]
	Unigene2346	NO	NADH dehydrogenase [ubiquinone] flavoprotein 2, mitochondrial [Nasonia vitripennis]
	Unigene2452	NO	PREDICTED: cytochrome c oxidase copper chaperone-like [Nasonia vitripennis]
	Unigene2537	NO	RecName: Full=ATP synthase lipid-binding protein [Manduca sexta]
	Unigene2660	NO	hypothetical protein KGM_13250 [Danaus plexippus]
	Unigene2666	NO	PREDICTED: V-type proton ATPase 116 kDa subunit a isoform 1-like isoform 1 [Nasonia vitripennis]
	Unigene2697	NO	cytochrome c oxidase polypeptide Vb [Bombyx mori]
	Unigene270	NO	PREDICTED: similar to H+-ATPase V-type subunit [Tribolium castaneum]
	Unigene2716	NO	PREDICTED: v-type proton ATPase subunit F 1-like [Bombus terrestris]
	Unigene2856	NO	succinate dehydrogenase [ubiquinone] cytochrome b small subunit, mitochondrial [Nasonia vitripennis]
	Unigene2903	NO	hypothetical protein KGM_05439 [Danaus plexippus]
	Unigene2980	NO	vacuolar ATP synthase subunit G [Bombyx mori]
	Unigene2981	NO	hypothetical protein SINV_03850 [Solenopsis invicta]
	Unigene3002	NO	PREDICTED: cytochrome c oxidase assembly protein COX11, mitochondrial-like [Nasonia vitripennis]
	Unigene3033	NO	PREDICTED: V-type proton ATPase 21 kDa proteolipid subunit-like [Apis florea]
	Unigene3081	NO	PREDICTED: V-type proton ATPase subunit H-like isoform 1 [Megachile rotundata]
	Unigene3334	NO	H+-ATPase B subunit, partial [Bos taurus]
	Unigene3385	NO	V-type ATPase B subunit [Oncorhynchus mykiss]
	Unigene3405	NO	ATP synthase [Bombyx mori]
	Unigene346	NO	GJ13165 [Drosophila virilis]
	Unigene372	NO	PREDICTED: hypothetical protein LOC100115623 isoform 1 [Nasonia vitripennis]
	Unigene3767	NO	PREDICTED: uncharacterized protein LOC100874943 [Megachile rotundata]
	Unigene3807	NO	V-type proton ATPase subunit F [Danaus plexippus]
	Unigene3882	NO	PREDICTED: NADH dehydrogenase [ubiquinone] iron-sulfur protein 4, mitochondrial-like [Megachile rotundata]
	Unigene3904	NO	Similar to CG4692 [Heliconius melpomene]
	Unigene4061	NO	hypothetical protein AND_01489 [Anopheles darlingi]
	Unigene4072	NO	hypothetical protein KGM_03909 [Danaus plexippus]
	Unigene4107	NO	hypothetical protein SINV_09114 [Solenopsis invicta]
	Unigene411	NO	cytochrome c oxidase subunit VI [Microplitis mediator]
	Unigene4122	NO	mitochondrial cytochrome c oxidase subunit VIa [Bombyx mori]
	Unigene426	NO	cytochrome c oxidase-like protein [Glyptapanteles flavicoxis]
	Unigene4333	NO	H+ transporting ATP synthase gamma subunit [Danaus plexippus]
	Unigene438	NO	cytochrome b-c1 complex subunit 9 [Nasonia vitripennis]
	Unigene4535	NO	hypothetical protein SINV_09114 [Solenopsis invicta]
	Unigene4584	NO	PREDICTED: NADH-ubiquinone oxidoreductase 75 kDa subunit, mitochondrial-like [Bombus terrestris]
	Unigene4624	NO	PREDICTED: cytochrome c oxidase assembly protein COX15 homolog [Bombus terrestris]
	Unigene467	NO	hypothetical protein SINV_11135 [Solenopsis invicta]
	Unigene468	NO	PREDICTED: v-type proton ATPase subunit G-like [Bombus terrestris]
	Unigene4761	NO	hypothetical protein AND_17443 [Anopheles darlingi]
	Unigene4852	NO	hypothetical protein KGM_08437 [Danaus plexippus]

Unigene4885	NO	ubiquinol-cytochrome C reductase complex protein [Danaus plexippus]
Unigene4959	NO	ATP synthase [Danaus plexippus]
Unigene5046	NO	PREDICTED: v-type proton ATPase 116 kDa subunit a isoform 1-like [Bombus terrestris]
Unigene505	NO	ATP synthase subunit g, mitochondrial [Nasonia vitripennis]
Unigene5072	NO	putative NADH:ubiquinone dehydrogenase [Danaus plexippus]
Unigene520	NO	cytochrome c oxidase subunit 4 isoform 1, mitochondrial [Nasonia vitripennis]
Unigene533	NO	ATP synthase subunit b, mitochondrial [Acromyrmex echinatior]
Unigene5492	NO	hypothetical protein KGM_00095 [Danaus plexippus]
Unigene553	NO	PREDICTED: cytochrome b-c1 complex subunit 6, mitochondrial-like [Megachile rotundata]
Unigene558	NO	AGAP008724-PA [Anopheles gambiae str. PEST]
Unigene560	NO	cytochrome c oxidase subunit III [Adoxophyes honmai]
Unigene564	NO	PREDICTED: similar to mitochondrial ATP synthase coupling factor 6 [Tribolium castaneum]
Unigene5658	NO	PREDICTED: V-type proton ATPase 116 kDa subunit a isoform 1-like isoform 2 [Megachile rotundata]
Unigene579	NO	cytochrome c oxidase subunit VIb isoform 1 [Rhipicephalus sanguineus]
Unigene581	NO	GI20386 [Drosophila mojavensis]
Unigene5855	NO	H+ transporting ATP synthase subunit e [Bombyx mori]
Unigene5893	NO	PREDICTED: protoheme IX farnesyltransferase, mitochondrial-like isoform 1 [Nasonia vitripennis]
Unigene6004	NO	GK12172 [Drosophila willistoni]
Unigene6060	NO	lethal neo18 protein [Danaus plexippus]
Unigene608	NO	hypothetical protein EAG_05916 [Camponotus floridanus]
Unigene614	NO	Cytochrome b-c1 complex subunit Rieske, mitochondrial [Camponotus floridanus]
Unigene624	NO	ATP synthase subunit gamma, mitochondrial [Nasonia vitripennis]
Unigene647	NO	PREDICTED: cytochrome b-c1 complex subunit Rieske, mitochondrial-like [Cavia porcellus]
Unigene6540	NO	Vacuolar proton translocating ATPase 116 kDa subunit a isoform 1 [Harpegnathos saltator]
Unigene6601	NO	H+ transporting ATP synthase subunit g [Danaus plexippus]
Unigene6630	NO	cytochrome c oxidase [Danaus plexippus]
Unigene663	NO	Cytochrome b-c1 complex subunit 7 [Camponotus floridanus]
Unigene6642	NO	NADH dehydrogenase [Danaus plexippus]
Unigene6695	NO	unnamed protein product [Heliconius melpomene]
Unigene6839	NO	V-type proton ATPase subunit E [Danaus plexippus]
Unigene6880	NO	NADH dehydrogenase subunit 1 [Phthonandria atrilineata]
Unigene6884	NO	H+ transporting ATP synthase O subunit isoform 1 [Bombyx mori]
Unigene694	NO	ATP synthase subunit O, mitochondrial [Camponotus floridanus]
Unigene6993	NO	V-type proton ATPase subunit d [Danaus plexippus]
Unigene7100	NO	cytochrome c oxidase polypeptide IV [Bombyx mori]
Unigene720	NO	PREDICTED: similar to H+ transporting ATP synthase subunit e [Tribolium castaneum]
Unigene731	NO	hypothetical protein SINV_08923 [Solenopsis invicta]
Unigene7341	NO	vacuolar ATPase, subunit M9.7 [Manduca sexta]
Unigene7392	NO	vacuolar ATP synthase subunit H [Bombyx mori]
Unigene743	NO	GF11012 [Drosophila ananassae]
Unigene7469	NO	NADH-ubiquinone oxidoreductase 75 kDa subunit, mitochondrial precursor [Nasonia vitripennis]
Unigene763	NO	hypothetical protein SINV_15147 [Solenopsis invicta]
Unigene787	NO	NADH dehydrogenase [ubiquinone] 1 alpha subcomplex subunit 6 [Nasonia vitripennis]
Unigene7938	NO	hypothetical protein KGM_04538 [Danaus plexippus]
Unigene799	NO	hypothetical protein SINV_06969 [Solenopsis invicta]
Unigene8069	NO	GK19648 [Drosophila willistoni]
Unigene8104	NO	NADH dehydrogenase [Danaus plexippus]
Unigene8196	NO	cytochrome c oxidase subunit III [Cotesia vestalis]
Unigene8222	NO	ubiquinol-cytochrome C reductase complex 14kD subunit [Danaus plexippus]
Unigene829	NO	ATP synthase subunit d, mitochondrial [Camponotus floridanus]
Unigene8339	NO	PREDICTED: hypothetical protein LOC100743562 isoform 2 [Bombus impatiens]
Unigene837	NO	hypothetical protein AND_29231 [Anopheles darlingi]
Unigene8416	NO	H+ transporting ATP synthase O subunit isoform 1 [Bombyx mori]
Unigene8674	NO	hypothetical protein AND_14091 [Anopheles darlingi]
Unigene8922	NO	mitochondrial ATP synthase coupling factor [Antheraea yamamai]
Unigene912	NO	cytochrome c1, heme protein, mitochondrial [Nasonia vitripennis]
Unigene9141	NO	PREDICTED: hypothetical protein LOC100743562 isoform 1 [Bombus impatiens]
Unigene9462	NO	NADH dehydrogenase ubiquinone Fe-S 8 [Bombyx mori]

	Unigene9485	NO	H+ transporting ATP synthase O subunit isoform 1 [Danaus plexippus]
	Unigene952	NO	PREDICTED: acyl carrier protein, mitochondrial-like [Megachile rotundata]
	Unigene959	NO	NADH dehydrogenase [ubiquinone] 1 beta subcomplex subunit 5, mitochondrial [Nasonia vitripennis]
	Unigene9646	NO	NADH dehydrogenase subunit 1 [Cotesia vestalis]
	Unigene9901	NO	PREDICTED: cytochrome c oxidase assembly protein COX15 homolog [Apis florea]
	Unigene991	NO	PREDICTED: NADH dehydrogenase [ubiquinone] 1 alpha subcomplex subunit 7-like [Megachile rotundata]
	Unigene9927	NO	Subunit VIb of cytochrome c oxidase [Danaus plexippus]
	Unigene9971	NO	hypothetical protein KGM_15724 [Danaus plexippus]
	Unigene9980	NO	vacuolar ATP synthase catalytic subunit A [Bombyx mori]
	CL124.Contig1	NO	succinate dehydrogenase cytochrome b560 subunit, mitochondrial [Nasonia vitripennis]
**sulfur metabolism**	Unigene10886	NO	PREDICTED: bifunctional 3'-phosphoadenosine 5'-phosphosulfate synthase-like [Nasonia vitripennis]
	Unigene12614	NO	myo inositol monophosphatase [Aedes aegypti]
	Unigene3423	NO	PREDICTED: bifunctional 3'-phosphoadenosine 5'-phosphosulfate synthase-like [Apis florea]
	Unigene3560	NO	Bifunctional 3'-phosphoadenosine 5'-phosphosulfate synthetase [Acromyrmex echinatior]
	Unigene4328	NO	PREDICTED: 3'(2'),5'-bisphosphate nucleotidase 1-like isoform 1 [Nasonia vitripennis]
	Unigene5848	NO	PREDICTED: 3'(2'),5'-bisphosphate nucleotidase 1-like isoform 1 [Nasonia vitripennis]
	Unigene8565	NO	PREDICTED: bifunctional 3'-phosphoadenosine 5'-phosphosulfate synthase-like [Nasonia vitripennis]
	Unigene3562	SP	Synaptic glycoprotein SC2 [Camponotus floridanus]
	CL677.Contig2	NO	GH14704 [Drosophila grimshawi]
	CL760.Contig1	NO	PREDICTED: retinal dehydrogenase 1-like [Megachile rotundata]
	CL760.Contig2	NO	aldehyde dehydrogenase isoform A [Lysiphlebus testaceipes]
	Unigene10665	NO	Carnitine O-palmitoyltransferase 2, mitochondrial [Harpegnathos saltator]
	Unigene10800	NO	Probable peroxisomal acyl-coenzyme A oxidase 1 [Harpegnathos saltator]
	Unigene10873	NO	PREDICTED: uncharacterized protein LOC100877790 [Megachile rotundata]
	Unigene11064	NO	Carnitine O-palmitoyltransferase 2, mitochondrial [Acromyrmex echinatior]
	Unigene12024	NO	PREDICTED: enoyl-CoA hydratase domain-containing protein 3, mitochondrial-like [Nasonia vitripennis]
	Unigene1256	NO	hydroxyacyl dehydrogenase subunit B/thiolase [Lysiphlebus testaceipes]
	Unigene1264	NO	PREDICTED: retinal dehydrogenase 1-like [Megachile rotundata]
	Unigene13460	NO	PREDICTED: peroxisomal acyl-coenzyme A oxidase 3-like isoform 2 [Nasonia vitripennis]
	Unigene1586	NO	PREDICTED: probable medium-chain specific acyl-CoA dehydrogenase, mitochondrial-like [Nasonia vitripennis]
	Unigene1685	NO	PREDICTED: trifunctional enzyme subunit alpha, mitochondrial-like [Nasonia vitripennis]
	Unigene1963	NO	PREDICTED: alcohol dehydrogenase class-3-like [Megachile rotundata]
	Unigene2085	NO	PREDICTED: hypothetical protein LOC100637207 [Amphimedon queenslandica]
	Unigene2194	NO	Long-chain-fatty-acid--CoA ligase 3 [Camponotus floridanus]
	Unigene2373	NO	Carnitine O-palmitoyltransferase I, liver isoform [Camponotus floridanus]
	Unigene2402	NO	PREDICTED: very long-chain-fatty-acid--CoA ligase bubblegum-like [Nasonia vitripennis]
	Unigene2470	NO	PREDICTED: long-chain-fatty-acid--CoA ligase 3-like [Megachile rotundata]
	Unigene2549	NO	PREDICTED: aldehyde dehydrogenase, mitochondrial-like [Nasonia vitripennis]
	Unigene2684	NO	PREDICTED: enoyl-CoA delta isomerase 1, mitochondrial-like [Megachile rotundata]
	Unigene2781	NO	Acyl-CoA-binding domain-containing protein 4 [Harpegnathos saltator]
	Unigene3043	NO	Acetyl-CoA acetyltransferase, mitochondrial [Camponotus floridanus]
	Unigene3141	NO	Long-chain-fatty-acid--CoA ligase ACSBG2 [Camponotus floridanus]
	Unigene3468	NO	PREDICTED: aldehyde dehydrogenase, dimeric NADP-preferring-like isoform 2 [Nasonia vitripennis]
	Unigene3795	NO	PREDICTED: probable enoyl-CoA hydratase, mitochondrial [Apis mellifera]
	Unigene3869	NO	mitochondrial aldehyde dehydrogenase [Danaus plexippus]
	Unigene4602	NO	PREDICTED: 3-ketoacyl-CoA thiolase B, peroxisomal-like [Anolis carolinensis]
	Unigene4698	NO	Acetyl-CoA acetyltransferase, mitochondrial [Camponotus floridanus]
	Unigene5952	NO	PREDICTED: acetyl-CoA acetyltransferase, cytosolic-like [Nasonia vitripennis]
	Unigene6215	NO	4-coumarate--CoA ligase 2 [Harpegnathos saltator]
	Unigene6226	NO	Carnitine O-palmitoyltransferase I, liver isoform [Harpegnathos saltator]
	Unigene6441	NO	unnamed protein product [Homo sapiens]

**Fatty acid biosynthesis and metabolism**	Unigene6746	NO	PREDICTED: peroxisomal acyl-coenzyme A oxidase 3-like isoform 2 [Nasonia vitripennis]
	Unigene693	NO	PREDICTED: uncharacterized protein LOC100879794 [Megachile rotundata]
	Unigene7310	NO	Probable peroxisomal acyl-coenzyme A oxidase 1 [Camponotus floridanus]
	Unigene736	NO	3-ketoacyl-CoA thiolase, mitochondrial [Camponotus floridanus]
	Unigene7667	NO	short-chain acyl-CoA dehydrogenase [Nasonia vitripennis]
	Unigene7687	NO	Acetyl-CoA acetyltransferase, cytosolic [Acromyrmex echinatior]
	Unigene8037	NO	PREDICTED: aldehyde dehydrogenase X, mitochondrial-like [Nasonia vitripennis]
	Unigene8132	NO	PREDICTED: glutaryl-CoA dehydrogenase, mitochondrial-like [Bombus impatiens]
	Unigene8368	NO	aldehyde dehydroxygenase [Heliothis virescens]
	Unigene8378	NO	PREDICTED: probable peroxisomal acyl-coenzyme A oxidase 1-like [Nasonia vitripennis]
	Unigene8811	NO	Probable peroxisomal acyl-coenzyme A oxidase 1 [Harpegnathos saltator]
	Unigene8920	NO	PREDICTED: acyl-CoA synthetase family member 3, mitochondrial-like isoform 1 [Nasonia vitripennis]
	Unigene8936	NO	PREDICTED: retinal dehydrogenase 1-like isoform 1 [Apis mellifera]
	Unigene9043	NO	PREDICTED: peroxisomal acyl-coenzyme A oxidase 3-like isoform 2 [Nasonia vitripennis]
	Unigene9463	NO	Short-chain specific acyl-CoA dehydrogenase, mitochondrial [Acromyrmex echinatior]
	Unigene9523	NO	hypothetical protein SINV_80107 [Solenopsis invicta]
	Unigene968	NO	PREDICTED: very long-chain specific acyl-CoA dehydrogenase, mitochondrial-like [Nasonia vitripennis]
	Unigene9902	NO	PREDICTED: hypothetical protein LOC411299 [Apis mellifera]
	CL677.Contig1	NO	GK23895 [Drosophila willistoni]
	CL677.Contig2	NO	GH14704 [Drosophila grimshawi]
	Unigene1038	NO	PREDICTED: cytochrome b5-like isoform 1 [Nasonia vitripennis]
	Unigene10800	NO	Probable peroxisomal acyl-coenzyme A oxidase 1 [Harpegnathos saltator]
	Unigene1192	NO	hypothetical protein SINV_09878 [Solenopsis invicta]
	Unigene13460	NO	PREDICTED: peroxisomal acyl-coenzyme A oxidase 3-like isoform 2 [Nasonia vitripennis]
	Unigene1586	NO	PREDICTED: probable medium-chain specific acyl-CoA dehydrogenase, mitochondrial-like [Nasonia vitripennis]
	Unigene1685	NO	PREDICTED: trifunctional enzyme subunit alpha, mitochondrial-like [Nasonia vitripennis]
	Unigene2453	NO	PREDICTED: trans-2,3-enoyl-CoA reductase-like [Nasonia vitripennis]
	Unigene4010	NO	Acyl-CoA Delta(11) desaturase [Harpegnathos saltator]
	Unigene4602	NO	PREDICTED: 3-ketoacyl-CoA thiolase B, peroxisomal-like [Anolis carolinensis]
	Unigene4813	NO	PREDICTED: cytochrome b5 type B-like [Nasonia vitripennis]
	Unigene5018	NO	PREDICTED: elongation of very long chain fatty acids protein 6-like [Bombus terrestris]
	Unigene5611	NO	PREDICTED: estradiol 17-beta-dehydrogenase 2-like [Nasonia vitripennis]
	Unigene5990	NO	Acyl-CoA Delta(11) desaturase [Acromyrmex echinatior]
	Unigene6746	NO	PREDICTED: peroxisomal acyl-coenzyme A oxidase 3-like isoform 2 [Nasonia vitripennis]
	Unigene7310	NO	Probable peroxisomal acyl-coenzyme A oxidase 1 [Camponotus floridanus]
	Unigene8378	NO	PREDICTED: probable peroxisomal acyl-coenzyme A oxidase 1-like [Nasonia vitripennis]
	Unigene8811	NO	Probable peroxisomal acyl-coenzyme A oxidase 1 [Harpegnathos saltator]
	Unigene9043	NO	PREDICTED: peroxisomal acyl-coenzyme A oxidase 3-like isoform 2 [Nasonia vitripennis]
	Unigene10144	NO	fatty acid synthase [Bombus lucorum]
	Unigene10605	NO	PREDICTED: fatty acid synthase-like isoform 1 [Nasonia vitripennis]
	Unigene11085	NO	Acetyl-CoA carboxylase [Harpegnathos saltator]
	Unigene11539	NO	PREDICTED: fatty acid synthase-like isoform 1 [Nasonia vitripennis]
	Unigene11782	NO	PREDICTED: fatty acid synthase-like isoform 1 [Nasonia vitripennis]
	Unigene12285	NO	PREDICTED: LOW QUALITY PROTEIN: acetyl-CoA carboxylase-like [Apis florea]
	Unigene12286	NO	Acetyl-CoA carboxylase [Acromyrmex echinatior]
	Unigene12329	NO	PREDICTED: similar to fatty acid synthase [Tribolium castaneum]
	Unigene12545	NO	PREDICTED: acetyl-CoA carboxylase-like [Bombus impatiens]
	Unigene12596	NO	PREDICTED: fatty acid synthase-like isoform 1 [Nasonia vitripennis]
	Unigene13723	NO	Acetyl-CoA carboxylase [Acromyrmex echinatior]
	Unigene5752	NO	PREDICTED: fatty acid synthase-like isoform 1 [Nasonia vitripennis]
	Unigene5869	NO	PREDICTED: fatty acid synthase-like isoform 1 [Nasonia vitripennis]
	Unigene7652	NO	PREDICTED: fatty acid synthase-like isoform 1 [Nasonia vitripennis]
	Unigene7700	NO	PREDICTED: acetyl-CoA carboxylase-like isoform 2 [Bombus terrestris]
	Unigene8537	NO	PREDICTED: fatty acid synthase-like [Apis mellifera]

	CL677.Contig1	NO	GK23895 [Drosophila willistoni]
**Fatty acid elongation**	Unigene3562	SP	Synaptic glycoprotein SC2 [Camponotus floridanus]
	Unigene12024	NO	PREDICTED: enoyl-CoA hydratase domain-containing protein 3, mitochondrial-like [Nasonia vitripennis]
	Unigene1256	NO	hydroxyacyl dehydrogenase subunit B/thiolase [Lysiphlebus testaceipes]
	Unigene1685	NO	PREDICTED: trifunctional enzyme subunit alpha, mitochondrial-like [Nasonia vitripennis]
	Unigene2085	NO	PREDICTED: hypothetical protein LOC100637207 [Amphimedon queenslandica]
	Unigene2453	NO	PREDICTED: trans-2,3-enoyl-CoA reductase-like [Nasonia vitripennis]
	Unigene3795	NO	PREDICTED: probable enoyl-CoA hydratase, mitochondrial [Apis mellifera]
	Unigene5018	NO	PREDICTED: elongation of very long chain fatty acids protein 6-like [Bombus terrestris]
	Unigene5350	NO	putative trans-2-enoyl-CoA reductase [Glyptapanteles flavicoxis]
	Unigene5611	NO	PREDICTED: estradiol 17-beta-dehydrogenase 2-like [Nasonia vitripennis]
	Unigene6933	NO	PREDICTED: elongation of very long chain fatty acids protein AAEL008004-like [Nasonia vitripennis]
	Unigene736	NO	3-ketoacyl-CoA thiolase, mitochondrial [Camponotus floridanus]
	Unigene7560	NO	putative trans-2-enoyl-CoA reductase [Glyptapanteles flavicoxis]
	Unigene8053	NO	Trans-2-enoyl-CoA reductase, putative [Glyptapanteles indiensis]
	Unigene10922	NO	PREDICTED: elongation of very long chain fatty acids protein AAEL008004-like [Apis florea]
	CL58.Contig1	SP	1-acylglycerophosphocholine O-acyltransferase 1 [Harpegnathos saltator]
	CL58.Contig2	SP	PREDICTED: 1-acylglycerophosphocholine O-acyltransferase 1-like [Apis mellifera]
	CL58.Contig4	SP	PREDICTED: 1-acylglycerophosphocholine O-acyltransferase 1-like [Megachile rotundata]
	Unigene2236	SP	PREDICTED: esterase FE4 [Nasonia vitripennis]
	Unigene3097	SP	PREDICTED: lipase member H-A-like [Apis mellifera]
	Unigene3237	SP	Juvenile hormone esterase [Camponotus floridanus]
	Unigene4301	SP	PREDICTED: hypothetical protein LOC100746784 [Bombus impatiens]
	Unigene5326	SP	PREDICTED: esterase FE4-like [Apis mellifera]
	Unigene8624	SP	PREDICTED: esterase FE4-like isoform 2 [Bombus terrestris]
	Unigene9340	SP	PREDICTED: venom carboxylesterase-6-like [Apis florea]
	Unigene965	SP	PREDICTED: 1-acylglycerophosphocholine O-acyltransferase 1-like [Bombus terrestris]
	CL164.Contig1	NO	PREDICTED: bifunctional ATP-dependent dihydroxyacetone kinase/FAD-AMP lyase (cyclizing)-like [Nasonia vitripennis]
	CL164.Contig2	NO	hypothetical protein SINV_80149 [Solenopsis invicta]
	CL164.Contig3	NO	Dihydroxyacetone kinase [Camponotus floridanus]
	CL362.Contig1	NO	neutral lipase [Danaus plexippus]
	CL760.Contig1	NO	PREDICTED: retinal dehydrogenase 1-like [Megachile rotundata]
	CL760.Contig2	NO	aldehyde dehydrogenase isoform A [Lysiphlebus testaceipes]
	CL793.Contig1	NO	neutral lipase [Danaus plexippus]
	Unigene10217	NO	PREDICTED: LOW QUALITY PROTEIN: aldose reductase-like [Apis florea]
	Unigene10280	NO	PREDICTED: phosphatidate phosphatase LPIN1-like [Megachile rotundata]
	Unigene10324	NO	PREDICTED: esterase FE4-like isoform 2 [Bombus terrestris]
	Unigene10347	NO	PREDICTED: diacylglycerol O-acyltransferase 1-like [Megachile rotundata]
	Unigene10483	NO	Diacylglycerol kinase delta [Camponotus floridanus]
	Unigene10644	NO	1-acyl-sn-glycerol-3-phosphate acyltransferase gamma [Harpegnathos saltator]
	Unigene10761	NO	Neurabin-1 [Harpegnathos saltator]
	Unigene10797	NO	GJ20221 [Drosophila virilis]
	Unigene10948	NO	Phosphatidic acid phosphatase type 2 domain-containing protein 1A [Harpegnathos saltator]
	Unigene11376	NO	PREDICTED: uncharacterized protein LOC100881659 [Megachile rotundata]
	Unigene11512	NO	PREDICTED: LOW QUALITY PROTEIN: diacylglycerol kinase eta-like [Megachile rotundata]
	Unigene12028	NO	PREDICTED: membrane-bound O-acyltransferase domain-containing protein 2-like [Bombus terrestris]
	Unigene12036	NO	aldo-keto reductase, partial [Schistocerca gregaria]
	Unigene12136	NO	PREDICTED: phosphatidate phosphatase LPIN2-like [Apis florea]
	Unigene12156	NO	Glycerate kinase [Camponotus floridanus]
	Unigene1264	NO	PREDICTED: retinal dehydrogenase 1-like [Megachile rotundata]
	Unigene13018	NO	PREDICTED: aldose reductase-like [Bombus terrestris]
	Unigene13064	NO	PREDICTED: diacylglycerol kinase eta-like [Nasonia vitripennis]
	Unigene13323	NO	PREDICTED: hypothetical protein LOC100115448 [Nasonia vitripennis]
	Unigene13371	NO	conserved hypothetical protein [Culex quinquefasciatus]
	Unigene2539	NO	PREDICTED: putative phosphatidate phosphatase-like [Nasonia vitripennis]

Metabolism of other lipids	Unigene2549	NO	PREDICTED: aldehyde dehydrogenase, mitochondrial-like [Nasonia vitripennis]
	Unigene2695	NO	aldose reductase, putative [Pediculus humanus corporis]
	Unigene2728	NO	PREDICTED: putative phosphatidate phosphatase-like [Nasonia vitripennis]
	Unigene3008	NO	aldo-keto reductase-like [Nasonia vitripennis]
	Unigene3056	NO	Lipase 3 [Harpegnathos saltator]
	Unigene3223	NO	PREDICTED: glycerol-3-phosphate acyltransferase 4-like isoform 1 [Apis florea]
	Unigene3430	NO	Pancreatic triacylglycerol lipase [Acromyrmex echinatior]
	Unigene3468	NO	PREDICTED: aldehyde dehydrogenase, dimeric NADP-preferring-like isoform 2 [Nasonia vitripennis]
	Unigene3869	NO	mitochondrial aldehyde dehydrogenase [Danaus plexippus]
	Unigene4152	NO	Glycerol kinase [Harpegnathos saltator]
	Unigene4272	NO	PREDICTED: diacylglycerol kinase eta-like [Bombus impatiens]
	Unigene472	NO	PREDICTED: putative glycerol kinase 3-like isoform 1 [Bombus impatiens]
	Unigene4887	NO	PREDICTED: glycerol-3-phosphate acyltransferase 3-like isoform 3 [Nasonia vitripennis]
	Unigene5602	NO	PREDICTED: acylglycerol kinase, mitochondrial-like [Nasonia vitripennis]
	Unigene5969	NO	PREDICTED: pancreatic triacylglycerol lipase-like [Megachile rotundata]
	Unigene6591	NO	PREDICTED: aldose reductase-like [Bombus impatiens]
	Unigene6727	NO	PREDICTED: phosphatidate phosphatase PPAPDC1A-like [Bombus impatiens]
	Unigene7058	NO	PREDICTED: diacylglycerol O-acyltransferase 1 [Apis mellifera]
	Unigene7072	NO	PREDICTED: 1-acyl-sn-glycerol-3-phosphate acyltransferase alpha-like [Megachile rotundata]
	Unigene7339	NO	PREDICTED: diacylglycerol O-acyltransferase 1-like isoform 1 [Nasonia vitripennis]
	Unigene7570	NO	PREDICTED: pancreatic triacylglycerol lipase-like [Bombus impatiens]
	Unigene7874	NO	carboxylesterase clade A, member 4 [Nasonia vitripennis]
	Unigene7883	NO	PREDICTED: aldo-keto reductase family 4 member C9-like [Megachile rotundata]
	Unigene8037	NO	PREDICTED: aldehyde dehydrogenase X, mitochondrial-like [Nasonia vitripennis]
	Unigene8046	NO	1-acyl-sn-glycerol-3-phosphate acyltransferase alpha [Acromyrmex echinatior]
	Unigene8199	NO	PREDICTED: diacylglycerol kinase eta-like [Bombus impatiens]
	Unigene8368	NO	aldehyde dehydroxygenase [Heliothis virescens]
	Unigene8452	NO	PREDICTED: LOW QUALITY PROTEIN: diacylglycerol kinase eta-like [Apis florea]
	Unigene8587	NO	Aldose reductase [Acromyrmex echinatior]
	Unigene8936	NO	PREDICTED: retinal dehydrogenase 1-like isoform 1 [Apis mellifera]
	Unigene8955	NO	PREDICTED: diacylglycerol kinase eta-like [Nasonia vitripennis]
	Unigene8977	NO	PREDICTED: glycerol-3-phosphate acyltransferase 1, mitochondrial-like [Megachile rotundata]
	Unigene9356	NO	pancreatic lipase 3 [Mamestra configurata]
	Unigene9515	NO	Pancreatic triacylglycerol lipase [Acromyrmex echinatior]
	Unigene9523	NO	hypothetical protein SINV_80107 [Solenopsis invicta]
	Unigene9867	NO	Pancreatic triacylglycerol lipase [Harpegnathos saltator]
	Unigene9995	NO	Lipin-2 [Harpegnathos saltator]
	CL58.Contig3	NO	1-acylglycerophosphocholine O-acyltransferase 1 [Harpegnathos saltator]
	Unigene100	NO	Diacylglycerol cholinephosphotransferase, putative [Pediculus humanus corporis]
	Unigene10121	NO	85 kDa calcium-independent phospholipase A2 [Camponotus floridanus]
	Unigene10184	NO	hypothetical protein DAPPUDRAFT_304184 [Daphnia pulex]
	Unigene10507	NO	Lissencephaly-1-like protein [Acromyrmex echinatior]
	Unigene10531	NO	phospholipase A2 precursor [Apis mellifera]
	Unigene10698	NO	WD repeat-containing protein 37 [Harpegnathos saltator]
	Unigene10948	NO	Phosphatidic acid phosphatase type 2 domain-containing protein 1A [Harpegnathos saltator]
	Unigene11598	NO	PREDICTED: group XIIA secretory phospholipase A2-like [Apis florea]
	Unigene11613	NO	PREDICTED: calcium-independent phospholipase A2-gamma-like [Bombus terrestris]
	Unigene12641	NO	PREDICTED: LOW QUALITY PROTEIN: phospholipase D1-like [Bombus terrestris]
	Unigene12870	NO	PREDICTED: LOW QUALITY PROTEIN: ethanolaminephosphotransferase 1-like [Nasonia vitripennis]
	Unigene13229	NO	PREDICTED: phospholipase D1-like isoform 1 [Megachile rotundata]
	Unigene13259	NO	PREDICTED: phospholipase D2-like [Apis florea]
	Unigene13599	NO	hypothetical protein SINV_08811 [Solenopsis invicta]
	Unigene1927	NO	PREDICTED: SH3 domain-binding glutamic acid-rich protein homolog [Megachile rotundata]
	Unigene2017	NO	hypothetical protein AaeL_AAEL006826 [Aedes aegypti]
	Unigene2539	NO	PREDICTED: putative phosphatidate phosphatase-like [Nasonia vitripennis]
	Unigene2728	NO	PREDICTED: putative phosphatidate phosphatase-like [Nasonia vitripennis]
	Unigene3125	NO	Striatin-3 [Harpegnathos saltator]
	Unigene3861	NO	PREDICTED: group XIIA secretory phospholipase A2-like [Nasonia vitripennis]
	Unigene4226	NO	PREDICTED: choline/ethanolaminephosphotransferase 1-like [Apis florea]

	Unigene6351	NO	PREDICTED: lissencephaly-1 homolog [Megachile rotundata]
	Unigene6727	NO	PREDICTED: phosphatidate phosphatase PPAPDC1A-like [Bombus impatiens]
	Unigene6951	NO	Lissencephaly-1-like protein [Harpegnathos saltator]
	Unigene8599	NO	Ethanolaminephosphotransferase 1 [Camponotus floridanus]
	Unigene8639	NO	PREDICTED: probable cytosolic iron-sulfur protein assembly protein Ciao1-like [Apis florea]
	Unigene8759	NO	Phospholipase D1 [Harpegnathos saltator]
	Unigene9461	NO	PREDICTED: LOW QUALITY PROTEIN: phospholipase D1-like [Bombus terrestris]
**Developmental regulation**			
	**Unigene ID**	**ignal peptide**	**Homologs**
	CL430.Contig2	SP	PREDICTED: tenascin-X-like [Nasonia vitripennis]
	Unigene12612	SP	Armadillo segment polarity protein [Harpegnathos saltator]
	Unigene191	SP	PREDICTED: similar to Prg4 protein [Taeniopygia guttata]
	Unigene758	SP	Protein toll [Harpegnathos saltator]
	Unigene96	SP	Vascular endothelial growth factor receptor 2 [Harpegnathos saltator]
	CL22.Contig2	NO	hypothetical protein NEMVEDRAFT_v1g222522 [Nematostella vectensis]
	CL238.Contig1	NO	PREDICTED: protein kinase shaggy-like isoform 2 [Nasonia vitripennis]
	CL238.Contig2	NO	PREDICTED: protein kinase shaggy-like isoform 2 [Nasonia vitripennis]
	CL238.Contig4	NO	Protein kinase shaggy [Acromyrmex echinatior]
	CL239.Contig1	NO	PREDICTED: uncharacterized protein LOC100878829 [Megachile rotundata]
	CL363.Contig1	NO	PREDICTED: serine/threonine-protein phosphatase 2B catalytic subunit 2-like [Apis florea]
	CL363.Contig2	NO	PREDICTED: serine/threonine-protein phosphatase 2B catalytic subunit 2-like [Apis florea]
	CL375.Contig1	NO	guanine nucleotide-binding protein subunit beta-like [Microplitis mediator]
	CL375.Contig2	NO	guanine nucleotide-binding protein subunit beta-like [Microplitis mediator]
	CL412.Contig1	NO	hypothetical protein SINV_01823 [Solenopsis invicta]
	CL515.Contig1	NO	hypothetical protein SINV_06010 [Solenopsis invicta]
	CL515.Contig2	NO	hypothetical protein SINV_06010 [Solenopsis invicta]
	CL591.Contig1	NO	PREDICTED: E3 ubiquitin-protein ligase IAP-3-like [Bombus impatiens]
	CL591.Contig2	NO	PREDICTED: E3 ubiquitin-protein ligase IAP-3-like [Megachile rotundata]
	CL735.Contig2	NO	G protein-coupled receptor kinase 1 [Camponotus floridanus]
	CL757.Contig2	NO	PREDICTED: uncharacterized protein LOC100876927 [Megachile rotundata]
	Unigene10083	NO	putative armadillo protein [Danaus plexippus]
	Unigene10121	NO	85 kDa calcium-independent phospholipase A2 [Camponotus floridanus]
	Unigene10138	NO	PREDICTED: uncharacterized protein LOC100881293 [Megachile rotundata]
	Unigene10184	NO	hypothetical protein DAPPUDRAFT_304184 [Daphnia pulex]
	Unigene10223	NO	PREDICTED: ribosomal protein S6 kinase beta-1-like [Apis mellifera]
	Unigene10410	NO	Mothers against decapentaplegic-like protein 3 [Acromyrmex echinatior]
	Unigene10531	NO	phospholipase A2 precursor [Apis mellifera]
	Unigene10610	NO	PREDICTED: hypothetical protein LOC100114432 [Nasonia vitripennis]
	Unigene10828	NO	PREDICTED: calcium/calmodulin-dependent protein kinase type II alpha chain-like isoform 3 [Bombus terrestris]
	Unigene1089	NO	Interferon regulatory factor 2-binding protein 2-A [Harpegnathos saltator]
	Unigene10956	NO	PREDICTED: LOW QUALITY PROTEIN: RING finger and SPRY domain-containing protein 1-like [Bombus terrestris]
	Unigene11062	NO	Stress-activated protein kinase JNK [Acromyrmex echinatior]
	Unigene11087	NO	PREDICTED: guanine nucleotide-binding protein G(i) subunit alpha-like [Megachile rotundata]
	Unigene11124	NO	Presenilin-like protein [Harpegnathos saltator]
	Unigene11200	NO	PREDICTED: rho-associated protein kinase 2-like [Bombus impatiens]
	Unigene11213	NO	Phosphatidylinositol 3-kinase regulatory subunit alpha [Camponotus floridanus]
	Unigene11447	NO	hypothetical protein SINV_14862 [Solenopsis invicta]
	Unigene11598	NO	PREDICTED: group XIIA secretory phospholipase A2-like [Apis florea]
	Unigene11613	NO	PREDICTED: calcium-independent phospholipase A2-gamma-like [Bombus terrestris]
	Unigene11645	NO	putative ETS-like protein pointed, isoform P1 [Danaus plexippus]
	Unigene11659	NO	GTP-binding protein (i) alpha subunit, gnai [Aedes aegypti]
	Unigene11690	NO	PREDICTED: protein son of sevenless-like [Megachile rotundata]
	Unigene11985	NO	PREDICTED: hypothetical protein LOC408354 [Apis mellifera]
	Unigene1213	NO	PREDICTED: calcineurin subunit B type 2-like isoform 1 [Nasonia vitripennis]
	Unigene12247	NO	PREDICTED: hypothetical protein LOC100743521 [Bombus impatiens]
	Unigene12735	NO	PREDICTED: armadillo segment polarity protein isoform 2 [Nasonia vitripennis]
	Unigene1310	NO	PREDICTED: troponin C, isoform 3 isoform 1 [Nasonia vitripennis]
	Unigene13553	NO	PREDICTED: protein BCL9 homolog [Apis florea]
	Unigene1513	NO	conserved hypothetical protein [Pediculus humanus corporis]

Cell survival	Unigene1580	NO	hypothetical protein EAI_14262 [Harpegnathos saltator]
	Unigene1630	NO	small heat shock protein [Pteromalus puparum]
	Unigene1894	NO	Ras-like GTP-binding protein Rho1 [Salmo salar]
	Unigene1925	NO	PREDICTED: cdc42 homolog isoform 1 [Nasonia vitripennis]
	Unigene1927	NO	PREDICTED: SH3 domain-binding glutamic acid-rich protein homolog [Megachile rotundata]
	Unigene2017	NO	hypothetical protein AaeL_AAEL006826 [Aedes aegypti]
	Unigene2069	NO	PREDICTED: ras-like protein 1-like [Apis florea]
	Unigene2152	NO	Guanine nucleotide-binding protein subunit gamma-1 [Camponotus floridanus]
	Unigene2158	NO	PREDICTED: WD repeat-containing protein 55 homolog [Nasonia vitripennis]
	Unigene2210	NO	PREDICTED: signal transducer and activator of transcription 5B-like [Megachile rotundata]
	Unigene2339	NO	Guanine nucleotide-binding protein subunit gamma-e [Harpegnathos saltator]
	Unigene2418	NO	PREDICTED: RAC serine/threonine-protein kinase-like [Bombus impatiens]
	Unigene2504	NO	hypothetical protein TRIADDRAFT_20496 [Trichoplax adhaerens]
	Unigene2544	NO	PREDICTED: uncharacterized protein LOC100879259 [Megachile rotundata]
	Unigene2630	NO	PREDICTED: ribosomal protein S6 kinase beta-1-like [Apis florea]
	Unigene2925	NO	PREDICTED: hypothetical protein LOC409983 [Apis mellifera]
	Unigene3005	NO	PREDICTED: calumenin-like [Nasonia vitripennis]
	Unigene3146	NO	Ran-binding protein 9 [Camponotus floridanus]
	Unigene3316	NO	PREDICTED: calcium-binding protein p22-like [Megachile rotundata]
	Unigene3692	NO	PREDICTED: LOW QUALITY PROTEIN: G protein-coupled receptor kinase 1-like [Apis florea]
	Unigene383	NO	PREDICTED: cytochrome c-like isoform 1 [Bombus terrestris]
	Unigene3861	NO	PREDICTED: group XIIA secretory phospholipase A2-like [Nasonia vitripennis]
	Unigene4045	NO	Dual specificity mitogen-activated protein kinase kinase 4 [Acromyrmex echinatior]
	Unigene4101	NO	hypothetical protein SINV_05984 [Solenopsis invicta]
	Unigene4156	NO	PREDICTED: rho-associated protein kinase 2-like isoform 2 [Nasonia vitripennis]
	Unigene4353	NO	RING finger protein unkempt-like protein [Harpegnathos saltator]
	Unigene4470	NO	Ras-like GTP-binding protein RHO [Lepeophtheirus salmonis]
	Unigene452	NO	PREDICTED: activating transcription factor of chaperone-like [Megachile rotundata]
	Unigene4655	NO	PREDICTED: transcription factor E2F3-like isoform 1 [Bombus terrestris]
	Unigene4710	NO	Dual specificity mitogen-activated protein kinase kinase 4 [Acromyrmex echinatior]
	Unigene4742	NO	PREDICTED: calmodulin-like protein 4-like isoform 1 [Bombus terrestris]
	Unigene4774	NO	mitochondrial cytochrome c [Bombyx mori]
	Unigene485	NO	AT15141p [Drosophila melanogaster]
	Unigene4920	NO	PREDICTED: mitogen-activated protein kinase 1-like [Megachile rotundata]
	Unigene514	NO	PREDICTED: serine/threonine-protein kinase KDX1-like [Bombus impatiens]
	Unigene5515	NO	PREDICTED: armadillo segment polarity protein-like [Megachile rotundata]
	Unigene5526	NO	PREDICTED: protein toll [Nasonia vitripennis]
	Unigene5593	NO	PREDICTED: hypothetical protein LOC100745810 isoform 2 [Bombus impatiens]
	Unigene5630	NO	PREDICTED: rho-associated protein kinase 2-like isoform 2 [Nasonia vitripennis]
	Unigene5691	NO	PREDICTED: hypothetical protein LOC412916 [Apis mellifera]
	Unigene5806	NO	PREDICTED: uncharacterized protein LOC100878829 [Megachile rotundata]
	Unigene6006	NO	PREDICTED: rho-associated protein kinase 2 [Apis florea]
	Unigene6140	NO	Ras-like GTP-binding protein Rho1 [Danaus plexippus]
	Unigene6295	NO	PREDICTED: dual specificity mitogen-activated protein kinase kinase hemipterous-like isoform 2 [Megachile rotundata]
	Unigene6363	NO	Mitogen-activated protein kinase 1 [Harpegnathos saltator]
	Unigene6600	NO	PREDICTED: presenilin homolog [Bombus impatiens]
	Unigene6671	NO	Ras-like GTP-binding protein RHO [Lepeophtheirus salmonis]
	Unigene669	NO	PREDICTED: protein enhancer of sevenless 2B-like [Nasonia vitripennis]
	Unigene7027	NO	AGAP005160-PA [Anopheles gambiae str. PEST]
	Unigene7102	NO	Multiple C2 and transmembrane domain-containing protein 2 [Acromyrmex echinatior]
	Unigene722	NO	PREDICTED: ras-like GTP-binding protein Rho1 [Nasonia vitripennis]
	Unigene7225	NO	PREDICTED: hypothetical protein LOC408577 [Apis mellifera]
	Unigene7498	NO	hypothetical protein SINV_12469 [Solenopsis invicta]
	Unigene7671	NO	PREDICTED: rho-associated protein kinase 2 [Megachile rotundata]
	Unigene7714	NO	Mitogen-activated protein kinase 14B [Harpegnathos saltator]
	Unigene7754	NO	PREDICTED: cyclin-dependent kinase 6-like [Apis florea]
	Unigene776	NO	AT15141p [Drosophila melanogaster]
	Unigene7916	NO	Dual specificity mitogen-activated protein kinase kinase 7 [Harpegnathos saltator]
	Unigene7980	NO	PREDICTED: dual specificity mitogen-activated protein kinase kinase 2-like [Apis florea]
	Unigene8266	NO	PREDICTED: mothers against decapentaplegic homolog 3 [Nasonia vitripennis]

	Unigene8344	NO	PREDICTED: phosphatidylinositol 3-kinase regulatory subunit alpha-like, partial [Apis florea]
	Unigene8372	NO	PREDICTED: baculoviral IAP repeat-containing protein 3-like [Nasonia vitripennis]
	Unigene8427	NO	PREDICTED: rho-associated protein kinase 2 [Megachile rotundata]
	Unigene8488	NO	PREDICTED: uncharacterized protein LOC100877711 [Megachile rotundata]
	Unigene8515	NO	PREDICTED: hypothetical protein LOC100642907 [Bombus terrestris]
	Unigene8714	NO	PREDICTED: G protein-coupled receptor kinase 1-like [Megachile rotundata]
	Unigene8755	NO	PREDICTED: LOW QUALITY PROTEIN: ribosomal protein S6 kinase beta-1-like [Apis florea]
	Unigene8839	NO	Rho-associated protein kinase 2 [Camponotus floridanus]
	Unigene905	NO	PREDICTED: transcription factor AP-1-like [Megachile rotundata]
	Unigene9217	NO	PREDICTED: stress-activated protein kinase JNK-like [Megachile rotundata]
	Unigene9259	NO	PREDICTED: calcium/calmodulin-dependent protein kinase type II alpha chain-like isoform 1 [Bombus impatiens]
	Unigene9295	NO	PREDICTED: rho-associated protein kinase 2-like [Bombus impatiens]
	Unigene9359	NO	PREDICTED: hypothetical protein LOC100743083 [Bombus impatiens]
	Unigene9378	NO	PREDICTED: armadillo segment polarity protein-like [Megachile rotundata]
	Unigene9415	NO	PREDICTED: mitogen-activated protein kinase 14B-like isoform 2 [Megachile rotundata]
	Unigene975	NO	Growth hormone-inducible transmembrane protein [Camponotus floridanus]
	Unigene9998	NO	PREDICTED: stress-activated protein kinase JNK-like [Megachile rotundata]
	CL238.Contig1	NO	PREDICTED: protein kinase shaggy-like isoform 2 [Nasonia vitripennis]
	CL334.Contig1	SP	Calcium-transporting ATPase sarcoplasmic/endoplasmic reticulum type [Acromyrmex echinatior]
	CL430.Contig2	SP	PREDICTED: tenascin-X-like [Nasonia vitripennis]
	CL590.Contig1	SP	PREDICTED: serine/threonine-protein kinase/endoribonuclease IRE1-like [Nasonia vitripennis]
	CL690.Contig1	SP	hypothetical protein SINV_04265 [Solenopsis invicta]
	CL690.Contig2	SP	GJ24134 [Drosophila virilis]
	Unigene1011	SP	PREDICTED: membrane metallo-endopeptidase-like 1-like [Bombus impatiens]
	Unigene10722	SP	Protein VAC14-like protein [Camponotus floridanus]
	Unigene12612	SP	Armadillo segment polarity protein [Harpegnathos saltator]
	Unigene1289	SP	cytochrome oxidase subunit I [Cotesia chilonis]
	Unigene2376	SP	hypothetical protein TcasGA2_TC010888 [Tribolium castaneum]
	Unigene244	SP	calreticulin [Cotesia rubecula]
	Unigene2802	SP	PREDICTED: serine/threonine-protein kinase/endoribonuclease IRE1-like [Megachile rotundata]
	Unigene3097	SP	PREDICTED: lipase member H-A-like [Apis mellifera]
	Unigene3386	SP	Calnexin [Camponotus floridanus]
	Unigene3500	SP	PREDICTED: serine/threonine-protein kinase/endoribonuclease IRE1-like [Nasonia vitripennis]
	Unigene4476	SP	hypothetical protein SINV_04265 [Solenopsis invicta]
	Unigene4608	SP	Serine/threonine-protein kinase/endoribonuclease ire-1 [Camponotus floridanus]
	Unigene6037	SP	PREDICTED: serine/threonine-protein kinase/endoribonuclease IRE1-like [Nasonia vitripennis]
	Unigene6049	SP	hypothetical protein SINV_07475 [Solenopsis invicta]
	Unigene6782	SP	PREDICTED: calnexin-like [Megachile rotundata]
	Unigene758	SP	Protein toll [Harpegnathos saltator]
	Unigene96	SP	Vascular endothelial growth factor receptor 2 [Harpegnathos saltator]
	CL205.Contig1	NO	PREDICTED: NADH dehydrogenase [ubiquinone] flavoprotein 1, mitochondrial-like [Apis florea]
	CL205.Contig2	NO	PREDICTED: NADH dehydrogenase [ubiquinone] flavoprotein 1, mitochondrial [Apis mellifera]
	CL231.Contig1	NO	ATP synthase subunit alpha, mitochondrial [Harpegnathos saltator]
	CL238.Contig1	NO	PREDICTED: protein kinase shaggy-like isoform 2 [Nasonia vitripennis]
	CL238.Contig2	NO	PREDICTED: protein kinase shaggy-like isoform 2 [Nasonia vitripennis]
	CL238.Contig4	NO	Protein kinase shaggy [Acromyrmex echinatior]
	CL239.Contig1	NO	PREDICTED: uncharacterized protein LOC100878829 [Megachile rotundata]
	CL352.Contig1	NO	cytochrome c oxidase subunit I [Cotesia vestalis]
	CL352.Contig2	NO	cytochrome oxidase subunit I [Bucculatrix artemisiella]
	CL352.Contig3	NO	cytochrome c oxidase subunit I [Cotesia vestalis]
	CL363.Contig1	NO	PREDICTED: serine/threonine-protein phosphatase 2B catalytic subunit 2-like [Apis florea]
	CL363.Contig2	NO	PREDICTED: serine/threonine-protein phosphatase 2B catalytic subunit 2-like [Apis florea]
	CL374.Contig1	NO	ATP synthase subunit beta, mitochondrial [Harpegnathos saltator]

CL374.Contig2	NO	ATP-synthase subunit beta [Schistocerca gregaria]
CL374.Contig3	NO	ATP synthase subunit beta, putative [Pediculus humanus corporis]
CL375.Contig1	NO	guanine nucleotide-binding protein subunit beta-like [Microplitis mediator]
CL375.Contig2	NO	guanine nucleotide-binding protein subunit beta-like [Microplitis mediator]
CL401.Contig2	NO	PREDICTED: ATP synthase subunit epsilon, mitochondrial-like isoform 1 [Apis mellifera]
CL412.Contig1	NO	hypothetical protein SINV_01823 [Solenopsis invicta]
CL421.Contig2	NO	PREDICTED: glyceraldehyde-3-phosphate dehydrogenase 2 [Nasonia vitripennis]
CL438.Contig1	NO	PREDICTED: GTP-binding nuclear protein Ran [Apis mellifera]
CL438.Contig2	NO	GTP-binding nuclear protein ran [Danaus plexippus]
CL438.Contig3	NO	GTP-binding nuclear protein ran [Danaus plexippus]
CL466.Contig1	NO	sugar transporter, putative [Ixodes scapularis]
CL483.Contig1	NO	PREDICTED: hypothetical protein LOC100743933 [Bombus impatiens]
CL483.Contig2	NO	Microtubule-associated protein 2 [Harpegnathos saltator]
CL51.Contig1	NO	hypothetical protein SINV_04265 [Solenopsis invicta]
CL51.Contig2	NO	hypothetical protein SINV_04265 [Solenopsis invicta]
CL515.Contig1	NO	hypothetical protein SINV_06010 [Solenopsis invicta]
CL515.Contig2	NO	hypothetical protein SINV_06010 [Solenopsis invicta]
CL560.Contig1	NO	cytochrome b [Cotesia vestalis]
CL591.Contig1	NO	PREDICTED: E3 ubiquitin-protein ligase IAP-3-like [Bombus impatiens]
CL591.Contig2	NO	PREDICTED: E3 ubiquitin-protein ligase IAP-3-like [Megachile rotundata]
CL597.Contig2	NO	cytochrome b [Cotesia vestalis]
CL676.Contig1	NO	PREDICTED: g1/S-specific cyclin-D2-like [Bombus terrestris]
CL735.Contig2	NO	G protein-coupled receptor kinase 1 [Camponotus floridanus]
CL740.Contig1	NO	PREDICTED: NADH dehydrogenase [ubiquinone] flavoprotein 1, mitochondrial-like [Megachile rotundata]
CL750.Contig1	NO	PREDICTED: ATP synthase lipid-binding protein, mitochondrial-like [Megachile rotundata]
CL757.Contig2	NO	PREDICTED: uncharacterized protein LOC100876927 [Megachile rotundata]
CL792.Contig1	NO	hypothetical protein SINV_06702 [Solenopsis invicta]
Unigene10083	NO	putative armadillo protein [Danaus plexippus]
Unigene10090	NO	PREDICTED: LOW QUALITY PROTEIN: g2/mitotic-specific cyclin-B3-like [Bombus terrestris]
Unigene1012	NO	Cytochrome b-c1 complex subunit 2, mitochondrial [Camponotus floridanus]
Unigene10125	NO	hypothetical protein SINV_80464 [Solenopsis invicta]
Unigene10138	NO	PREDICTED: uncharacterized protein LOC100881293 [Megachile rotundata]
Unigene1014	NO	PREDICTED: NADH dehydrogenase [ubiquinone] iron-sulfur protein 7, mitochondrial-like [Apis florea]
Unigene10183	NO	Talin-1 [Acromyrmex echinatior]
Unigene10223	NO	PREDICTED: ribosomal protein S6 kinase beta-1-like [Apis mellifera]
Unigene10285	NO	PREDICTED: exportin-1-like [Megachile rotundata]
Unigene10363	NO	GTP-binding protein alpha subunit, gna [Culex quinquefasciatus]
Unigene10410	NO	Mothers against decapentaplegic-like protein 3 [Acromyrmex echinatior]
Unigene1043	NO	PREDICTED: cytochrome c oxidase subunit 6C-like [Acyrthosiphon pisum]
Unigene10437	NO	PREDICTED: exportin-1-like [Megachile rotundata]
Unigene1044	NO	GF11341 [Drosophila ananassae]
Unigene10512	NO	Mitotic checkpoint protein BUB3 [Acromyrmex echinatior]
Unigene10590	NO	hypothetical protein KGM_04009 [Danaus plexippus]
Unigene10610	NO	PREDICTED: hypothetical protein LOC100114432 [Nasonia vitripennis]
Unigene1074	NO	NADH dehydrogenase [ubiquinone] 1 alpha subcomplex subunit 13 [Camponotus floridanus]
Unigene10847	NO	PREDICTED: nicastrin-like [Megachile rotundata]
Unigene1089	NO	Interferon regulatory factor 2-binding protein 2-A [Harpegnathos saltator]
Unigene1090	NO	PREDICTED: similar to NADH:ubiquinone dehydrogenase, putative [Tribolium castaneum]
Unigene10956	NO	PREDICTED: LOW QUALITY PROTEIN: RING finger and SPRY domain-containing protein 1-like [Bombus terrestris]
Unigene11062	NO	Stress-activated protein kinase JNK [Acromyrmex echinatior]
Unigene11087	NO	PREDICTED: guanine nucleotide-binding protein G(i) subunit alpha-like [Megachile rotundata]
Unigene11124	NO	Presenilin-like protein [Harpegnathos saltator]
Unigene11156	NO	PREDICTED: nicastrin [Apis mellifera]
Unigene1116	NO	Ras-related nuclear protein [Mizuhopecten yessoensis]
Unigene1120	NO	PREDICTED: endothelin-converting enzyme 1-like [Nasonia vitripennis]
Unigene11200	NO	PREDICTED: rho-associated protein kinase 2-like [Bombus impatiens]
Unigene11213	NO	Phosphatidylinositol 3-kinase regulatory subunit alpha [Camponotus floridanus]

Unigene11321	NO	hypothetical protein SINV_80464 [Solenopsis invicta]
Unigene11447	NO	hypothetical protein SINV_14862 [Solenopsis invicta]
Unigene11450	NO	conserved hypothetical protein [Pediculus humanus corporis]
Unigene1149	NO	hypothetical protein DAPPUDRAFT_51702 [Daphnia pulex]
Unigene11645	NO	putative ETS-like protein pointed, isoform P1 [Danaus plexippus]
Unigene11659	NO	GTP-binding protein (i) alpha subunit, gnai [Aedes aegypti]
Unigene11690	NO	PREDICTED: protein son of sevenless-like [Megachile rotundata]
Unigene11731	NO	GE14316 [Drosophila yakuba]
Unigene11735	NO	PREDICTED: eukaryotic translation initiation factor 2-alpha kinase-like [Bombus terrestris]
Unigene11808	NO	cytochrome c oxidase subunit II [Cotesia vestalis]
Unigene11921	NO	PREDICTED: TATA-box-binding protein-like [Bombus terrestris]
Unigene11930	NO	PREDICTED: cell division cycle protein 16 homolog [Megachile rotundata]
Unigene11985	NO	PREDICTED: hypothetical protein LOC408354 [Apis mellifera]
Unigene12096	NO	PREDICTED: protein VAC14 homolog [Megachile rotundata]
Unigene1213	NO	PREDICTED: calcineurin subunit B type 2-like isoform 1 [Nasonia vitripennis]
Unigene12162	NO	PREDICTED: disintegrin and metalloproteinase domain-containing protein 10-like [Megachile rotundata]
Unigene12247	NO	PREDICTED: hypothetical protein LOC100743521 [Bombus impatiens]
Unigene12335	NO	PREDICTED: insulin-degrading enzyme-like isoform 2 [Nasonia vitripennis]
Unigene1243	NO	GI19985 [Drosophila mojavensis]
Unigene1263	NO	PREDICTED: microtubule-associated protein tau-like [Apis florea]
Unigene12735	NO	PREDICTED: armadillo segment polarity protein isoform 2 [Nasonia vitripennis]
Unigene12807	NO	PREDICTED: disintegrin and metalloproteinase domain-containing protein 10-like [Megachile rotundata]
Unigene1286	NO	ADP/ATP translocase [Helicoverpa armigera]
Unigene1290	NO	PREDICTED: succinate dehydrogenase [ubiquinone] flavoprotein subunit, mitochondrial-like [Megachile rotundata]
Unigene13000	NO	PREDICTED: gamma-secretase subunit pen-2-like [Megachile rotundata]
Unigene1310	NO	PREDICTED: troponin C, isoform 3 isoform 1 [Nasonia vitripennis]
Unigene13136	NO	hypothetical protein SINV_10030 [Solenopsis invicta]
Unigene13138	NO	PREDICTED: segment polarity protein dishevelled homolog DVL-3-like [Megachile rotundata]
Unigene13191	NO	PREDICTED: cyclic AMP-dependent transcription factor ATF-2-like [Apis mellifera]
Unigene1327	NO	hypothetical protein SINV_09347 [Solenopsis invicta]
Unigene13515	NO	PREDICTED: TATA-box-binding protein-like [Megachile rotundata]
Unigene13553	NO	PREDICTED: protein BCL9 homolog [Apis florea]
Unigene13656	NO	cytochrome oxidase subunit II [Xyrosaris lichneuta]
Unigene1371	NO	NADH dehydrogenase [ubiquinone] 1 beta subcomplex subunit 8, mitochondrial [Nasonia vitripennis]
Unigene1404	NO	hypothetical protein AND_29000 [Anopheles darlingi]
Unigene1437	NO	PREDICTED: translocator protein-like [Bombus impatiens]
Unigene1440	NO	NADH dehydrogenase (ubiquinone) 1 beta subcomplex, 7 [Tribolium castaneum]
Unigene1446	NO	PREDICTED: proto-oncogene c-Fos-like [Bombus terrestris]
Unigene1447	NO	NADH dehydrogenase [ubiquinone] iron-sulfur protein 6, mitochondrial [Nasonia vitripennis]
Unigene1473	NO	hypothetical protein EAI_14262 [Harpegnathos saltator]
Unigene1513	NO	conserved hypothetical protein [Pediculus humanus corporis]
Unigene1546	NO	PREDICTED: hypothetical protein LOC100119331 [Nasonia vitripennis]
Unigene1574	NO	AGAP009602-PA [Anopheles gambiae str. PEST]
Unigene1611	NO	PREDICTED: probable NADH dehydrogenase [ubiquinone] 1 alpha subcomplex subunit 12-like [Megachile rotundata]
Unigene1633	NO	NADH dehydrogenase [ubiquinone] iron-sulfur protein 8, mitochondrial [Nasonia vitripennis]
Unigene1646	NO	NADH dehydrogenase [Culex quinquefasciatus]
Unigene1653	NO	AGAP012374-PA [Anopheles gambiae str. PEST]
Unigene1688	NO	GD20916 [Drosophila simulans]
Unigene1736	NO	AGAP009824-PA [Anopheles gambiae str. PEST]
Unigene1738	NO	succinate dehydrogenase iron sulfur subunit B [Lysiphlebus testaceipes]
Unigene1749	NO	NADH dehydrogenase [ubiquinone] 1 beta subcomplex subunit 2, mitochondrial [Camponotus floridanus]
Unigene1756	NO	3-hydroxyacyl-CoA dehydrogenase type-2 [Camponotus floridanus]
Unigene1800	NO	NADH dehydrogenase (ubiquinone) 1 alpha subcomplex, 9, 39kDa [Nasonia vitripennis]
Unigene1833	NO	NADH dehydrogenase [ubiquinone] 1 alpha subcomplex subunit 2 [Camponotus floridanus]

	Unigene1894	NO	Ras-like GTP-binding protein Rho1 [Salmo salar]
	Unigene1925	NO	PREDICTED: cdc42 homolog isoform 1 [Nasonia vitripennis]
	Unigene2059	NO	budding uninhibited by benzimidazoles 3 [Tribolium castaneum]
	Unigene2069	NO	PREDICTED: ras-like protein 1-like [Apis florea]
	Unigene2076	NO	--
	Unigene2152	NO	Guanine nucleotide-binding protein subunit gamma-1 [Camponotus floridanus]
	Unigene2158	NO	PREDICTED: WD repeat-containing protein 55 homolog [Nasonia vitripennis]
	Unigene2196	NO	PREDICTED: serine/threonine-protein kinase grp isoform 2 [Apis mellifera]
	Unigene2210	NO	PREDICTED: signal transducer and activator of transcription 5B-like [Megachile rotundata]
	Unigene2224	NO	NADH dehydrogenase [Lysiphlebus testaceipes]
	Unigene2244	NO	AGAP006918-PA [Anopheles gambiae str. PEST]
	Unigene2339	NO	Guanine nucleotide-binding protein subunit gamma-e [Harpegnathos saltator]
	Unigene2346	NO	NADH dehydrogenase [ubiquinone] flavoprotein 2, mitochondrial [Nasonia vitripennis]
	Unigene2365	NO	PREDICTED: ran-specific GTPase-activating protein-like [Bombus impatiens]
	Unigene2418	NO	PREDICTED: RAC serine/threonine-protein kinase-like [Bombus impatiens]
	Unigene2469	NO	Cell division protein kinase 5 [Harpegnathos saltator]
	Unigene25	NO	PREDICTED: ras-like protein 2-like [Nasonia vitripennis]
	Unigene2504	NO	hypothetical protein TRIADDRAFT_20496 [Trichoplax adhaerens]
	Unigene2537	NO	ATP synthase subunit c [Manduca sexta]
	Unigene2544	NO	PREDICTED: uncharacterized protein LOC100879259 [Megachile rotundata]
	Unigene2610	NO	hypothetical protein SINV_04265 [Solenopsis invicta]
	Unigene2630	NO	PREDICTED: ribosomal protein S6 kinase beta-1-like [Apis florea]
	Unigene2697	NO	cytochrome c oxidase polypeptide Vb [Bombyx mori]
	Unigene2856	NO	succinate dehydrogenase [ubiquinone] cytochrome b small subunit, mitochondrial [Nasonia vitripennis]
	Unigene2903	NO	hypothetical protein KGM_05439 [Danaus plexippus]
	Unigene2925	NO	PREDICTED: hypothetical protein LOC409983 [Apis mellifera]
	Unigene2926	NO	PREDICTED: calcium-transporting ATPase sarcoplasmic/endoplasmic reticulum type-like [Megachile rotundata]
**Cell apoptosis**	Unigene2956	NO	hypothetical protein SINV_02345 [Solenopsis invicta]
	Unigene2992	NO	Exportin-1 [Harpegnathos saltator]
	Unigene3005	NO	PREDICTED: calumenin-like [Nasonia vitripennis]
	Unigene3137	NO	PREDICTED: ran-specific GTPase-activating protein-like [Apis mellifera]
	Unigene3146	NO	Ran-binding protein 9 [Camponotus floridanus]
	Unigene3257	NO	PREDICTED: hypothetical protein LOC100122162 [Nasonia vitripennis]
	Unigene3316	NO	PREDICTED: calcium-binding protein p22-like [Megachile rotundata]
	Unigene3344	NO	PREDICTED: solute carrier family 2, facilitated glucose transporter member 1-like [Nasonia vitripennis]
	Unigene3360	NO	Low-density lipoprotein receptor-related protein 1B [Camponotus floridanus]
	Unigene3376	NO	PREDICTED: hypothetical protein LOC100745000 [Bombus impatiens]
	Unigene3405	NO	ATP synthase [Bombyx mori]
	Unigene3407	NO	PREDICTED: sideroflexin-1-like isoform 2 [Nasonia vitripennis]
	Unigene346	NO	GJ13165 [Drosophila virilis]
	Unigene3519	NO	PREDICTED: uncharacterized protein LOC100867253 [Apis florea]
	Unigene368	NO	PREDICTED: ADP,ATP carrier protein 2-like [Bombus impatiens]
	Unigene3692	NO	PREDICTED: LOW QUALITY PROTEIN: G protein-coupled receptor kinase 1-like [Apis florea]
	Unigene372	NO	PREDICTED: hypothetical protein LOC100115623 isoform 1 [Nasonia vitripennis]
	Unigene3745	NO	PREDICTED: protein Dr1-like [Megachile rotundata]
	Unigene3754	NO	PREDICTED: hypothetical protein LOC100122162 [Nasonia vitripennis]
	Unigene3784	NO	Ran-binding protein 3 [Camponotus floridanus]
	Unigene383	NO	PREDICTED: cytochrome c-like isoform 1 [Bombus terrestris]
	Unigene3866	NO	hypothetical protein EAI_17259 [Harpegnathos saltator]
	Unigene3882	NO	PREDICTED: NADH dehydrogenase [ubiquinone] iron-sulfur protein 4, mitochondrial-like [Megachile rotundata]
	Unigene3960	NO	PREDICTED: myb-related protein A-like [Nasonia vitripennis]
	Unigene4045	NO	Dual specificity mitogen-activated protein kinase kinase 4 [Acromyrmex echinatior]
	Unigene4061	NO	hypothetical protein AND_01489 [Anopheles darlingi]
	Unigene4066	NO	PREDICTED: uncharacterized protein LOC100875487 [Megachile rotundata]
	Unigene4072	NO	hypothetical protein KGM_03909 [Danaus plexippus]
	Unigene4101	NO	hypothetical protein SINV_05984 [Solenopsis invicta]
	Unigene4107	NO	hypothetical protein SINV_09114 [Solenopsis invicta]
	Unigene411	NO	cytochrome c oxidase subunit VI [Microplitis mediator]
	Unigene412	NO	hypothetical protein SINV_01823 [Solenopsis invicta]
	Unigene4122	NO	mitochondrial cytochrome c oxidase subunit VIa [Bombyx mori]

Unigene4136	NO	Myb protein [Camponotus floridanus]
Unigene4156	NO	PREDICTED: rho-associated protein kinase 2-like isoform 2 [Nasonia vitripennis]
Unigene4193	NO	hypothetical protein SINV_10623 [Solenopsis invicta]
Unigene426	NO	cytochrome c oxidase-like protein [Glyptapanteles flavicoxis]
Unigene4333	NO	H+ transporting ATP synthase gamma subunit [Danaus plexippus]
Unigene4353	NO	RING finger protein unkempt-like protein [Harpegnathos saltator]
Unigene438	NO	cytochrome b-c1 complex subunit 9 [Nasonia vitripennis]
Unigene4442	NO	PREDICTED: insulin-degrading enzyme-like isoform 2 [Nasonia vitripennis]
Unigene4470	NO	Ras-like GTP-binding protein RHO [Lepeophtheirus salmonis]
Unigene4485	NO	PREDICTED: LOW QUALITY PROTEIN: talin-1-like [Megachile rotundata]
Unigene452	NO	PREDICTED: activating transcription factor of chaperone-like [Megachile rotundata]
Unigene4535	NO	hypothetical protein SINV_09114 [Solenopsis invicta]
Unigene4584	NO	PREDICTED: NADH-ubiquinone oxidoreductase 75 kDa subunit, mitochondrial-like [Bombus terrestris]
Unigene4589	NO	Low-density lipoprotein receptor-related protein 1 [Harpegnathos saltator]
Unigene4655	NO	PREDICTED: transcription factor E2F3-like isoform 1 [Bombus terrestris]
Unigene466	NO	PREDICTED: hypothetical protein LOC100747421 [Bombus impatiens]
Unigene467	NO	hypothetical protein SINV_11135 [Solenopsis invicta]
Unigene4710	NO	Dual specificity mitogen-activated protein kinase kinase 4 [Acromyrmex echinatior]
Unigene4742	NO	PREDICTED: calmodulin-like protein 4-like isoform 1 [Bombus terrestris]
Unigene4774	NO	mitochondrial cytochrome c [Bombyx mori]
Unigene485	NO	AT15141p [Drosophila melanogaster]
Unigene4852	NO	hypothetical protein KGM_08437 [Danaus plexippus]
Unigene4885	NO	ubiquinol-cytochrome C reductase complex protein [Danaus plexippus]
Unigene4903	NO	putative voltage-dependent anion-selective channel isoform 1 [Danaus plexippus]
Unigene4920	NO	PREDICTED: mitogen-activated protein kinase 1-like [Megachile rotundata]
Unigene4959	NO	ATP synthase [Danaus plexippus]
Unigene4973	NO	PREDICTED: serine/threonine-protein kinase grp isoform 2 [Apis mellifera]
Unigene5020	NO	hypothetical protein AND_02161 [Anopheles darlingi]
Unigene5043	NO	PREDICTED: hypothetical protein LOC100122162 [Nasonia vitripennis]
Unigene5072	NO	putative NADH:ubiquinone dehydrogenase [Danaus plexippus]
Unigene514	NO	PREDICTED: serine/threonine-protein kinase KDX1-like [Bombus impatiens]
Unigene517	NO	GK10857 [Drosophila willistoni]
Unigene5171	NO	PREDICTED: talin-2-like [Bombus terrestris]
Unigene520	NO	cytochrome c oxidase subunit 4 isoform 1, mitochondrial [Nasonia vitripennis]
Unigene5212	NO	Proliferating cell nuclear antigen [Camponotus floridanus]
Unigene5280	NO	PREDICTED: LOW QUALITY PROTEIN: NEDD8-activating enzyme E1 regulatory subunit [Apis mellifera]
Unigene533	NO	ATP synthase subunit b, mitochondrial [Acromyrmex echinatior]
Unigene5357	NO	PREDICTED: exportin-1-like [Nasonia vitripennis]
Unigene5446	NO	PREDICTED: G1/S-specific cyclin-D2-like [Megachile rotundata]
Unigene5492	NO	hypothetical protein KGM_00095 [Danaus plexippus]
Unigene5515	NO	PREDICTED: armadillo segment polarity protein-like [Megachile rotundata]
Unigene553	NO	PREDICTED: cytochrome b-c1 complex subunit 6, mitochondrial-like [Megachile rotundata]
Unigene558	NO	AGAP008724-PA [Anopheles gambiae str. PEST]
Unigene5593	NO	PREDICTED: hypothetical protein LOC100745810 isoform 2 [Bombus impatiens]
Unigene5630	NO	PREDICTED: rho-associated protein kinase 2-like isoform 2 [Nasonia vitripennis]
Unigene564	NO	PREDICTED: similar to mitochondrial ATP synthase coupling factor 6 [Tribolium castaneum]
Unigene5691	NO	PREDICTED: hypothetical protein LOC412916 [Apis mellifera]
Unigene579	NO	cytochrome c oxidase subunit VIb isoform 1 [Rhipicephalus sanguineus]
Unigene5806	NO	PREDICTED: uncharacterized protein LOC100878829 [Megachile rotundata]
Unigene6006	NO	PREDICTED: rho-associated protein kinase 2 [Apis florea]
Unigene6060	NO	lethal neo18 protein [Danaus plexippus]
Unigene608	NO	hypothetical protein EAG_05916 [Camponotus floridanus]
Unigene614	NO	Cytochrome b-c1 complex subunit Rieske, mitochondrial [Camponotus floridanus]
Unigene6140	NO	Ras-like GTP-binding protein Rho1 [Danaus plexippus]
Unigene624	NO	ATP synthase subunit gamma, mitochondrial [Nasonia vitripennis]
Unigene626	NO	Voltage-dependent anion-selective channel [Acromyrmex echinatior]
Unigene6363	NO	Mitogen-activated protein kinase 1 [Harpegnathos saltator]
Unigene6394	NO	Talin-1 [Camponotus floridanus]
Unigene647	NO	PREDICTED: cytochrome b-c1 complex subunit Rieske, mitochondrial-like [Cavia porcellus]
Unigene6600	NO	PREDICTED: presenilin homolog [Bombus impatiens]
Unigene663	NO	Cytochrome b-c1 complex subunit 7 [Camponotus floridanus]

Unigene6630	NO	cytochrome c oxidase [Danaus plexippus]
Unigene6640	NO	Nicastrin [Camponotus floridanus]
Unigene6642	NO	NADH dehydrogenase [Danaus plexippus]
Unigene6671	NO	Ras-like GTP-binding protein RHO [Lepeophtheirus salmonis]
Unigene669	NO	PREDICTED: protein enhancer of sevenless 2B-like [Nasonia vitripennis]
Unigene6695	NO	unnamed protein product [Heliconius melpomene]
Unigene6700	NO	Talin-1 [Camponotus floridanus]
Unigene6842	NO	guanine nucleotide binding protein alpha q polypeptide [Glossina morsitans morsitans]
Unigene6884	NO	H+ transporting ATP synthase O subunit isoform 1 [Bombyx mori]
Unigene6899	NO	PREDICTED: proliferating cell nuclear antigen-like [Nasonia vitripennis]
Unigene694	NO	ATP synthase subunit O, mitochondrial [Camponotus floridanus]
Unigene6948	NO	PREDICTED: anaphase-promoting complex subunit CDC26-like isoform 1 [Apis florea]
Unigene698	NO	ADP/ATP translocase [Manduca sexta]
Unigene6991	NO	PREDICTED: anaphase-promoting complex subunit 10-like [Megachile rotundata]
Unigene7027	NO	AGAP005160-PA [Anopheles gambiae str. PEST]
Unigene7044	NO	PREDICTED: cell division cycle protein 20 homolog [Canis lupus familiaris]
Unigene7068	NO	PREDICTED: nuclear transcription factor Y subunit beta-like [Bombus impatiens]
Unigene71	NO	hypothetical protein EAI_14262 [Harpegnathos saltator]
Unigene7100	NO	cytochrome c oxidase polypeptide IV [Bombyx mori]
Unigene7102	NO	Multiple C2 and transmembrane domain-containing protein 2 [Acromyrmex echinatior]
Unigene7161	NO	PREDICTED: sideroflexin-1-like isoform 2 [Nasonia vitripennis]
Unigene7180	NO	39S ribosomal protein L46, mitochondrial [Acromyrmex echinatior]
Unigene722	NO	PREDICTED: ras-like GTP-binding protein Rho1 [Nasonia vitripennis]
Unigene7225	NO	PREDICTED: hypothetical protein LOC408577 [Apis mellifera]
Unigene7237	NO	PREDICTED: hypothetical protein LOC100747412 isoform 2 [Bombus impatiens]
Unigene731	NO	hypothetical protein SINV_08923 [Solenopsis invicta]
Unigene7340	NO	hypothetical protein SINV_10840 [Solenopsis invicta]
Unigene743	NO	GF11012 [Drosophila ananassae]
Unigene7464	NO	Eukaryotic translation initiation factor 2-alpha kinase [Acromyrmex echinatior]
Unigene7469	NO	NADH-ubiquinone oxidoreductase 75 kDa subunit, mitochondrial precursor [Nasonia vitripennis]
Unigene7498	NO	hypothetical protein SINV_12469 [Solenopsis invicta]
Unigene7595	NO	PREDICTED: anaphase-promoting complex subunit 11-like [Apis mellifera]
Unigene7619	NO	hypothetical protein SINV_04265 [Solenopsis invicta]
Unigene763	NO	hypothetical protein SINV_15147 [Solenopsis invicta]
Unigene7655	NO	PREDICTED: cell division cycle protein 27 homolog [Megachile rotundata]
Unigene7671	NO	PREDICTED: rho-associated protein kinase 2 [Megachile rotundata]
Unigene7714	NO	Mitogen-activated protein kinase 14B [Harpegnathos saltator]
Unigene7739	NO	control protein HCTL024 [Heliconius hortense]
Unigene7754	NO	PREDICTED: cyclin-dependent kinase 6-like [Apis florea]
Unigene776	NO	AT15141p [Drosophila melanogaster]
Unigene7857	NO	hypothetical protein SINV_10861 [Solenopsis invicta]
Unigene787	NO	NADH dehydrogenase [ubiquinone] 1 alpha subcomplex subunit 6 [Nasonia vitripennis]
Unigene7938	NO	hypothetical protein KGM_04538 [Danaus plexippus]
Unigene7980	NO	PREDICTED: dual specificity mitogen-activated protein kinase kinase 2-like [Apis florea]
Unigene799	NO	hypothetical protein SINV_06969 [Solenopsis invicta]
Unigene7994	NO	PREDICTED: hypothetical protein LOC100122162 [Nasonia vitripennis]
Unigene8049	NO	Talin-1 [Camponotus floridanus]
Unigene8102	NO	hypothetical protein SINV_10861 [Solenopsis invicta]
Unigene8104	NO	NADH dehydrogenase [Danaus plexippus]
Unigene8113	NO	Gamma-secretase subunit Aph-1 [Camponotus floridanus]
Unigene8196	NO	cytochrome c oxidase subunit III [Cotesia vestalis]
Unigene8222	NO	ubiquinol-cytochrome C reductase complex 14kD subunit [Danaus plexippus]
Unigene8266	NO	PREDICTED: mothers against decapentaplegic homolog 3 [Nasonia vitripennis]
Unigene8267	NO	Insulin-degrading enzyme [Camponotus floridanus]
Unigene829	NO	ATP synthase subunit d, mitochondrial [Camponotus floridanus]
Unigene8339	NO	PREDICTED: hypothetical protein LOC100743562 isoform 2 [Bombus impatiens]
Unigene8344	NO	PREDICTED: phosphatidylinositol 3-kinase regulatory subunit alpha-like, partial [Apis florea]
Unigene837	NO	hypothetical protein AND_29231 [Anopheles darlingi]
Unigene8372	NO	PREDICTED: baculoviral IAP repeat-containing protein 3-like [Nasonia vitripennis]
Unigene8416	NO	H+ transporting ATP synthase O subunit isoform 1 [Bombyx mori]
Unigene8427	NO	PREDICTED: rho-associated protein kinase 2 [Megachile rotundata]
Unigene8488	NO	PREDICTED: uncharacterized protein LOC100877711 [Megachile rotundata]

	Unigene8610	NO	PREDICTED: talin-1-like [Apis mellifera]
	Unigene8714	NO	PREDICTED: G protein-coupled receptor kinase 1-like [Megachile rotundata]
	Unigene8755	NO	PREDICTED: LOW QUALITY PROTEIN: ribosomal protein S6 kinase beta-1-like [Apis florea]
	Unigene8839	NO	Rho-associated protein kinase 2 [Camponotus floridanus]
	Unigene8887	NO	PREDICTED: gamma-secretase subunit Aph-1-like [Megachile rotundata]
	Unigene8922	NO	mitochondrial ATP synthase coupling factor [Antheraea yamamai]
	Unigene9008	NO	PREDICTED: guanine nucleotide-binding protein G(q) subunit alpha-like isoform 5 [Megachile rotundata]
	Unigene905	NO	PREDICTED: transcription factor AP-1-like [Megachile rotundata]
	Unigene912	NO	cytochrome c1, heme protein, mitochondrial [Nasonia vitripennis]
	Unigene9141	NO	PREDICTED: hypothetical protein LOC100743562 isoform 1 [Bombus impatiens]
	Unigene9217	NO	PREDICTED: stress-activated protein kinase JNK-like [Megachile rotundata]
	Unigene9292	NO	PREDICTED: LOW QUALITY PROTEIN: talin-2-like [Apis florea]
	Unigene9295	NO	PREDICTED: rho-associated protein kinase 2-like [Bombus impatiens]
	Unigene9328	NO	PREDICTED: LOW QUALITY PROTEIN: talin-1-like [Megachile rotundata]
	Unigene934	NO	PREDICTED: farnesyl pyrophosphate synthase-like [Nasonia vitripennis]
	Unigene9356	NO	pancreatic lipase 3 [Mamestra configurata]
	Unigene9359	NO	PREDICTED: hypothetical protein LOC100743083 [Bombus impatiens]
	Unigene9378	NO	PREDICTED: armadillo segment polarity protein-like [Megachile rotundata]
	Unigene9415	NO	PREDICTED: mitogen-activated protein kinase 14B-like isoform 2 [Megachile rotundata]
	Unigene9462	NO	NADH dehydrogenase ubiquinone Fe-S 8 [Bombyx mori]
	Unigene9485	NO	H+ transporting ATP synthase O subunit isoform 1 [Danaus plexippus]
	Unigene9517	NO	PREDICTED: insulin-degrading enzyme-like isoform 2 [Nasonia vitripennis]
	Unigene952	NO	PREDICTED: acyl carrier protein, mitochondrial-like [Megachile rotundata]
	Unigene9582	NO	PREDICTED: talin-2-like [Bombus impatiens]
	Unigene959	NO	NADH dehydrogenase [ubiquinone] 1 beta subcomplex subunit 5, mitochondrial [Nasonia vitripennis]
	Unigene9659	NO	sarco/endoplasmic reticulum calcium ATPase [Bombyx mori]
	Unigene975	NO	Growth hormone-inducible transmembrane protein [Camponotus floridanus]
	Unigene9782	NO	Segment polarity protein dishevelled-like protein DVL-3 [Harpegnathos saltator]
	Unigene991	NO	PREDICTED: NADH dehydrogenase [ubiquinone] 1 alpha subcomplex subunit 7-like [Megachile rotundata]
	Unigene9927	NO	Subunit VIb of cytochrome c oxidase [Danaus plexippus]
	Unigene9971	NO	hypothetical protein KGM_15724 [Danaus plexippus]
	Unigene9998	NO	PREDICTED: stress-activated protein kinase JNK-like [Megachile rotundata]
	CL363.Contig1	NO	PREDICTED: serine/threonine-protein phosphatase 2B catalytic subunit 2-like [Apis florea]
	CL124.Contig1	NO	succinate dehydrogenase cytochrome b560 subunit, mitochondrial [Nasonia vitripennis]
	CL22.Contig1	SP	hypothetical protein EAG_13414 [Camponotus floridanus]
	CL690.Contig1	SP	hypothetical protein SINV_04265 [Solenopsis invicta]
	CL690.Contig2	SP	GJ24134 [Drosophila virilis]
	Unigene11423	SP	PREDICTED: LOW QUALITY PROTEIN: laminin subunit gamma-1-like [Megachile rotundata]
	Unigene12612	SP	Armadillo segment polarity protein [Harpegnathos saltator]
	Unigene1366	SP	Protein 60A [Camponotus floridanus]
	Unigene3097	SP	PREDICTED: lipase member H-A-like [Apis mellifera]
	Unigene3100	SP	PREDICTED: uncharacterized protein LOC100867171 [Apis florea]
	Unigene758	SP	Protein toll [Harpegnathos saltator]
	Unigene96	SP	Vascular endothelial growth factor receptor 2 [Harpegnathos saltator]
	CL212.Contig1	NO	Cordon-bleu protein-like 1 [Harpegnathos saltator]
	CL238.Contig1	NO	PREDICTED: protein kinase shaggy-like isoform 2 [Nasonia vitripennis]
	CL238.Contig2	NO	PREDICTED: protein kinase shaggy-like isoform 2 [Nasonia vitripennis]
	CL238.Contig4	NO	Protein kinase shaggy [Acromyrmex echinatior]
	CL239.Contig1	NO	PREDICTED: uncharacterized protein LOC100878829 [Megachile rotundata]
	CL363.Contig1	NO	PREDICTED: serine/threonine-protein phosphatase 2B catalytic subunit 2-like [Apis florea]
	CL363.Contig2	NO	PREDICTED: serine/threonine-protein phosphatase 2B catalytic subunit 2-like [Apis florea]
	CL375.Contig1	NO	guanine nucleotide-binding protein subunit beta-like [Microplitis mediator]
	CL375.Contig2	NO	guanine nucleotide-binding protein subunit beta-like [Microplitis mediator]
	CL417.Contig1	NO	PREDICTED: dual specificity protein phosphatase 10-like [Bombus terrestris]
	CL417.Contig2	NO	PREDICTED: dual specificity protein phosphatase 10-like [Megachile rotundata]
	CL466.Contig1	NO	sugar transporter, putative [Ixodes scapularis]
	CL481.Contig1	NO	ring box protein [Bombyx mori]

CL483.Contig1	NO	PREDICTED: hypothetical protein LOC100743933 [Bombus impatiens]
CL483.Contig2	NO	Microtubule-associated protein 2 [Harpegnathos saltator]
CL51.Contig2	NO	hypothetical protein SINV_04265 [Solenopsis invicta]
CL515.Contig1	NO	hypothetical protein SINV_06010 [Solenopsis invicta]
CL515.Contig2	NO	hypothetical protein SINV_06010 [Solenopsis invicta]
CL591.Contig1	NO	PREDICTED: E3 ubiquitin-protein ligase IAP-3-like [Bombus impatiens]
CL591.Contig2	NO	PREDICTED: E3 ubiquitin-protein ligase IAP-3-like [Megachile rotundata]
CL624.Contig1	NO	S-phase kinase-associated protein [Danaus plexippus]
CL624.Contig2	NO	S-phase kinase-associated protein [Danaus plexippus]
CL663.Contig1	NO	Serine/threonine-protein phosphatase 2A 65 kDa regulatory subunit A alpha isoform [Camponotus floridanus]
CL663.Contig2	NO	Serine/threonine-protein phosphatase 2A 65 kDa regulatory subunit A alpha isoform [Camponotus floridanus]
CL724.Contig1	NO	PREDICTED: dual specificity protein phosphatase Mpk3-like [Megachile rotundata]
CL735.Contig2	NO	G protein-coupled receptor kinase 1 [Camponotus floridanus]
CL757.Contig2	NO	PREDICTED: uncharacterized protein LOC100876927 [Megachile rotundata]
CL792.Contig1	NO	hypothetical protein SINV_06702 [Solenopsis invicta]
Unigene1004	NO	hypothetical protein SINV_14598 [Solenopsis invicta]
Unigene10082	NO	type IV collagen [Bombyx mori]
Unigene10083	NO	putative armadillo protein [Danaus plexippus]
Unigene10121	NO	85 kDa calcium-independent phospholipase A2 [Camponotus floridanus]
Unigene10125	NO	hypothetical protein SINV_80464 [Solenopsis invicta]
Unigene10138	NO	PREDICTED: uncharacterized protein LOC100881293 [Megachile rotundata]
Unigene10184	NO	hypothetical protein DAPPUDRAFT_304184 [Daphnia pulex]
Unigene10223	NO	PREDICTED: ribosomal protein S6 kinase beta-1-like [Apis mellifera]
Unigene10321	NO	PREDICTED: retinoblastoma-like protein 1-like isoform 2 [Bombus impatiens]
Unigene10343	NO	PREDICTED: bone morphogenetic protein receptor type-1B-like, partial [Nasonia vitripennis]
Unigene10365	NO	PREDICTED: activin receptor type-2B [Apis mellifera]
Unigene10410	NO	Mothers against decapentaplegic-like protein 3 [Acromyrmex echinatior]
Unigene10531	NO	phospholipase A2 precursor [Apis mellifera]
Unigene10610	NO	PREDICTED: hypothetical protein LOC100114432 [Nasonia vitripennis]
Unigene10672	NO	putative collagen alpha-2IV chain protein [Danaus plexippus]
Unigene10828	NO	PREDICTED: calcium/calmodulin-dependent protein kinase type II alpha chain-like isoform 3 [Bombus terrestris]
Unigene10884	NO	hypothetical protein KGM_05418 [Danaus plexippus]
Unigene1089	NO	Interferon regulatory factor 2-binding protein 2-A [Harpegnathos saltator]
Unigene10891	NO	putative laminin A chain [Danaus plexippus]
Unigene10956	NO	PREDICTED: LOW QUALITY PROTEIN: RING finger and SPRY domain-containing protein 1-like [Bombus terrestris]
Unigene11062	NO	Stress-activated protein kinase JNK [Acromyrmex echinatior]
Unigene11077	NO	PREDICTED: dual specificity protein phosphatase Mpk3-like [Megachile rotundata]
Unigene11087	NO	PREDICTED: guanine nucleotide-binding protein G(i) subunit alpha-like [Megachile rotundata]
Unigene11124	NO	Presenilin-like protein [Harpegnathos saltator]
Unigene11160	NO	hypothetical protein TcasGA2_TC014326 [Tribolium castaneum]
Unigene11200	NO	PREDICTED: rho-associated protein kinase 2-like [Bombus impatiens]
Unigene11213	NO	Phosphatidylinositol 3-kinase regulatory subunit alpha [Camponotus floridanus]
Unigene11321	NO	hypothetical protein SINV_80464 [Solenopsis invicta]
Unigene11447	NO	hypothetical protein SINV_14862 [Solenopsis invicta]
Unigene11450	NO	conserved hypothetical protein [Pediculus humanus corporis]
Unigene11455	NO	PREDICTED: serine/threonine/tyrosine-interacting protein-like [Bombus impatiens]
Unigene11518	NO	PREDICTED: mitogen-activated protein kinase kinase kinase 4-like [Bombus terrestris]
Unigene11598	NO	PREDICTED: group XIIA secretory phospholipase A2-like [Apis florea]
Unigene11613	NO	PREDICTED: calcium-independent phospholipase A2-gamma-like [Bombus terrestris]
Unigene11645	NO	putative ETS-like protein pointed, isoform P1 [Danaus plexippus]
Unigene11659	NO	GTP-binding protein (i) alpha subunit, gnai [Aedes aegypti]
Unigene11690	NO	PREDICTED: protein son of sevenless-like [Megachile rotundata]
Unigene11985	NO	PREDICTED: hypothetical protein LOC408354 [Apis mellifera]
Unigene11993	NO	Mitogen-activated protein kinase kinase kinase 4 [Harpegnathos saltator]
Unigene1213	NO	PREDICTED: calcineurin subunit B type 2-like isoform 1 [Nasonia vitripennis]
Unigene1216	NO	PREDICTED: transmembrane protein 208-like [Bombus impatiens]
Unigene12247	NO	PREDICTED: hypothetical protein LOC100743521 [Bombus impatiens]
Unigene1263	NO	PREDICTED: microtubule-associated protein tau-like [Apis florea]
Unigene12641	NO	PREDICTED: LOW QUALITY PROTEIN: phospholipase D1-like [Bombus terrestris]
Unigene12735	NO	PREDICTED: armadillo segment polarity protein isoform 2 [Nasonia vitripennis]

Cell differentiation	Unigene1310	NO	PREDICTED: troponin C, isoform 3 isoform 1 [Nasonia vitripennis]
	Unigene13138	NO	PREDICTED: segment polarity protein dishevelled homolog DVL-3-like [Megachile rotundata]
	Unigene13191	NO	PREDICTED: cyclic AMP-dependent transcription factor ATF-2-like [Apis mellifera]
	Unigene13259	NO	PREDICTED: phospholipase D2-like [Apis florea]
	Unigene13553	NO	PREDICTED: protein BCL9 homolog [Apis florea]
	Unigene13568	NO	PREDICTED: protein mothers against dpp-like [Megachile rotundata]
	Unigene13635	NO	PREDICTED: LOW QUALITY PROTEIN: BTB/POZ domain-containing protein KCTD3-like [Nasonia vitripennis]
	Unigene13736	NO	hypothetical protein KGM_03021 [Danaus plexippus]
	Unigene1454	NO	Ras-related protein Rab-7a [Harpegnathos saltator]
	Unigene1513	NO	conserved hypothetical protein [Pediculus humanus corporis]
	Unigene1580	NO	hypothetical protein EAI_14262 [Harpegnathos saltator]
	Unigene1630	NO	small heat shock protein [Pteromalus puparum]
	Unigene1894	NO	Ras-like GTP-binding protein Rho1 [Salmo salar]
	Unigene1925	NO	PREDICTED: cdc42 homolog isoform 1 [Nasonia vitripennis]
	Unigene1927	NO	PREDICTED: SH3 domain-binding glutamic acid-rich protein homolog [Megachile rotundata]
	Unigene2017	NO	hypothetical protein AaeL_AAEL006826 [Aedes aegypti]
	Unigene2069	NO	PREDICTED: ras-like protein 1-like [Apis florea]
	Unigene2152	NO	Guanine nucleotide-binding protein subunit gamma-1 [Camponotus floridanus]
	Unigene2210	NO	PREDICTED: signal transducer and activator of transcription 5B-like [Megachile rotundata]
	Unigene2299	NO	PREDICTED: s-phase kinase-associated protein 1-like [Amphimedon queenslandica]
	Unigene2339	NO	Guanine nucleotide-binding protein subunit gamma-e [Harpegnathos saltator]
	Unigene2418	NO	PREDICTED: RAC serine/threonine-protein kinase-like [Bombus impatiens]
	Unigene25	NO	PREDICTED: ras-like protein 2-like [Nasonia vitripennis]
	Unigene2504	NO	hypothetical protein TRIADDRAFT_20496 [Trichoplax adhaerens]
	Unigene2544	NO	PREDICTED: uncharacterized protein LOC100879259 [Megachile rotundata]
	Unigene2630	NO	PREDICTED: ribosomal protein S6 kinase beta-1-like [Apis florea]
	Unigene2925	NO	PREDICTED: hypothetical protein LOC409983 [Apis mellifera]
	Unigene2974	NO	PREDICTED: transcription factor Dp-1-like [Megachile rotundata]
	Unigene3005	NO	PREDICTED: calumenin-like [Nasonia vitripennis]
	Unigene3146	NO	Ran-binding protein 9 [Camponotus floridanus]
	Unigene3188	NO	PREDICTED: serine/threonine-protein phosphatase 2A catalytic subunit beta isoform-like [Amphimedon queenslandica]
	Unigene3288	NO	Zinc finger FYVE domain-containing protein 19 [Harpegnathos saltator]
	Unigene3316	NO	PREDICTED: calcium-binding protein p22-like [Megachile rotundata]
	Unigene3344	NO	PREDICTED: solute carrier family 2, facilitated glucose transporter member 1-like [Nasonia vitripennis]
	Unigene3692	NO	PREDICTED: LOW QUALITY PROTEIN: G protein-coupled receptor kinase 1-like [Apis florea]
	Unigene3774	NO	PREDICTED: uncharacterized protein LOC100874971 [Megachile rotundata]
	Unigene383	NO	PREDICTED: cytochrome c-like isoform 1 [Bombus terrestris]
	Unigene3861	NO	PREDICTED: group XIIA secretory phospholipase A2-like [Nasonia vitripennis]
	Unigene4	NO	hypothetical protein KGM_05418 [Danaus plexippus]
	Unigene4045	NO	Dual specificity mitogen-activated protein kinase kinase 4 [Acromyrmex echinatior]
	Unigene4101	NO	hypothetical protein SINV_05984 [Solenopsis invicta]
	Unigene4156	NO	PREDICTED: rho-associated protein kinase 2-like isoform 2 [Nasonia vitripennis]
	Unigene4303	NO	extra macrochaetae [Apis mellifera]
	Unigene4353	NO	RING finger protein unkempt-like protein [Harpegnathos saltator]
	Unigene4470	NO	Ras-like GTP-binding protein RHO [Lepeophtheirus salmonis]
	Unigene452	NO	PREDICTED: activating transcription factor of chaperone-like [Megachile rotundata]
	Unigene4604	NO	hypothetical protein KGM_05418 [Danaus plexippus]
	Unigene4655	NO	PREDICTED: transcription factor E2F3-like isoform 1 [Bombus terrestris]
	Unigene4710	NO	Dual specificity mitogen-activated protein kinase kinase 4 [Acromyrmex echinatior]
	Unigene4717	NO	PREDICTED: cullin-1 isoform 2 [Megachile rotundata]
	Unigene4742	NO	PREDICTED: calmodulin-like protein 4-like isoform 1 [Bombus terrestris]
	Unigene4774	NO	mitochondrial cytochrome c [Bombyx mori]
	Unigene4829	NO	PREDICTED: retinoblastoma-family protein [Apis mellifera]
	Unigene485	NO	AT15141p [Drosophila melanogaster]
	Unigene4920	NO	PREDICTED: mitogen-activated protein kinase 1-like [Megachile rotundata]
	Unigene4950	NO	PREDICTED: dual specificity mitogen-activated protein kinase kinase 6-like [Megachile rotundata]
	Unigene5047	NO	PREDICTED: NFU1 iron-sulfur cluster scaffold homolog, mitochondrial-like [Bombus terrestris]

Unigene514	NO	PREDICTED: serine/threonine-protein kinase KDX1-like [Bombus impatiens]
Unigene5161	NO	PREDICTED: hypothetical protein LOC409983 [Apis mellifera]
Unigene5515	NO	PREDICTED: armadillo segment polarity protein-like [Megachile rotundata]
Unigene5593	NO	PREDICTED: hypothetical protein LOC100745810 isoform 2 [Bombus impatiens]
Unigene5630	NO	PREDICTED: rho-associated protein kinase 2-like isoform 2 [Nasonia vitripennis]
Unigene5691	NO	PREDICTED: hypothetical protein LOC412916 [Apis mellifera]
Unigene5806	NO	PREDICTED: uncharacterized protein LOC100878829 [Megachile rotundata]
Unigene6006	NO	PREDICTED: rho-associated protein kinase 2 [Apis florea]
Unigene6140	NO	Ras-like GTP-binding protein Rho1 [Danaus plexippus]
Unigene6195	NO	PREDICTED: dual specificity protein phosphatase Mpk3-like [Bombus impatiens]
Unigene6295	NO	PREDICTED: dual specificity mitogen-activated protein kinase kinase hemipterous-like isoform 2 [Megachile rotundata]
Unigene6354	NO	PREDICTED: similar to bone morphogenetic protein 5 preproprotein [Hydra magnipapillata]
Unigene6363	NO	Mitogen-activated protein kinase 1 [Harpegnathos saltator]
Unigene639	NO	conserved hypothetical protein [Pediculus humanus corporis]
Unigene6600	NO	PREDICTED: presenilin homolog [Bombus impatiens]
Unigene6671	NO	Ras-like GTP-binding protein RHO [Lepeophtheirus salmonis]
Unigene669	NO	PREDICTED: protein enhancer of sevenless 2B-like [Nasonia vitripennis]
Unigene6735	NO	hypothetical protein SINV_14598 [Solenopsis invicta]
Unigene7027	NO	AGAP005160-PA [Anopheles gambiae str. PEST]
Unigene7074	NO	PREDICTED: zinc finger FYVE domain-containing protein 9-like [Megachile rotundata]
Unigene71	NO	hypothetical protein EAI_14262 [Harpegnathos saltator]
Unigene7102	NO	Multiple C2 and transmembrane domain-containing protein 2 [Acromyrmex echinatior]
Unigene722	NO	PREDICTED: ras-like GTP-binding protein Rho1 [Nasonia vitripennis]
Unigene7225	NO	PREDICTED: hypothetical protein LOC408577 [Apis mellifera]
Unigene7314	NO	Cullin-1 [Acromyrmex echinatior]
Unigene7498	NO	hypothetical protein SINV_12469 [Solenopsis invicta]
Unigene7543	NO	hypothetical protein SINV_04935 [Solenopsis invicta]
Unigene7671	NO	PREDICTED: rho-associated protein kinase 2 [Megachile rotundata]
Unigene7714	NO	Mitogen-activated protein kinase 14B [Harpegnathos saltator]
Unigene7754	NO	PREDICTED: cyclin-dependent kinase 6-like [Apis florea]
Unigene776	NO	AT15141p [Drosophila melanogaster]
Unigene7818	NO	PREDICTED: LOW QUALITY PROTEIN: retinoblastoma-like protein 1-like [Apis florea]
Unigene7916	NO	Dual specificity mitogen-activated protein kinase kinase 7 [Harpegnathos saltator]
Unigene7980	NO	PREDICTED: dual specificity mitogen-activated protein kinase kinase 2-like [Apis florea]
Unigene8266	NO	PREDICTED: mothers against decapentaplegic homolog 3 [Nasonia vitripennis]
Unigene8344	NO	PREDICTED: phosphatidylinositol 3-kinase regulatory subunit alpha-like, partial [Apis florea]
Unigene8372	NO	PREDICTED: baculoviral IAP repeat-containing protein 3-like [Nasonia vitripennis]
Unigene8408	NO	putative laminin A chain [Danaus plexippus]
Unigene8427	NO	PREDICTED: rho-associated protein kinase 2 [Megachile rotundata]
Unigene8488	NO	PREDICTED: uncharacterized protein LOC100877711 [Megachile rotundata]
Unigene8515	NO	PREDICTED: hypothetical protein LOC100642907 [Bombus terrestris]
Unigene8581	NO	hypothetical protein SINV_13032 [Solenopsis invicta]
Unigene8714	NO	PREDICTED: G protein-coupled receptor kinase 1-like [Megachile rotundata]
Unigene8755	NO	PREDICTED: LOW QUALITY PROTEIN: ribosomal protein S6 kinase beta-1-like [Apis florea]
Unigene8759	NO	Phospholipase D1 [Harpegnathos saltator]
Unigene8772	NO	putative collagen alpha-2IV chain protein [Danaus plexippus]
Unigene8839	NO	Rho-associated protein kinase 2 [Camponotus floridanus]
Unigene905	NO	PREDICTED: transcription factor AP-1-like [Megachile rotundata]
Unigene9081	NO	putative laminin A chain [Danaus plexippus]
Unigene9217	NO	PREDICTED: stress-activated protein kinase JNK-like [Megachile rotundata]
Unigene9259	NO	PREDICTED: calcium/calmodulin-dependent protein kinase type II alpha chain-like isoform 1 [Bombus impatiens]
Unigene9295	NO	PREDICTED: rho-associated protein kinase 2-like [Bombus impatiens]
Unigene9356	NO	pancreatic lipase 3 [Mamestra configurata]
Unigene9359	NO	PREDICTED: hypothetical protein LOC100743083 [Bombus impatiens]
Unigene9378	NO	PREDICTED: armadillo segment polarity protein-like [Megachile rotundata]
Unigene9404	NO	Transcription factor E2F5 [Camponotus floridanus]
Unigene9415	NO	PREDICTED: mitogen-activated protein kinase 14B-like isoform 2 [Megachile rotundata]

	Unigene9499	NO	hypothetical protein KGM_05418 [Danaus plexippus]
	Unigene9604	NO	PREDICTED: protein mothers against dpp-like [Megachile rotundata]
	Unigene975	NO	Growth hormone-inducible transmembrane protein [Camponotus floridanus]
	Unigene9768	NO	PREDICTED: LOW QUALITY PROTEIN: retinoblastoma-like protein 1-like [Apis florea]
	Unigene9782	NO	Segment polarity protein dishevelled-like protein DVL-3 [Harpegnathos saltator]
	Unigene9998	NO	PREDICTED: stress-activated protein kinase JNK-like [Megachile rotundata]
	CL334.Contig1	SP	Calcium-transporting ATPase sarcoplasmic/endoplasmic reticulum type [Acromyrmex echinatior]
	CL690.Contig1	SP	hypothetical protein SINV_04265 [Solenopsis invicta]
	CL690.Contig2	SP	GJ24134 [Drosophila virilis]
	Unigene1011	SP	PREDICTED: membrane metallo-endopeptidase-like 1-like [Bombus impatiens]
	Unigene10722	SP	Protein VAC14-like protein [Camponotus floridanus]
	Unigene11423	SP	PREDICTED: LOW QUALITY PROTEIN: laminin subunit gamma-1-like [Megachile rotundata]
	Unigene12612	SP	Armadillo segment polarity protein [Harpegnathos saltator]
	Unigene244	SP	calreticulin [Cotesia rubecula]
	Unigene3386	SP	Calnexin [Camponotus floridanus]
	Unigene6782	SP	PREDICTED: calnexin-like [Megachile rotundata]
	Unigene96	SP	Vascular endothelial growth factor receptor 2 [Harpegnathos saltator]
	CL238.Contig1	NO	PREDICTED: protein kinase shaggy-like isoform 2 [Nasonia vitripennis]
	CL238.Contig1	NO	PREDICTED: protein kinase shaggy-like isoform 2 [Nasonia vitripennis]
	CL238.Contig2	NO	PREDICTED: protein kinase shaggy-like isoform 2 [Nasonia vitripennis]
	CL238.Contig4	NO	Protein kinase shaggy [Acromyrmex echinatior]
	CL239.Contig1	NO	PREDICTED: uncharacterized protein LOC100878829 [Megachile rotundata]
	CL363.Contig1	NO	PREDICTED: serine/threonine-protein phosphatase 2B catalytic subunit 2-like [Apis florea]
	CL363.Contig2	NO	PREDICTED: serine/threonine-protein phosphatase 2B catalytic subunit 2-like [Apis florea]
	CL417.Contig1	NO	PREDICTED: dual specificity protein phosphatase 10-like [Bombus terrestris]
	CL417.Contig2	NO	PREDICTED: dual specificity protein phosphatase 10-like [Megachile rotundata]
	CL438.Contig1	NO	PREDICTED: GTP-binding nuclear protein Ran [Apis mellifera]
	CL438.Contig2	NO	GTP-binding nuclear protein ran [Danaus plexippus]
	CL438.Contig3	NO	GTP-binding nuclear protein ran [Danaus plexippus]
	CL466.Contig1	NO	sugar transporter, putative [Ixodes scapularis]
	CL481.Contig1	NO	ring box protein [Bombyx mori]
	CL483.Contig1	NO	PREDICTED: hypothetical protein LOC100743933 [Bombus impatiens]
	CL483.Contig2	NO	Microtubule-associated protein 2 [Harpegnathos saltator]
	CL51.Contig2	NO	hypothetical protein SINV_04265 [Solenopsis invicta]
	CL515.Contig1	NO	hypothetical protein SINV_06010 [Solenopsis invicta]
	CL515.Contig2	NO	hypothetical protein SINV_06010 [Solenopsis invicta]
	CL591.Contig1	NO	PREDICTED: E3 ubiquitin-protein ligase IAP-3-like [Bombus impatiens]
	CL591.Contig2	NO	PREDICTED: E3 ubiquitin-protein ligase IAP-3-like [Megachile rotundata]
	CL663.Contig1	NO	Serine/threonine-protein phosphatase 2A 65 kDa regulatory subunit A alpha isoform [Camponotus floridanus]
	CL663.Contig2	NO	Serine/threonine-protein phosphatase 2A 65 kDa regulatory subunit A alpha isoform [Camponotus floridanus]
	CL676.Contig1	NO	PREDICTED: g1/S-specific cyclin-D2-like [Bombus terrestris]
	CL724.Contig1	NO	PREDICTED: dual specificity protein phosphatase Mpk3-like [Megachile rotundata]
	CL757.Contig2	NO	PREDICTED: uncharacterized protein LOC100876927 [Megachile rotundata]
	CL792.Contig1	NO	hypothetical protein SINV_06702 [Solenopsis invicta]
	Unigene1004	NO	hypothetical protein SINV_14598 [Solenopsis invicta]
	Unigene10082	NO	type IV collagen [Bombyx mori]
	Unigene10083	NO	putative armadillo protein [Danaus plexippus]
	Unigene10090	NO	PREDICTED: LOW QUALITY PROTEIN: g2/mitotic-specific cyclin-B3-like [Bombus terrestris]
	Unigene10121	NO	85 kDa calcium-independent phospholipase A2 [Camponotus floridanus]
	Unigene10125	NO	hypothetical protein SINV_80464 [Solenopsis invicta]
	Unigene10138	NO	PREDICTED: uncharacterized protein LOC100881293 [Megachile rotundata]
	Unigene10183	NO	Talin-1 [Acromyrmex echinatior]
	Unigene10184	NO	hypothetical protein DAPPUDRAFT_304184 [Daphnia pulex]
	Unigene10223	NO	PREDICTED: ribosomal protein S6 kinase beta-1-like [Apis mellifera]
	Unigene10285	NO	PREDICTED: exportin-1-like [Megachile rotundata]
	Unigene10363	NO	GTP-binding protein alpha subunit, gna [Culex quinquefasciatus]
	Unigene10410	NO	Mothers against decapentaplegic-like protein 3 [Acromyrmex echinatior]
	Unigene10437	NO	PREDICTED: exportin-1-like [Megachile rotundata]

Unigene10512	NO	Mitotic checkpoint protein BUB3 [Acromyrmex echinatior]
Unigene10531	NO	phospholipase A2 [Apis mellifera]
Unigene10610	NO	PREDICTED: hypothetical protein LOC100114432 [Nasonia vitripennis]
Unigene10672	NO	putative collagen alpha-2IV chain protein [Danaus plexippus]
Unigene10828	NO	PREDICTED: calcium/calmodulin-dependent protein kinase type II alpha chain-like isoform 3 [Bombus terrestris]
Unigene10884	NO	hypothetical protein KGM_05418 [Danaus plexippus]
Unigene10891	NO	putative laminin A chain [Danaus plexippus]
Unigene10956	NO	PREDICTED: LOW QUALITY PROTEIN: RING finger and SPRY domain-containing protein 1-like [Bombus terrestris]
Unigene11062	NO	Stress-activated protein kinase JNK [Acromyrmex echinatior]
Unigene11077	NO	PREDICTED: dual specificity protein phosphatase Mpk3-like [Megachile rotundata]
Unigene11087	NO	PREDICTED: guanine nucleotide-binding protein G(i) subunit alpha-like [Megachile rotundata]
Unigene1116	NO	Ras-related nuclear protein [Mizuhopecten yessoensis]
Unigene11160	NO	hypothetical protein TcasGA2_TC014326 [Tribolium castaneum]
Unigene1120	NO	PREDICTED: endothelin-converting enzyme 1-like [Nasonia vitripennis]
Unigene11213	NO	Phosphatidylinositol 3-kinase regulatory subunit alpha [Camponotus floridanus]
Unigene11321	NO	hypothetical protein SINV_80464 [Solenopsis invicta]
Unigene11447	NO	hypothetical protein SINV_14862 [Solenopsis invicta]
Unigene11455	NO	PREDICTED: serine/threonine/tyrosine-interacting protein-like [Bombus impatiens]
Unigene1149	NO	hypothetical protein DAPPUDRAFT_51702 [Daphnia pulex]
Unigene11518	NO	PREDICTED: mitogen-activated protein kinase kinase kinase 4-like [Bombus terrestris]
Unigene11598	NO	PREDICTED: group XIIA secretory phospholipase A2-like [Apis florea]
Unigene11613	NO	PREDICTED: calcium-independent phospholipase A2-gamma-like [Bombus terrestris]
Unigene11645	NO	putative ETS-like protein pointed, isoform P1 [Danaus plexippus]
Unigene11659	NO	GTP-binding protein (i) alpha subunit, gnai [Aedes aegypti]
Unigene11690	NO	PREDICTED: protein son of sevenless-like [Megachile rotundata]
Unigene11735	NO	PREDICTED: eukaryotic translation initiation factor 2-alpha kinase-like [Bombus terrestris]
Unigene11921	NO	PREDICTED: TATA-box-binding protein-like [Bombus terrestris]
Unigene11930	NO	PREDICTED: cell division cycle protein 16 homolog [Megachile rotundata]
Unigene11985	NO	PREDICTED: hypothetical protein LOC408354 [Apis mellifera]
Unigene11993	NO	Mitogen-activated protein kinase kinase kinase 4 [Harpegnathos saltator]
Unigene12096	NO	PREDICTED: protein VAC14 homolog [Megachile rotundata]
Unigene1213	NO	PREDICTED: calcineurin subunit B type 2-like isoform 1 [Nasonia vitripennis]
Unigene12162	NO	PREDICTED: disintegrin and metalloproteinase domain-containing protein 10-like [Megachile rotundata]
Unigene12247	NO	PREDICTED: hypothetical protein LOC100743521 [Bombus impatiens]
Unigene1263	NO	PREDICTED: microtubule-associated protein tau-like [Apis florea]
Unigene12641	NO	PREDICTED: LOW QUALITY PROTEIN: phospholipase D1-like [Bombus terrestris]
Unigene12735	NO	PREDICTED: armadillo segment polarity protein isoform 2 [Nasonia vitripennis]
Unigene12807	NO	PREDICTED: disintegrin and metalloproteinase domain-containing protein 10-like [Megachile rotundata]
Unigene1286	NO	ADP/ATP translocase [Helicoverpa armigera]
Unigene1310	NO	PREDICTED: troponin C, isoform 3 isoform 1 [Nasonia vitripennis]
Unigene13136	NO	hypothetical protein SINV_10030 [Solenopsis invicta]
Unigene13138	NO	PREDICTED: segment polarity protein dishevelled homolog DVL-3-like [Megachile rotundata]
Unigene13165	NO	PREDICTED: alpha-actinin, sarcomeric-like [Megachile rotundata]
Unigene13191	NO	PREDICTED: cyclic AMP-dependent transcription factor ATF-2-like [Apis mellifera]
Unigene13259	NO	PREDICTED: phospholipase D2-like [Apis florea]
Unigene13515	NO	PREDICTED: TATA-box-binding protein-like [Megachile rotundata]
Unigene13553	NO	PREDICTED: protein BCL9 homolog [Apis florea]
Unigene13736	NO	hypothetical protein KGM_03021 [Danaus plexippus]
Unigene1437	NO	PREDICTED: translocator protein-like [Bombus impatiens]
Unigene1446	NO	PREDICTED: proto-oncogene c-Fos-like [Bombus terrestris]
Unigene1513	NO	conserved hypothetical protein [Pediculus humanus corporis]
Unigene1630	NO	small heat shock protein [Pteromalus puparum]
Unigene1746	NO	TFIID [Acanthamoeba castellanii]
Unigene1894	NO	Ras-like GTP-binding protein Rho1 [Salmo salar]
Unigene1925	NO	PREDICTED: cdc42 homolog isoform 1 [Nasonia vitripennis]
Unigene1927	NO	PREDICTED: SH3 domain-binding glutamic acid-rich protein homolog [Megachile rotundata]
Unigene2017	NO	hypothetical protein AaeL_AAEL006826 [Aedes aegypti]
Unigene2059	NO	budding uninhibited by benzimidazoles 3 [Tribolium castaneum]

**Cell proliferation**	Unigene2069	NO	PREDICTED: ras-like protein 1-like [Apis florea]
	Unigene2152	NO	Guanine nucleotide-binding protein subunit gamma-1 [Camponotus floridanus]
	Unigene2158	NO	PREDICTED: WD repeat-containing protein 55 homolog [Nasonia vitripennis]
	Unigene2196	NO	PREDICTED: serine/threonine-protein kinase grp isoform 2 [Apis mellifera]
	Unigene2210	NO	PREDICTED: signal transducer and activator of transcription 5B-like [Megachile rotundata]
	Unigene2365	NO	PREDICTED: ran-specific GTPase-activating protein-like [Bombus impatiens]
	Unigene2418	NO	PREDICTED: RAC serine/threonine-protein kinase-like [Bombus impatiens]
	Unigene25	NO	PREDICTED: ras-like protein 2-like [Nasonia vitripennis]
	Unigene2504	NO	hypothetical protein TRIADDRAFT_20496 [Trichoplax adhaerens]
	Unigene2544	NO	PREDICTED: uncharacterized protein LOC100879259 [Megachile rotundata]
	Unigene2630	NO	PREDICTED: ribosomal protein S6 kinase beta-1-like [Apis florea]
	Unigene2925	NO	PREDICTED: hypothetical protein LOC409983 [Apis mellifera]
	Unigene2926	NO	PREDICTED: calcium-transporting ATPase sarcoplasmic/endoplasmic reticulum type-like [Megachile rotundata]
	Unigene2956	NO	hypothetical protein SINV_02345 [Solenopsis invicta]
	Unigene2992	NO	Exportin-1 [Harpegnathos saltator]
	Unigene3005	NO	PREDICTED: calumenin-like [Nasonia vitripennis]
	Unigene3137	NO	PREDICTED: ran-specific GTPase-activating protein-like [Apis mellifera]
	Unigene3146	NO	Ran-binding protein 9 [Camponotus floridanus]
	Unigene3188	NO	PREDICTED: serine/threonine-protein phosphatase 2A catalytic subunit beta isoform-like [Amphimedon queenslandica]
	Unigene3316	NO	PREDICTED: calcium-binding protein p22-like [Megachile rotundata]
	Unigene3344	NO	PREDICTED: solute carrier family 2, facilitated glucose transporter member 1-like [Nasonia vitripennis]
	Unigene3376	NO	PREDICTED: hypothetical protein LOC100745000 [Bombus impatiens]
	Unigene3407	NO	PREDICTED: sideroflexin-1-like isoform 2 [Nasonia vitripennis]
	Unigene3519	NO	PREDICTED: uncharacterized protein LOC100867253 [Apis florea]
	Unigene368	NO	PREDICTED: ADP,ATP carrier protein 2-like [Bombus impatiens]
	Unigene3745	NO	PREDICTED: protein Dr1-like [Megachile rotundata]
	Unigene3784	NO	Ran-binding protein 3 [Camponotus floridanus]
	Unigene3817	NO	EH domain-binding protein 1 [Camponotus floridanus]
	Unigene383	NO	PREDICTED: cytochrome c-like isoform 1 [Bombus terrestris]
	Unigene3861	NO	PREDICTED: group XIIA secretory phospholipase A2-like [Nasonia vitripennis]
	Unigene3866	NO	hypothetical protein EAI_17259 [Harpegnathos saltator]
	Unigene3960	NO	PREDICTED: myb-related protein A-like [Nasonia vitripennis]
	Unigene4	NO	hypothetical protein KGM_05418 [Danaus plexippus]
	Unigene4045	NO	Dual specificity mitogen-activated protein kinase kinase 4 [Acromyrmex echinatior]
	Unigene4101	NO	hypothetical protein SINV_05984 [Solenopsis invicta]
	Unigene4136	NO	Myb protein [Camponotus floridanus]
	Unigene4193	NO	hypothetical protein SINV_10623 [Solenopsis invicta]
	Unigene4353	NO	RING finger protein unkempt-like protein [Harpegnathos saltator]
	Unigene4470	NO	Ras-like GTP-binding protein RHO [Lepeophtheirus salmonis]
	Unigene4485	NO	PREDICTED: LOW QUALITY PROTEIN: talin-1-like [Megachile rotundata]
	Unigene452	NO	PREDICTED: activating transcription factor of chaperone-like [Megachile rotundata]
	Unigene4604	NO	hypothetical protein KGM_05418 [Danaus plexippus]
	Unigene4655	NO	PREDICTED: transcription factor E2F3-like isoform 1 [Bombus terrestris]
	Unigene466	NO	PREDICTED: hypothetical protein LOC100747421 [Bombus impatiens]
	Unigene4710	NO	Dual specificity mitogen-activated protein kinase kinase 4 [Acromyrmex echinatior]
	Unigene4742	NO	PREDICTED: calmodulin-like protein 4-like isoform 1 [Bombus terrestris]
	Unigene4774	NO	mitochondrial cytochrome c [Bombyx mori]
	Unigene485	NO	AT15141p [Drosophila melanogaster]
	Unigene4903	NO	putative voltage-dependent anion-selective channel isoform 1 [Danaus plexippus]
	Unigene4920	NO	PREDICTED: mitogen-activated protein kinase 1-like [Megachile rotundata]
	Unigene4950	NO	PREDICTED: dual specificity mitogen-activated protein kinase kinase 6-like [Megachile rotundata]
	Unigene4973	NO	serine/threonine-protein kinase grp-like [Apis mellifera]
	Unigene5020	NO	hypothetical protein AND_02161 [Anopheles darlingi]
	Unigene5047	NO	PREDICTED: NFU1 iron-sulfur cluster scaffold homolog, mitochondrial-like [Bombus terrestris]
	Unigene514	NO	PREDICTED: serine/threonine-protein kinase KDX1-like [Bombus impatiens]
	Unigene5171	NO	PREDICTED: talin-2-like [Bombus terrestris]
	Unigene5212	NO	Proliferating cell nuclear antigen [Camponotus floridanus]
	Unigene5357	NO	PREDICTED: exportin-1-like [Nasonia vitripennis]
	Unigene5446	NO	PREDICTED: G1/S-specific cyclin-D2-like [Megachile rotundata]
	Unigene5515	NO	PREDICTED: armadillo segment polarity protein-like [Megachile rotundata]

Unigene5593	NO	PREDICTED: hypothetical protein LOC100745810 isoform 2 [Bombus impatiens]
Unigene5691	NO	PREDICTED: hypothetical protein LOC412916 [Apis mellifera]
Unigene5806	NO	PREDICTED: uncharacterized protein LOC100878829 [Megachile rotundata]
Unigene6140	NO	Ras-like GTP-binding protein Rho1 [Danaus plexippus]
Unigene6195	NO	PREDICTED: dual specificity protein phosphatase Mpk3-like [Bombus impatiens]
Unigene626	NO	Voltage-dependent anion-selective channel [Acromyrmex echinatior]
Unigene6295	NO	PREDICTED: dual specificity mitogen-activated protein kinase kinase hemipterous-like isoform 2 [Megachile rotundata]
Unigene6363	NO	Mitogen-activated protein kinase 1 [Harpegnathos saltator]
Unigene6394	NO	Talin-1 [Camponotus floridanus]
Unigene6671	NO	Ras-like GTP-binding protein RHO [Lepeophtheirus salmonis]
Unigene669	NO	PREDICTED: protein enhancer of sevenless 2B-like [Nasonia vitripennis]
Unigene6700	NO	Talin-1 [Camponotus floridanus]
Unigene6735	NO	hypothetical protein SINV_14598 [Solenopsis invicta]
Unigene6842	NO	guanine nucleotide binding protein alpha q polypeptide [Glossina morsitans morsitans]
Unigene6899	NO	PREDICTED: proliferating cell nuclear antigen-like [Nasonia vitripennis]
Unigene6948	NO	PREDICTED: anaphase-promoting complex subunit CDC26-like isoform 1 [Apis florea]
Unigene698	NO	ADP/ATP translocase [Manduca sexta]
Unigene6991	NO	PREDICTED: anaphase-promoting complex subunit 10-like [Megachile rotundata]
Unigene7027	NO	AGAP005160-PA [Anopheles gambiae str. PEST]
Unigene7044	NO	PREDICTED: cell division cycle protein 20 homolog [Canis lupus familiaris]
Unigene7068	NO	PREDICTED: nuclear transcription factor Y subunit beta-like [Bombus impatiens]
Unigene71	NO	hypothetical protein EAI_14262 [Harpegnathos saltator]
Unigene7102	NO	Multiple C2 and transmembrane domain-containing protein 2 [Acromyrmex echinatior]
Unigene7161	NO	PREDICTED: sideroflexin-1-like isoform 2 [Nasonia vitripennis]
Unigene7180	NO	39S ribosomal protein L46, mitochondrial [Acromyrmex echinatior]
Unigene722	NO	PREDICTED: ras-like GTP-binding protein Rho1 [Nasonia vitripennis]
Unigene7225	NO	PREDICTED: hypothetical protein LOC408577 [Apis mellifera]
Unigene7237	NO	PREDICTED: hypothetical protein LOC100747412 isoform 2 [Bombus impatiens]
Unigene7340	NO	hypothetical protein SINV_10840 [Solenopsis invicta]
Unigene7464	NO	Eukaryotic translation initiation factor 2-alpha kinase [Acromyrmex echinatior]
Unigene7498	NO	hypothetical protein SINV_12469 [Solenopsis invicta]
Unigene7595	NO	PREDICTED: anaphase-promoting complex subunit 11-like [Apis mellifera]
Unigene7655	NO	PREDICTED: cell division cycle protein 27 homolog [Megachile rotundata]
Unigene7714	NO	Mitogen-activated protein kinase 14B [Harpegnathos saltator]
Unigene7739	NO	control protein HCTL024 [Heliconius hortense]
Unigene7754	NO	PREDICTED: cyclin-dependent kinase 6-like [Apis florea]
Unigene776	NO	AT15141p [Drosophila melanogaster]
Unigene7857	NO	hypothetical protein SINV_10861 [Solenopsis invicta]
Unigene7916	NO	Dual specificity mitogen-activated protein kinase kinase 7 [Harpegnathos saltator]
Unigene7980	NO	PREDICTED: dual specificity mitogen-activated protein kinase kinase 2-like [Apis florea]
Unigene8035	NO	PREDICTED: guanine nucleotide-binding protein G(s) subunit alpha-like [Megachile rotundata]
Unigene8049	NO	Talin-1 [Camponotus floridanus]
Unigene8102	NO	hypothetical protein SINV_10861 [Solenopsis invicta]
Unigene8266	NO	PREDICTED: mothers against decapentaplegic homolog 3 [Nasonia vitripennis]
Unigene8344	NO	PREDICTED: phosphatidylinositol 3-kinase regulatory subunit alpha-like, partial [Apis florea]
Unigene8372	NO	PREDICTED: baculoviral IAP repeat-containing protein 3-like [Nasonia vitripennis]
Unigene8408	NO	putative laminin A chain [Danaus plexippus]
Unigene8488	NO	PREDICTED: uncharacterized protein LOC100877711 [Megachile rotundata]
Unigene8515	NO	PREDICTED: hypothetical protein LOC100642907 [Bombus terrestris]
Unigene8610	NO	PREDICTED: talin-1-like [Apis mellifera]
Unigene8755	NO	PREDICTED: LOW QUALITY PROTEIN: ribosomal protein S6 kinase beta-1-like [Apis florea]
Unigene8759	NO	Phospholipase D1 [Harpegnathos saltator]
Unigene8772	NO	putative collagen alpha-2IV chain protein [Danaus plexippus]
Unigene9008	NO	PREDICTED: guanine nucleotide-binding protein G(q) subunit alpha-like isoform 5 [Megachile rotundata]
Unigene905	NO	PREDICTED: transcription factor AP-1-like [Megachile rotundata]
Unigene9081	NO	putative laminin A chain [Danaus plexippus]
Unigene9217	NO	PREDICTED: stress-activated protein kinase JNK-like [Megachile rotundata]
Unigene925	NO	PREDICTED: Golgi reassembly-stacking protein 1-like [Apis florea]

	Unigene9259	NO	PREDICTED: calcium/calmodulin-dependent protein kinase type II alpha chain-like isoform 1 [Bombus impatiens]
	Unigene9292	NO	PREDICTED: LOW QUALITY PROTEIN: talin-2-like [Apis florea]
	Unigene9328	NO	PREDICTED: LOW QUALITY PROTEIN: talin-1-like [Megachile rotundata]
	Unigene934	NO	PREDICTED: farnesyl pyrophosphate synthase-like [Nasonia vitripennis]
	Unigene9359	NO	PREDICTED: hypothetical protein LOC100743083 [Bombus impatiens]
	Unigene9378	NO	PREDICTED: armadillo segment polarity protein-like [Megachile rotundata]
	Unigene9415	NO	PREDICTED: mitogen-activated protein kinase 14B-like isoform 2 [Megachile rotundata]
	Unigene9499	NO	hypothetical protein KGM_05418 [Danaus plexippus]
	Unigene9582	NO	PREDICTED: talin-2-like [Bombus impatiens]
	Unigene9653	NO	PREDICTED: guanine nucleotide-binding protein G(s) subunit alpha-like [Megachile rotundata]
	Unigene9659	NO	sarco/endoplasmic reticulum calcium ATPase [Bombyx mori]
	Unigene9782	NO	Segment polarity protein dishevelled-like protein DVL-3 [Harpegnathos saltator]
	Unigene9797	NO	PREDICTED: alpha-actinin, sarcomeric-like [Megachile rotundata]
	Unigene9818	NO	PREDICTED: uncharacterized protein LOC100875893 [Megachile rotundata]
	Unigene9998	NO	PREDICTED: stress-activated protein kinase JNK-like [Megachile rotundata]
	Unigene1289	SP	cytochrome oxidase subunit I [Cotesia chilonis]
	Unigene244	SP	calreticulin [Cotesia rubecula]
	Unigene758	SP	Protein toll [Harpegnathos saltator]
	CL205.Contig1	NO	PREDICTED: NADH dehydrogenase [ubiquinone] flavoprotein 1, mitochondrial-like [Apis florea]
	CL205.Contig2	NO	PREDICTED: NADH dehydrogenase [ubiquinone] flavoprotein 1, mitochondrial [Apis mellifera]
	CL231.Contig1	NO	ATP synthase subunit alpha, mitochondrial [Harpegnathos saltator]
	CL352.Contig1	NO	cytochrome c oxidase subunit I [Cotesia vestalis]
	CL352.Contig2	NO	cytochrome oxidase subunit I [Bucculatrix artemisiella]
	CL352.Contig3	NO	cytochrome c oxidase subunit I [Cotesia vestalis]
	CL374.Contig1	NO	ATP synthase subunit beta, mitochondrial [Harpegnathos saltator]
	CL374.Contig2	NO	ATP-synthase subunit beta [Schistocerca gregaria]
	CL374.Contig3	NO	ATP synthase subunit beta, putative [Pediculus humanus corporis]
	CL401.Contig2	NO	PREDICTED: ATP synthase subunit epsilon, mitochondrial-like isoform 1 [Apis mellifera]
	CL560.Contig1	NO	cytochrome b [Cotesia vestalis]
	CL597.Contig2	NO	cytochrome b [Cotesia vestalis]
	CL663.Contig1	NO	Serine/threonine-protein phosphatase 2A 65 kDa regulatory subunit A alpha isoform [Camponotus floridanus]
	CL663.Contig2	NO	Serine/threonine-protein phosphatase 2A 65 kDa regulatory subunit A alpha isoform [Camponotus floridanus]
	CL740.Contig1	NO	PREDICTED: NADH dehydrogenase [ubiquinone] flavoprotein 1, mitochondrial-like [Megachile rotundata]
	CL750.Contig1	NO	PREDICTED: ATP synthase lipid-binding protein, mitochondrial-like [Megachile rotundata]
	Unigene1004	NO	hypothetical protein SINV_14598 [Solenopsis invicta]
	Unigene1012	NO	Cytochrome b-c1 complex subunit 2, mitochondrial [Camponotus floridanus]
	Unigene1014	NO	PREDICTED: NADH dehydrogenase [ubiquinone] iron-sulfur protein 7, mitochondrial-like [Apis florea]
	Unigene10363	NO	GTP-binding protein alpha subunit, gna [Culex quinquefasciatus]
	Unigene10410	NO	Mothers against decapentaplegic-like protein 3 [Acromyrmex echinatior]
	Unigene1043	NO	PREDICTED: cytochrome c oxidase subunit 6C-like [Acyrthosiphon pisum]
	Unigene1044	NO	GF11341 [Drosophila ananassae]
	Unigene10590	NO	hypothetical protein KGM_04009 [Danaus plexippus]
	Unigene1074	NO	NADH dehydrogenase [ubiquinone] 1 alpha subcomplex subunit 13 [Camponotus floridanus]
	Unigene1090	NO	PREDICTED: similar to NADH:ubiquinone dehydrogenase, putative [Tribolium castaneum]
	Unigene11062	NO	Stress-activated protein kinase JNK [Acromyrmex echinatior]
	Unigene11087	NO	PREDICTED: guanine nucleotide-binding protein G(i) subunit alpha-like [Megachile rotundata]
	Unigene11213	NO	Phosphatidylinositol 3-kinase regulatory subunit alpha [Camponotus floridanus]
	Unigene11447	NO	hypothetical protein SINV_14862 [Solenopsis invicta]
	Unigene11659	NO	GTP-binding protein (i) alpha subunit, gnai [Aedes aegypti]
	Unigene11731	NO	GE14316 [Drosophila yakuba]
	Unigene11808	NO	cytochrome c oxidase subunit II [Cotesia vestalis]
	Unigene12247	NO	PREDICTED: hypothetical protein LOC100743521 [Bombus impatiens]

	Unigene1243	NO	GI19985 [Drosophila mojavensis]
	Unigene1286	NO	ADP/ATP translocase [Helicoverpa armigera]
	Unigene1290	NO	PREDICTED: succinate dehydrogenase [ubiquinone] flavoprotein subunit, mitochondrial-like [Megachile rotundata]
	Unigene1327	NO	hypothetical protein SINV_09347 [Solenopsis invicta]
	Unigene13656	NO	cytochrome oxidase subunit II [Xyrosaris lichneuta]
	Unigene1371	NO	NADH dehydrogenase [ubiquinone] 1 beta subcomplex subunit 8, mitochondrial [Nasonia vitripennis]
	Unigene1404	NO	hypothetical protein AND_29000 [Anopheles darlingi]
	Unigene1440	NO	NADH dehydrogenase (ubiquinone) 1 beta subcomplex, 7 [Tribolium castaneum]
	Unigene1447	NO	NADH dehydrogenase [ubiquinone] iron-sulfur protein 6, mitochondrial [Nasonia vitripennis]
	Unigene1546	NO	PREDICTED: hypothetical protein LOC100119331 [Nasonia vitripennis]
	Unigene1574	NO	AGAP009602-PA [Anopheles gambiae str. PEST]
	Unigene1611	NO	PREDICTED: probable NADH dehydrogenase [ubiquinone] 1 alpha subcomplex subunit 12-like [Megachile rotundata]
	Unigene1633	NO	NADH dehydrogenase [ubiquinone] iron-sulfur protein 8, mitochondrial [Nasonia vitripennis]
	Unigene1646	NO	NADH dehydrogenase [Culex quinquefasciatus]
	Unigene1653	NO	AGAP012374-PA [Anopheles gambiae str. PEST]
	Unigene1688	NO	GD20916 [Drosophila simulans]
	Unigene1736	NO	AGAP009824-PA [Anopheles gambiae str. PEST]
	Unigene1738	NO	succinate dehydrogenase iron sulfur subunit B [Lysiphlebus testaceipes]
	Unigene1749	NO	NADH dehydrogenase [ubiquinone] 1 beta subcomplex subunit 2, mitochondrial [Camponotus floridanus]
	Unigene1800	NO	NADH dehydrogenase (ubiquinone) 1 alpha subcomplex, 9, 39kDa [Nasonia vitripennis]
	Unigene1833	NO	NADH dehydrogenase [ubiquinone] 1 alpha subcomplex subunit 2 [Camponotus floridanus]
	Unigene2076	NO	--
	Unigene2158	NO	PREDICTED: WD repeat-containing protein 55 homolog [Nasonia vitripennis]
	Unigene2224	NO	NADH dehydrogenase [Lysiphlebus testaceipes]
	Unigene2244	NO	AGAP006918-PA [Anopheles gambiae str. PEST]
	Unigene2346	NO	NADH dehydrogenase [ubiquinone] flavoprotein 2, mitochondrial [Nasonia vitripennis]
	Unigene2418	NO	PREDICTED: RAC serine/threonine-protein kinase-like [Bombus impatiens]
	Unigene2537	NO	ATP synthase subunit c [Manduca sexta]
	Unigene2697	NO	cytochrome c oxidase polypeptide Vb [Bombyx mori]
	Unigene2856	NO	succinate dehydrogenase [ubiquinone] cytochrome b small subunit, mitochondrial [Nasonia vitripennis]
	Unigene2903	NO	hypothetical protein KGM_05439 [Danaus plexippus]
	Unigene3188	NO	PREDICTED: serine/threonine-protein phosphatase 2A catalytic subunit beta isoform-like [Amphimedon queenslandica]
	Unigene3405	NO	ATP synthase [Bombyx mori]
	Unigene346	NO	GJ13165 [Drosophila virilis]
	Unigene368	NO	PREDICTED: ADP,ATP carrier protein 2-like [Bombus impatiens]
	Unigene372	NO	PREDICTED: hypothetical protein LOC100115623 isoform 1 [Nasonia vitripennis]
	Unigene383	NO	PREDICTED: cytochrome c-like isoform 1 [Bombus terrestris]
	Unigene3882	NO	PREDICTED: NADH dehydrogenase [ubiquinone] iron-sulfur protein 4, mitochondrial-like [Megachile rotundata]
**Cell death**	Unigene4045	NO	Dual specificity mitogen-activated protein kinase kinase 4 [Acromyrmex echinatior]
	Unigene4061	NO	hypothetical protein AND_01489 [Anopheles darlingi]
	Unigene4072	NO	hypothetical protein KGM_03909 [Danaus plexippus]
	Unigene4107	NO	hypothetical protein SINV_09114 [Solenopsis invicta]
	Unigene411	NO	cytochrome c oxidase subunit VI [Microplitis mediator]
	Unigene4122	NO	mitochondrial cytochrome c oxidase subunit VIa [Bombyx mori]
	Unigene426	NO	cytochrome c oxidase-like protein [Glyptapanteles flavicoxis]
	Unigene4333	NO	H+ transporting ATP synthase gamma subunit [Danaus plexippus]
	Unigene438	NO	cytochrome b-c1 complex subunit 9 [Nasonia vitripennis]
	Unigene4535	NO	hypothetical protein SINV_09114 [Solenopsis invicta]
	Unigene4584	NO	PREDICTED: NADH-ubiquinone oxidoreductase 75 kDa subunit, mitochondrial-like [Bombus terrestris]
	Unigene467	NO	hypothetical protein SINV_11135 [Solenopsis invicta]
	Unigene4710	NO	Dual specificity mitogen-activated protein kinase kinase 4 [Acromyrmex echinatior]
	Unigene4774	NO	mitochondrial cytochrome c [Bombyx mori]
	Unigene4852	NO	hypothetical protein KGM_08437 [Danaus plexippus]
	Unigene4885	NO	ubiquinol-cytochrome C reductase complex protein [Danaus plexippus]

Unigene4903	NO	putative voltage-dependent anion-selective channel isoform 1 [Danaus plexippus]
Unigene4920	NO	PREDICTED: mitogen-activated protein kinase 1-like [Megachile rotundata]
Unigene4959	NO	ATP synthase [Danaus plexippus]
Unigene5047	NO	PREDICTED: NFU1 iron-sulfur cluster scaffold homolog, mitochondrial-like [Bombus terrestris]
Unigene5072	NO	putative NADH:ubiquinone dehydrogenase [Danaus plexippus]
Unigene520	NO	cytochrome c oxidase subunit 4 isoform 1, mitochondrial [Nasonia vitripennis]
Unigene533	NO	ATP synthase subunit b, mitochondrial [Acromyrmex echinatior]
Unigene5492	NO	hypothetical protein KGM_00095 [Danaus plexippus]
Unigene5526	NO	PREDICTED: protein toll [Nasonia vitripennis]
Unigene553	NO	PREDICTED: cytochrome b-c1 complex subunit 6, mitochondrial-like [Megachile rotundata]
Unigene558	NO	AGAP008724-PA [Anopheles gambiae str. PEST]
Unigene564	NO	PREDICTED: similar to mitochondrial ATP synthase coupling factor 6 [Tribolium castaneum]
Unigene579	NO	cytochrome c oxidase subunit VIb isoform 1 [Rhipicephalus sanguineus]
Unigene6060	NO	lethal neo18 protein [Danaus plexippus]
Unigene608	NO	hypothetical protein EAG_05916 [Camponotus floridanus]
Unigene614	NO	Cytochrome b-c1 complex subunit Rieske, mitochondrial [Camponotus floridanus]
Unigene624	NO	ATP synthase subunit gamma, mitochondrial [Nasonia vitripennis]
Unigene626	NO	Voltage-dependent anion-selective channel [Acromyrmex echinatior]
Unigene6363	NO	Mitogen-activated protein kinase 1 [Harpegnathos saltator]
Unigene647	NO	PREDICTED: cytochrome b-c1 complex subunit Rieske, mitochondrial-like [Cavia porcellus]
Unigene663	NO	Cytochrome b-c1 complex subunit 7 [Camponotus floridanus]
Unigene6630	NO	cytochrome c oxidase [Danaus plexippus]
Unigene6642	NO	NADH dehydrogenase [Danaus plexippus]
Unigene6695	NO	unnamed protein product [Heliconius melpomene]
Unigene6735	NO	hypothetical protein SINV_14598 [Solenopsis invicta]
Unigene6842	NO	guanine nucleotide binding protein alpha q polypeptide [Glossina morsitans morsitans]
Unigene6884	NO	H+ transporting ATP synthase O subunit isoform 1 [Bombyx mori]
Unigene694	NO	ATP synthase subunit O, mitochondrial [Camponotus floridanus]
Unigene698	NO	ADP/ATP translocase [Manduca sexta]
Unigene7100	NO	cytochrome c oxidase polypeptide IV [Bombyx mori]
Unigene7225	NO	PREDICTED: hypothetical protein LOC408577 [Apis mellifera]
Unigene731	NO	hypothetical protein SINV_08923 [Solenopsis invicta]
Unigene743	NO	GF11012 [Drosophila ananassae]
Unigene7469	NO	NADH-ubiquinone oxidoreductase 75 kDa subunit, mitochondrial precursor [Nasonia vitripennis]
Unigene763	NO	hypothetical protein SINV_15147 [Solenopsis invicta]
Unigene7714	NO	Mitogen-activated protein kinase 14B [Harpegnathos saltator]
Unigene7739	NO	control protein HCTL024 [Heliconius hortense]
Unigene787	NO	NADH dehydrogenase [ubiquinone] 1 alpha subcomplex subunit 6 [Nasonia vitripennis]
Unigene7938	NO	hypothetical protein KGM_04538 [Danaus plexippus]
Unigene799	NO	hypothetical protein SINV_06969 [Solenopsis invicta]
Unigene8035	NO	PREDICTED: guanine nucleotide-binding protein G(s) subunit alpha-like [Megachile rotundata]
Unigene8104	NO	NADH dehydrogenase [Danaus plexippus]
Unigene8196	NO	cytochrome c oxidase subunit III [Cotesia vestalis]
Unigene8222	NO	ubiquinol-cytochrome C reductase complex 14kD subunit [Danaus plexippus]
Unigene8266	NO	PREDICTED: mothers against decapentaplegic homolog 3 [Nasonia vitripennis]
Unigene829	NO	ATP synthase subunit d, mitochondrial [Camponotus floridanus]
Unigene8339	NO	PREDICTED: hypothetical protein LOC100743562 isoform 2 [Bombus impatiens]
Unigene8344	NO	PREDICTED: phosphatidylinositol 3-kinase regulatory subunit alpha-like, partial [Apis florea]
Unigene837	NO	hypothetical protein AND_29231 [Anopheles darlingi]
Unigene8416	NO	H+ transporting ATP synthase O subunit isoform 1 [Bombyx mori]
Unigene8488	NO	PREDICTED: uncharacterized protein LOC100877711 [Megachile rotundata]
Unigene8922	NO	mitochondrial ATP synthase coupling factor [Antheraea yamamai]
Unigene9008	NO	PREDICTED: guanine nucleotide-binding protein G(q) subunit alpha-like isoform 5 [Megachile rotundata]
Unigene905	NO	PREDICTED: transcription factor AP-1-like [Megachile rotundata]
Unigene912	NO	cytochrome c1, heme protein, mitochondrial [Nasonia vitripennis]
Unigene9141	NO	PREDICTED: hypothetical protein LOC100743562 isoform 1 [Bombus impatiens]
Unigene9217	NO	PREDICTED: stress-activated protein kinase JNK-like [Megachile rotundata]
Unigene9359	NO	PREDICTED: hypothetical protein LOC100743083 [Bombus impatiens]

	Unigene9415	NO	PREDICTED: mitogen-activated protein kinase 14B-like isoform 2 [Megachile rotundata]
	Unigene9462	NO	NADH dehydrogenase ubiquinone Fe-S 8 [Bombyx mori]
	Unigene9485	NO	H+ transporting ATP synthase O subunit isoform 1 [Danaus plexippus]
	Unigene952	NO	PREDICTED: acyl carrier protein, mitochondrial-like [Megachile rotundata]
	Unigene959	NO	NADH dehydrogenase [ubiquinone] 1 beta subcomplex subunit 5, mitochondrial [Nasonia vitripennis]
	Unigene9653	NO	PREDICTED: guanine nucleotide-binding protein G(s) subunit alpha-like [Megachile rotundata]
	Unigene991	NO	PREDICTED: NADH dehydrogenase [ubiquinone] 1 alpha subcomplex subunit 7-like [Megachile rotundata]
	Unigene9927	NO	Subunit VIb of cytochrome c oxidase [Danaus plexippus]
	Unigene9971	NO	hypothetical protein KGM_15724 [Danaus plexippus]
	Unigene9998	NO	PREDICTED: stress-activated protein kinase JNK-like [Megachile rotundata]
	CL124.Contig1	NO	succinate dehydrogenase cytochrome b560 subunit, mitochondrial [Nasonia vitripennis]
**Ion channel regulation**	CL375.Contig1	NO	guanine nucleotide-binding protein subunit beta-like [Microplitis mediator]
	CL375.Contig2	NO	guanine nucleotide-binding protein subunit beta-like [Microplitis mediator]
	CL417.Contig1	NO	PREDICTED: dual specificity protein phosphatase 10-like [Bombus terrestris]
	CL417.Contig2	NO	PREDICTED: dual specificity protein phosphatase 10-like [Megachile rotundata]
	CL724.Contig1	NO	PREDICTED: dual specificity protein phosphatase Mpk3-like [Megachile rotundata]
	CL724.Contig2	NO	PREDICTED: dual specificity protein phosphatase Mpk3-like [Megachile rotundata]
	Unigene10095	NO	dopa decarboxylase [Papilio machaon]
	Unigene10121	NO	85 kDa calcium-independent phospholipase A2 [Camponotus floridanus]
	Unigene10184	NO	hypothetical protein DAPPUDRAFT_304184 [Daphnia pulex]
	Unigene10363	NO	GTP-binding protein alpha subunit, gna [Culex quinquefasciatus]
	Unigene10531	NO	phospholipase A2 precursor [Apis mellifera]
	Unigene11077	NO	PREDICTED: dual specificity protein phosphatase Mpk3-like [Megachile rotundata]
	Unigene11087	NO	PREDICTED: guanine nucleotide-binding protein G(i) subunit alpha-like [Megachile rotundata]
	Unigene11455	NO	PREDICTED: serine/threonine/tyrosine-interacting protein-like [Bombus impatiens]
	Unigene11598	NO	PREDICTED: group XIIA secretory phospholipase A2-like [Apis florea]
	Unigene11613	NO	PREDICTED: calcium-independent phospholipase A2-gamma-like [Bombus terrestris]
	Unigene11659	NO	GTP-binding protein (i) alpha subunit, gnai [Aedes aegypti]
	Unigene13476	NO	Aromatic-L-amino-acid decarboxylase [Acromyrmex echinatior]
	Unigene13635	NO	PREDICTED: LOW QUALITY PROTEIN: BTB/POZ domain-containing protein KCTD3-like [Nasonia vitripennis]
	Unigene1927	NO	PREDICTED: SH3 domain-binding glutamic acid-rich protein homolog [Megachile rotundata]
	Unigene2017	NO	hypothetical protein AaeL_AAEL006826 [Aedes aegypti]
	Unigene2069	NO	PREDICTED: ras-like protein 1-like [Apis florea]
	Unigene2152	NO	Guanine nucleotide-binding protein subunit gamma-1 [Camponotus floridanus]
	Unigene2339	NO	Guanine nucleotide-binding protein subunit gamma-e [Harpegnathos saltator]
	Unigene2474	NO	dopa decarboxylase [Mythimna separata]
	Unigene2504	NO	hypothetical protein TRIADDRAFT_20496 [Trichoplax adhaerens]
	Unigene3861	NO	PREDICTED: group XIIA secretory phospholipase A2-like [Nasonia vitripennis]
	Unigene4101	NO	hypothetical protein SINV_05984 [Solenopsis invicta]
	Unigene4920	NO	PREDICTED: mitogen-activated protein kinase 1-like [Megachile rotundata]
	Unigene6195	NO	PREDICTED: dual specificity protein phosphatase Mpk3-like [Bombus impatiens]
	Unigene6363	NO	Mitogen-activated protein kinase 1 [Harpegnathos saltator]
	Unigene6842	NO	guanine nucleotide binding protein alpha q polypeptide [Glossina morsitans morsitans]
	Unigene7102	NO	Multiple C2 and transmembrane domain-containing protein 2 [Acromyrmex echinatior]
	Unigene7980	NO	PREDICTED: dual specificity mitogen-activated protein kinase kinase 2-like [Apis florea]
	Unigene8035	NO	PREDICTED: guanine nucleotide-binding protein G(s) subunit alpha-like [Megachile rotundata]
	Unigene8571	NO	Aromatic-L-amino-acid decarboxylase [Acromyrmex echinatior]
	Unigene9008	NO	PREDICTED: guanine nucleotide-binding protein G(q) subunit alpha-like isoform 5 [Megachile rotundata]
	Unigene9653	NO	PREDICTED: guanine nucleotide-binding protein G(s) subunit alpha-like [Megachile rotundata]
	CL334.Contig1	SP	Calcium-transporting ATPase sarcoplasmic/endoplasmic reticulum type [Acromyrmex echinatior]
	CL430.Contig2	SP	PREDICTED: tenascin-X-like [Nasonia vitripennis]
	CL590.Contig1	SP	PREDICTED: serine/threonine-protein kinase/endoribonuclease IRE1-like [Nasonia vitripennis]
	Unigene1011	SP	PREDICTED: membrane metallo-endopeptidase-like 1-like [Bombus impatiens]

Unigene1289	SP	cytochrome oxidase subunit I [Cotesia chilonis]
Unigene2376	SP	hypothetical protein TcasGA2_TC010888 [Tribolium castaneum]
Unigene2802	SP	PREDICTED: serine/threonine-protein kinase/endoribonuclease IRE1-like [Megachile rotundata]
Unigene3097	SP	PREDICTED: lipase member H-A-like [Apis mellifera]
Unigene3500	SP	PREDICTED: serine/threonine-protein kinase/endoribonuclease IRE1-like [Nasonia vitripennis]
Unigene4476	SP	hypothetical protein SINV_04265 [Solenopsis invicta]
Unigene4608	SP	Serine/threonine-protein kinase/endoribonuclease ire-1 [Camponotus floridanus]
Unigene6037	SP	PREDICTED: serine/threonine-protein kinase/endoribonuclease IRE1-like [Nasonia vitripennis]
Unigene6049	SP	hypothetical protein SINV_07475 [Solenopsis invicta]
CL124.Contig1	NO	succinate dehydrogenase cytochrome b560 subunit, mitochondrial [Nasonia vitripennis]
CL205.Contig1	NO	PREDICTED: NADH dehydrogenase [ubiquinone] flavoprotein 1, mitochondrial-like [Apis florea]
CL205.Contig2	NO	PREDICTED: NADH dehydrogenase [ubiquinone] flavoprotein 1, mitochondrial [Apis mellifera]
CL231.Contig1	NO	ATP synthase subunit alpha, mitochondrial [Harpegnathos saltator]
CL238.Contig1	NO	PREDICTED: protein kinase shaggy-like isoform 2 [Nasonia vitripennis]
CL238.Contig2	NO	PREDICTED: protein kinase shaggy-like isoform 2 [Nasonia vitripennis]
CL238.Contig4	NO	Protein kinase shaggy [Acromyrmex echinatior]
CL352.Contig1	NO	cytochrome c oxidase subunit I [Cotesia vestalis]
CL352.Contig2	NO	cytochrome oxidase subunit I [Bucculatrix artemisiella]
CL352.Contig3	NO	cytochrome c oxidase subunit I [Cotesia vestalis]
CL363.Contig1	NO	PREDICTED: serine/threonine-protein phosphatase 2B catalytic subunit 2-like [Apis florea]
CL363.Contig2	NO	PREDICTED: serine/threonine-protein phosphatase 2B catalytic subunit 2-like [Apis florea]
CL374.Contig1	NO	ATP synthase subunit beta, mitochondrial [Harpegnathos saltator]
CL374.Contig2	NO	ATP-synthase subunit beta [Schistocerca gregaria]
CL374.Contig3	NO	ATP synthase subunit beta, putative [Pediculus humanus corporis]
CL401.Contig2	NO	PREDICTED: ATP synthase subunit epsilon, mitochondrial-like isoform 1 [Apis mellifera]
CL412.Contig1	NO	hypothetical protein SINV_01823 [Solenopsis invicta]
CL421.Contig2	NO	PREDICTED: glyceraldehyde-3-phosphate dehydrogenase 2 [Nasonia vitripennis]
CL483.Contig1	NO	PREDICTED: hypothetical protein LOC100743933 [Bombus impatiens]
CL483.Contig2	NO	Microtubule-associated protein 2 [Harpegnathos saltator]
CL51.Contig1	NO	hypothetical protein SINV_04265 [Solenopsis invicta]
CL560.Contig1	NO	cytochrome b [Cotesia vestalis]
CL597.Contig2	NO	cytochrome b [Cotesia vestalis]
CL740.Contig1	NO	PREDICTED: NADH dehydrogenase [ubiquinone] flavoprotein 1, mitochondrial-like [Megachile rotundata]
CL750.Contig1	NO	PREDICTED: ATP synthase lipid-binding protein, mitochondrial-like [Megachile rotundata]
CL757.Contig2	NO	PREDICTED: uncharacterized protein LOC100876927 [Megachile rotundata]
Unigene1012	NO	Cytochrome b-c1 complex subunit 2, mitochondrial [Camponotus floridanus]
Unigene1014	NO	PREDICTED: NADH dehydrogenase [ubiquinone] iron-sulfur protein 7, mitochondrial-like [Apis florea]
Unigene10363	NO	GTP-binding protein alpha subunit, gna [Culex quinquefasciatus]
Unigene1043	NO	PREDICTED: cytochrome c oxidase subunit 6C-like [Acyrthosiphon pisum]
Unigene1044	NO	GF11341 [Drosophila ananassae]
Unigene10590	NO	hypothetical protein KGM_04009 [Danaus plexippus]
Unigene1074	NO	NADH dehydrogenase [ubiquinone] 1 alpha subcomplex subunit 13 [Camponotus floridanus]
Unigene10847	NO	PREDICTED: nicastrin-like [Megachile rotundata]
Unigene1090	NO	PREDICTED: similar to NADH:ubiquinone dehydrogenase, putative [Tribolium castaneum]
Unigene11062	NO	Stress-activated protein kinase JNK [Acromyrmex echinatior]
Unigene11124	NO	Presenilin-like protein [Harpegnathos saltator]
Unigene11156	NO	PREDICTED: nicastrin [Apis mellifera]
Unigene1120	NO	PREDICTED: endothelin-converting enzyme 1-like [Nasonia vitripennis]
Unigene11213	NO	Phosphatidylinositol 3-kinase regulatory subunit alpha [Camponotus floridanus]
Unigene11447	NO	hypothetical protein SINV_14862 [Solenopsis invicta]
Unigene11731	NO	GE14316 [Drosophila yakuba]
Unigene11735	NO	PREDICTED: eukaryotic translation initiation factor 2-alpha kinase-like [Bombus terrestris]

Mitochondrial dysfunction	Unigene11808	NO	cytochrome c oxidase subunit II [Cotesia vestalis]
	Unigene11921	NO	PREDICTED: TATA-box-binding protein-like [Bombus terrestris]
	Unigene1213	NO	PREDICTED: calcineurin subunit B type 2-like isoform 1 [Nasonia vitripennis]
	Unigene12162	NO	PREDICTED: disintegrin and metalloproteinase domain-containing protein 10-like [Megachile rotundata]
	Unigene12247	NO	PREDICTED: hypothetical protein LOC100743521 [Bombus impatiens]
	Unigene12335	NO	PREDICTED: insulin-degrading enzyme-like isoform 2 [Nasonia vitripennis]
	Unigene1243	NO	GI19985 [Drosophila mojavensis]
	Unigene1263	NO	PREDICTED: microtubule-associated protein tau-like [Apis florea]
	Unigene12807	NO	PREDICTED: disintegrin and metalloproteinase domain-containing protein 10-like [Megachile rotundata]
	Unigene1286	NO	ADP/ATP translocase [Helicoverpa armigera]
	Unigene1290	NO	PREDICTED: succinate dehydrogenase [ubiquinone] flavoprotein subunit, mitochondrial-like [Megachile rotundata]
	Unigene13000	NO	PREDICTED: gamma-secretase subunit pen-2-like [Megachile rotundata]
	Unigene1310	NO	PREDICTED: troponin C, isoform 3 isoform 1 [Nasonia vitripennis]
	Unigene13136	NO	hypothetical protein SINV_10030 [Solenopsis invicta]
	Unigene1327	NO	hypothetical protein SINV_09347 [Solenopsis invicta]
	Unigene13515	NO	PREDICTED: TATA-box-binding protein-like [Megachile rotundata]
	Unigene13553	NO	PREDICTED: protein BCL9 homolog [Apis florea]
	Unigene13656	NO	cytochrome oxidase subunit II [Xyrosaris lichneuta]
	Unigene1371	NO	NADH dehydrogenase [ubiquinone] 1 beta subcomplex subunit 8, mitochondrial [Nasonia vitripennis]
	Unigene1404	NO	hypothetical protein AND_29000 [Anopheles darlingi]
	Unigene1440	NO	NADH dehydrogenase (ubiquinone) 1 beta subcomplex, 7 [Tribolium castaneum]
	Unigene1447	NO	NADH dehydrogenase [ubiquinone] iron-sulfur protein 6, mitochondrial [Nasonia vitripennis]
	Unigene1473	NO	hypothetical protein EAI_14262 [Harpegnathos saltator]
	Unigene1546	NO	PREDICTED: hypothetical protein LOC100119331 [Nasonia vitripennis]
	Unigene1574	NO	AGAP009602-PA [Anopheles gambiae str. PEST]
	Unigene1611	NO	PREDICTED: probable NADH dehydrogenase [ubiquinone] 1 alpha subcomplex subunit 12-like [Megachile rotundata]
	Unigene1633	NO	NADH dehydrogenase [ubiquinone] iron-sulfur protein 8, mitochondrial [Nasonia vitripennis]
	Unigene1646	NO	NADH dehydrogenase [Culex quinquefasciatus]
	Unigene1653	NO	AGAP012374-PA [Anopheles gambiae str. PEST]
	Unigene1688	NO	GD20916 [Drosophila simulans]
	Unigene1736	NO	AGAP009824-PA [Anopheles gambiae str. PEST]
	Unigene1738	NO	succinate dehydrogenase iron sulfur subunit B [Lysiphlebus testaceipes]
	Unigene1746	NO	TFIID [Acanthamoeba castellanii]
	Unigene1749	NO	NADH dehydrogenase [ubiquinone] 1 beta subcomplex subunit 2, mitochondrial [Camponotus floridanus]
	Unigene1756	NO	3-hydroxyacyl-CoA dehydrogenase type-2 [Camponotus floridanus]
	Unigene1800	NO	NADH dehydrogenase (ubiquinone) 1 alpha subcomplex, 9, 39kDa [Nasonia vitripennis]
	Unigene1833	NO	NADH dehydrogenase [ubiquinone] 1 alpha subcomplex subunit 2 [Camponotus floridanus]
	Unigene2076	NO	--
	Unigene2158	NO	PREDICTED: WD repeat-containing protein 55 homolog [Nasonia vitripennis]
	Unigene2224	NO	NADH dehydrogenase [Lysiphlebus testaceipes]
	Unigene2244	NO	AGAP006918-PA [Anopheles gambiae str. PEST]
	Unigene2346	NO	NADH dehydrogenase [ubiquinone] flavoprotein 2, mitochondrial [Nasonia vitripennis]
	Unigene2469	NO	Cell division protein kinase 5 [Harpegnathos saltator]
	Unigene2537	NO	ATP synthase subunit c [Manduca sexta]
	Unigene2610	NO	hypothetical protein SINV_04265 [Solenopsis invicta]
	Unigene2697	NO	cytochrome c oxidase polypeptide Vb [Bombyx mori]
	Unigene2856	NO	succinate dehydrogenase [ubiquinone] cytochrome b small subunit, mitochondrial [Nasonia vitripennis]
	Unigene2903	NO	hypothetical protein KGM_05439 [Danaus plexippus]
	Unigene2925	NO	PREDICTED: hypothetical protein LOC409983 [Apis mellifera]
	Unigene2926	NO	PREDICTED: calcium-transporting ATPase sarcoplasmic/endoplasmic reticulum type-like [Megachile rotundata]
	Unigene2956	NO	hypothetical protein SINV_02345 [Solenopsis invicta]
	Unigene3005	NO	PREDICTED: calumenin-like [Nasonia vitripennis]
	Unigene3257	NO	PREDICTED: hypothetical protein LOC100122162 [Nasonia vitripennis]
	Unigene3316	NO	PREDICTED: calcium-binding protein p22-like [Megachile rotundata]

Unigene3360	NO	Low-density lipoprotein receptor-related protein 1B [Camponotus floridanus]
Unigene3405	NO	ATP synthase [Bombyx mori]
Unigene346	NO	GJ13165 [Drosophila virilis]
Unigene368	NO	PREDICTED: ADP,ATP carrier protein 2-like [Bombus impatiens]
Unigene372	NO	PREDICTED: hypothetical protein LOC100115623 isoform 1 [Nasonia vitripennis]
Unigene3754	NO	PREDICTED: hypothetical protein LOC100122162 [Nasonia vitripennis]
Unigene383	NO	PREDICTED: cytochrome c-like isoform 1 [Bombus terrestris]
Unigene3882	NO	PREDICTED: NADH dehydrogenase [ubiquinone] iron-sulfur protein 4, mitochondrial-like [Megachile rotundata]
Unigene4061	NO	hypothetical protein AND_01489 [Anopheles darlingi]
Unigene4066	NO	PREDICTED: uncharacterized protein LOC100875487 [Megachile rotundata]
Unigene4072	NO	hypothetical protein KGM_03909 [Danaus plexippus]
Unigene4107	NO	hypothetical protein SINV_09114 [Solenopsis invicta]
Unigene411	NO	cytochrome c oxidase subunit VI [Microplitis mediator]
Unigene412	NO	hypothetical protein SINV_01823 [Solenopsis invicta]
Unigene4122	NO	mitochondrial cytochrome c oxidase subunit VIa [Bombyx mori]
Unigene426	NO	cytochrome c oxidase-like protein [Glyptapanteles flavicoxis]
Unigene4333	NO	H+ transporting ATP synthase gamma subunit [Danaus plexippus]
Unigene438	NO	cytochrome b-c1 complex subunit 9 [Nasonia vitripennis]
Unigene4442	NO	PREDICTED: insulin-degrading enzyme-like isoform 2 [Nasonia vitripennis]
Unigene4535	NO	hypothetical protein SINV_09114 [Solenopsis invicta]
Unigene4584	NO	PREDICTED: NADH-ubiquinone oxidoreductase 75 kDa subunit, mitochondrial-like [Bombus terrestris]
Unigene4589	NO	Low-density lipoprotein receptor-related protein 1 [Harpegnathos saltator]
Unigene467	NO	hypothetical protein SINV_11135 [Solenopsis invicta]
Unigene4742	NO	PREDICTED: calmodulin-like protein 4-like isoform 1 [Bombus terrestris]
Unigene4774	NO	mitochondrial cytochrome c [Bombyx mori]
Unigene485	NO	AT15141p [Drosophila melanogaster]
Unigene4852	NO	hypothetical protein KGM_08437 [Danaus plexippus]
Unigene4885	NO	ubiquinol-cytochrome C reductase complex protein [Danaus plexippus]
Unigene4903	NO	putative voltage-dependent anion-selective channel isoform 1 [Danaus plexippus]
Unigene4920	NO	PREDICTED: mitogen-activated protein kinase 1-like [Megachile rotundata]
Unigene4959	NO	ATP synthase [Danaus plexippus]
Unigene5043	NO	PREDICTED: hypothetical protein LOC100122162 [Nasonia vitripennis]
Unigene5072	NO	putative NADH:ubiquinone dehydrogenase [Danaus plexippus]
Unigene517	NO	GK10857 [Drosophila willistoni]
Unigene520	NO	cytochrome c oxidase subunit 4 isoform 1, mitochondrial [Nasonia vitripennis]
Unigene5280	NO	PREDICTED: LOW QUALITY PROTEIN: NEDD8-activating enzyme E1 regulatory subunit [Apis mellifera]
Unigene533	NO	ATP synthase subunit b, mitochondrial [Acromyrmex echinatior]
Unigene5492	NO	hypothetical protein KGM_00095 [Danaus plexippus]
Unigene553	NO	PREDICTED: cytochrome b-c1 complex subunit 6, mitochondrial-like [Megachile rotundata]
Unigene558	NO	AGAP008724-PA [Anopheles gambiae str. PEST]
Unigene564	NO	PREDICTED: similar to mitochondrial ATP synthase coupling factor 6 [Tribolium castaneum]
Unigene579	NO	cytochrome c oxidase subunit VIb isoform 1 [Rhipicephalus sanguineus]
Unigene6060	NO	lethal neo18 protein [Danaus plexippus]
Unigene608	NO	hypothetical protein EAG_05916 [Camponotus floridanus]
Unigene614	NO	Cytochrome b-c1 complex subunit Rieske, mitochondrial [Camponotus floridanus]
Unigene624	NO	ATP synthase subunit gamma, mitochondrial [Nasonia vitripennis]
Unigene626	NO	Voltage-dependent anion-selective channel [Acromyrmex echinatior]
Unigene6363	NO	Mitogen-activated protein kinase 1 [Harpegnathos saltator]
Unigene647	NO	PREDICTED: cytochrome b-c1 complex subunit Rieske, mitochondrial-like [Cavia porcellus]
Unigene6600	NO	PREDICTED: presenilin homolog [Bombus impatiens]
Unigene663	NO	Cytochrome b-c1 complex subunit 7 [Camponotus floridanus]
Unigene6630	NO	cytochrome c oxidase [Danaus plexippus]
Unigene6640	NO	Nicastrin [Camponotus floridanus]
Unigene6642	NO	NADH dehydrogenase [Danaus plexippus]
Unigene6695	NO	unnamed protein product [Heliconius melpomene]
Unigene6842	NO	guanine nucleotide binding protein alpha q polypeptide [Glossina morsitans morsitans]
Unigene6884	NO	H+ transporting ATP synthase O subunit isoform 1 [Bombyx mori]
Unigene694	NO	ATP synthase subunit O, mitochondrial [Camponotus floridanus]
Unigene698	NO	ADP/ATP translocase [Manduca sexta]
Unigene71	NO	hypothetical protein EAI_14262 [Harpegnathos saltator]

	Unigene7100	NO	cytochrome c oxidase polypeptide IV [Bombyx mori]
	Unigene7180	NO	39S ribosomal protein L46, mitochondrial [Acromyrmex echinatior]
	Unigene7225	NO	PREDICTED: hypothetical protein LOC408577 [Apis mellifera]
	Unigene731	NO	hypothetical protein SINV_08923 [Solenopsis invicta]
	Unigene743	NO	GF11012 [Drosophila ananassae]
	Unigene7464	NO	Eukaryotic translation initiation factor 2-alpha kinase [Acromyrmex echinatior]
	Unigene7469	NO	NADH-ubiquinone oxidoreductase 75 kDa subunit, mitochondrial precursor [Nasonia vitripennis]
	Unigene7619	NO	hypothetical protein SINV_04265 [Solenopsis invicta]
	Unigene763	NO	hypothetical protein SINV_15147 [Solenopsis invicta]
	Unigene7739	NO	control protein HCTL024 [Heliconius hortense]
	Unigene776	NO	AT15141p [Drosophila melanogaster]
	Unigene787	NO	NADH dehydrogenase [ubiquinone] 1 alpha subcomplex subunit 6 [Nasonia vitripennis]
	Unigene7938	NO	hypothetical protein KGM_04538 [Danaus plexippus]
	Unigene799	NO	hypothetical protein SINV_06969 [Solenopsis invicta]
	Unigene7994	NO	PREDICTED: hypothetical protein LOC100122162 [Nasonia vitripennis]
	Unigene8104	NO	NADH dehydrogenase [Danaus plexippus]
	Unigene8113	NO	Gamma-secretase subunit Aph-1 [Camponotus floridanus]
	Unigene8196	NO	cytochrome c oxidase subunit III [Cotesia vestalis]
	Unigene8222	NO	ubiquinol-cytochrome C reductase complex 14kD subunit [Danaus plexippus]
	Unigene8267	NO	Insulin-degrading enzyme [Camponotus floridanus]
	Unigene829	NO	ATP synthase subunit d, mitochondrial [Camponotus floridanus]
	Unigene8339	NO	PREDICTED: hypothetical protein LOC100743562 isoform 2 [Bombus impatiens]
	Unigene8344	NO	PREDICTED: phosphatidylinositol 3-kinase regulatory subunit alpha-like, partial [Apis florea]
	Unigene837	NO	hypothetical protein AND_29231 [Anopheles darlingi]
	Unigene8416	NO	H+ transporting ATP synthase O subunit isoform 1 [Bombyx mori]
	Unigene8887	NO	PREDICTED: gamma-secretase subunit Aph-1-like [Megachile rotundata]
	Unigene8922	NO	mitochondrial ATP synthase coupling factor [Antheraea yamamai]
	Unigene9008	NO	PREDICTED: guanine nucleotide-binding protein G(q) subunit alpha-like isoform 5 [Megachile rotundata]
	Unigene912	NO	cytochrome c1, heme protein, mitochondrial [Nasonia vitripennis]
	Unigene9141	NO	PREDICTED: hypothetical protein LOC100743562 isoform 1 [Bombus impatiens]
	Unigene9217	NO	PREDICTED: stress-activated protein kinase JNK-like [Megachile rotundata]
	Unigene9356	NO	pancreatic lipase 3 [Mamestra configurata]
	Unigene9359	NO	PREDICTED: hypothetical protein LOC100743083 [Bombus impatiens]
	Unigene9462	NO	NADH dehydrogenase (ubiquinone) Fe-S protein 8 [Bombyx mori]
	Unigene9485	NO	H+ transporting ATP synthase O subunit isoform 1 [Danaus plexippus]
	Unigene9517	NO	PREDICTED: insulin-degrading enzyme-like isoform 2 [Nasonia vitripennis]
	Unigene952	NO	PREDICTED: acyl carrier protein, mitochondrial-like [Megachile rotundata]
	Unigene959	NO	NADH dehydrogenase [ubiquinone] 1 beta subcomplex subunit 5, mitochondrial [Nasonia vitripennis]
	Unigene9659	NO	sarco/endoplasmic reticulum calcium ATPase [Bombyx mori]
	Unigene991	NO	PREDICTED: NADH dehydrogenase [ubiquinone] 1 alpha subcomplex subunit 7-like [Megachile rotundata]
	Unigene9927	NO	Subunit VIb of cytochrome c oxidase [Danaus plexippus]
	Unigene9971	NO	hypothetical protein KGM_15724 [Danaus plexippus]
	Unigene9998	NO	PREDICTED: stress-activated protein kinase JNK-like [Megachile rotundata]
	CL182.Contig1	NO	pyruvate kinase [Trypanosoma brucei]
	CL334.Contig1	SP	Calcium-transporting ATPase sarcoplasmic/endoplasmic reticulum type [Acromyrmex echinatior]
	CL430.Contig2	SP	PREDICTED: tenascin-X-like [Nasonia vitripennis]
	CL590.Contig1	SP	PREDICTED: serine/threonine-protein kinase/endoribonuclease IRE1-like [Nasonia vitripennis]
	Unigene1011	SP	PREDICTED: membrane metallo-endopeptidase-like 1-like [Bombus impatiens]
	Unigene10722	SP	Protein VAC14-like protein [Camponotus floridanus]
	Unigene12612	SP	Armadillo segment polarity protein [Harpegnathos saltator]
	Unigene1289	SP	cytochrome oxidase subunit I [Cotesia chilonis]
	Unigene2376	SP	hypothetical protein TcasGA2_TC010888 [Tribolium castaneum]
	Unigene244	SP	calreticulin [Cotesia rubecula]
	Unigene2802	SP	PREDICTED: serine/threonine-protein kinase/endoribonuclease IRE1-like [Megachile rotundata]
	Unigene3097	SP	PREDICTED: lipase member H-A-like [Apis mellifera]
	Unigene3386	SP	Calnexin [Camponotus floridanus]
	Unigene3500	SP	PREDICTED: serine/threonine-protein kinase/endoribonuclease IRE1-like [Nasonia vitripennis]

Unigene4476	SP	hypothetical protein SINV_04265 [Solenopsis invicta]
Unigene4608	SP	Serine/threonine-protein kinase/endoribonuclease ire-1 [Camponotus floridanus]
Unigene6037	SP	PREDICTED: serine/threonine-protein kinase/endoribonuclease IRE1-like [Nasonia vitripennis]
Unigene6049	SP	hypothetical protein SINV_07475 [Solenopsis invicta]
Unigene6782	SP	PREDICTED: calnexin-like [Megachile rotundata]
Unigene96	SP	Vascular endothelial growth factor receptor 2 [Harpegnathos saltator]
CL205.Contig1	NO	PREDICTED: NADH dehydrogenase [ubiquinone] flavoprotein 1, mitochondrial-like [Apis florea]
CL205.Contig2	NO	PREDICTED: NADH dehydrogenase [ubiquinone] flavoprotein 1, mitochondrial [Apis mellifera]
CL231.Contig1	NO	ATP synthase subunit alpha, mitochondrial [Harpegnathos saltator]
CL238.Contig1	NO	PREDICTED: protein kinase shaggy-like isoform 2 [Nasonia vitripennis]
CL238.Contig2	NO	PREDICTED: protein kinase shaggy-like isoform 2 [Nasonia vitripennis]
CL238.Contig4	NO	Protein kinase shaggy [Acromyrmex echinatior]
CL239.Contig1	NO	PREDICTED: uncharacterized protein LOC100878829 [Megachile rotundata]
CL352.Contig1	NO	cytochrome c oxidase subunit I [Cotesia vestalis]
CL352.Contig2	NO	cytochrome oxidase subunit I [Bucculatrix artemisiella]
CL352.Contig3	NO	cytochrome c oxidase subunit I [Cotesia vestalis]
CL363.Contig1	NO	PREDICTED: serine/threonine-protein phosphatase 2B catalytic subunit 2-like [Apis florea]
CL363.Contig2	NO	PREDICTED: serine/threonine-protein phosphatase 2B catalytic subunit 2-like [Apis florea]
CL374.Contig1	NO	ATP synthase subunit beta, mitochondrial [Harpegnathos saltator]
CL374.Contig2	NO	ATP-synthase subunit beta [Schistocerca gregaria]
CL374.Contig3	NO	ATP synthase subunit beta, putative [Pediculus humanus corporis]
CL401.Contig2	NO	PREDICTED: ATP synthase subunit epsilon, mitochondrial-like isoform 1 [Apis mellifera]
CL412.Contig1	NO	hypothetical protein SINV_01823 [Solenopsis invicta]
CL421.Contig2	NO	PREDICTED: glyceraldehyde-3-phosphate dehydrogenase 2 [Nasonia vitripennis]
CL438.Contig1	NO	PREDICTED: GTP-binding nuclear protein Ran [Apis mellifera]
CL438.Contig2	NO	GTP-binding nuclear protein ran [Danaus plexippus]
CL438.Contig3	NO	GTP-binding nuclear protein ran [Danaus plexippus]
CL466.Contig1	NO	sugar transporter, putative [Ixodes scapularis]
CL483.Contig1	NO	PREDICTED: hypothetical protein LOC100743933 [Bombus impatiens]
CL483.Contig2	NO	Microtubule-associated protein 2 [Harpegnathos saltator]
CL51.Contig1	NO	hypothetical protein SINV_04265 [Solenopsis invicta]
CL560.Contig1	NO	cytochrome b [Cotesia vestalis]
CL591.Contig1	NO	PREDICTED: E3 ubiquitin-protein ligase IAP-3-like [Bombus impatiens]
CL591.Contig2	NO	PREDICTED: E3 ubiquitin-protein ligase IAP-3-like [Megachile rotundata]
CL597.Contig2	NO	cytochrome b [Cotesia vestalis]
CL676.Contig1	NO	PREDICTED: g1/S-specific cyclin-D2-like [Bombus terrestris]
CL740.Contig1	NO	PREDICTED: NADH dehydrogenase [ubiquinone] flavoprotein 1, mitochondrial-like [Megachile rotundata]
CL750.Contig1	NO	PREDICTED: ATP synthase lipid-binding protein, mitochondrial-like [Megachile rotundata]
CL757.Contig2	NO	PREDICTED: uncharacterized protein LOC100876927 [Megachile rotundata]
CL792.Contig1	NO	hypothetical protein SINV_06702 [Solenopsis invicta]
Unigene10083	NO	putative armadillo protein [Danaus plexippus]
Unigene10090	NO	PREDICTED: LOW QUALITY PROTEIN: g2/mitotic-specific cyclin-B3-like [Bombus terrestris]
Unigene1012	NO	Cytochrome b-c1 complex subunit 2, mitochondrial [Camponotus floridanus]
Unigene10125	NO	hypothetical protein SINV_80464 [Solenopsis invicta]
Unigene10138	NO	PREDICTED: uncharacterized protein LOC100881293 [Megachile rotundata]
Unigene1014	NO	PREDICTED: NADH dehydrogenase [ubiquinone] iron-sulfur protein 7, mitochondrial-like [Apis florea]
Unigene10183	NO	Talin-1 [Acromyrmex echinatior]
Unigene10285	NO	PREDICTED: exportin-1-like [Megachile rotundata]
Unigene10363	NO	GTP-binding protein alpha subunit, gna [Culex quinquefasciatus]
Unigene10410	NO	Mothers against decapentaplegic-like protein 3 [Acromyrmex echinatior]
Unigene1043	NO	PREDICTED: cytochrome c oxidase subunit 6C-like [Acyrthosiphon pisum]
Unigene10437	NO	PREDICTED: exportin-1-like [Megachile rotundata]
Unigene1044	NO	GF11341 [Drosophila ananassae]
Unigene10512	NO	Mitotic checkpoint protein BUB3 [Acromyrmex echinatior]
Unigene10590	NO	hypothetical protein KGM_04009 [Danaus plexippus]
Unigene10610	NO	PREDICTED: hypothetical protein LOC100114432 [Nasonia vitripennis]

Unigene1074	NO	NADH dehydrogenase [ubiquinone] 1 alpha subcomplex subunit 13 [Camponotus floridanus]
Unigene10847	NO	PREDICTED: nicastrin-like [Megachile rotundata]
Unigene1090	NO	PREDICTED: similar to NADH:ubiquinone dehydrogenase, putative [Tribolium castaneum]
Unigene10956	NO	PREDICTED: LOW QUALITY PROTEIN: RING finger and SPRY domain-containing protein 1-like [Bombus terrestris]
Unigene11062	NO	Stress-activated protein kinase JNK [Acromyrmex echinatior]
Unigene11124	NO	Presenilin-like protein [Harpegnathos saltator]
Unigene11156	NO	PREDICTED: nicastrin [Apis mellifera]
Unigene116	NO	Ras-related nuclear protein [Mizuhopecten yessoensis]
Unigene1120	NO	PREDICTED: endothelin-converting enzyme 1-like [Nasonia vitripennis]
Unigene11213	NO	Phosphatidylinositol 3-kinase regulatory subunit alpha [Camponotus floridanus]
Unigene11321	NO	hypothetical protein SINV_80464 [Solenopsis invicta]
Unigene11447	NO	hypothetical protein SINV_14862 [Solenopsis invicta]
Unigene1149	NO	hypothetical protein DAPPUDRAFT_51702 [Daphnia pulex]
Unigene11645	NO	putative ETS-like protein pointed, isoform P1 [Danaus plexippus]
Unigene11731	NO	GE14316 [Drosophila yakuba]
Unigene11735	NO	PREDICTED: eukaryotic translation initiation factor 2-alpha kinase-like [Bombus terrestris]
Unigene11808	NO	cytochrome c oxidase subunit II [Cotesia vestalis]
Unigene11921	NO	PREDICTED: TATA-box-binding protein-like [Bombus terrestris]
Unigene11930	NO	PREDICTED: cell division cycle protein 16 homolog [Megachile rotundata]
Unigene11985	NO	PREDICTED: hypothetical protein LOC408354 [Apis mellifera]
Unigene12096	NO	PREDICTED: protein VAC14 homolog [Megachile rotundata]
Unigene1213	NO	PREDICTED: calcineurin subunit B type 2-like isoform 1 [Nasonia vitripennis]
Unigene12162	NO	PREDICTED: disintegrin and metalloproteinase domain-containing protein 10-like [Megachile rotundata]
Unigene12247	NO	PREDICTED: hypothetical protein LOC100743521 [Bombus impatiens]
Unigene12335	NO	PREDICTED: insulin-degrading enzyme-like isoform 2 [Nasonia vitripennis]
Unigene1243	NO	GI19985 [Drosophila mojavensis]
Unigene1263	NO	PREDICTED: microtubule-associated protein tau-like [Apis florea]
Unigene12735	NO	PREDICTED: armadillo segment polarity protein isoform 2 [Nasonia vitripennis]
Unigene12807	NO	PREDICTED: disintegrin and metalloproteinase domain-containing protein 10-like [Megachile rotundata]
Unigene1286	NO	ADP/ATP translocase [Helicoverpa armigera]
Unigene1290	NO	PREDICTED: succinate dehydrogenase [ubiquinone] flavoprotein subunit, mitochondrial-like [Megachile rotundata]
Unigene13000	NO	PREDICTED: gamma-secretase subunit pen-2-like [Megachile rotundata]
Unigene1310	NO	PREDICTED: troponin C, isoform 3 isoform 1 [Nasonia vitripennis]
Unigene13136	NO	hypothetical protein SINV_10030 [Solenopsis invicta]
Unigene13138	NO	PREDICTED: segment polarity protein dishevelled homolog DVL-3-like [Megachile rotundata]
Unigene13191	NO	PREDICTED: cyclic AMP-dependent transcription factor ATF-2-like [Apis mellifera]
Unigene1327	NO	hypothetical protein SINV_09347 [Solenopsis invicta]
Unigene13515	NO	PREDICTED: TATA-box-binding protein-like [Megachile rotundata]
Unigene13553	NO	PREDICTED: protein BCL9 homolog [Apis florea]
Unigene13656	NO	cytochrome oxidase subunit II [Xyrosaris lichneuta]
Unigene1371	NO	NADH dehydrogenase [ubiquinone] 1 beta subcomplex subunit 8, mitochondrial [Nasonia vitripennis]
Unigene1404	NO	hypothetical protein AND_29000 [Anopheles darlingi]
Unigene1437	NO	PREDICTED: translocator protein-like [Bombus impatiens]
Unigene1440	NO	NADH dehydrogenase (ubiquinone) 1 beta subcomplex, 7 [Tribolium castaneum]
Unigene1446	NO	PREDICTED: proto-oncogene c-Fos-like [Bombus terrestris]
Unigene1447	NO	NADH dehydrogenase [ubiquinone] iron-sulfur protein 6, mitochondrial [Nasonia vitripennis]
Unigene1473	NO	hypothetical protein EAI_14262 [Harpegnathos saltator]
Unigene1546	NO	PREDICTED: hypothetical protein LOC100119331 [Nasonia vitripennis]
Unigene1574	NO	AGAP009602-PA [Anopheles gambiae str. PEST]
Unigene1611	NO	PREDICTED: probable NADH dehydrogenase [ubiquinone] 1 alpha subcomplex subunit 12-like [Megachile rotundata]
Unigene1633	NO	NADH dehydrogenase [ubiquinone] iron-sulfur protein 8, mitochondrial [Nasonia vitripennis]
Unigene1646	NO	NADH dehydrogenase [Culex quinquefasciatus]
Unigene1653	NO	AGAP012374-PA [Anopheles gambiae str. PEST]
Unigene1736	NO	AGAP009824-PA [Anopheles gambiae str. PEST]

DNA damage and repair	Unigene1738	NO	succinate dehydrogenase iron sulfur subunit B [Lysiphlebus testaceipes]
	Unigene1749	NO	NADH dehydrogenase [ubiquinone] 1 beta subcomplex subunit 2, mitochondrial [Camponotus floridanus]
	Unigene1756	NO	3-hydroxyacyl-CoA dehydrogenase type-2 [Camponotus floridanus]
	Unigene1800	NO	NADH dehydrogenase (ubiquinone) 1 alpha subcomplex, 9, 39kDa [Nasonia vitripennis]
	Unigene1833	NO	NADH dehydrogenase [ubiquinone] 1 alpha subcomplex subunit 2 [Camponotus floridanus]
	Unigene2059	NO	budding uninhibited by benzimidazoles 3 [Tribolium castaneum]
	Unigene2069	NO	PREDICTED: ras-like protein 1-like [Apis florea]
	Unigene2076	NO	--
	Unigene2158	NO	PREDICTED: WD repeat-containing protein 55 homolog [Nasonia vitripennis]
	Unigene2196	NO	PREDICTED: serine/threonine-protein kinase grp isoform 2 [Apis mellifera]
	Unigene2210	NO	PREDICTED: signal transducer and activator of transcription 5B-like [Megachile rotundata]
	Unigene2224	NO	NADH dehydrogenase [Lysiphlebus testaceipes]
	Unigene2244	NO	AGAP006918-PA [Anopheles gambiae str. PEST]
	Unigene2346	NO	NADH dehydrogenase [ubiquinone] flavoprotein 2, mitochondrial [Nasonia vitripennis]
	Unigene2365	NO	PREDICTED: ran-specific GTPase-activating protein-like [Bombus impatiens]
	Unigene2418	NO	PREDICTED: RAC serine/threonine-protein kinase-like [Bombus impatiens]
	Unigene2469	NO	Cell division protein kinase 5 [Harpegnathos saltator]
	Unigene25	NO	PREDICTED: ras-like protein 2-like [Nasonia vitripennis]
	Unigene2537	NO	ATP synthase subunit c [Manduca sexta]
	Unigene2544	NO	PREDICTED: uncharacterized protein LOC100879259 [Megachile rotundata]
	Unigene2610	NO	hypothetical protein SINV_04265 [Solenopsis invicta]
	Unigene2697	NO	cytochrome c oxidase polypeptide Vb [Bombyx mori]
	Unigene2856	NO	succinate dehydrogenase [ubiquinone] cytochrome b small subunit, mitochondrial [Nasonia vitripennis]
	Unigene2903	NO	hypothetical protein KGM_05439 [Danaus plexippus]
	Unigene2925	NO	PREDICTED: hypothetical protein LOC409983 [Apis mellifera]
	Unigene2926	NO	PREDICTED: calcium-transporting ATPase sarcoplasmic/endoplasmic reticulum type-like [Megachile rotundata]
	Unigene2956	NO	hypothetical protein SINV_02345 [Solenopsis invicta]
	Unigene2992	NO	Exportin-1 [Harpegnathos saltator]
	Unigene3005	NO	PREDICTED: calumenin-like [Nasonia vitripennis]
	Unigene3137	NO	PREDICTED: ran-specific GTPase-activating protein-like [Apis mellifera]
	Unigene3146	NO	Ran-binding protein 9 [Camponotus floridanus]
	Unigene3257	NO	PREDICTED: hypothetical protein LOC100122162 [Nasonia vitripennis]
	Unigene3316	NO	PREDICTED: calcium-binding protein p22-like [Megachile rotundata]
	Unigene3344	NO	PREDICTED: solute carrier family 2, facilitated glucose transporter member 1-like [Nasonia vitripennis]
	Unigene3360	NO	Low-density lipoprotein receptor-related protein 1B [Camponotus floridanus]
	Unigene3376	NO	PREDICTED: hypothetical protein LOC100745000 [Bombus impatiens]
	Unigene3405	NO	ATP synthase [Bombyx mori]
	Unigene3407	NO	PREDICTED: sideroflexin-1-like isoform 2 [Nasonia vitripennis]
	Unigene346	NO	GJ13165 [Drosophila virilis]
	Unigene3519	NO	PREDICTED: uncharacterized protein LOC100867253 [Apis florea]
	Unigene368	NO	PREDICTED: ADP,ATP carrier protein 2-like [Bombus impatiens]
	Unigene372	NO	PREDICTED: hypothetical protein LOC100115623 isoform 1 [Nasonia vitripennis]
	Unigene3745	NO	PREDICTED: protein Dr1-like [Megachile rotundata]
	Unigene3754	NO	PREDICTED: hypothetical protein LOC100122162 [Nasonia vitripennis]
	Unigene3784	NO	Ran-binding protein 3 [Camponotus floridanus]
	Unigene383	NO	PREDICTED: cytochrome c-like isoform 1 [Bombus terrestris]
	Unigene3866	NO	hypothetical protein EAI_17259 [Harpegnathos saltator]
	Unigene3882	NO	PREDICTED: NADH dehydrogenase [ubiquinone] iron-sulfur protein 4, mitochondrial-like [Megachile rotundata]
	Unigene3960	NO	PREDICTED: myb-related protein A-like [Nasonia vitripennis]
	Unigene4045	NO	Dual specificity mitogen-activated protein kinase kinase 4 [Acromyrmex echinatior]
	Unigene4061	NO	hypothetical protein AND_01489 [Anopheles darlingi]
	Unigene4066	NO	PREDICTED: uncharacterized protein LOC100875487 [Megachile rotundata]
	Unigene4072	NO	hypothetical protein KGM_03909 [Danaus plexippus]
	Unigene4107	NO	hypothetical protein SINV_09114 [Solenopsis invicta]
	Unigene411	NO	cytochrome c oxidase subunit VI [Microplitis mediator]
	Unigene412	NO	hypothetical protein SINV_01823 [Solenopsis invicta]
	Unigene4122	NO	mitochondrial cytochrome c oxidase subunit VIa [Bombyx mori]
	Unigene4136	NO	Myb protein [Camponotus floridanus]

Unigene4193	NO	hypothetical protein SINV_10623 [Solenopsis invicta]
Unigene426	NO	cytochrome c oxidase-like protein [Glyptapanteles flavicoxis]
Unigene4333	NO	H+ transporting ATP synthase gamma subunit [Danaus plexippus]
Unigene4353	NO	RING finger protein unkempt-like protein [Harpegnathos saltator]
Unigene438	NO	cytochrome b-c1 complex subunit 9 [Nasonia vitripennis]
Unigene4442	NO	PREDICTED: insulin-degrading enzyme-like isoform 2 [Nasonia vitripennis]
Unigene4485	NO	PREDICTED: LOW QUALITY PROTEIN: talin-1-like [Megachile rotundata]
Unigene452	NO	PREDICTED: activating transcription factor of chaperone-like [Megachile rotundata]
Unigene4535	NO	hypothetical protein SINV_09114 [Solenopsis invicta]
Unigene4584	NO	PREDICTED: NADH-ubiquinone oxidoreductase 75 kDa subunit, mitochondrial-like [Bombus terrestris]
Unigene4589	NO	Low-density lipoprotein receptor-related protein 1 [Harpegnathos saltator]
Unigene4655	NO	PREDICTED: transcription factor E2F3-like isoform 1 [Bombus terrestris]
Unigene466	NO	PREDICTED: hypothetical protein LOC100747421 [Bombus impatiens]
Unigene467	NO	hypothetical protein SINV_11135 [Solenopsis invicta]
Unigene4710	NO	Dual specificity mitogen-activated protein kinase kinase 4 [Acromyrmex echinatior]
Unigene4742	NO	PREDICTED: calmodulin-like protein 4-like isoform 1 [Bombus terrestris]
Unigene4774	NO	mitochondrial cytochrome c [Bombyx mori]
Unigene485	NO	AT15141p [Drosophila melanogaster]
Unigene4852	NO	hypothetical protein KGM_08437 [Danaus plexippus]
Unigene4885	NO	ubiquinol-cytochrome C reductase complex protein [Danaus plexippus]
Unigene4903	NO	putative voltage-dependent anion-selective channel isoform 1 [Danaus plexippus]
Unigene4920	NO	PREDICTED: mitogen-activated protein kinase 1-like [Megachile rotundata]
Unigene4959	NO	ATP synthase [Danaus plexippus]
Unigene4973	NO	PREDICTED: serine/threonine-protein kinase grp isoform 2 [Apis mellifera]
Unigene5020	NO	hypothetical protein AND_02161 [Anopheles darlingi]
Unigene5043	NO	PREDICTED: hypothetical protein LOC100122162 [Nasonia vitripennis]
Unigene5072	NO	putative NADH:ubiquinone dehydrogenase [Danaus plexippus]
Unigene514	NO	PREDICTED: serine/threonine-protein kinase KDX1-like [Bombus impatiens]
Unigene517	NO	GK10857 [Drosophila willistoni]
Unigene5171	NO	PREDICTED: talin-2-like [Bombus terrestris]
Unigene520	NO	cytochrome c oxidase subunit 4 isoform 1, mitochondrial [Nasonia vitripennis]
Unigene5212	NO	Proliferating cell nuclear antigen [Camponotus floridanus]
Unigene5280	NO	PREDICTED: LOW QUALITY PROTEIN: NEDD8-activating enzyme E1 regulatory subunit [Apis mellifera]
Unigene533	NO	ATP synthase subunit b, mitochondrial [Acromyrmex echinatior]
Unigene5357	NO	PREDICTED: exportin-1-like [Nasonia vitripennis]
Unigene5446	NO	PREDICTED: G1/S-specific cyclin-D2-like [Megachile rotundata]
Unigene5492	NO	hypothetical protein KGM_00095 [Danaus plexippus]
Unigene5515	NO	PREDICTED: armadillo segment polarity protein-like [Megachile rotundata]
Unigene553	NO	PREDICTED: cytochrome b-c1 complex subunit 6, mitochondrial-like [Megachile rotundata]
Unigene558	NO	AGAP008724-PA [Anopheles gambiae str. PEST]
Unigene5593	NO	PREDICTED: hypothetical protein LOC100745810 isoform 2 [Bombus impatiens]
Unigene564	NO	PREDICTED: similar to mitochondrial ATP synthase coupling factor 6 [Tribolium castaneum]
Unigene5691	NO	PREDICTED: hypothetical protein LOC412916 [Apis mellifera]
Unigene579	NO	cytochrome c oxidase subunit VIb isoform 1 [Rhipicephalus sanguineus]
Unigene5806	NO	PREDICTED: uncharacterized protein LOC100878829 [Megachile rotundata]
Unigene6060	NO	lethal neo18 protein [Danaus plexippus]
Unigene608	NO	hypothetical protein EAG_05916 [Camponotus floridanus]
Unigene614	NO	Cytochrome b-c1 complex subunit Rieske, mitochondrial [Camponotus floridanus]
Unigene624	NO	ATP synthase subunit gamma, mitochondrial [Nasonia vitripennis]
Unigene626	NO	Voltage-dependent anion-selective channel [Acromyrmex echinatior]
Unigene6363	NO	Mitogen-activated protein kinase 1 [Harpegnathos saltator]
Unigene6394	NO	Talin-1 [Camponotus floridanus]
Unigene647	NO	PREDICTED: cytochrome b-c1 complex subunit Rieske, mitochondrial-like [Cavia porcellus]
Unigene6600	NO	PREDICTED: presenilin homolog [Bombus impatiens]
Unigene663	NO	Cytochrome b-c1 complex subunit 7 [Camponotus floridanus]
Unigene6630	NO	cytochrome c oxidase [Danaus plexippus]
Unigene6640	NO	Nicastrin [Camponotus floridanus]
Unigene6642	NO	NADH dehydrogenase [Danaus plexippus]
Unigene6695	NO	unnamed protein product [Heliconius melpomene]
Unigene6700	NO	Talin-1 [Camponotus floridanus]
Unigene6842	NO	guanine nucleotide binding protein alpha q polypeptide [Glossina morsitans morsitans]

Unigene6884	NO	H+ transporting ATP synthase O subunit isoform 1 [Bombyx mori]
Unigene6899	NO	PREDICTED: proliferating cell nuclear antigen-like [Nasonia vitripennis]
Unigene694	NO	ATP synthase subunit O, mitochondrial [Camponotus floridanus]
Unigene6948	NO	PREDICTED: anaphase-promoting complex subunit CDC26-like isoform 1 [Apis florea]
Unigene698	NO	ADP/ATP translocase [Manduca sexta]
Unigene6991	NO	PREDICTED: anaphase-promoting complex subunit 10-like [Megachile rotundata]
Unigene7044	NO	PREDICTED: cell division cycle protein 20 homolog [Canis lupus familiaris]
Unigene7068	NO	PREDICTED: nuclear transcription factor Y subunit beta-like [Bombus impatiens]
Unigene71	NO	hypothetical protein EAI_14262 [Harpegnathos saltator]
Unigene7100	NO	cytochrome c oxidase polypeptide IV [Bombyx mori]
Unigene7161	NO	PREDICTED: sideroflexin-1-like isoform 2 [Nasonia vitripennis]
Unigene7180	NO	39S ribosomal protein L46, mitochondrial [Acromyrmex echinatior]
Unigene7225	NO	PREDICTED: hypothetical protein LOC408577 [Apis mellifera]
Unigene7237	NO	PREDICTED: hypothetical protein LOC100747412 isoform 2 [Bombus impatiens]
Unigene731	NO	hypothetical protein SINV_08923 [Solenopsis invicta]
Unigene7340	NO	hypothetical protein SINV_10840 [Solenopsis invicta]
Unigene743	NO	GF11012 [Drosophila ananassae]
Unigene7464	NO	Eukaryotic translation initiation factor 2-alpha kinase [Acromyrmex echinatior]
Unigene7469	NO	NADH-ubiquinone oxidoreductase 75 kDa subunit, mitochondrial precursor [Nasonia vitripennis]
Unigene7498	NO	hypothetical protein SINV_12469 [Solenopsis invicta]
Unigene7595	NO	PREDICTED: anaphase-promoting complex subunit 11-like [Apis mellifera]
Unigene7619	NO	hypothetical protein SINV_04265 [Solenopsis invicta]
Unigene763	NO	hypothetical protein SINV_15147 [Solenopsis invicta]
Unigene7655	NO	PREDICTED: cell division cycle protein 27 homolog [Megachile rotundata]
Unigene7739	NO	control protein HCTL024 [Heliconius hortense]
Unigene7754	NO	PREDICTED: cyclin-dependent kinase 6-like [Apis florea]
Unigene776	NO	AT15141p [Drosophila melanogaster]
Unigene7857	NO	hypothetical protein SINV_10861 [Solenopsis invicta]
Unigene787	NO	NADH dehydrogenase [ubiquinone] 1 alpha subcomplex subunit 6 [Nasonia vitripennis]
Unigene7938	NO	hypothetical protein KGM_04538 [Danaus plexippus]
Unigene799	NO	hypothetical protein SINV_06969 [Solenopsis invicta]
Unigene7994	NO	PREDICTED: hypothetical protein LOC100122162 [Nasonia vitripennis]
Unigene8049	NO	Talin-1 [Camponotus floridanus]
Unigene8102	NO	hypothetical protein SINV_10861 [Solenopsis invicta]
Unigene8104	NO	NADH dehydrogenase [Danaus plexippus]
Unigene8113	NO	Gamma-secretase subunit Aph-1 [Camponotus floridanus]
Unigene8196	NO	cytochrome c oxidase subunit III [Cotesia vestalis]
Unigene8222	NO	ubiquinol-cytochrome C reductase complex 14kD subunit [Danaus plexippus]
Unigene8266	NO	PREDICTED: mothers against decapentaplegic homolog 3 [Nasonia vitripennis]
Unigene8267	NO	Insulin-degrading enzyme [Camponotus floridanus]
Unigene829	NO	ATP synthase subunit d, mitochondrial [Camponotus floridanus]
Unigene8339	NO	PREDICTED: hypothetical protein LOC100743562 isoform 2 [Bombus impatiens]
Unigene8344	NO	PREDICTED: phosphatidylinositol 3-kinase regulatory subunit alpha-like, partial [Apis florea]
Unigene837	NO	hypothetical protein AND_29231 [Anopheles darlingi]
Unigene8372	NO	PREDICTED: baculoviral IAP repeat-containing protein 3-like [Nasonia vitripennis]
Unigene8416	NO	H+ transporting ATP synthase O subunit isoform 1 [Bombyx mori]
Unigene8488	NO	PREDICTED: uncharacterized protein LOC100877711 [Megachile rotundata]
Unigene8610	NO	PREDICTED: talin-1-like [Apis mellifera]
Unigene8887	NO	PREDICTED: gamma-secretase subunit Aph-1-like [Megachile rotundata]
Unigene8922	NO	mitochondrial ATP synthase coupling factor [Antheraea yamamai]
Unigene9008	NO	PREDICTED: guanine nucleotide-binding protein G(q) subunit alpha-like isoform 5 [Megachile rotundata]
Unigene905	NO	PREDICTED: transcription factor AP-1-like [Megachile rotundata]
Unigene912	NO	cytochrome c1, heme protein, mitochondrial [Nasonia vitripennis]
Unigene9141	NO	PREDICTED: hypothetical protein LOC100743562 isoform 1 [Bombus impatiens]
Unigene9217	NO	PREDICTED: stress-activated protein kinase JNK-like [Megachile rotundata]
Unigene9292	NO	PREDICTED: LOW QUALITY PROTEIN: talin-2-like [Apis florea]
Unigene9328	NO	PREDICTED: LOW QUALITY PROTEIN: talin-1-like [Megachile rotundata]
Unigene934	NO	PREDICTED: farnesyl pyrophosphate synthase-like [Nasonia vitripennis]
Unigene9356	NO	pancreatic lipase 3 [Mamestra configurata]
Unigene9359	NO	PREDICTED: hypothetical protein LOC100743083 [Bombus impatiens]
Unigene9378	NO	PREDICTED: armadillo segment polarity protein-like [Megachile rotundata]
Unigene9462	NO	NADH dehydrogenase ubiquinone Fe-S 8 [Bombyx mori]

	Unigene9485	NO	H+ transporting ATP synthase O subunit isoform 1 [Danaus plexippus]
	Unigene9517	NO	PREDICTED: insulin-degrading enzyme-like isoform 2 [Nasonia vitripennis]
	Unigene952	NO	PREDICTED: acyl carrier protein, mitochondrial-like [Megachile rotundata]
	Unigene9582	NO	PREDICTED: talin-2-like [Bombus impatiens]
	Unigene959	NO	NADH dehydrogenase [ubiquinone] 1 beta subcomplex subunit 5, mitochondrial [Nasonia vitripennis]
	Unigene9659	NO	sarco/endoplasmic reticulum calcium ATPase [Bombyx mori]
	Unigene9782	NO	Segment polarity protein dishevelled-like protein DVL-3 [Harpegnathos saltator]
	Unigene991	NO	PREDICTED: NADH dehydrogenase [ubiquinone] 1 alpha subcomplex subunit 7-like [Megachile rotundata]
	Unigene9927	NO	Subunit VIb of cytochrome c oxidase [Danaus plexippus]
	Unigene9971	NO	hypothetical protein KGM_15724 [Danaus plexippus]
	Unigene9998	NO	PREDICTED: stress-activated protein kinase JNK-like [Megachile rotundata]
	CL124.Contig1	NO	succinate dehydrogenase cytochrome b560 subunit, mitochondrial [Nasonia vitripennis]
	CL334.Contig1	SP	Calcium-transporting ATPase sarcoplasmic/endoplasmic reticulum type [Acromyrmex echinatior]
	CL690.Contig1	SP	hypothetical protein SINV_04265 [Solenopsis invicta]
	CL690.Contig2	SP	GJ24134 [Drosophila virilis]
	Unigene12612	SP	Armadillo segment polarity protein [Harpegnathos saltator]
	Unigene1289	SP	cytochrome oxidase subunit I [Cotesia chilonis]
	Unigene758	SP	Protein toll [Harpegnathos saltator]
	CL238.Contig1	NO	PREDICTED: protein kinase shaggy-like isoform 2 [Nasonia vitripennis]
	CL238.Contig1	NO	PREDICTED: protein kinase shaggy-like isoform 2 [Nasonia vitripennis]
	CL238.Contig2	NO	PREDICTED: protein kinase shaggy-like isoform 2 [Nasonia vitripennis]
	CL238.Contig4	NO	Protein kinase shaggy [Acromyrmex echinatior]
	CL352.Contig1	NO	cytochrome c oxidase subunit I [Cotesia vestalis]
	CL352.Contig2	NO	cytochrome oxidase subunit I [Bucculatrix artemisiella]
	CL352.Contig3	NO	cytochrome c oxidase subunit I [Cotesia vestalis]
	CL51.Contig2	NO	hypothetical protein SINV_04265 [Solenopsis invicta]
	CL515.Contig1	NO	hypothetical protein SINV_06010 [Solenopsis invicta]
	CL515.Contig2	NO	hypothetical protein SINV_06010 [Solenopsis invicta]
	CL560.Contig1	NO	cytochrome b [Cotesia vestalis]
	CL597.Contig2	NO	cytochrome b [Cotesia vestalis]
	Unigene10083	NO	putative armadillo protein [Danaus plexippus]
	Unigene1012	NO	Cytochrome b-c1 complex subunit 2, mitochondrial [Camponotus floridanus]
	Unigene10223	NO	PREDICTED: ribosomal protein S6 kinase beta-1-like [Apis mellifera]
	Unigene1043	NO	PREDICTED: cytochrome c oxidase subunit 6C-like [Acyrthosiphon pisum]
	Unigene10590	NO	hypothetical protein KGM_04009 [Danaus plexippus]
	Unigene10828	NO	PREDICTED: calcium/calmodulin-dependent protein kinase type II alpha chain-like isoform 3 [Bombus terrestris]
	Unigene1089	NO	Interferon regulatory factor 2-binding protein 2-A [Harpegnathos saltator]
	Unigene11062	NO	Stress-activated protein kinase JNK [Acromyrmex echinatior]
	Unigene11200	NO	PREDICTED: rho-associated protein kinase 2-like [Bombus impatiens]
	Unigene11213	NO	Phosphatidylinositol 3-kinase regulatory subunit alpha [Camponotus floridanus]
	Unigene11447	NO	hypothetical protein SINV_14862 [Solenopsis invicta]
	Unigene11690	NO	PREDICTED: protein son of sevenless-like [Megachile rotundata]
	Unigene11808	NO	cytochrome c oxidase subunit II [Cotesia vestalis]
	Unigene12247	NO	PREDICTED: hypothetical protein LOC100743521 [Bombus impatiens]
	Unigene12735	NO	PREDICTED: armadillo segment polarity protein isoform 2 [Nasonia vitripennis]
	Unigene1310	NO	PREDICTED: troponin C, isoform 3 isoform 1 [Nasonia vitripennis]
	Unigene13656	NO	cytochrome oxidase subunit II [Xyrosaris lichneuta]
	Unigene1513	NO	conserved hypothetical protein [Pediculus humanus corporis]
	Unigene1894	NO	Ras-like GTP-binding protein Rho1 [Salmo salar]
	Unigene1925	NO	PREDICTED: cdc42 homolog isoform 1 [Nasonia vitripennis]
	Unigene2069	NO	PREDICTED: ras-like protein 1-like [Apis florea]
	Unigene2210	NO	PREDICTED: signal transducer and activator of transcription 5B-like [Megachile rotundata]
	Unigene2418	NO	PREDICTED: RAC serine/threonine-protein kinase-like [Bombus impatiens]
	Unigene2504	NO	hypothetical protein TRIADDRAFT_20496 [Trichoplax adhaerens]
	Unigene2544	NO	PREDICTED: uncharacterized protein LOC100879259 [Megachile rotundata]
	Unigene2630	NO	PREDICTED: ribosomal protein S6 kinase beta-1-like [Apis florea]
	Unigene2697	NO	cytochrome c oxidase polypeptide Vb [Bombyx mori]
	Unigene2903	NO	hypothetical protein KGM_05439 [Danaus plexippus]
	Unigene2926	NO	PREDICTED: calcium-transporting ATPase sarcoplasmic/endoplasmic reticulum type-like [Megachile rotundata]
	Unigene3005	NO	PREDICTED: calumenin-like [Nasonia vitripennis]

Protein synthesis and degradation	Unigene346	NO	GJ13165 [Drosophila virilis]
	Unigene372	NO	PREDICTED: hypothetical protein LOC100115623 isoform 1 [Nasonia vitripennis]
	Unigene383	NO	PREDICTED: cytochrome c-like isoform 1 [Bombus terrestris]
	Unigene4045	NO	Dual specificity mitogen-activated protein kinase kinase 4 [Acromyrmex echinatior]
	Unigene4101	NO	hypothetical protein SINV_05984 [Solenopsis invicta]
	Unigene411	NO	cytochrome c oxidase subunit VI [Microplitis mediator]
	Unigene4122	NO	mitochondrial cytochrome c oxidase subunit VIa [Bombyx mori]
	Unigene4156	NO	PREDICTED: rho-associated protein kinase 2-like isoform 2 [Nasonia vitripennis]
	Unigene426	NO	cytochrome c oxidase-like protein [Glyptapanteles flavicoxis]
	Unigene438	NO	cytochrome b-c1 complex subunit 9 [Nasonia vitripennis]
	Unigene4470	NO	Ras-like GTP-binding protein RHO [Lepeophtheirus salmonis]
	Unigene467	NO	hypothetical protein SINV_11135 [Solenopsis invicta]
	Unigene4710	NO	Dual specificity mitogen-activated protein kinase kinase 4 [Acromyrmex echinatior]
	Unigene4742	NO	PREDICTED: calmodulin-like protein 4-like isoform 1 [Bombus terrestris]
	Unigene4774	NO	mitochondrial cytochrome c [Bombyx mori]
	Unigene485	NO	AT15141p [Drosophila melanogaster]
	Unigene4885	NO	ubiquinol-cytochrome C reductase complex protein [Danaus plexippus]
	Unigene4920	NO	PREDICTED: mitogen-activated protein kinase 1-like [Megachile rotundata]
	Unigene520	NO	cytochrome c oxidase subunit 4 isoform 1, mitochondrial [Nasonia vitripennis]
	Unigene5515	NO	PREDICTED: armadillo segment polarity protein-like [Megachile rotundata]
	Unigene553	NO	PREDICTED: cytochrome b-c1 complex subunit 6, mitochondrial-like [Megachile rotundata]
	Unigene558	NO	AGAP008724-PA [Anopheles gambiae str. PEST]
	Unigene5630	NO	PREDICTED: rho-associated protein kinase 2-like isoform 2 [Nasonia vitripennis]
	Unigene579	NO	cytochrome c oxidase subunit VIb isoform 1 [Rhipicephalus sanguineus]
	Unigene6006	NO	PREDICTED: rho-associated protein kinase 2 [Apis florea]
	Unigene608	NO	hypothetical protein EAG_05916 [Camponotus floridanus]
	Unigene614	NO	Cytochrome b-c1 complex subunit Rieske, mitochondrial [Camponotus floridanus]
	Unigene6140	NO	Ras-like GTP-binding protein Rho1 [Danaus plexippus]
	Unigene6295	NO	PREDICTED: dual specificity mitogen-activated protein kinase kinase hemipterous-like isoform 2 [Megachile rotundata]
	Unigene6363	NO	Mitogen-activated protein kinase 1 [Harpegnathos saltator]
	Unigene647	NO	PREDICTED: cytochrome b-c1 complex subunit Rieske, mitochondrial-like [Cavia porcellus]
	Unigene663	NO	Cytochrome b-c1 complex subunit 7 [Camponotus floridanus]
	Unigene6630	NO	cytochrome c oxidase [Danaus plexippus]
	Unigene6671	NO	Ras-like GTP-binding protein RHO [Lepeophtheirus salmonis]
	Unigene669	NO	PREDICTED: protein enhancer of sevenless 2B-like [Nasonia vitripennis]
	Unigene7027	NO	AGAP005160-PA [Anopheles gambiae str. PEST]
	Unigene7100	NO	cytochrome c oxidase polypeptide IV [Bombyx mori]
	Unigene7102	NO	Multiple C2 and transmembrane domain-containing protein 2 [Acromyrmex echinatior]
	Unigene722	NO	PREDICTED: ras-like GTP-binding protein Rho1 [Nasonia vitripennis]
	Unigene7225	NO	PREDICTED: hypothetical protein LOC408577 [Apis mellifera]
	Unigene7671	NO	PREDICTED: rho-associated protein kinase 2 [Megachile rotundata]
	Unigene7714	NO	Mitogen-activated protein kinase 14B [Harpegnathos saltator]
	Unigene776	NO	AT15141p [Drosophila melanogaster]
	Unigene7916	NO	Dual specificity mitogen-activated protein kinase kinase 7 [Harpegnathos saltator]
	Unigene7980	NO	PREDICTED: dual specificity mitogen-activated protein kinase kinase 2-like [Apis florea]
	Unigene8196	NO	cytochrome c oxidase subunit III [Cotesia vestalis]
	Unigene8222	NO	ubiquinol-cytochrome C reductase complex 14kD subunit [Danaus plexippus]
	Unigene8344	NO	PREDICTED: phosphatidylinositol 3-kinase regulatory subunit alpha-like, partial [Apis florea]
	Unigene8427	NO	PREDICTED: rho-associated protein kinase 2 [Megachile rotundata]
	Unigene8488	NO	PREDICTED: uncharacterized protein LOC100877711 [Megachile rotundata]
	Unigene8515	NO	PREDICTED: hypothetical protein LOC100642907 [Bombus terrestris]
	Unigene8755	NO	PREDICTED: LOW QUALITY PROTEIN: ribosomal protein S6 kinase beta-1-like [Apis florea]
	Unigene8839	NO	Rho-associated protein kinase 2 [Camponotus floridanus]
	Unigene905	NO	PREDICTED: transcription factor AP-1-like [Megachile rotundata]
	Unigene912	NO	cytochrome c1, heme protein, mitochondrial [Nasonia vitripennis]
	Unigene9217	NO	PREDICTED: stress-activated protein kinase JNK-like [Megachile rotundata]
	Unigene9259	NO	PREDICTED: calcium/calmodulin-dependent protein kinase type II alpha chain-like isoform 1 [Bombus impatiens]
	Unigene9295	NO	PREDICTED: rho-associated protein kinase 2-like [Bombus impatiens]
	Unigene9359	NO	PREDICTED: hypothetical protein LOC100743083 [Bombus impatiens]

	Unigene9378	NO	PREDICTED: armadillo segment polarity protein-like [Megachile rotundata]
	Unigene9415	NO	PREDICTED: mitogen-activated protein kinase 14B-like isoform 2 [Megachile rotundata]
	Unigene9659	NO	sarco/endoplasmic reticulum calcium ATPase [Bombyx mori]
	Unigene975	NO	Growth hormone-inducible transmembrane protein [Camponotus floridanus]
	Unigene9927	NO	Subunit VIb of cytochrome c oxidase [Danaus plexippus]
	Unigene9971	NO	hypothetical protein KGM_15724 [Danaus plexippus]
	Unigene9998	NO	PREDICTED: stress-activated protein kinase JNK-like [Megachile rotundata]
	CL356.Contig2	SP	venom protein 2 [Microctonus hyperodae]
	CL557.Contig1	SP	PREDICTED: serine protease inhibitor 3/4-like [Bombus impatiens]
	CL690.Contig1	SP	hypothetical protein SINV_04265 [Solenopsis invicta]
	CL690.Contig2	SP	GJ24134 [Drosophila virilis]
	Unigene11423	SP	PREDICTED: LOW QUALITY PROTEIN: laminin subunit gamma-1-like [Megachile rotundata]
	Unigene152	SP	PREDICTED: serine protease inhibitor 3/4 [Nasonia vitripennis]
	Unigene154	SP	arrestin domain containing 4 [Danaus plexippus]
	Unigene1762	SP	PREDICTED: neuroserpin-like [Megachile rotundata]
	Unigene191	SP	PREDICTED: similar to Prg4 protein [Taeniopygia guttata]
	Unigene333	SP	PREDICTED: neuroserpin-like [Megachile rotundata]
	Unigene335	SP	venom protein 2 [Microctonus hyperodae]
	Unigene336	SP	venom protein 2 [Microctonus hyperodae]
	Unigene338	SP	venom protein 2 [Microctonus hyperodae]
	Unigene348	SP	venom protein 2 [Microctonus hyperodae]
	Unigene351	SP	venom protein 2 [Microctonus hyperodae]
	Unigene369	SP	PREDICTED: serine protease inhibitor 3/4-like [Bombus impatiens]
	Unigene3933	SP	conserved hypothetical protein [Culex quinquefasciatus]
	Unigene424	SP	PREDICTED: serine protease inhibitor 3/4 [Nasonia vitripennis]
	Unigene516	SP	PREDICTED: neuroserpin-like [Megachile rotundata]
	Unigene5497	SP	venom protein 2 [Microctonus hyperodae]
	Unigene569	SP	PREDICTED: neuroserpin-like [Megachile rotundata]
	Unigene645	SP	Leukocyte elastase inhibitor [Acromyrmex echinatior]
	Unigene6457	SP	cuticular protein RR-2 family member 59 precursor [Nasonia vitripennis]
	Unigene6575	SP	PREDICTED: serine protease inhibitor 3/4-like [Bombus impatiens]
	Unigene758	SP	Protein toll [Harpegnathos saltator]
	Unigene788	SP	PREDICTED: serine protease inhibitor 3/4 [Nasonia vitripennis]
	CL105.Contig1	NO	Transient receptor potential protein [Harpegnathos saltator]
	CL112.Contig1	NO	PREDICTED: histone H2A-like [Nasonia vitripennis]
	CL112.Contig2	NO	PREDICTED: histone H2A-like [Nasonia vitripennis]
	CL125.Contig1	NO	PREDICTED: histone H1C-like [Nasonia vitripennis]
	CL177.Contig2	NO	GI23529 [Drosophila mojavensis]
	CL207.Contig1	NO	scavenger receptor SR-C-like protein [Danaus plexippus]
	CL208.Contig1	NO	PREDICTED: ras-related protein Rab-5A-like isoform 1 [Nasonia vitripennis]
	CL208.Contig2	NO	PREDICTED: ras-related protein Rab-5A-like isoform 1 [Nasonia vitripennis]
	CL411.Contig1	NO	PREDICTED: similar to Histone H4 replacement CG3379-PC, partial [Hydra magnipapillata]
	CL411.Contig2	NO	PREDICTED: similar to Histone H4 replacement CG3379-PC, partial [Hydra magnipapillata]
	CL411.Contig3	NO	PREDICTED: histone H4 [Equus caballus]
	CL43.Contig2	NO	Histone H3.3 [Harpegnathos saltator]
	CL43.Contig4	NO	PREDICTED: hypothetical protein LOC704845 [Macaca mulatta]
	CL51.Contig2	NO	hypothetical protein SINV_04265 [Solenopsis invicta]
	CL534.Contig2	NO	venom protein 2 [Microctonus hyperodae]
	CL584.Contig1	NO	venom protein 2 [Microctonus hyperodae]
	CL603.Contig2	NO	venom protein 2 [Microctonus hyperodae]
	CL623.Contig1	NO	PREDICTED: histone H2B 1/2-like [Nasonia vitripennis]
	CL623.Contig2	NO	PREDICTED: histone H2B 1/2-like [Oreochromis niloticus]
	CL719.Contig1	NO	PREDICTED: histone H2A.V-like [Apis florea]
	Unigene10075	NO	Vinculin [Camponotus floridanus]
	Unigene10082	NO	type IV collagen [Bombyx mori]
	Unigene10165	NO	hypothetical protein KGM_15381 [Danaus plexippus]
	Unigene10329	NO	PREDICTED: vinculin-like isoform 2 [Bombus impatiens]
	Unigene10363	NO	GTP-binding protein alpha subunit, gna [Culex quinquefasciatus]
	Unigene10451	NO	putative collagen alpha-2IV chain protein [Danaus plexippus]
	Unigene10672	NO	putative collagen alpha-2IV chain protein [Danaus plexippus]
	Unigene10808	NO	PREDICTED: protein peanut-like [Nasonia vitripennis]
	Unigene10884	NO	hypothetical protein KGM_05418 [Danaus plexippus]

Tissue damage	Unigene10891	NO	putative laminin A chain [Danaus plexippus]
	Unigene11160	NO	hypothetical protein TcasGA2_TC014326 [Tribolium castaneum]
	Unigene11180	NO	small nuclear ribonucleoprotein Sm D1 [Bombyx mori]
	Unigene11213	NO	Phosphatidylinositol 3-kinase regulatory subunit alpha [Camponotus floridanus]
	Unigene11447	NO	hypothetical protein SINV_14862 [Solenopsis invicta]
	Unigene1152	NO	PREDICTED: leukocyte elastase inhibitor-like [Bombus impatiens]
	Unigene11586	NO	GL10309 [Drosophila persimilis]
	Unigene11667	NO	small nuclear ribonucleoprotein polypeptide [Bombyx mori]
	Unigene11795	NO	PREDICTED: vinculin-like [Megachile rotundata]
	Unigene12173	NO	PREDICTED: alaserpin-like [Bombus impatiens]
	Unigene12247	NO	PREDICTED: hypothetical protein LOC100743521 [Bombus impatiens]
	Unigene1299	NO	PREDICTED: similar to GA15301-PA [Tribolium castaneum]
	Unigene13165	NO	PREDICTED: alpha-actinin, sarcomeric-like [Megachile rotundata]
	Unigene13736	NO	hypothetical protein KGM_03021 [Danaus plexippus]
	Unigene1454	NO	Ras-related protein Rab-7a [Harpegnathos saltator]
	Unigene1484	NO	La autoantigen homolog [Cotesia congregata]
	Unigene1512	NO	venom protein 2 [Microctonus hyperodae]
	Unigene1513	NO	conserved hypothetical protein [Pediculus humanus corporis]
	Unigene1630	NO	small heat shock protein [Pteromalus puparum]
	Unigene1786	NO	PREDICTED: histone H2A-like isoform 6 [Apis mellifera]
	Unigene180	NO	PREDICTED: neuroserpin-like [Megachile rotundata]
	Unigene1879	NO	small ribonucleoprotein particle protein B [Apis mellifera]
	Unigene2023	NO	Small nuclear ribonucleoprotein Sm D3 [Harpegnathos saltator]
	Unigene2314	NO	venom protein 2 [Microctonus hyperodae]
	Unigene2504	NO	PREDICTED: histone H2A-like isoform 6 [Apis mellifera]
	Unigene2555	NO	Serpin B10 [Acromyrmex echinatior]
	Unigene2770	NO	CCR4-NOT transcription complex subunit 2 [Harpegnathos saltator]
	Unigene2940	NO	La autoantigen homolog [Cotesia congregata]
	Unigene3013	NO	serine protease inhibitor 4, serpin-4 [Aedes aegypti]
	Unigene332	NO	unnamed protein product [Macaca fascicularis]
	Unigene3501	NO	PREDICTED: antitrypsin-like [Apis florea]
	Unigene3626	NO	serine protease inhibitor 4 precursor [Bombyx mori]
	Unigene375	NO	PREDICTED: histone H3.3-like [Nomascus leucogenys]
	Unigene3817	NO	EH domain-binding protein 1 [Camponotus floridanus]
	Unigene4	NO	hypothetical protein KGM_05418 [Danaus plexippus]
	Unigene408	NO	PREDICTED: antitrypsin-like [Apis florea]
	Unigene4604	NO	hypothetical protein KGM_05418 [Danaus plexippus]
	Unigene461	NO	histone H3v [Euplotes octocarinatus]
	Unigene4620	NO	Protein AF-10 [Camponotus floridanus]
	Unigene5082	NO	serpin-4 [Bombyx mori]
	Unigene5220	NO	PREDICTED: la-related protein 1-like [Nasonia vitripennis]
	Unigene5526	NO	PREDICTED: protein toll [Nasonia vitripennis]
	Unigene5585	NO	PREDICTED: microtubule-actin cross-linking factor 1-like [Bombus impatiens]
	Unigene5619	NO	PREDICTED: probable small nuclear ribonucleoprotein Sm D1-like [Nasonia vitripennis]
	Unigene5634	NO	PREDICTED: uncharacterized protein LOC100882509 [Megachile rotundata]
	Unigene5654	NO	hypothetical protein KGM_04926 [Danaus plexippus]
	Unigene5846	NO	PREDICTED: vinculin-like [Megachile rotundata]
	Unigene6128	NO	seminal fluid protein CSSFP043 [Chilo suppressalis]
	Unigene6417	NO	PREDICTED: uncharacterized protein CG10915-like [Nasonia vitripennis]
	Unigene6513	NO	seminal fluid protein CSSFP043 [Chilo suppressalis]
	Unigene6790	NO	PREDICTED: hypothetical protein LOC100588064 [Nomascus leucogenys]
	Unigene6842	NO	guanine nucleotide binding protein alpha q polypeptide [Glossina morsitans morsitans]
	Unigene6897	NO	PREDICTED: uncharacterized protein LOC100870900 [Apis florea]
	Unigene6958	NO	seminal fluid protein CSSFP042 [Chilo suppressalis]
	Unigene71	NO	hypothetical protein EAI_14262 [Harpegnathos saltator]
	Unigene7102	NO	Multiple C2 and transmembrane domain-containing protein 2 [Acromyrmex echinatior]
	Unigene7225	NO	PREDICTED: hypothetical protein LOC408577 [Apis mellifera]
	Unigene7522	NO	PREDICTED: protein GDAP2 homolog [Apis florea]
	Unigene7541	NO	PREDICTED: antitrypsin-like [Apis florea]
	Unigene7893	NO	PREDICTED: LSM domain-containing protein 1-like [Megachile rotundata]
	Unigene8035	NO	PREDICTED: guanine nucleotide-binding protein G(s) subunit alpha-like [Megachile rotundata]
	Unigene8099	NO	PREDICTED: alaserpin-like [Bombus impatiens]
	Unigene8344	NO	PREDICTED: phosphatidylinositol 3-kinase regulatory subunit alpha-like, partial [Apis florea]

	Unigene8408	NO	putative laminin A chain [Danaus plexippus]
	Unigene8501	NO	PREDICTED: lupus La protein-like [Amphimedon queenslandica]
	Unigene8515	NO	PREDICTED: hypothetical protein LOC100642907 [Bombus terrestris]
	Unigene8702	NO	seminal fluid protein CSSFP045 [Chilo suppressalis]
	Unigene8718	NO	serine protease inhibitor 12 [Danaus plexippus]
	Unigene8772	NO	putative collagen alpha-2IV chain protein [Danaus plexippus]
	Unigene8948	NO	PREDICTED: vinculin-like [Megachile rotundata]
	Unigene9008	NO	PREDICTED: guanine nucleotide-binding protein G(q) subunit alpha-like isoform 5 [Megachile rotundata]
	Unigene9081	NO	putative laminin A chain [Danaus plexippus]
	Unigene9110	NO	PREDICTED: serine protease inhibitor 3/4 [Nasonia vitripennis]
	Unigene921	NO	histone H3 variant, putative [Trypanosoma cruzi]
	Unigene925	NO	PREDICTED: Golgi reassembly-stacking protein 1-like [Apis florea]
	Unigene9359	NO	PREDICTED: hypothetical protein LOC100743083 [Bombus impatiens]
	Unigene9405	NO	PREDICTED: neuroserpin-like [Megachile rotundata]
	Unigene9499	NO	hypothetical protein KGM_05418 [Danaus plexippus]
	Unigene9653	NO	PREDICTED: guanine nucleotide-binding protein G(s) subunit alpha-like [Megachile rotundata]
	Unigene9797	NO	PREDICTED: alpha-actinin, sarcomeric-like [Megachile rotundata]
	Unigene9818	NO	PREDICTED: uncharacterized protein LOC100875893 [Megachile rotundata]
	CL177.Contig1	NO	GI23529 [Drosophila mojavensis]
**Gonadal growth**	Unigene1366	SP	Protein 60A [Camponotus floridanus]
	Unigene3100	SP	PREDICTED: uncharacterized protein LOC100867171 [Apis florea]
	CL212.Contig1	NO	Cordon-bleu protein-like 1 [Harpegnathos saltator]
	CL481.Contig1	NO	ring box protein [Bombyx mori]
	CL515.Contig1	NO	hypothetical protein SINV_06010 [Solenopsis invicta]
	CL515.Contig2	NO	hypothetical protein SINV_06010 [Solenopsis invicta]
	CL624.Contig1	NO	S-phase kinase-associated protein [Danaus plexippus]
	CL624.Contig2	NO	S-phase kinase-associated protein [Danaus plexippus]
	CL663.Contig1	NO	Serine/threonine-protein phosphatase 2A 65 kDa regulatory subunit A alpha isoform [Camponotus floridanus]
	CL663.Contig2	NO	Serine/threonine-protein phosphatase 2A 65 kDa regulatory subunit A alpha isoform [Camponotus floridanus]
	CL757.Contig2	NO	PREDICTED: uncharacterized protein LOC100876927 [Megachile rotundata]
	Unigene1004	NO	hypothetical protein SINV_14598 [Solenopsis invicta]
	Unigene10223	NO	PREDICTED: ribosomal protein S6 kinase beta-1-like [Apis mellifera]
	Unigene10321	NO	PREDICTED: retinoblastoma-like protein 1-like isoform 2 [Bombus impatiens]
	Unigene10343	NO	PREDICTED: bone morphogenetic protein receptor type-1B-like, partial [Nasonia vitripennis]
	Unigene10365	NO	PREDICTED: activin receptor type-2B [Apis mellifera]
	Unigene10410	NO	Mothers against decapentaplegic-like protein 3 [Acromyrmex echinatior]
	Unigene1089	NO	Interferon regulatory factor 2-binding protein 2-A [Harpegnathos saltator]
	Unigene11200	NO	PREDICTED: rho-associated protein kinase 2-like [Bombus impatiens]
	Unigene11450	NO	conserved hypothetical protein [Pediculus humanus corporis]
	Unigene1216	NO	PREDICTED: transmembrane protein 208-like [Bombus impatiens]
	Unigene13553	NO	PREDICTED: protein BCL9 homolog [Apis florea]
	Unigene13568	NO	PREDICTED: protein mothers against dpp-like [Megachile rotundata]
	Unigene13635	NO	PREDICTED: LOW QUALITY PROTEIN: BTB/POZ domain-containing protein KCTD3-like [Nasonia vitripennis]
	Unigene1580	NO	hypothetical protein EAI_14262 [Harpegnathos saltator]
	Unigene1894	NO	Ras-like GTP-binding protein Rho1 [Salmo salar]
	Unigene2299	NO	PREDICTED: s-phase kinase-associated protein 1-like [Amphimedon queenslandica]
	Unigene2544	NO	PREDICTED: uncharacterized protein LOC100879259 [Megachile rotundata]
	Unigene2630	NO	PREDICTED: ribosomal protein S6 kinase beta-1-like [Apis florea]
	Unigene2925	NO	PREDICTED: hypothetical protein LOC409983 [Apis mellifera]
	Unigene2974	NO	PREDICTED: transcription factor Dp-1-like [Megachile rotundata]
	Unigene3188	NO	PREDICTED: serine/threonine-protein phosphatase 2A catalytic subunit beta isoform-like [Amphimedon queenslandica]
	Unigene3288	NO	Zinc finger FYVE domain-containing protein 19 [Harpegnathos saltator]
	Unigene3774	NO	PREDICTED: uncharacterized protein LOC100874971 [Megachile rotundata]
	Unigene4156	NO	PREDICTED: rho-associated protein kinase 2-like isoform 2 [Nasonia vitripennis]
	Unigene4303	NO	extra macrochaetae [Apis mellifera]
	Unigene4470	NO	Ras-like GTP-binding protein RHO [Lepeophtheirus salmonis]
	Unigene4717	NO	PREDICTED: cullin-1 isoform 2 [Megachile rotundata]
	Unigene4829	NO	PREDICTED: retinoblastoma-family protein [Apis mellifera]
	Unigene4920	NO	PREDICTED: mitogen-activated protein kinase 1-like [Megachile rotundata]

	Unigene5047	NO	PREDICTED: NFU1 iron-sulfur cluster scaffold homolog, mitochondrial-like [Bombus terrestris]
	Unigene5161	NO	PREDICTED: hypothetical protein LOC409983 [Apis mellifera]
	Unigene5630	NO	PREDICTED: rho-associated protein kinase 2-like isoform 2 [Nasonia vitripennis]
	Unigene6006	NO	PREDICTED: rho-associated protein kinase 2 [Apis florea]
	Unigene6140	NO	Ras-like GTP-binding protein Rho1 [Danaus plexippus]
	Unigene6354	NO	PREDICTED: similar to bone morphogenetic protein 5 preproprotein [Hydra magnipapillata]
	Unigene6363	NO	Mitogen-activated protein kinase 1 [Harpegnathos saltator]
	Unigene639	NO	conserved hypothetical protein [Pediculus humanus corporis]
	Unigene6671	NO	Ras-like GTP-binding protein RHO [Lepeophtheirus salmonis]
	Unigene6735	NO	hypothetical protein SINV_14598 [Solenopsis invicta]
	Unigene7027	NO	AGAP005160-PA [Anopheles gambiae str. PEST]
	Unigene7074	NO	PREDICTED: zinc finger FYVE domain-containing protein 9-like [Megachile rotundata]
	Unigene722	NO	PREDICTED: ras-like GTP-binding protein Rho1 [Nasonia vitripennis]
	Unigene7314	NO	Cullin-1 [Acromyrmex echinatior]
	Unigene7543	NO	hypothetical protein SINV_04935 [Solenopsis invicta]
	Unigene7671	NO	PREDICTED: rho-associated protein kinase 2 [Megachile rotundata]
	Unigene7818	NO	PREDICTED: LOW QUALITY PROTEIN: retinoblastoma-like protein 1-like [Apis florea]
	Unigene8266	NO	PREDICTED: mothers against decapentaplegic homolog 3 [Nasonia vitripennis]
	Unigene8427	NO	PREDICTED: rho-associated protein kinase 2 [Megachile rotundata]
	Unigene8581	NO	hypothetical protein SINV_13032 [Solenopsis invicta]
	Unigene8755	NO	PREDICTED: LOW QUALITY PROTEIN: ribosomal protein S6 kinase beta-1-like [Apis florea]
	Unigene8839	NO	Rho-associated protein kinase 2 [Camponotus floridanus]
	Unigene9295	NO	PREDICTED: rho-associated protein kinase 2-like [Bombus impatiens]
	Unigene9404	NO	Transcription factor E2F5 [Camponotus floridanus]
	Unigene9604	NO	PREDICTED: protein mothers against dpp-like [Megachile rotundata]
	Unigene975	NO	Growth hormone-inducible transmembrane protein [Camponotus floridanus]
	Unigene9768	NO	PREDICTED: LOW QUALITY PROTEIN: retinoblastoma-like protein 1-like [Apis florea]
	Unigene10121	NO	85 kDa calcium-independent phospholipase A2 [Camponotus floridanus]
	Unigene10184	NO	hypothetical protein DAPPUDRAFT_304184 [Daphnia pulex]
	Unigene10363	NO	GTP-binding protein alpha subunit, gna [Culex quinquefasciatus]
	Unigene10531	NO	phospholipase A2 precursor [Apis mellifera]
	Unigene10779	NO	matrix metalloproteinase 1 isoform 2 [Bombyx mori]
	Unigene10828	NO	PREDICTED: calcium/calmodulin-dependent protein kinase type II alpha chain-like isoform 3 [Bombus terrestris]
	Unigene11045	NO	matrix metalloproteinase 1 [Nasonia vitripennis]
	Unigene11062	NO	Stress-activated protein kinase JNK [Acromyrmex echinatior]
	Unigene11518	NO	PREDICTED: mitogen-activated protein kinase kinase kinase 4-like [Bombus terrestris]
	Unigene11598	NO	PREDICTED: group XIIA secretory phospholipase A2-like [Apis florea]
	Unigene11613	NO	PREDICTED: calcium-independent phospholipase A2-gamma-like [Bombus terrestris]
	Unigene11690	NO	PREDICTED: protein son of sevenless-like [Megachile rotundata]
	Unigene11993	NO	Mitogen-activated protein kinase kinase kinase 4 [Harpegnathos saltator]
	Unigene12641	NO	PREDICTED: LOW QUALITY PROTEIN: phospholipase D1-like [Bombus terrestris]
	Unigene1310	NO	PREDICTED: troponin C, isoform 3 isoform 1 [Nasonia vitripennis]
	Unigene13259	NO	PREDICTED: phospholipase D2-like [Apis florea]
	Unigene1925	NO	PREDICTED: cdc42 homolog isoform 1 [Nasonia vitripennis]
	Unigene1927	NO	PREDICTED: SH3 domain-binding glutamic acid-rich protein homolog [Megachile rotundata]
	Unigene2017	NO	hypothetical protein AaeL_AAEL006826 [Aedes aegypti]
	Unigene2069	NO	PREDICTED: ras-like protein 1-like [Apis florea]
	Unigene2504	NO	hypothetical protein TRIADDRAFT_20496 [Trichoplax adhaerens]
	Unigene3005	NO	PREDICTED: calumenin-like [Nasonia vitripennis]
	Unigene3861	NO	PREDICTED: group XIIA secretory phospholipase A2-like [Nasonia vitripennis]
	Unigene4045	NO	Dual specificity mitogen-activated protein kinase kinase 4 [Acromyrmex echinatior]
	Unigene4470	NO	Ras-like GTP-binding protein RHO [Lepeophtheirus salmonis]
	Unigene452	NO	PREDICTED: activating transcription factor of chaperone-like [Megachile rotundata]
	Unigene4710	NO	Dual specificity mitogen-activated protein kinase kinase 4 [Acromyrmex echinatior]
	Unigene4742	NO	PREDICTED: calmodulin-like protein 4-like isoform 1 [Bombus terrestris]
	Unigene485	NO	AT15141p [Drosophila melanogaster]
**Gonadotropins gene expression&secretion**	Unigene4920	NO	PREDICTED: mitogen-activated protein kinase 1-like [Megachile rotundata]

	Unigene4950	NO	PREDICTED: dual specificity mitogen-activated protein kinase kinase 6-like [Megachile rotundata]
	Unigene6295	NO	PREDICTED: dual specificity mitogen-activated protein kinase kinase hemipterous-like isoform 2 [Megachile rotundata]
	Unigene6363	NO	Mitogen-activated protein kinase 1 [Harpegnathos saltator]
	Unigene669	NO	PREDICTED: protein enhancer of sevenless 2B-like [Nasonia vitripennis]
	Unigene6842	NO	guanine nucleotide binding protein alpha q polypeptide [Glossina morsitans morsitans]
	Unigene7102	NO	Multiple C2 and transmembrane domain-containing protein 2 [Acromyrmex echinatior]
	Unigene7714	NO	Mitogen-activated protein kinase 14B [Harpegnathos saltator]
	Unigene776	NO	AT15141p [Drosophila melanogaster]
	Unigene7916	NO	Dual specificity mitogen-activated protein kinase kinase 7 [Harpegnathos saltator]
	Unigene7980	NO	PREDICTED: dual specificity mitogen-activated protein kinase kinase 2-like [Apis florea]
	Unigene8035	NO	PREDICTED: guanine nucleotide-binding protein G(s) subunit alpha-like [Megachile rotundata]
	Unigene8759	NO	Phospholipase D1 [Harpegnathos saltator]
	Unigene9008	NO	PREDICTED: guanine nucleotide-binding protein G(q) subunit alpha-like isoform 5 [Megachile rotundata]
	Unigene905	NO	PREDICTED: transcription factor AP-1-like [Megachile rotundata]
	Unigene9217	NO	PREDICTED: stress-activated protein kinase JNK-like [Megachile rotundata]
	Unigene9259	NO	PREDICTED: calcium/calmodulin-dependent protein kinase type II alpha chain-like isoform 1 [Bombus impatiens]
	Unigene9415	NO	PREDICTED: mitogen-activated protein kinase 14B-like isoform 2 [Megachile rotundata]
	Unigene9653	NO	PREDICTED: guanine nucleotide-binding protein G(s) subunit alpha-like [Megachile rotundata]
	Unigene9884	NO	PREDICTED: matrix metalloproteinase-14-like [Bombus terrestris]
	Unigene9998	NO	PREDICTED: stress-activated protein kinase JNK-like [Megachile rotundata]
	CL466.Contig1	NO	sugar transporter, putative [Ixodes scapularis]
	CL620.Contig1	NO	PREDICTED: hypothetical protein LOC100750102 [Bombus impatiens]
	CL620.Contig2	NO	PREDICTED: uncharacterized protein LOC100882269 [Megachile rotundata]
	CL763.Contig1	NO	PREDICTED: programmed cell death protein 6-like isoform 2 [Bombus terrestris]
	CL763.Contig2	NO	PREDICTED: programmed cell death protein 6-like isoform 2 [Bombus terrestris]
	Unigene10689	NO	Calcium-independent protein kinase C [Camponotus floridanus]
	Unigene11062	NO	Stress-activated protein kinase JNK [Acromyrmex echinatior]
	Unigene11085	NO	Acetyl-CoA carboxylase [Harpegnathos saltator]
	Unigene12285	NO	PREDICTED: LOW QUALITY PROTEIN: acetyl-CoA carboxylase-like [Apis florea]
	Unigene12286	NO	Acetyl-CoA carboxylase [Acromyrmex echinatior]
	Unigene12545	NO	PREDICTED: acetyl-CoA carboxylase-like [Bombus impatiens]
	Unigene1315	NO	nuclear receptor RXR-l [Locusta migratoria]
	Unigene13532	NO	PREDICTED: serine/threonine-protein kinase mTOR [Megachile rotundata]
	Unigene13723	NO	Acetyl-CoA carboxylase [Acromyrmex echinatior]
	Unigene1838	NO	ADIPOR-like receptor CG5315 [Harpegnathos saltator]
	Unigene2043	NO	PREDICTED: hypothetical protein LOC100119908 [Nasonia vitripennis]
	Unigene2194	NO	Long-chain-fatty-acid--CoA ligase 3 [Camponotus floridanus]
	Unigene2373	NO	Carnitine O-palmitoyltransferase I, liver isoform [Camponotus floridanus]
	Unigene2402	NO	PREDICTED: very long-chain-fatty-acid--CoA ligase bubblegum-like [Nasonia vitripennis]
	Unigene2418	NO	PREDICTED: RAC serine/threonine-protein kinase-like [Bombus impatiens]
	Unigene2470	NO	PREDICTED: long-chain-fatty-acid--CoA ligase 3-like [Megachile rotundata]
	Unigene3141	NO	Long-chain-fatty-acid--CoA ligase ACSBG2 [Camponotus floridanus]
	Unigene3344	NO	PREDICTED: solute carrier family 2, facilitated glucose transporter member 1-like [Nasonia vitripennis]
	Unigene3438	NO	5'-AMP-activated protein kinase subunit beta-2 [Harpegnathos saltator]
	Unigene3484	NO	Insulin receptor substrate 1-B [Harpegnathos saltator]
	Unigene3570	NO	Hypothetical protein CBG06417 [Caenorhabditis briggsae]
	Unigene3628	NO	PREDICTED: uncharacterized protein LOC100879231 [Megachile rotundata]
	Unigene3643	NO	PREDICTED: uncharacterized protein LOC100882269 [Megachile rotundata]
	Unigene3844	NO	Insulin receptor substrate 1-B [Camponotus floridanus]
	Unigene5332	NO	RecName: Full=Probable serine/threonine-protein kinase mkcD; AltName: Full=MAP kinase cascade D
	Unigene5608	NO	PREDICTED: tyrosine-protein phosphatase corkscrew [Apis mellifera]
	Unigene592	NO	Insulin receptor substrate 1 [Acromyrmex echinatior]
	Unigene6215	NO	4-coumarate--CoA ligase 2 [Harpegnathos saltator]
	Unigene6226	NO	Carnitine O-palmitoyltransferase I, liver isoform [Harpegnathos saltator]
	Unigene6441	NO	unnamed protein product [Homo sapiens]

**Insulin resistance**	Unigene7700	NO	PREDICTED: acetyl-CoA carboxylase-like isoform 2 [Bombus terrestris]
	Unigene7702	NO	PREDICTED: uncharacterized protein LOC100870622 [Apis florea]
	Unigene8488	NO	PREDICTED: uncharacterized protein LOC100877711 [Megachile rotundata]
	Unigene8920	NO	PREDICTED: acyl-CoA synthetase family member 3, mitochondrial-like isoform 1 [Nasonia vitripennis]
	Unigene9201	NO	PREDICTED: 5'-AMP-activated protein kinase catalytic subunit alpha-2-like [Bombus impatiens]
	Unigene9217	NO	PREDICTED: stress-activated protein kinase JNK-like [Megachile rotundata]
	Unigene9998	NO	PREDICTED: stress-activated protein kinase JNK-like [Megachile rotundata]
	CL182.Contig1	NO	pyruvate kinase [Trypanosoma brucei]
	CL575.Contig1	NO	PREDICTED: pyruvate kinase isoform 2 [Nasonia vitripennis]
	CL575.Contig2	NO	PREDICTED: pyruvate kinase isoform 1 [Nasonia vitripennis]
	CL575.Contig3	NO	PREDICTED: pyruvate kinase isoform 2 [Nasonia vitripennis]
	CL575.Contig4	NO	PREDICTED: pyruvate kinase isoform 1 [Nasonia vitripennis]
	Unigene1039	NO	Sugar transporter ERD6-like 6 [Harpegnathos saltator]
	Unigene11213	NO	Phosphatidylinositol 3-kinase regulatory subunit alpha [Camponotus floridanus]
	Unigene11378	NO	insulin receptor-1 [Solenopsis invicta]
	Unigene11447	NO	hypothetical protein SINV_14862 [Solenopsis invicta]
	Unigene11834	NO	PREDICTED: insulin-like peptide receptor-like [Bombus terrestris]
	Unigene12247	NO	PREDICTED: hypothetical protein LOC100743521 [Bombus impatiens]
	Unigene12766	NO	hexokinase [Biston betularia]
	Unigene12937	NO	insulin receptor-2 [Solenopsis invicta]
	Unigene1302	NO	PREDICTED: protein kinase C iota type isoform 1 [Apis mellifera]
	Unigene13525	NO	Insulin receptor [Camponotus floridanus]
	Unigene1432	NO	PREDICTED: hexokinase type 2-like isoform 1 [Nasonia vitripennis]
	Unigene1479	NO	hypothetical protein SINV_14712 [Solenopsis invicta]
	Unigene1559	NO	Solute carrier family 2, facilitated glucose transporter member 8 [Acromyrmex echinatior]
	Unigene1726	NO	Hexokinase-2 [Acromyrmex echinatior]
	Unigene2494	NO	PREDICTED: hexokinase type 2-like isoform 2 [Nasonia vitripennis]
	Unigene3213	NO	Protein kinase C, zeta [Mus musculus]
	Unigene4920	NO	PREDICTED: mitogen-activated protein kinase 1-like [Megachile rotundata]
	Unigene6363	NO	Mitogen-activated protein kinase 1 [Harpegnathos saltator]
	Unigene7225	NO	PREDICTED: hypothetical protein LOC408577 [Apis mellifera]
	Unigene8344	NO	PREDICTED: phosphatidylinositol 3-kinase regulatory subunit alpha-like, partial [Apis florea]
	Unigene9359	NO	PREDICTED: hypothetical protein LOC100743083 [Bombus impatiens]
	Unigene9641	NO	PREDICTED: LOW QUALITY PROTEIN: phosphatidylinositol-4,5-bisphosphate 3-kinase catalytic subunit delta isoform-like [Apis florea]
**Juvenile hormine regulation**	Unigene3237	SP	Juvenile hormone esterase [Camponotus floridanus]
	Unigene665	SP	PREDICTED: esterase FE4 [Nasonia vitripennis]
	Unigene7763	SP	--
	Unigene8624	SP	Juvenile hormone esterase binding protein [Nasonia vitripennis]
	CL525.Contig2	NO	Cytochrome P450 306a1 [Camponotus floridanus]
	Unigene10920	NO	3-chlorobenzoate-3,4-dioxygenase oxygenase subunit [Camponotus floridanus]
	Unigene1201	NO	PREDICTED: ecdysone 20-monooxygenase-like [Acyrthosiphon pisum]
	Unigene12917	NO	PREDICTED: venom carboxylesterase-6-like [Apis florea]
	Unigene13155	NO	Cytochrome P450 302a1, mitochondrial [Acromyrmex echinatior]
	Unigene1754	NO	Ecdysone biosynthesis protein [Daphnia pulex]
	Unigene3698	NO	PREDICTED: cytochrome P450 315a1, mitochondrial-like [Apis florea]
	Unigene4264	NO	3-chlorobenzoate-3,4-dioxygenase oxygenase subunit [Camponotus floridanus]
	Unigene4703	NO	PREDICTED: cytochrome P450 18a1-like [Bombus impatiens]
	Unigene725	NO	Chlorophyllide a oxygenase, chloroplastic [Harpegnathos saltator]
	Unigene7431	NO	Cytochrome P450 302a1, mitochondrial [Harpegnathos saltator]
	Unigene2607	NO	PREDICTED: glyoxalase domain-containing protein 4-like [Megachile rotundata]
	Unigene19005	NO	
	CL525.Contig1	NO	Cytochrome P450 306a1 [Camponotus floridanus]
	Unigene3601	SP	Ecdysone-inducible gene E1
	Unigene384	SP	Ecdysteroid regulated 16 kDa
	Unigene665	SP	PREDICTED: esterase FE4 [Nasonia vitripennis]
	Unigene8624	SP	PREDICTED: esterase FE4-like isoform 2 [Bombus terrestris]
	CL525.Contig2	NO	Cytochrome P450 306a1 [Camponotus floridanus]
	Unigene10920	NO	3-chlorobenzoate-3,4-dioxygenase oxygenase subunit [Camponotus floridanus]
	Unigene1201	NO	PREDICTED: ecdysone 20-monooxygenase-like [Acyrthosiphon pisum]
	Unigene12917	NO	PREDICTED: venom carboxylesterase-6-like [Apis florea]
	Unigene13155	NO	Cytochrome P450 302a1, mitochondrial [Acromyrmex echinatior]

Molting hormone regulation	Unigene1754	NO	Ecdysone biosynthesis protein [Daphnia pulex]
	Unigene3698	NO	PREDICTED: cytochrome P450 315a1, mitochondrial-like [Apis florea]
	Unigene4264	NO	3-chlorobenzoate-3,4-dioxygenase oxygenase subunit [Camponotus floridanus]
	Unigene4703	NO	PREDICTED: cytochrome P450 18a1-like [Bombus impatiens]
	Unigene725	NO	Chlorophyllide a oxygenase, chloroplastic [Harpegnathos saltator]
	Unigene7431	NO	Cytochrome P450 302a1, mitochondrial [Harpegnathos saltator]
	Unigene10540	NO	Ecdysteroid-regulated gene E74 [Apis mellifera]
	Unigene3153	NO	PREDICTED: ecdysone-inducible protein E75-like [Megachile rotundata]
	Unigene3417	NO	Ecdysone-induced protein 75
	Unigene3653	NO	Ecdysone-induced protein 75
	Unigene1201	NO	PREDICTED: ecdysone 20-monooxygenase-like [Acyrthosiphon pisum]
	Unigene10540	NO	Ecdysteroid-regulated gene E74 [Apis mellifera]
	CL525.Contig1	NO	Cytochrome P450 306a1 [Camponotus floridanus]
Neuronal growth	CL363.Contig1	NO	PREDICTED: serine/threonine-protein phosphatase 2B catalytic subunit 2-like [Apis florea]
	CL363.Contig2	NO	PREDICTED: serine/threonine-protein phosphatase 2B catalytic subunit 2-like [Apis florea]
	CL375.Contig1	NO	guanine nucleotide-binding protein subunit beta-like [Microplitis mediator]
	CL375.Contig2	NO	guanine nucleotide-binding protein subunit beta-like [Microplitis mediator]
	CL735.Contig2	NO	G protein-coupled receptor kinase 1 [Camponotus floridanus]
	Unigene10081	NO	PREDICTED: glutamate receptor, ionotropic kainate 2-like [Megachile rotundata]
	Unigene10121	NO	85 kDa calcium-independent phospholipase A2 [Camponotus floridanus]
	Unigene10184	NO	hypothetical protein DAPPUDRAFT_304184 [Daphnia pulex]
	Unigene10363	NO	GTP-binding protein alpha subunit, gna [Culex quinquefasciatus]
	Unigene10531	NO	phospholipase A2 precursor [Apis mellifera]
	Unigene11087	NO	PREDICTED: guanine nucleotide-binding protein G(i) subunit alpha-like [Megachile rotundata]
	Unigene11230	NO	Sorting nexin-27 [Acromyrmex echinatior]
	Unigene11557	NO	PREDICTED: metabotropic glutamate receptor 7-like [Bombus terrestris]
	Unigene11598	NO	PREDICTED: group XIIA secretory phospholipase A2-like [Apis florea]
	Unigene11613	NO	PREDICTED: calcium-independent phospholipase A2-gamma-like [Bombus terrestris]
	Unigene11659	NO	GTP-binding protein (i) alpha subunit, gnai [Aedes aegypti]
	Unigene12049	NO	PREDICTED: glutaminase kidney isoform, mitochondrial-like isoform 2 [Bombus impatiens]
	Unigene1213	NO	PREDICTED: calcineurin subunit B type 2-like isoform 1 [Nasonia vitripennis]
	Unigene12299	NO	Homer protein-like protein 2 [Acromyrmex echinatior]
	Unigene12641	NO	PREDICTED: LOW QUALITY PROTEIN: phospholipase D1-like [Bombus terrestris]
	Unigene13259	NO	PREDICTED: phospholipase D2-like [Apis florea]
	Unigene1927	NO	PREDICTED: SH3 domain-binding glutamic acid-rich protein homolog [Megachile rotundata]
	Unigene2017	NO	hypothetical protein AaeL_AAEL006826 [Aedes aegypti]
	Unigene2152	NO	Guanine nucleotide-binding protein subunit gamma-1 [Camponotus floridanus]
	Unigene2339	NO	Guanine nucleotide-binding protein subunit gamma-e [Harpegnathos saltator]
	Unigene2504	NO	hypothetical protein TRIADDRAFT_20496 [Trichoplax adhaerens]
	Unigene3316	NO	PREDICTED: calcium-binding protein p22-like [Megachile rotundata]
	Unigene3692	NO	PREDICTED: LOW QUALITY PROTEIN: G protein-coupled receptor kinase 1-like [Apis florea]
	Unigene3820	NO	PREDICTED: vesicular glutamate transporter 3-like [Bombus terrestris]
	Unigene3861	NO	PREDICTED: group XIIA secretory phospholipase A2-like [Nasonia vitripennis]
	Unigene3961	NO	PREDICTED: glutamine synthetase 2 cytoplasmic-like isoform 1 [Bombus terrestris]
	Unigene4920	NO	PREDICTED: mitogen-activated protein kinase 1-like [Megachile rotundata]
	Unigene6363	NO	Mitogen-activated protein kinase 1 [Harpegnathos saltator]
	Unigene6842	NO	guanine nucleotide binding protein alpha q polypeptide [Glossina morsitans morsitans]
	Unigene7102	NO	Multiple C2 and transmembrane domain-containing protein 2 [Acromyrmex echinatior]
	Unigene7141	NO	Glutamine synthetase 2 cytoplasmic [Camponotus floridanus]
	Unigene7820	NO	PREDICTED: glutaminase kidney isoform, mitochondrial-like [Nasonia vitripennis]
	Unigene8035	NO	PREDICTED: guanine nucleotide-binding protein G(s) subunit alpha-like [Megachile rotundata]
	Unigene8190	NO	PREDICTED: homer protein homolog 1-like [Megachile rotundata]
	Unigene8714	NO	PREDICTED: G protein-coupled receptor kinase 1-like [Megachile rotundata]
	Unigene8759	NO	Phospholipase D1 [Harpegnathos saltator]
	Unigene9008	NO	PREDICTED: guanine nucleotide-binding protein G(q) subunit alpha-like isoform 5 [Megachile rotundata]
	Unigene9529	NO	PREDICTED: hypothetical protein LOC100741120 [Bombus impatiens]
	Unigene9653	NO	PREDICTED: guanine nucleotide-binding protein G(s) subunit alpha-like [Megachile rotundata]

	CL334.Contig1	SP	Calcium-transporting ATPase sarcoplasmic/endoplasmic reticulum type [Acromyrmex echinatior]
	CL430.Contig2	SP	PREDICTED: tenascin-X-like [Nasonia vitripennis]
	CL590.Contig1	SP	PREDICTED: serine/threonine-protein kinase/endoribonuclease IRE1-like [Nasonia vitripennis]
	Unigene1011	SP	PREDICTED: membrane metallo-endopeptidase-like 1-like [Bombus impatiens]
	Unigene11423	SP	PREDICTED: LOW QUALITY PROTEIN: laminin subunit gamma-1-like [Megachile rotundata]
	Unigene1289	SP	cytochrome oxidase subunit I [Cotesia chilonis]
	Unigene2376	SP	hypothetical protein TcasGA2_TC010888 [Tribolium castaneum]
	Unigene2802	SP	PREDICTED: serine/threonine-protein kinase/endoribonuclease IRE1-like [Megachile rotundata]
	Unigene3097	SP	PREDICTED: lipase member H-A-like [Apis mellifera]
	Unigene3500	SP	PREDICTED: serine/threonine-protein kinase/endoribonuclease IRE1-like [Nasonia vitripennis]
	Unigene4476	SP	hypothetical protein SINV_04265 [Solenopsis invicta]
	Unigene4608	SP	Serine/threonine-protein kinase/endoribonuclease ire-1 [Camponotus floridanus]
	Unigene6037	SP	PREDICTED: serine/threonine-protein kinase/endoribonuclease IRE1-like [Nasonia vitripennis]
	Unigene6049	SP	hypothetical protein SINV_07475 [Solenopsis invicta]
	CL205.Contig1	NO	PREDICTED: NADH dehydrogenase [ubiquinone] flavoprotein 1, mitochondrial-like [Apis florea]
	CL205.Contig2	NO	PREDICTED: NADH dehydrogenase [ubiquinone] flavoprotein 1, mitochondrial [Apis mellifera]
	CL231.Contig1	NO	ATP synthase subunit alpha, mitochondrial [Harpegnathos saltator]
	CL238.Contig1	NO	PREDICTED: protein kinase shaggy-like isoform 2 [Nasonia vitripennis]
	CL238.Contig2	NO	PREDICTED: protein kinase shaggy-like isoform 2 [Nasonia vitripennis]
	CL238.Contig4	NO	Protein kinase shaggy [Acromyrmex echinatior]
	CL352.Contig1	NO	cytochrome c oxidase subunit I [Cotesia vestalis]
	CL352.Contig2	NO	cytochrome oxidase subunit I [Bucculatrix artemisiella]
	CL352.Contig3	NO	cytochrome c oxidase subunit I [Cotesia vestalis]
	CL363.Contig1	NO	PREDICTED: serine/threonine-protein phosphatase 2B catalytic subunit 2-like [Apis florea]
	CL363.Contig2	NO	PREDICTED: serine/threonine-protein phosphatase 2B catalytic subunit 2-like [Apis florea]
	CL374.Contig1	NO	ATP synthase subunit beta, mitochondrial [Harpegnathos saltator]
	CL374.Contig2	NO	ATP-synthase subunit beta [Schistocerca gregaria]
	CL374.Contig3	NO	ATP synthase subunit beta, putative [Pediculus humanus corporis]
	CL401.Contig2	NO	PREDICTED: ATP synthase subunit epsilon, mitochondrial-like isoform 1 [Apis mellifera]
	CL412.Contig1	NO	hypothetical protein SINV_01823 [Solenopsis invicta]
	CL421.Contig2	NO	PREDICTED: glyceraldehyde-3-phosphate dehydrogenase 2 [Nasonia vitripennis]
	CL483.Contig1	NO	PREDICTED: hypothetical protein LOC100743933 [Bombus impatiens]
	CL483.Contig2	NO	Microtubule-associated protein 2 [Harpegnathos saltator]
	CL51.Contig1	NO	hypothetical protein SINV_04265 [Solenopsis invicta]
	CL560.Contig1	NO	cytochrome b [Cotesia vestalis]
	CL597.Contig2	NO	cytochrome b [Cotesia vestalis]
	CL740.Contig1	NO	PREDICTED: NADH dehydrogenase [ubiquinone] flavoprotein 1, mitochondrial-like [Megachile rotundata]
	CL750.Contig1	NO	PREDICTED: ATP synthase lipid-binding protein, mitochondrial-like [Megachile rotundata]
	Unigene1012	NO	Cytochrome b-c1 complex subunit 2, mitochondrial [Camponotus floridanus]
	Unigene1014	NO	PREDICTED: NADH dehydrogenase [ubiquinone] iron-sulfur protein 7, mitochondrial-like [Apis florea]
	Unigene10363	NO	GTP-binding protein alpha subunit, gna [Culex quinquefasciatus]
	Unigene1043	NO	PREDICTED: cytochrome c oxidase subunit 6C-like [Acyrthosiphon pisum]
	Unigene1044	NO	GF11341 [Drosophila ananassae]
	Unigene10590	NO	hypothetical protein KGM_04009 [Danaus plexippus]
	Unigene1074	NO	NADH dehydrogenase [ubiquinone] 1 alpha subcomplex subunit 13 [Camponotus floridanus]
	Unigene10847	NO	PREDICTED: nicastrin-like [Megachile rotundata]
	Unigene10891	NO	putative laminin A chain [Danaus plexippus]
	Unigene1090	NO	PREDICTED: similar to NADH:ubiquinone dehydrogenase, putative [Tribolium castaneum]
	Unigene11124	NO	Presenilin-like protein [Harpegnathos saltator]
	Unigene11156	NO	PREDICTED: nicastrin [Apis mellifera]

Neuronal injury	Unigene1120	NO	PREDICTED: endothelin-converting enzyme 1-like [Nasonia vitripennis]
	Unigene11731	NO	GE14316 [Drosophila yakuba]
	Unigene11735	NO	PREDICTED: eukaryotic translation initiation factor 2-alpha kinase-like [Bombus terrestris]
	Unigene11808	NO	cytochrome c oxidase subunit II [Cotesia vestalis]
	Unigene1213	NO	PREDICTED: calcineurin subunit B type 2-like isoform 1 [Nasonia vitripennis]
	Unigene12162	NO	PREDICTED: disintegrin and metalloproteinase domain-containing protein 10-like [Megachile rotundata]
	Unigene12335	NO	PREDICTED: insulin-degrading enzyme-like isoform 2 [Nasonia vitripennis]
	Unigene1243	NO	GI19985 [Drosophila mojavensis]
	Unigene1263	NO	PREDICTED: microtubule-associated protein tau-like [Apis florea]
	Unigene12807	NO	PREDICTED: disintegrin and metalloproteinase domain-containing protein 10-like [Megachile rotundata]
	Unigene1290	NO	PREDICTED: succinate dehydrogenase [ubiquinone] flavoprotein subunit, mitochondrial-like [Megachile rotundata]
	Unigene13000	NO	PREDICTED: gamma-secretase subunit pen-2-like [Megachile rotundata]
	Unigene1310	NO	PREDICTED: troponin C, isoform 3 isoform 1 [Nasonia vitripennis]
	Unigene1327	NO	hypothetical protein SINV_09347 [Solenopsis invicta]
	Unigene13656	NO	cytochrome oxidase subunit II [Xyrosaris lichneuta]
	Unigene1371	NO	NADH dehydrogenase [ubiquinone] 1 beta subcomplex subunit 8, mitochondrial [Nasonia vitripennis]
	Unigene1404	NO	hypothetical protein AND_29000 [Anopheles darlingi]
	Unigene1440	NO	NADH dehydrogenase (ubiquinone) 1 beta subcomplex, 7 [Tribolium castaneum]
	Unigene1447	NO	NADH dehydrogenase [ubiquinone] iron-sulfur protein 6, mitochondrial [Nasonia vitripennis]
	Unigene1473	NO	hypothetical protein EAI_14262 [Harpegnathos saltator]
	Unigene1513	NO	conserved hypothetical protein [Pediculus humanus corporis]
	Unigene1546	NO	PREDICTED: hypothetical protein LOC100119331 [Nasonia vitripennis]
	Unigene1574	NO	AGAP009602-PA [Anopheles gambiae str. PEST]
	Unigene1580	NO	hypothetical protein EAI_14262 [Harpegnathos saltator]
	Unigene1611	NO	PREDICTED: probable NADH dehydrogenase [ubiquinone] 1 alpha subcomplex subunit 12-like [Megachile rotundata]
	Unigene1633	NO	NADH dehydrogenase [ubiquinone] iron-sulfur protein 8, mitochondrial [Nasonia vitripennis]
	Unigene1646	NO	NADH dehydrogenase [Culex quinquefasciatus]
	Unigene1653	NO	AGAP012374-PA [Anopheles gambiae str. PEST]
	Unigene1688	NO	GD20916 [Drosophila simulans]
	Unigene1736	NO	AGAP009824-PA [Anopheles gambiae str. PEST]
	Unigene1738	NO	succinate dehydrogenase iron sulfur subunit B [Lysiphlebus testaceipes]
	Unigene1749	NO	NADH dehydrogenase [ubiquinone] 1 beta subcomplex subunit 2, mitochondrial [Camponotus floridanus]
	Unigene1756	NO	3-hydroxyacyl-CoA dehydrogenase type-2 [Camponotus floridanus]
	Unigene1800	NO	NADH dehydrogenase (ubiquinone) 1 alpha subcomplex, 9, 39kDa [Nasonia vitripennis]
	Unigene1833	NO	NADH dehydrogenase [ubiquinone] 1 alpha subcomplex subunit 2 [Camponotus floridanus]
	Unigene1894	NO	Ras-like GTP-binding protein Rho1 [Salmo salar]
	Unigene2076	NO	--
	Unigene2158	NO	PREDICTED: WD repeat-containing protein 55 homolog [Nasonia vitripennis]
	Unigene2224	NO	NADH dehydrogenase [Lysiphlebus testaceipes]
	Unigene2244	NO	AGAP006918-PA [Anopheles gambiae str. PEST]
	Unigene2346	NO	NADH dehydrogenase [ubiquinone] flavoprotein 2, mitochondrial [Nasonia vitripennis]
	Unigene2469	NO	Cell division protein kinase 5 [Harpegnathos saltator]
	Unigene2537	NO	ATP synthase subunit c [Manduca sexta]
	Unigene2610	NO	hypothetical protein SINV_04265 [Solenopsis invicta]
	Unigene2697	NO	cytochrome c oxidase polypeptide Vb [Bombyx mori]
	Unigene2856	NO	succinate dehydrogenase [ubiquinone] cytochrome b small subunit, mitochondrial [Nasonia vitripennis]
	Unigene2903	NO	hypothetical protein KGM_05439 [Danaus plexippus]
	Unigene2926	NO	PREDICTED: calcium-transporting ATPase sarcoplasmic/endoplasmic reticulum type-like [Megachile rotundata]
	Unigene2956	NO	hypothetical protein SINV_02345 [Solenopsis invicta]
	Unigene3005	NO	PREDICTED: calumenin-like [Nasonia vitripennis]
	Unigene3257	NO	PREDICTED: hypothetical protein LOC100122162 [Nasonia vitripennis]
	Unigene3316	NO	PREDICTED: calcium-binding protein p22-like [Megachile rotundata]
	Unigene3360	NO	Low-density lipoprotein receptor-related protein 1B [Camponotus floridanus]

Unigene3376	NO	PREDICTED: hypothetical protein LOC100745000 [Bombus impatiens]
Unigene3405	NO	ATP synthase [Bombyx mori]
Unigene346	NO	GJ13165 [Drosophila virilis]
Unigene372	NO	PREDICTED: hypothetical protein LOC100115623 isoform 1 [Nasonia vitripennis]
Unigene3754	NO	PREDICTED: hypothetical protein LOC100122162 [Nasonia vitripennis]
Unigene383	NO	PREDICTED: cytochrome c-like isoform 1 [Bombus terrestris]
Unigene3882	NO	PREDICTED: NADH dehydrogenase [ubiquinone] iron-sulfur protein 4, mitochondrial-like [Megachile rotundata]
Unigene4061	NO	hypothetical protein AND_01489 [Anopheles darlingi]
Unigene4066	NO	PREDICTED: uncharacterized protein LOC100875487 [Megachile rotundata]
Unigene4072	NO	hypothetical protein KGM_03909 [Danaus plexippus]
Unigene4107	NO	hypothetical protein SINV_09114 [Solenopsis invicta]
Unigene411	NO	cytochrome c oxidase subunit VI [Microplitis mediator]
Unigene412	NO	hypothetical protein SINV_01823 [Solenopsis invicta]
Unigene4122	NO	mitochondrial cytochrome c oxidase subunit VIa [Bombyx mori]
Unigene426	NO	cytochrome c oxidase-like protein [Glyptapanteles flavicoxis]
Unigene4333	NO	H+ transporting ATP synthase gamma subunit [Danaus plexippus]
Unigene438	NO	cytochrome b-c1 complex subunit 9 [Nasonia vitripennis]
Unigene4442	NO	PREDICTED: insulin-degrading enzyme-like isoform 2 [Nasonia vitripennis]
Unigene4535	NO	hypothetical protein SINV_09114 [Solenopsis invicta]
Unigene4584	NO	PREDICTED: NADH-ubiquinone oxidoreductase 75 kDa subunit, mitochondrial-like [Bombus terrestris]
Unigene4589	NO	Low-density lipoprotein receptor-related protein 1 [Harpegnathos saltator]
Unigene467	NO	hypothetical protein SINV_11135 [Solenopsis invicta]
Unigene4742	NO	PREDICTED: calmodulin-like protein 4-like isoform 1 [Bombus terrestris]
Unigene4774	NO	mitochondrial cytochrome c [Bombyx mori]
Unigene485	NO	AT15141p [Drosophila melanogaster]
Unigene4852	NO	hypothetical protein KGM_08437 [Danaus plexippus]
Unigene4885	NO	ubiquinol-cytochrome C reductase complex protein [Danaus plexippus]
Unigene4920	NO	PREDICTED: mitogen-activated protein kinase 1-like [Megachile rotundata]
Unigene4950	NO	PREDICTED: dual specificity mitogen-activated protein kinase kinase 6-like [Megachile rotundata]
Unigene4959	NO	ATP synthase [Danaus plexippus]
Unigene5043	NO	PREDICTED: hypothetical protein LOC100122162 [Nasonia vitripennis]
Unigene5072	NO	putative NADH:ubiquinone dehydrogenase [Danaus plexippus]
Unigene517	NO	GK10857 [Drosophila willistoni]
Unigene520	NO	cytochrome c oxidase subunit 4 isoform 1, mitochondrial [Nasonia vitripennis]
Unigene5280	NO	PREDICTED: LOW QUALITY PROTEIN: NEDD8-activating enzyme E1 regulatory subunit [Apis mellifera]
Unigene533	NO	ATP synthase subunit b, mitochondrial [Acromyrmex echinatior]
Unigene5492	NO	hypothetical protein KGM_00095 [Danaus plexippus]
Unigene553	NO	PREDICTED: cytochrome b-c1 complex subunit 6, mitochondrial-like [Megachile rotundata]
Unigene558	NO	AGAP008724-PA [Anopheles gambiae str. PEST]
Unigene564	NO	PREDICTED: similar to mitochondrial ATP synthase coupling factor 6 [Tribolium castaneum]
Unigene579	NO	cytochrome c oxidase subunit VIb isoform 1 [Rhipicephalus sanguineus]
Unigene6060	NO	lethal neo18 protein [Danaus plexippus]
Unigene608	NO	hypothetical protein EAG_05916 [Camponotus floridanus]
Unigene614	NO	Cytochrome b-c1 complex subunit Rieske, mitochondrial [Camponotus floridanus]
Unigene624	NO	ATP synthase subunit gamma, mitochondrial [Nasonia vitripennis]
Unigene6363	NO	Mitogen-activated protein kinase 1 [Harpegnathos saltator]
Unigene647	NO	PREDICTED: cytochrome b-c1 complex subunit Rieske, mitochondrial-like [Cavia porcellus]
Unigene6600	NO	PREDICTED: presenilin homolog [Bombus impatiens]
Unigene663	NO	Cytochrome b-c1 complex subunit 7 [Camponotus floridanus]
Unigene6630	NO	cytochrome c oxidase [Danaus plexippus]
Unigene6640	NO	Nicastrin [Camponotus floridanus]
Unigene6642	NO	NADH dehydrogenase [Danaus plexippus]
Unigene6695	NO	unnamed protein product [Heliconius melpomene]
Unigene6842	NO	guanine nucleotide binding protein alpha q polypeptide [Glossina morsitans morsitans]
Unigene6884	NO	H+ transporting ATP synthase O subunit isoform 1 [Bombyx mori]
Unigene694	NO	ATP synthase subunit O, mitochondrial [Camponotus floridanus]
Unigene71	NO	hypothetical protein EAI_14262 [Harpegnathos saltator]
Unigene7100	NO	cytochrome c oxidase polypeptide IV [Bombyx mori]
Unigene7180	NO	39S ribosomal protein L46, mitochondrial [Acromyrmex echinatior]

	Unigene731	NO	hypothetical protein SINV_08923 [Solenopsis invicta]
	Unigene743	NO	GF11012 [Drosophila ananassae]
	Unigene7464	NO	Eukaryotic translation initiation factor 2-alpha kinase [Acromyrmex echinatior]
	Unigene7469	NO	NADH-ubiquinone oxidoreductase 75 kDa subunit, mitochondrial precursor [Nasonia vitripennis]
	Unigene7619	NO	hypothetical protein SINV_04265 [Solenopsis invicta]
	Unigene763	NO	hypothetical protein SINV_15147 [Solenopsis invicta]
	Unigene7714	NO	Mitogen-activated protein kinase 14B [Harpegnathos saltator]
	Unigene776	NO	AT15141p [Drosophila melanogaster]
	Unigene787	NO	NADH dehydrogenase [ubiquinone] 1 alpha subcomplex subunit 6 [Nasonia vitripennis]
	Unigene7938	NO	hypothetical protein KGM_04538 [Danaus plexippus]
	Unigene7980	NO	PREDICTED: dual specificity mitogen-activated protein kinase kinase 2-like [Apis florea]
	Unigene799	NO	hypothetical protein SINV_06969 [Solenopsis invicta]
	Unigene7994	NO	PREDICTED: hypothetical protein LOC100122162 [Nasonia vitripennis]
	Unigene8104	NO	NADH dehydrogenase [Danaus plexippus]
	Unigene8113	NO	Gamma-secretase subunit Aph-1 [Camponotus floridanus]
	Unigene8196	NO	cytochrome c oxidase subunit III [Cotesia vestalis]
	Unigene8222	NO	ubiquinol-cytochrome C reductase complex 14kD subunit [Danaus plexippus]
	Unigene8267	NO	Insulin-degrading enzyme [Camponotus floridanus]
	Unigene829	NO	ATP synthase subunit d, mitochondrial [Camponotus floridanus]
	Unigene8339	NO	PREDICTED: hypothetical protein LOC100743562 isoform 2 [Bombus impatiens]
	Unigene837	NO	hypothetical protein AND_29231 [Anopheles darlingi]
	Unigene8416	NO	H+ transporting ATP synthase O subunit isoform 1 [Bombyx mori]
	Unigene8887	NO	PREDICTED: gamma-secretase subunit Aph-1-like [Megachile rotundata]
	Unigene8922	NO	mitochondrial ATP synthase coupling factor [Antheraea yamamai]
	Unigene9008	NO	PREDICTED: guanine nucleotide-binding protein G(q) subunit alpha-like isoform 5 [Megachile rotundata]
	Unigene9081	NO	putative laminin A chain [Danaus plexippus]
	Unigene912	NO	cytochrome c1, heme protein, mitochondrial [Nasonia vitripennis]
	Unigene9141	NO	PREDICTED: hypothetical protein LOC100743562 isoform 1 [Bombus impatiens]
	Unigene9356	NO	pancreatic lipase 3 [Mamestra configurata]
	Unigene9415	NO	PREDICTED: mitogen-activated protein kinase 14B-like isoform 2 [Megachile rotundata]
	Unigene9462	NO	NADH dehydrogenase ubiquinone Fe-S 8 [Bombyx mori]
	Unigene9485	NO	H+ transporting ATP synthase O subunit isoform 1 [Danaus plexippus]
	Unigene9517	NO	PREDICTED: insulin-degrading enzyme-like isoform 2 [Nasonia vitripennis]
	Unigene952	NO	PREDICTED: acyl carrier protein, mitochondrial-like [Megachile rotundata]
	Unigene959	NO	NADH dehydrogenase [ubiquinone] 1 beta subcomplex subunit 5, mitochondrial [Nasonia vitripennis]
	Unigene9659	NO	sarco/endoplasmic reticulum calcium ATPase [Bombyx mori]
	Unigene991	NO	PREDICTED: NADH dehydrogenase [ubiquinone] 1 alpha subcomplex subunit 7-like [Megachile rotundata]
	Unigene9927	NO	Subunit VIb of cytochrome c oxidase [Danaus plexippus]
	Unigene9971	NO	hypothetical protein KGM_15724 [Danaus plexippus]
	CL124.Contig1	NO	succinate dehydrogenase cytochrome b560 subunit, mitochondrial [Nasonia vitripennis]
	Unigene12984	SP	Allatostatin C preprohormone [Nasonia vitripennis]
	Unigene16846	SP	NPF-like [Nasonia vitripennis]
	CL375.Contig2	NO	guanine nucleotide-binding protein subunit beta-like [Microplitis mediator]
	CL417.Contig1	NO	PREDICTED: dual specificity protein phosphatase 10-like [Bombus terrestris]
	CL417.Contig2	NO	PREDICTED: dual specificity protein phosphatase 10-like [Megachile rotundata]
	CL724.Contig1	NO	PREDICTED: dual specificity protein phosphatase Mpk3-like [Megachile rotundata]
	CL724.Contig2	NO	PREDICTED: dual specificity protein phosphatase Mpk3-like [Megachile rotundata]
	Unigene10095	NO	dopa decarboxylase [Papilio machaon]
	Unigene10121	NO	85 kDa calcium-independent phospholipase A2 [Camponotus floridanus]
	Unigene10184	NO	hypothetical protein DAPPUDRAFT_304184 [Daphnia pulex]
	Unigene10363	NO	GTP-binding protein alpha subunit, gna [Culex quinquefasciatus]
	Unigene10531	NO	phospholipase A2 precursor [Apis mellifera]
	Unigene11077	NO	PREDICTED: dual specificity protein phosphatase Mpk3-like [Megachile rotundata]
	Unigene11087	NO	PREDICTED: guanine nucleotide-binding protein G(i) subunit alpha-like [Megachile rotundata]
	Unigene11455	NO	PREDICTED: serine/threonine/tyrosine-interacting protein-like [Bombus impatiens]
	Unigene11598	NO	PREDICTED: group XIIA secretory phospholipase A2-like [Apis florea]
	Unigene11613	NO	PREDICTED: calcium-independent phospholipase A2-gamma-like [Bombus terrestris]
	Unigene11659	NO	GTP-binding protein (i) alpha subunit, gnai [Aedes aegypti]
	Unigene13476	NO	Aromatic-L-amino-acid decarboxylase [Acromyrmex echinatior]

**Neuronal secretion**	Unigene13635	NO	PREDICTED: LOW QUALITY PROTEIN: BTB/POZ domain-containing protein KCTD3-like [Nasonia vitripennis]
	Unigene1927	NO	PREDICTED: SH3 domain-binding glutamic acid-rich protein homolog [Megachile rotundata]
	Unigene2017	NO	hypothetical protein AaeL_AAEL006826 [Aedes aegypti] >
	Unigene2069	NO	PREDICTED: ras-like protein 1-like [Apis florea]
	Unigene2152	NO	Guanine nucleotide-binding protein subunit gamma-1 [Camponotus floridanus]
	Unigene2339	NO	Guanine nucleotide-binding protein subunit gamma-e [Harpegnathos saltator]
	Unigene2474	NO	dopa decarboxylase [Mythimna separata]
	Unigene2504	NO	hypothetical protein TRIADDRAFT_20496 [Trichoplax adhaerens]
	Unigene3861	NO	PREDICTED: group XIIA secretory phospholipase A2-like [Nasonia vitripennis]
	Unigene4101	NO	hypothetical protein SINV_05984 [Solenopsis invicta]
	Unigene4920	NO	PREDICTED: mitogen-activated protein kinase 1-like [Megachile rotundata]
	Unigene6195	NO	PREDICTED: dual specificity protein phosphatase Mpk3-like [Bombus impatiens]
	Unigene6363	NO	Mitogen-activated protein kinase 1 [Harpegnathos saltator]
	Unigene6842	NO	guanine nucleotide binding protein alpha q polypeptide [Glossina morsitans morsitans]
	Unigene7102	NO	Multiple C2 and transmembrane domain-containing protein 2 [Acromyrmex echinatior]
	Unigene7980	NO	PREDICTED: dual specificity mitogen-activated protein kinase kinase 2-like [Apis florea]
	Unigene8035	NO	PREDICTED: guanine nucleotide-binding protein G(s) subunit alpha-like [Megachile rotundata]
	Unigene8571	NO	Aromatic-L-amino-acid decarboxylase [Acromyrmex echinatior]
	Unigene9008	NO	PREDICTED: guanine nucleotide-binding protein G(q) subunit alpha-like isoform 5 [Megachile rotundata]
	Unigene9653	NO	PREDICTED: guanine nucleotide-binding protein G(s) subunit alpha-like [Megachile rotundata]
	Unigene10984	NO	Partner of bursicon [Camponotus floridanus]
	Unigene12277	NO	Partner of bursicon [Camponotus floridanus]
	CL375.Contig1	NO	guanine nucleotide-binding protein subunit beta-like [Microplitis mediator]
**Immune regulation**			
	**Unigene ID**	**ignal peptid**	**Homologs**
**Toll signaling pathway**	Unigene13453	SP	PREDICTED: protein toll-like [Megachile rotundata]
	Unigene2015	SP	venom serine protease [Bombus ignitus]
	Unigene280	SP	PREDICTED: heat shock 70 kDa protein cognate 3-like [Nasonia vitripennis]
	Unigene758	SP	Protein toll [Harpegnathos saltator]
	Unigene899	SP	Protein toll [Acromyrmex echinatior]
	Unigene932	SP	PREDICTED: ubiquitin-conjugating enzyme E2-17 kDa-like [Bombus impatiens]
	Unigene933	SP	Ubiquitin-conjugating enzyme E2-17 kDa [Camponotus floridanus]
	Unigene6211	NO	serine proteinase-like protein 1b [Manduca sexta]
	Unigene5363	NO	seminal fluid protein CSSFP025 [Chilo suppressalis]
	CL99.Contig1	NO	heat shock protein 70 [Microplitis mediator]
	Unigene701	NO	heat shock protein 70 [Agrotis ipsilon]
	CL99.Contig5	NO	Heat shock 70 kDa protein cognate 4 [Camponotus floridanus]
	CL99.Contig2	NO	heat shock protein 70 [Microplitis mediator]
	Unigene10792	NO	heat shock protein 70-3 [Bombyx mori]
	CL99.Contig3	NO	heat-shock protein 70 [Cotesia rubecula]
	CL99.Contig4	NO	heat-shock protein 70 [Cotesia rubecula]
	Unigene724	NO	PREDICTED: similar to heat shock protein 70 [Tribolium castaneum]
	Unigene10221	NO	Uncharacterized protein C19orf29 [Harpegnathos saltator]
	Unigene11697	NO	PREDICTED: uncharacterized protein C19orf29-like [Megachile rotundata]
	Unigene2463	NO	PREDICTED: proto-oncogene c-Rel [Bombus terrestris]
	Unigene1619	NO	PREDICTED: putative transcription factor p65 homolog [Megachile rotundata]
	Unigene3441	NO	Protein pellino [Acromyrmex echinatior]
	Unigene1019	NO	PREDICTED: stress-induced-phosphoprotein 1-like [Megachile rotundata]
	Unigene927	NO	PREDICTED: toll-interacting protein-like [Megachile rotundata]
	Unigene66	NO	Relish [Drepanotermes rubriceps]
	Unigene1702	NO	Ubiquitin-conjugating enzyme E2 N [Camponotus floridanus]
	Unigene4733	NO	Peptidoglycan-recognition protein-LF [Harpegnathos saltator]
	CL624.Contig2	NO	S-phase kinase-associated protein [Danaus plexippus]
	CL624.Contig1	NO	S-phase kinase-associated protein [Danaus plexippus]
	Unigene7819	NO	TATA-binding protein-associated factor 172 [Acromyrmex echinatior]
	Unigene3678	NO	PREDICTED: caspase-1-like [Nasonia vitripennis]
	Unigene13759	NO	PREDICTED: E3 ubiquitin-protein ligase CBL-B-like [Nasonia vitripennis]
	Unigene12089	NO	E3 ubiquitin-protein ligase CBL-B [Acromyrmex echinatior]
	Unigene742	NO	Serine protease snake [Acromyrmex echinatior]

	Unigene8313	NO	hypothetical protein SINV_00720 [Solenopsis invicta]
	Unigene8688	NO	Sortilin-related receptor [Harpegnathos saltator]
	Unigene9032	NO	TNF receptor-associated factor 4 [Harpegnathos saltator]
**JNK signaling pathway**	CL178.Contig3	NO	heat shock protein 90 [Microplitis mediator]
	CL178.Contig2	NO	Heat shock protein HSP 90-alpha [Harpegnathos saltator]
	Unigene1169	NO	heat shock protein 90 [Microplitis mediator]
	Unigene858	NO	heat shock protein 90 [Microplitis mediator]
	Unigene1913	NO	Transcription factor kayak [Acromyrmex echinatior]
	Unigene905	NO	PREDICTED: transcription factor AP-1-like [Megachile rotundata]
	CL417.Contig2	NO	PREDICTED: dual specificity protein phosphatase 10-like [Megachile rotundata]
	CL417.Contig1	NO	PREDICTED: dual specificity protein phosphatase 10-like [Bombus terrestris]
	Unigene9217	NO	PREDICTED: stress-activated protein kinase JNK-like [Megachile rotundata]
	Unigene11062	NO	Stress-activated protein kinase JNK [Acromyrmex echinatior]
	Unigene9998	NO	PREDICTED: stress-activated protein kinase JNK-like [Megachile rotundata]
	Unigene6295	NO	PREDICTED: dual specificity mitogen-activated protein kinase kinase hemipterous-like isoform 2 [Megachile rotundata]
	Unigene2418	NO	PREDICTED: RAC serine/threonine-protein kinase-like [Bombus impatiens]
	Unigene9415	NO	PREDICTED: mitogen-activated protein kinase 14B-like isoform 2 [Megachile rotundata]
	Unigene8421	NO	PREDICTED: KRR1 small subunit processome component homolog [Nasonia vitripennis]
	Unigene8094	NO	PREDICTED: serine/threonine-protein kinase hippo-like isoform 2 [Nasonia vitripennis]
	Unigene12110	NO	PREDICTED: serine/threonine-protein kinase 3-like [Megachile rotundata]
	CL88.Contig2	NO	Serine/threonine-protein kinase 3 [Harpegnathos saltator]
	CL88.Contig1	NO	Serine/threonine-protein kinase 3 [Harpegnathos saltator]
	Unigene5477	NO	PREDICTED: serine/threonine-protein kinase 25-like [Nasonia vitripennis]
**JAK-STAT signaling pathway**	CL668.Contig1	SP	PREDICTED: endoplasmin-like [Megachile rotundata]
	Unigene11830	SP	PREDICTED: CD109 antigen-like [Bombus impatiens]
	Unigene2241	SP	hypothetical protein SINV_07362 [Solenopsis invicta]
	Unigene280	SP	PREDICTED: heat shock 70 kDa protein cognate 3-like [Nasonia vitripennis]
	Unigene7815	SP	hypothetical protein SINV_07362 [Solenopsis invicta]
	Unigene7824	SP	heat shock protein 70-3 [Bombyx mori]
	Unigene1887	NO	60 kDa heat shock protein, mitochondrial [Harpegnathos saltator]
	Unigene3777	NO	heat shock protein 60 [Pteromalus puparum]
	CL99.Contig5	NO	Heat shock 70 kDa protein cognate 4 [Camponotus floridanus]
	CL99.Contig3	NO	heat-shock protein 70 [Cotesia rubecula]
	CL99.Contig2	NO	heat shock protein 70 [Microplitis mediator]
	Unigene701	NO	heat shock protein 70 [Agrotis ipsilon]
	CL99.Contig1	NO	heat shock protein 70 [Microplitis mediator]
	Unigene1630	NO	small heat shock protein [Pteromalus puparum]
	Unigene10792	NO	heat shock protein 70-3 [Bombyx mori]
	Unigene10376	NO	hypothetical protein KGM_13870 [Danaus plexippus]
	CL271.Contig1	NO	ADP-ribosylation factor 1 [Harpegnathos saltator]
	Unigene4378	NO	hypothetical protein SINV_14695 [Solenopsis invicta]
	CL271.Contig2	NO	ADP-ribosylation factor [Bombyx mori]
	Unigene9217	NO	PREDICTED: stress-activated protein kinase JNK-like [Megachile rotundata]
	Unigene9998	NO	PREDICTED: stress-activated protein kinase JNK-like [Megachile rotundata]
	Unigene209	NO	PREDICTED: polyubiquitin-A-like, partial [Apis florea]
	Unigene208	NO	PREDICTED: polyubiquitin-A-like, partial [Apis florea]
	Unigene6788	NO	ubiquitin C, partial [Mylabris cichorii]
	CL10.Contig2	NO	ubiquitin precursor [Trypanosoma cruzi]
	Unigene1466	NO	PREDICTED: heat shock 70 kDa protein cognate 5-like [Nasonia vitripennis]
	Unigene724	NO	PREDICTED: similar to heat shock protein 70 [Tribolium castaneum]
	Unigene2418	NO	PREDICTED: RAC serine/threonine-protein kinase-like [Bombus impatiens]
	Unigene2210	NO	PREDICTED: signal transducer and activator of transcription 5B-like [Megachile rotundata]
	Unigene1896	NO	PREDICTED: heat shock 70 kDa protein 4L-like [Bombus terrestris]
	Unigene1070	NO	Heat shock protein 105 kDa [Harpegnathos saltator]
	Unigene2949	NO	PREDICTED: E3 SUMO-protein ligase PIAS1-like [Megachile rotundata]
	Unigene2562	NO	E3 SUMO-protein ligase PIAS3 [Acromyrmex echinatior]
	Unigene3251	NO	PREDICTED: LOW QUALITY PROTEIN: E3 SUMO-protein ligase PIAS3-like [Apis florea]
	Unigene5367	NO	cytokine-inducible SH2-like protein [Glyptapanteles indiensis]
	Unigene10073	NO	Suppressor of cytokine signaling 5 [Acromyrmex echinatior]

	Unigene8421	NO	PREDICTED: KRR1 small subunit processome component homolog [Nasonia vitripennis]
	Unigene8094	NO	PREDICTED: serine/threonine-protein kinase hippo-like isoform 2 [Nasonia vitripennis]
	Unigene12110	NO	PREDICTED: serine/threonine-protein kinase 3-like [Megachile rotundata]
	CL88.Contig2	NO	Serine/threonine-protein kinase 3 [Harpegnathos saltator]
	CL88.Contig1	NO	Serine/threonine-protein kinase 3 [Harpegnathos saltator]
	Unigene5477	NO	PREDICTED: serine/threonine-protein kinase 25-like [Nasonia vitripennis]
	Unigene11904	NO	hypothetical protein KGM_05477 [Danaus plexippus]
	Unigene526	NO	heat shock cognate protein [Bombyx mori]
	Unigene6783	NO	heat shock protein [Helicoverpa armigera]
	Unigene10829	NO	heat shock protein 60 [Chilo suppressalis]
	Unigene8994	NO	Heat shock protein 75 kDa, mitochondrial [Acromyrmex echinatior]
	CL178.Contig3	NO	heat shock protein 90 [Microplitis mediator]
	Unigene858	NO	heat shock protein 90 [Microplitis mediator]
	CL178.Contig2	NO	Heat shock protein HSP 90-alpha [Harpegnathos saltator]
	Unigene1169	NO	heat shock protein 90 [Microplitis mediator]
**IMD signaling pathway**	Unigene13453	SP	PREDICTED: protein toll-like [Megachile rotundata]
	Unigene2015	SP	venom serine protease [Bombus ignitus]
	Unigene280	SP	PREDICTED: heat shock 70 kDa protein cognate 3-like [Nasonia vitripennis]
	Unigene758	SP	Protein toll [Harpegnathos saltator]
	Unigene899	SP	Protein toll [Acromyrmex echinatior]
	Unigene932	SP	PREDICTED: ubiquitin-conjugating enzyme E2-17 kDa-like [Bombus impatiens]
	Unigene933	SP	Ubiquitin-conjugating enzyme E2-17 kDa [Camponotus floridanus]
	Unigene6211	NO	serine proteinase-like protein 1b [Manduca sexta]
	Unigene5363	NO	seminal fluid protein CSSFP025 [Chilo suppressalis]
	CL99.Contig1	NO	heat shock protein 70 [Microplitis mediator]
	Unigene701	NO	heat shock protein 70 [Agrotis ipsilon]
	CL99.Contig5	NO	Heat shock 70 kDa protein cognate 4 [Camponotus floridanus]
	CL99.Contig2	NO	heat shock protein 70 [Microplitis mediator]
	Unigene10792	NO	heat shock protein 70-3 [Bombyx mori]
	CL99.Contig3	NO	heat-shock protein 70 [Cotesia rubecula]
	CL99.Contig4	NO	heat-shock protein 70 [Cotesia rubecula]
	Unigene724	NO	PREDICTED: similar to heat shock protein 70 [Tribolium castaneum]
	Unigene10221	NO	Uncharacterized protein C19orf29 [Harpegnathos saltator]
	Unigene11697	NO	PREDICTED: uncharacterized protein C19orf29-like [Megachile rotundata]
	Unigene2463	NO	PREDICTED: proto-oncogene c-Rel [Bombus terrestris]
	Unigene1619	NO	PREDICTED: putative transcription factor p65 homolog [Megachile rotundata]
	Unigene3441	NO	Protein pellino [Acromyrmex echinatior]
	Unigene1019	NO	PREDICTED: stress-induced-phosphoprotein 1-like [Megachile rotundata]
	Unigene927	NO	PREDICTED: toll-interacting protein-like [Megachile rotundata]
	Unigene66	NO	Relish [Drepanotermes rubriceps]
	Unigene1702	NO	Ubiquitin-conjugating enzyme E2 N [Camponotus floridanus]
	Unigene4733	NO	Peptidoglycan-recognition protein-LF [Harpegnathos saltator]
	CL624.Contig2	NO	S-phase kinase-associated protein [Danaus plexippus]
	CL624.Contig1	NO	S-phase kinase-associated protein [Danaus plexippus]
	Unigene7819	NO	TATA-binding protein-associated factor 172 [Acromyrmex echinatior]
	Unigene3678	NO	PREDICTED: caspase-1-like [Nasonia vitripennis]
	Unigene13759	NO	PREDICTED: E3 ubiquitin-protein ligase CBL-B-like [Nasonia vitripennis]
	Unigene12089	NO	E3 ubiquitin-protein ligase CBL-B [Acromyrmex echinatior]
	Unigene742	NO	Serine protease snake [Acromyrmex echinatior]
	Unigene8313	NO	hypothetical protein SINV_00720 [Solenopsis invicta]
	Unigene8688	NO	Sortilin-related receptor [Harpegnathos saltator]
	Unigene9032	NO	TNF receptor-associated factor 4 [Harpegnathos saltator]
	CL154.Contig1	SP	venom protein Vn4.6 [Cotesia rubecula]
	CL488.Contig1	SP	phenoloxidase inhibitor-like protein [Simulium nigrimanum]
	Unigene1055	SP	C-type lectin [Acyrthosiphon pisum]
	Unigene1401	SP	C-type lectin [Acyrthosiphon pisum]
	Unigene2015	SP	Proclotting enzyme [Acromyrmex echinatior]
	Unigene239	SP	venom serine protease [Bombus ignitus]
	Unigene739	SP	venom protein Vn50 [Cotesia rubecula]
	Unigene842	SP	PREDICTED: peroxiredoxin-4-like [Nasonia vitripennis]
	Unigene7007	NO	thiol peroxiredoxin [Danaus plexippus]
	Unigene3602	NO	thiol peroxiredoxin [Bombyx mori]
	CL178.Contig3	NO	heat shock protein 90 [Microplitis mediator]
	CL178.Contig2	NO	Heat shock protein HSP 90-alpha [Harpegnathos saltator]

**Humoral response**	Unigene1169	NO	heat shock protein 90 [Microplitis mediator]
	Unigene2136	NO	PREDICTED: dihydropteridine reductase-like [Nasonia vitripennis]
	CL510.Contig1	NO	annexin IX [Helicoverpa armigera]
	Unigene2509	NO	putative annexin IX-C [Manduca sexta]
	Unigene544	NO	PREDICTED: annexin-B9-like [Megachile rotundata]
	Unigene2233	NO	PREDICTED: peroxiredoxin-6-like [Nasonia vitripennis]
	Unigene7315	NO	PREDICTED: uncharacterized protein LOC100875503 [Megachile rotundata]
	Unigene572	NO	Cu,Zn superoxidase dismutase [Bombus ignitus]
	Unigene8571	NO	Aromatic-L-amino-acid decarboxylase [Acromyrmex echinatior]
	Unigene13104	NO	GTP cyclohydrolase 1 [Harpegnathos saltator]
	Unigene4386	NO	PREDICTED: hypothetical protein LOC100648816 [Bombus terrestris]
	Unigene2566	NO	PREDICTED: superoxide dismutase [Mn] 1, mitochondrial-like [Nasonia vitripennis]
	Unigene687	NO	PREDICTED: peroxiredoxin 1-like [Megachile rotundata]
	Unigene8475	NO	Negative elongation factor A [Camponotus floridanus]
	Unigene7594	NO	PREDICTED: negative elongation factor A-like [Apis florea]
	Unigene3723	NO	Vascular endothelial growth factor receptor 1 [Camponotus floridanus]
	Unigene11836	NO	PREDICTED: vascular endothelial growth factor receptor 1 [Apis mellifera]
	Unigene2315	NO	Vascular endothelial growth factor receptor 1 [Camponotus floridanus]
**AMPs**	Unigene11974	SP	Crustin 4 [Panulirus japonicus]
	Unigene148	SP	Hymenoptaecin [Apis cerana cerana]
	Unigene164	SP	PREDICTED: hymenoptaecin-like [Megachile rotundata]
	Unigene165	SP	Hymenoptaecin [Acromyrmex echinatior]
	Unigene971	SP	Defensin [Apis cerana japonica]
	Unigene284	SP	Defensin 2 precursor [Cotesia vestalis]
	Unigene80	SP	Defensin 2 precursor [Cotesia vestalis]
**MAPK signaling pathway**	CL61.Contig6	SP	hypothetical protein GIP_L3_0010 [Glyptapanteles indiensis]
	CL17.Contig1	NO	PREDICTED: protein phosphatase 1B-like [Apis florea]
	CL17.Contig2	NO	PREDICTED: protein phosphatase 1B-like [Apis florea]
	CL17.Contig3	NO	PREDICTED: protein phosphatase 1B-like [Megachile rotundata]
	CL17.Contig4	NO	PREDICTED: protein phosphatase 1B-like [Megachile rotundata]
	CL252.Contig1	NO	MAP kinase-interacting serine/threonine-protein kinase 1 [Camponotus floridanus]
	CL252.Contig2	NO	MAP kinase-interacting serine/threonine-protein kinase 1 [Camponotus floridanus]
	CL363.Contig1	NO	PREDICTED: serine/threonine-protein phosphatase 2B catalytic subunit 2-like [Apis florea]
	CL363.Contig2	NO	PREDICTED: serine/threonine-protein phosphatase 2B catalytic subunit 2-like [Apis florea]
	CL417.Contig1	NO	PREDICTED: dual specificity protein phosphatase 10-like [Bombus terrestris]
	CL417.Contig2	NO	PREDICTED: dual specificity protein phosphatase 10-like [Megachile rotundata]
	CL483.Contig1	NO	PREDICTED: hypothetical protein LOC100743933 [Bombus impatiens]
	CL483.Contig2	NO	Microtubule-associated protein 2 [Harpegnathos saltator]
	CL510.Contig1	NO	annexin IX [Helicoverpa armigera]
	CL513.Contig1	NO	hypothetical protein PANDA_004153 [Ailuropoda melanoleuca]
	CL717.Contig1	NO	PREDICTED: beta-arrestin-1-like isoform 2 [Bombus impatiens]
	CL717.Contig2	NO	PREDICTED: beta-arrestin-1-like isoform 2 [Bombus impatiens]
	CL724.Contig1	NO	PREDICTED: dual specificity protein phosphatase Mpk3-like [Megachile rotundata]
	CL724.Contig2	NO	PREDICTED: dual specificity protein phosphatase Mpk3-like [Megachile rotundata]
	CL88.Contig1	NO	Serine/threonine-protein kinase 3 [Harpegnathos saltator]
	CL88.Contig2	NO	Serine/threonine-protein kinase 3 [Harpegnathos saltator]
	CL99.Contig1	NO	heat shock protein 70 [Microplitis mediator]
	CL99.Contig2	NO	heat shock protein 70 [Microplitis mediator]
	CL99.Contig3	NO	heat-shock protein 70 [Cotesia rubecula]
	CL99.Contig4	NO	heat-shock protein 70 [Cotesia rubecula]
	CL99.Contig5	NO	Heat shock 70 kDa protein cognate 4 [Camponotus floridanus]
	Unigene10121	NO	85 kDa calcium-independent phospholipase A2 [Camponotus floridanus]
	Unigene10138	NO	PREDICTED: uncharacterized protein LOC100881293 [Megachile rotundata]
	Unigene10158	NO	PREDICTED: serine/threonine-protein kinase TAO1-like [Bombus impatiens]
	Unigene10169	NO	PREDICTED: uncharacterized protein LOC100866420 [Apis florea]
	Unigene10184	NO	hypothetical protein DAPPUDRAFT_304184 [Daphnia pulex]
**Cell cycle regulation**	Unigene2418	NO	PREDICTED: RAC serine/threonine-protein kinase-like [Bombus impatiens]
	Unigene7027	NO	AGAP005160-PA [Anopheles gambiae str. PEST]
	Unigene1968	NO	Ras-related protein Rac1 [Camponotus floridanus]
	Unigene13262	NO	PREDICTED: uncharacterized protein LOC100876362 [Megachile rotundata]
	Unigene8421	NO	PREDICTED: KRR1 small subunit processome component homolog [Nasonia vitripennis]
	Unigene8094	NO	PREDICTED: serine/threonine-protein kinase hippo-like isoform 2 [Nasonia vitripennis]

	Unigene12110	NO	PREDICTED: serine/threonine-protein kinase 3-like [Megachile rotundata]
	CL88.Contig2	NO	Serine/threonine-protein kinase 3 [Harpegnathos saltator]
	CL88.Contig1	NO	Serine/threonine-protein kinase 3 [Harpegnathos saltator]
	Unigene5477	NO	PREDICTED: serine/threonine-protein kinase 25-like [Nasonia vitripennis]

Table S4   Verified genes

Unigene ID	RT-PCR verified transcripts or homologs	GenBank accession number of full length cDNA
Unigene19276	*Trehalase* , *Tre*	/
Unigene20417	*Fatty acid binding protein* , *FABP*	/
Unigene5800	*Metrix metalloproteinase 14* , *MMP-14*	JX399873
Unigene239	*Hexamerin* , *HexL*	/
Unigene3237	*Juvenile hormone esterase* , *JHE*	/
Unigene262	*TSP-13*	JX399877
Unigene4760	*TSVP-GGCT*	JX399879
Unigene336	*Venom protein 2* , *VEP-2*	/
Unigene449	*Venom protein 8* , *VEP-8*	/
Unigene494	*Chitinase 3* , *Chi-3*	/
CL154.Contig1	*TSVP-8*	JX399876
Unigene739	*TSVP-42*	JX399875
Unigene1055	*C-type lectin* , *CTL*	JX399878
Unigene971	*CvT-defensin 1*	KC306694
Unigene80	*CvT-defensin 2*	KC306695
Unigene12543	*TSVP-allergen*	/
Unigene2015	*TSVP-SEP*	JX399874
Unigene11254	*Supervillin* , *SUP*	/
Unigene12637	*Moesin/ezrin/radixin homolog 1* , *MERH-1*	/
Unigene7188	*Plastin 3* , *Pla-3*	/
Unigene15088	*CvT-defensin 3*	JX282323
Unigene31308	*Peptidoglycan-recognition protein 1, PGRP 1*	KT322130
Unigene5994	*peptidoglycan-recognition protein 2, PGRP 2*	KT309131

Table S5 Summary statistics for DGE sequencing of *Cotesia vestalis* teratocytes

Summary	Sample*	DGE		
		Libary 1	Library 2	SE**
Total reads	Ter-1d	5895449	5823820	50649.35
	Ter-3d	6272972	6217489	39232.41
	Ter-5d	6214769	6122485	65254.64
Qualit-filtered reads	Ter-1d	5823740	5784567	27699.49
	Ter-3d	6217468	6147511	49467.07
	Ter-5d	6124139	6072356	36616.11
Mapped reads	Ter-1d	4721581	5432489	502687.87
	Ter-3d	3638272	5848840	1563107.62
	Ter-5d	6232402	5600784	446621.37
Mapped genes	Ter-1d	9788	9718	49.50
	Ter-3d	8947	8866	57.28
	Ter-5d	9186	9102	59.40

*Teratocytes were collected from hosts at 1, 3 and 5 days (d) post-parasitism.

**Standard error for the means from libraries 1 and 2.

Table S6 Primers used in gene verification (RT-PCR)

Gene	Forward（5’－ 3’）	Reward (5'- 3')
Tre	AGTGATGGTAATAGCAACGGCAAGT	CGAATGTCTTGGAGTCGGGAAATAT
Fatty acid binding protein, FABP	TTGGAACTGAGTGGGAAGAGGTGAT	GTGTCCTCCTCAATGGTAGTAGTAT
Metrix metalloproteinase 14, MMP-14	ACTAAGACACAACAAGCTTCACCTG	TTGGTCCCGCGATAACTGTTA
Hexamerin, HexL	ATACCAATGGACTTCCCTTCCCTCA	GGGGTGTAAATGTTTACTGCTCGGG
Juvenile hormone esterase , JHE	GGGATCCATGGCGATCAATTTAATGCC	CCTCGAGCACGAACGAGCCCTGG
TSP-13	TGACTGTTGTAATAAATTCGATGGGT	ATTTAGCCCACGGTTTCAGGT
TSVP-GGCT	TGACATTGAGCACTTAGATCACCAG	GAAGTTCACGATCTAATGCTCCCTT
Venom protein 2, VEP-2	GGTTCCAGGCTTCGGACTTTCGTAA	GAACAACCCGGCACCTTTAAGCG
Venom protein 8, VEP-8	GTGGTTTGAAGACTCCTATGGACAT	CCAACGAGTCTCTAGCAAATCTCAT
Chitinase 3 , Chi-3	GTTTTCAACGCACTCCTAGGCGG	CAATTGCATGAATTCCTTGTCCGTC
TSVP-8	TTAATCGTTCTGTATGGGCTCGTT	TTTAGCTTTAACACGCTTTTACAACG
TSVP-42	GTGAACCACTTCCACATCAAAATCG	TAAGCGACGTTTGCATAGATACCTG
C-type lectin , CTL	TTGGCACTCCTGAAAGACAGAAT	CAGCTTCATTATTATCAGCTCCTGT
CvT-defensin 1, CvT-Def 1	CGCTTGACCTGCGATTTGTTCTCCT	ATCCGGTGACCAAAGTGAACATAGAGT
CvT-defensin 2, CvT-Def 2	ATTGTTCACACCAGCAAATGTTGTTTGC	GAAATATTTGAGACGTGTGACGTTGAT
TSVP-allergen	AGGCGCTCAAGAATGGGCTAAT	GAACTCCTCGGGACAGTTATGCT
TSVP-SEP	AACACGACCAATGTCCTCTAACTCT	ACGCATTCTTTGATCGGATAGTCAT
Supervillin, SUP	ATCTGGAGACGCTCGGCGGTAT	TAGAGCTGGAAGATAACGAGCCCAT
Moesin/ezrin/radixin homolog 1 , MERH-1	ACGGGCGGTAACAAGAGAGGAGAT	GCGTCGTTGTAGAACTTTCCAGCA
Plastin 3 , Pla-3	AATGGTATCAGCAAAGTCCGAGC	ACACCAATTTCTCTAGCAATTGGAT
CvT-defensin 3, CvT-Def 3	ATGTGTCCTAGGATTGTAACGCG	TTACCCACGTGAATAATCTCCAAT
CvT-PGRP 1	ATGAGACGGAAGCTAATGCATG	TTATGAATGGGAATAATCGAATTTT
CvT-PGRP 2	ATGGCCAAATTCCTGGTTTTTTT	TTAATGACGGCAATGACAGACTCCT
β-tubulin	ATGTCTGCCACCTTTATCGGGAAC	GCCTCTGTGAACTCCATCTCGTCC
18S rRNA	CGCCTTTCAAGATACCAAAATACGCC	TAGCTCTTTCTTGATTCGGTGGGTG

Table S7 Primers used in RACE

Gene	Primers for 3'RACE (5'- 3')	Primers for 5'RACE (5'- 3')
MMP-14	AGGTGGAACTCTTGCTCACGCTTAC	ATGAGCGGCTACTTGGAACAAATTG
	CGGTGTAGCCAGTGGGTATCCTAG	GATTCTCTTGCCGACGTACCTCC
TSP-13	TGACTGTTGTAATAAATTCGATGGGT	TTCTCGAACATTCTATCGGATGTCT
	AGCAGTTGTAGACATCCGATAGAAT	ATTTAGCCCACGGTTTCAGGT
TSVP-GGCT	TGACATTGAGCACTTAGATCACCAG	GGATTAGGCACCTTTATTTGCTGAT
	ATCAGCAAATAAAGGTGCCTAATCC	GAAGTTCACGATCTAATGCTCCCTT
TSVP-8	TTAATCGTTCTGTATGGGCTCGTT	TCGCAAAATTGTGAAACTTCTTGTT
	GTCAAAAGGCTGAACCATAAACTCAT	TTTAGCTTTAACACGCTTTTACAACG
TSVP-42	GTGAACCACTTCCACATCAAAATCG	TAATACCGCAAAGTCATTGTGTAAAT
	AGTCCATTAGTCTGCCCTCTTCGT	TAAGCGACGTTTGCATAGATACCTG
TSVP-SEP	GCTGCCCGACAAGTGAAAGTAAT	GCCATAGGATGTGTTCCGTAGTG
	AGACTTGTATGTTGTCCGTCTTGGT	TCATGGCAATATCATCGGTATAATG
TSVP-allergen	AGGCGCTCAAGAATGGGCTAAT	GCATGGCTTCGAGTTATACCAATAT
	GGCAAACACCTATCTAGTCGGCT	GAACTCCTCGGGACAGTTATGCT
CvT-def 1	CGCTTGACCTGCGATTTGTTCTCCT	CCACCTGCCTCTGCAATGACAAACT
	ACGCGGCGGCTCTTGCTCAAAT	ATCCGGTGACCAAAGTGAACATAGAGT
CvT-def 2	TGACGTGTCAATCGGAAGCTGCGTT	ATTGTTCACACCAGCAAATGTTGTTTGC
	GCAAACAACATTTGCTGGTGTGAAC	GAAATATTTGAGACGTGTGACGTTGAT
CvT-def 3	TCGTTTGCCACGGGCTACC	GTTTTACGATGGCCAAATTCCT
	CGGAGTAGGTGATTCTGCTTGTGC	TACAACGATCTGATTAATGACGGC
CvT-PGRP 1	TATTGGAATAGGCTTGATCGGGACT	ATGCCGAAAGTCGTGGGAGGT
	GAAATGGTAAAATAAACGAAAGTGCTCA	AAGGAGGGCACTACTGTGGAGGA
CvT-PGRP 2	TCAAGAATGGAAGGCAAGAAACG	TGAATTTTCTTGGCGGGAATACG
	GATACAATTTTGTCATCGGGGAAGA	AGAGCACTCTTTTATGTCGTAACA

Table S8 Primers used for cloning of putatively full-length ORFs

Gene	Forward（5’－ 3’）	Reward (5'- 3')
MMP-14	ATGAAGAATACCTGGAGAACAGAG	CTACGTAATTATACAGGTCTTAGTGAT
TSP-13	ATGAAATTAAACGTTTTGATTTTTG	TCATTCGTCGTGCCATTTAG
TSVP-GGCT	ATGAACAAGAAAGTGTTGTATTTTG	ACAACATAGCACTAGGGTTGTTG
TSVP-8	ATGTCAAAAATAATTTTGTCTATTT	TTATGGTTCAGCCTTTTGACAT
TSVP-42	ATGACATTCAGCACCAAAGGC	TAGCCAGTAAATCCTCCAAATGA
TSVP-SEP	ATGAACGTGCACCCCAGAT	TTAATCTAGATTAGATGTTATAAAATCG
TSVP-allergen	ATGGTGGTCAAATTTTTTTTGGT	TCAAATTTCTTCGTCATCAATCG
CvT-def 1	ATGAAGTTGTTAATTGAATTTATCT	TTATTTACCAAACCACCTGCCTCTG
CvT-def 2	ATGGCAAAAAATTACAGTTCAATGT	TTATTGTTCACACCAGCAAATGTT
CvT-PGRP 1	TTTACAATACAGTTTTTCCCGCT	AATTACCCACGTGAATAATCTCCAA
CvT-PGRP 2	GAATTCAGAAAATGAGACGGAAG	TATTTATGAATGGGAATAATCGAAT

Table S9 Primers used in qPCR

Gene	Forward (5'- 3')	Reward (5'- 3')
Trehalase , Tre	AGTGATGGTAATAGCAACGGCAAGT	CGAATGTCTTGGAGTCGGGAAATAT
Fatty acid binding protein, FABP	TTGGAACTGAGTGGGAAGAGGTGAT	TCTCGCTTCGTTCATGCTGATACTT
Metrix metalloproteinase 14 , MMP-14	AGGTGGAACTCTTGCTCACGCTTAC	ATGAGCGGCTACTTGGAACAAATTG
Hexamerin, HexL	CTGAAGAAGCAGAAACCCGCAT	GGGGTGTAAATGTTTACTGCTCGGG
Juvenile hormone esterase , JHE	TGTTGAGCAGCCAGTTGAATCCAT	GACAATACCGCCAATGACAGGAACT
TSP-13	TGACTGTTGTAATAAATTCGATGGGT	TTCTCGAACATTCTATCGGATGTCT
TSVP-GGCT	TGACATTGAGCACTTAGATCACCAG	GGATTAGGCACCTTTATTTGCTGAT
Venom protein 2, VEP-2	GGTTCCAGGCTTCGGACTTTCGTAA	GAACAACCCGGCACCTTTAAGCG
Venom protein 8, VEP-8	TACGATGATACCCATGGACAATGAA	TCGCGATACCTAAAGGCTGGGTT
Chitinase 3 , Chi-3	GTTTTCAACGCACTCCTAGGCGG	CAATTGCATGAATTCCTTGTCCGTC
TSVP-8	TTAATCGTTCTGTATGGGCTCGTT	TCGCAAAATTGTGAAACTTCTTGTT
TSVP-42	AGTCCATTAGTCTGCCCTCTTCGT	TAAGCGACGTTTGCATAGATACCTG
C-type lectin , CTL	AGACGCATGAGTATTGGGAAGAGACT	CCTGGGAGGTAACGATAGCAAGA
CvT-defensin 1, CvT-Def 1	CGCTTGACCTGCGATTTGTTCTCCT	CCACCTGCCTCTGCAATGACAAACT
CvT-defensin 2, CvT-Def 2	TGACGTGTCAATCGGAAGCTGCGTT	ATTGTTCACACCAGCAAATGTTGTTTGC
TSVP-allergen	AGGCGCTCAAGAATGGGCTAAT	GCATGGCTTCGAGTTATACCAATAT
TSVP-SEP	CATTGCGACTGCGACCAAAGAT	ACGCATTCTTTGATCGGATAGTCAT
Supervillin , SUP	ATCTGGAGACGCTCGGCGGTAT	GCCCAGGTTGGTATTAATCCAGTCG
Moesin/ezrin/radixin homolog 1 , MERH-1	ACGGGCGGTAACAAGAGAGGAGAT	GCGTCGTTGTAGAACTTTCCAGCA
Plastin 3 , Pla-3	GAAAACTGCAACTACGCCGTGGA	CTTCTCATCAGCTGCCAAATCAAGG
CvT-defensin 3, CvT-Def 3	GTTTTACGATGGCCAAATTCCT	TACAACGATCTGATTAATGACGGC
CvT-PGRP 1	AGAAGGCAACAAATGGGACGATA	CTCAAAGTTTCCGAGACCTGACG
CvT-PGRP 2	CAAGAATGGAAGGCAAGAAACG	TTGAAATCCCGATGCTTTGAGTA

Table 10 Primers used in recombinant plasmid construction

Genes	Forward (5'- 3')	Reward (5'- 3')
TSVP-42	GACTGAATTCATGACATTCAGCACCAAAG	CTGAAAGCTTTTAGCCAGTAAATCCTCCAAAT
TSVP-8	CCGCTCGAGCGGATGTCAAAAATAATTTTGTC	CGCGGATCCGCGTTATGGTTCAGCCTTTTGAC

Table S11 Synthesized CvT-def peptides

Genes	amino acid sequences (5'- 3')
CvT-def 1	RRLTCDLFSFQSQWVSPNHSACAAKCIAKGKRGGSCSNGVCHCRGRWFGK
CvT-def 3	ATCDLLSGFGVGDSACAVHCLAKKFKGGWCEKGVCHCRH

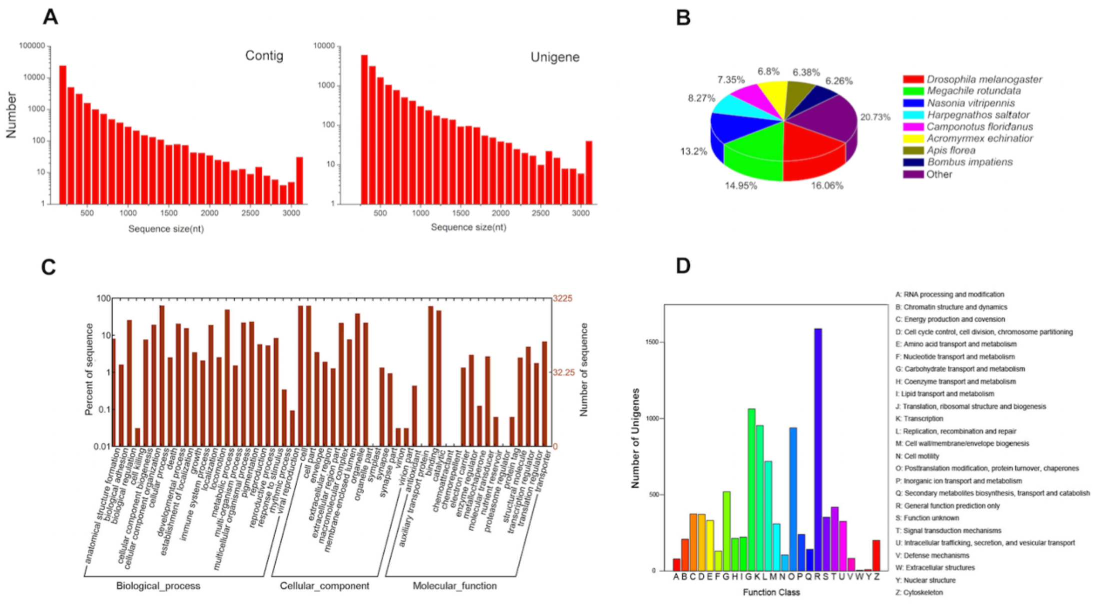

Figure S1. Summary of *Cotesia vestalis* teratocytes transcriptome data. (A) Length distribution of contigs and unigenes. (B) Species distribution of homology search of Illumina sequences against the Nr database. Species distribution is shown as a percentage of the total homologous sequences with an E-value of at least 1.0E-5. We used the first hit of each sequence for analysis. (C) Histogram presentation of Gene Ontology classification. The results are summarized in three main categories: biological process, cellular component and molecular function. The right y-axis indicates the number of genes in a category. The left y-axis indicates the percentage of a specific category of genes in that main category. (D) Histogram presentation of clusters of orthologous groups (COG) classification. Among 8,072 sequences annotated in Nr database, 4,395 sequences could be classified into 25 COG categories.

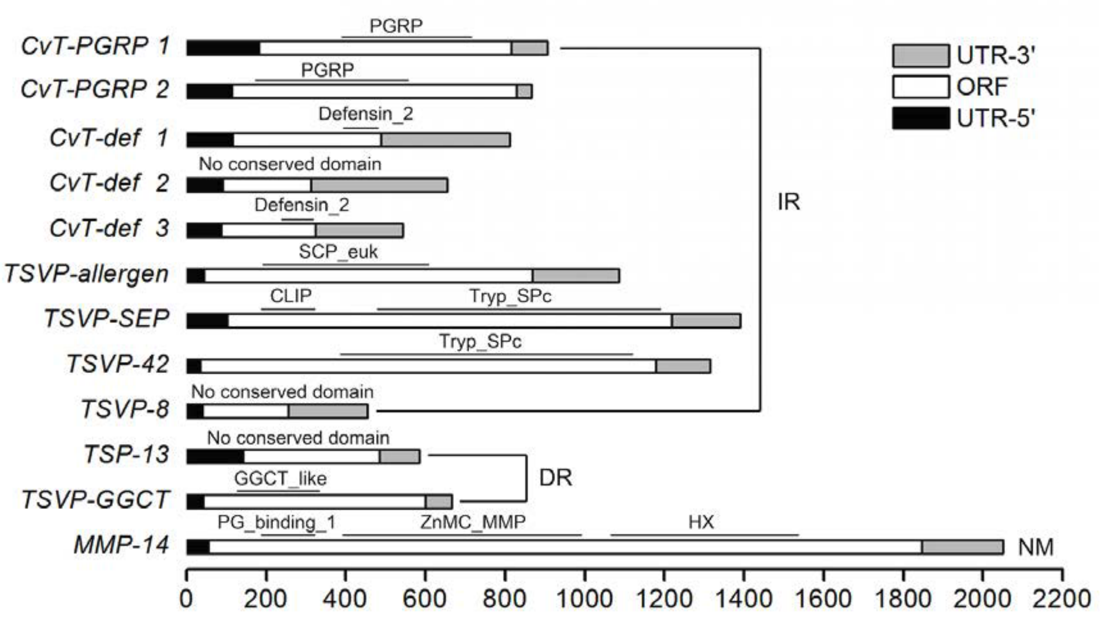

Figure S2. Structure of 12 *Cotesia vestalis* teratocytes for which full-length cDNAs were cloned. UTR-3': non-coding region of 3' end; ORF: open reading frame; NCR-5': non-coding region of 5' end. IR: immune regulation, DR: developmental regulation, NM: nutrient metabolism. Solid lines top on the ORF indicate the locations of corresponding domains.

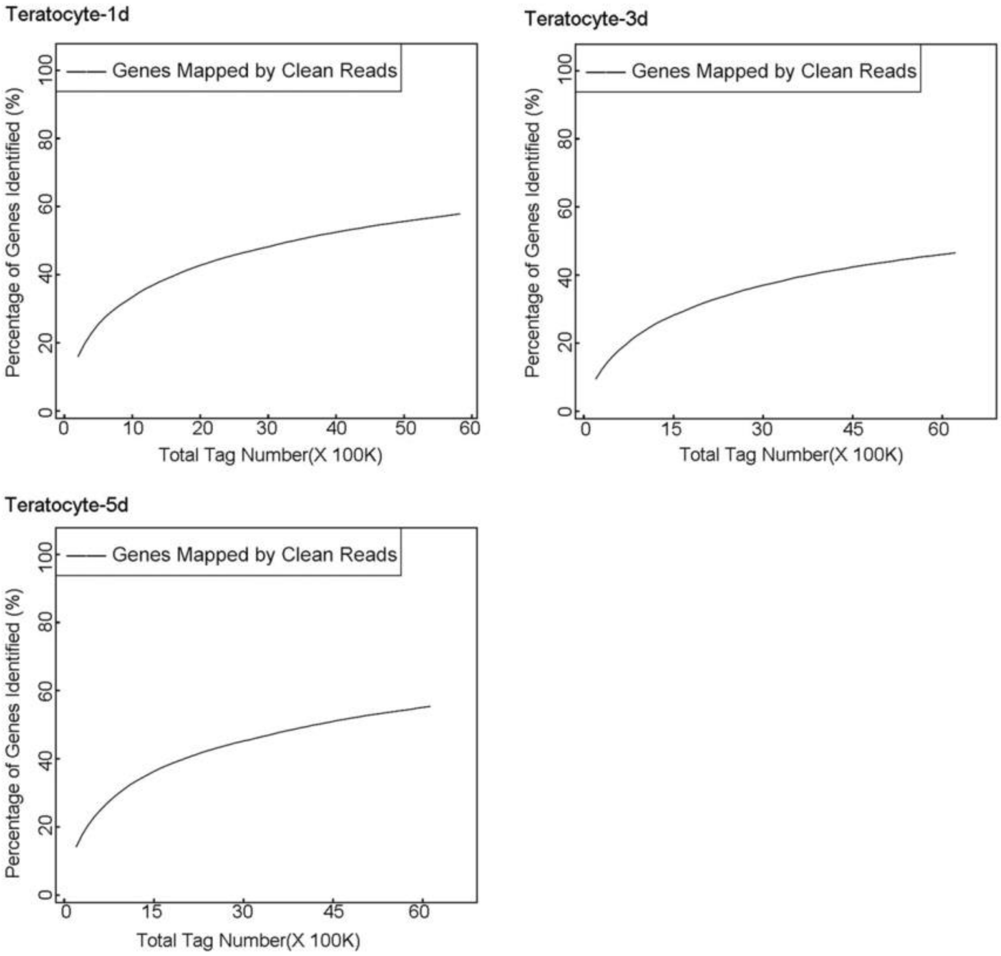

Figure S3. Saturation analysis of DGE sequencing. When the number of total reads reached 4 million the number of mapped genes almost ceased to increase.

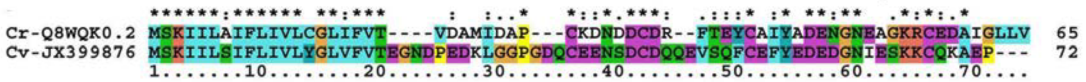

Figure S4. Amino acid sequences alignment of TVSP-8 (Cv-JX399876) with Vn4.6 (Cr-Q8WQK0.2)

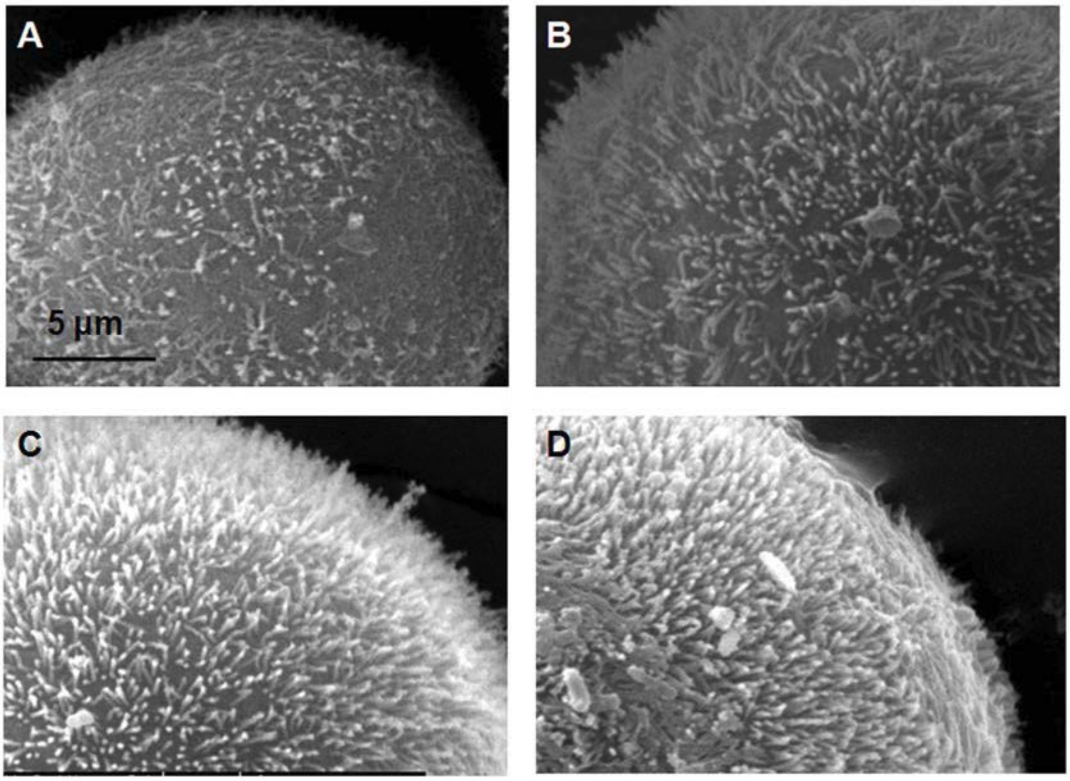

Figure S5. Microvilli on the surface of *Cotesia vestalis* teratocytes that were collected from hosts at 2 h (A), 1 day (B), 3 days (C) and 5 days (D) post-parasitism. Images are electron micrographs with the scale bar in (A) being the same for the images in (B-D).

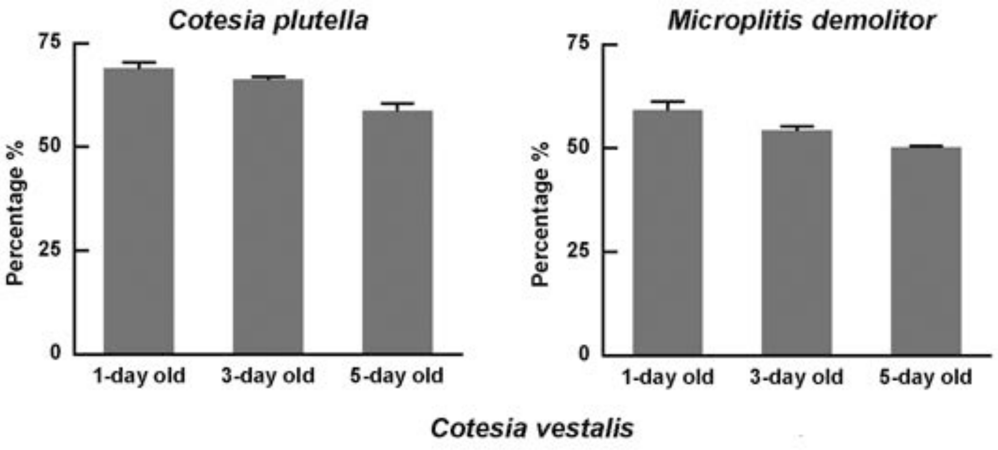

Figure S6. Matches of ESTs from the teratocyte transcriptomes of *Cotesia plutella* and *Microplitis demolitor* to transcriptomes of *Cotesia vestalis* teratocytes collected from hosts at 1, 3 and 5 days post-parasitism. The percentage of transcripts that are homologs of one another are indicated along the left axis with error bars indicating differences between the two sequencing libraries generated for each *C. vestalis* teratocyte library.
